# Neopterygian phylogeny: the merger assay

**DOI:** 10.1098/rsos.172337

**Published:** 2018-03-21

**Authors:** Adriana López-Arbarello, Emilia Sferco

**Affiliations:** 1 Department of Earth and Environmental Sciences, Palaeontology and Geobiology, and GeoBio-Center, Ludwig Maximilian University, Richard-Wagner-Strasse 10, 80333 Munich, Germany; 2 CICTERRA-CONICET-UNC, Av. Velez Sarsfield 1611, X0516GCA, Córdoba, Argentina

**Keywords:** Mesozoic, Actinopterygii, Neopterygii, Holostei, systematics, phylogeny

## Abstract

The phylogenetic relationships of the recently described genus ^†^*Ticinolepis* from the Middle Triassic of the Monte San Giorgio are explored through cladistic analyses of the so far largest morphological dataset for fossil actinopterygians, including representatives of the crown-neopterygian clades Halecomorphi, Ginglymodi and Teleostei, and merging the characters from previously published systematic studies together with newly proposed characters. ^†^*Ticinolepis* is retrieved as the most basal Ginglymodi and our results support the monophyly of Teleostei and Holostei, as well as Halecomorphi and Ginglymodi within the latter clade. The patterns of relationships within these clades mostly agree with those of previous studies, although a few important differences require future research. According to our results, ionoscopiforms are not monophyletic, caturids are not amiiforms and leptolepids and luisiellids form a monophyletic clade. Our phylogenetic hypothesis confirms the rapid radiation of the holostean clades Halecomorphi and Ginglymodi during the Early and Middle Triassic and the radiation of pholidophoriform teleosts during the Late Triassic. Crown-group Halecomorphi have an enormous ghost lineage throughout half of the Mesozoic, but ginglymodians and teleosts show a second radiation during the Early Jurassic. The crown-groups of Halecomorphi, Ginglymodi and Teleostei originated within parallel events of radiation during the Late Jurassic.

## 1. Introduction

The Neopterygii is the largest group of living vertebrates, including *ca* 32 650 valid species [1], the vast majority of which are teleosts. The origin of this important clade goes back to the Palaeozoic [2,3], but its most important radiation occurred in the early Mesozoic [3]. Ginglymodi, Halecomorphi and Teleostei are the three major clades currently recognized among crown-neopterygians (Actinopterygii: Neopterygii). Whereas for much of the second half of the last century halecomorphs and teleosts were regarded as sister-groups, to the exclusion of ginglymodians, recent morphological (e.g. [4,5]), molecular [6–10] and combined [11] studies support the monophyly of the Holostei: a major clade including both Ginglymodi and Halecomorphi, which is, in turn, the sister-group of Teleostei. Although there is clearly a strong molecular signal supporting the Holostei clade, there is still uncertainty in the morphological data (see review by [2]) and the Holostei hypothesis is yet being questioned by challenging new studies (e.g. [12]). The main problem of molecular phylogenies is the lack of important information due to the exclusion of fossils and, thus, the concomitant historical information on the stem lineage of the clade under study (past morphological disparity, morphological evolution that led to the modern forms and those morphologies that did not succeed, and the reasons why they did not, past events of diversification and the understanding of the context in which they occurred etc.). The missing information is especially important when extinct lineages are excluded as they might represent a significant expansion of the currently expressed morphospace of a lineage. Only the combination of both molecular and morphological data leads to optimal phylogenetic results: a Total Evidence approach [13–15].

Including fossil taxa in Total Evidence analyses is necessary not only to achieve a solid pattern of neopterygian phylogenetic relationships, but also to fully understand the macroevolutionary processes that led to the near extinction of the holosteans along with the peerless radiation of teleosts (see Hunt & Slater [16] for a review of the advantages of including fossils in phylogenetic analyses). The main obstacle for the Total Evidence method is the usual large amount of missing data [17–19]. It is known that the amount of morphological data for fossil taxa depends on the quality of preservation (in particular, soft tissues are only exceptionally preserved), which, together with the lack of molecular information, leads to a very large proportion of missing data in Total Evidence matrices. However, the effects of missing data in these analyses have been investigated by Guillerme & Cooper [20,21], who demonstrated that the amount of missing data in fossil taxa is not problematic when there are enough morphological characters and enough morphological information of living taxa in the data matrix. Fortunately, this is the case in holosteans (see below).

Dramatically, in contrast to the *ca* 32 640 species of living teleosts, non-teleost neopterygians are currently represented by only seven species of gars (Ginglymodi: Lepisosteiformes) and the bowfin, *Amia calva* (Halecomorphi: Amiiformes). This tremendous asymmetry in the number of recent representatives of the crown-neopterygian lineages does, by far, not reflect the situation during the early Mesozoic. During the Triassic–Jurassic, the diversity of ginglymodians and halecomorphs probably equated or even exceeded that of teleosts [22]. Numerous studies have been dedicated to the morphology of living gars, *Amia* and their direct fossil relatives (see [5,23] and literature cited therein) and, although the morphology of many fossil holosteans is still poorly known, numerous fossil holostean and teleost taxa, several of them representing extinct lineages, have been studied in detail and included in cladistic analyses during the last decades (e.g. [24–40]). These studies represent important steps towards a Total Evidence analysis of the Neopterygii, in which missing information should not be problematic because the fossil taxa will be properly anchored in the tree thanks to the available molecular and morphological information on the living representatives [20,21]. However, more taxa and characters are needed and a lot of work remains to be done in this direction.

Triassic neopterygians are particularly interesting for several reasons. Most recent molecular phylogenies predict divergence dates during the Devonian (394–290 Ma) for the Neopterygii, and during the Carboniferous–Permian for the Holostei (312–245 Ma) and Teleostei (333–250 Ma) (data from Sallan [2]: table 1). On the other hand, the earliest certain representative of the lineage leading to crown-Neopterygii is known from the Permian, while the earliest members of the holostean and teleost total groups are Triassic [3]. Even if future palaeontological discoveries might fill the gap between the estimated divergence dates and the age of the currently oldest known fossils, the fossil record shows that the first important radiation of the Neopterygii occurred during the Triassic [3]. Triassic neopterygians further present conflicting combinations of morphological features that have been proposed as synapomorphies for one or the other of the three crown-neopterygian clades (e.g. [37,41,42]). Including Triassic taxa in phylogenetic studies of Neopterygii is thus necessary, even if they might increase the level of homoplasy [43].

Within this context, the present contribution is dedicated to explore the phylogenetic relationships of the recently described Middle Triassic neopterygian genus ^†^*Ticinolepis* López-Arbarello, Bürgin, Furrer and Stockar, 2016 [37]. This is one of the taxa showing a mixture of morphological features typically ascribed to Ginglymodi or Halecomorphi, together with other features observed only in teleosts [37]. For this reason, to study the relationships of ^†^*Ticinolepis*, we conducted a cladistic analysis based on the so far largest morphological dataset for fossil actinopterygians, including representatives of the three crown-neopterygian clades and merging the lists of characters from previously published systematic studies of neopterygians together with newly proposed characters. Our list of characters is not just the simple assemblage of characters taken from previous works. We have carefully revised previous lists of characters and the hypotheses of primary homology behind them. We have merged and modified the definition of most characters to avoid problematic coding as discussed by Jenner [44] and Brazeau [45]. Our list of 339 morphological characters is not yet complete, more characters will hopefully be added in the future, but it is certainly a solid base to start working on the endless task of completing information seeking for the neopterygian evolutionary tree.

## 2. Material and methods

### 2.1. Taxonomic sampling and nomenclature

The investigation of the phylogenetic relationships of ^†^*Ticinolepis* was performed through a parsimony analysis of a matrix of 339 morphological characters scored for 99 species (92 extinct and 7 living taxa). All operational taxonomic units are species (electronic supplementary material, file S1).

According to previous phylogenetic hypotheses for crown-neopterygians (summarized by Friedman [3]) and considering the availability of published morphological information, the Early Triassic stem neopterygians ^†^*Australosomus kochi* Stensiö, 1932 [46], ^†^*Boreosomus piveteaui* Nielsen, 1942 [47], ^†^*Pteronisculus stensioi* (Nielsen, 1942) [47] and ^†^*Plesiofuro mingshuica* Su, 1993 [48], were chosen as out-group taxa. The in-group includes 36 ginglymodians (36%; two living species), 25 halecomorphs (25%; one living species), 29 teleosts (29%; four living species) and 5 taxa of uncertain relationships (the two species of ^†^*Ticinolepis* and three species of ^†^*Dapedium*).

Taxonomic names are used, proposed and/or defined according to the rules and recommendations of the International Code of Zoological Nomenclature (ICZN 2000) and in agreement with the PhyloCode [49]. Accordingly, the following clades are here recognized: total clade Neopterygii *sensu* Regan [50], crown-group Neopterygii *sensu* Patterson [51], Holostei *sensu* Grande [5], Ginglymodi *sensu* López-Arbarello [26], Halecomorphi *sensu* Patterson [52] and total clade Teleostei *sensu* de Pinna [53].

### 2.2. Character coding and scoring

López-Arbarello *et al*. [37] described the genus ^†^*Ticinolepis* from the Ladinian (Middle Triassic) of the Monte San Giorgio and discussed the resemblance of this fish with ginglymodians, but also with halecomorphs and teleosts. Owing to this mixture of morphological features, none of the available data matrices would have been adequate to explore the phylogenetic relationships of this genus because each of them was designed for cladistic analyses of the relationships within one or the other of the three main crown-neopterygian groups, and solving the systematic position of ^†^*Ticinolepis* required the inclusion of all of them. Compiling such a comprehensive data matrix needed the revision of all hypotheses of homology and the consequent redefinition of many characters and character states. The resulting data matrix includes a total of 339 morphological characters (see complete list of characters in electronic supplementary material, file S2). Among them, 76 are newly defined and the remaining characters are the result of merging and modifying characters from most previous cladistic analyses of crown-neopterygians (see complete list below). Emended definitions of characters are based on a thorough revision of primary homology hypotheses, taking special care to avoid those definitions that imply the use of unspecified absence states [44] as well as repeated absences, pseudo-ordering and compound characters [45].

Whenever possible, character scoring was based on direct examination of specimens or on descriptions in the literature if the material was not available to us (see list of examined material and literature in electronic supplementary material, file S1). The data matrix was prepared with Mesquite v. 3.31 [54]. There are 127 multistate characters with an average of 3 and a maximum of 9 character states (ch. 124: Largest infraorbital bone). The matrix contains average proportions of 37% and 35% missing data and 7% and 10% inapplicable scorings for the taxa and characters, respectively. The maximal amount of missing data is 75% for the ginglymodian ^†^*Cammerichthys lunae*, which is only known from a partially complete skull, and 92% for character 211 ‘Infrapharyngobranchial tooth plates’, which is a feature rarely observed in fossils. The maximal amount of inapplicable scorings is 27% for ^†^*Australosomus kochi* among the out-group taxa and 15% for ^†^*Pachycormus macropterus* within the in-group and 92% for character 193 ‘anterior notch of preopercle’, which is a feature unique of a few pholidophoriform teleosts. Despite the high amount of missing data in certain characters (notably higher for endocranial features), all characters are parsimony informative.

Autapomorphic character states are not unusual in our data matrix. These states are not informative for the tree search, but are informative concerning the amount of homoplasy. The only valid alternative instead of scoring an autapomorphic character state would be to score the feature ‘inapplicable’ for the taxon in question. However, it is reasonable to keep the autapomorphic character states because they will probably be informative for other possible studies (e.g. disparity analyses), which might be based on these data in the future.

### 2.3. Cladistic methodology

The cladistic analyses were performed with TNT [55] under equal and implied weighting [56]. In contrast to the commonly used equal weighting analyses, where all characters are given the same weight, implied weighting analyses were designed to down-weight characters according to their level of homoplasy in order to obtain a hypothesis that maximizes the influence of the more reliable characters at the expense of the more homoplastic ones. In these procedures, the fit of each character is calculated with a concave function of its number of extra steps (i.e. the more homoplastic, the less fit) and the preferred tree(s) are those which maximize the total fit. The weighting strength (i.e. how strongly homoplastic characters are down-weighted) is determined by using different concavity constant (*K*-values). We have used several concavity constants to explore variations in the resulting pattern of relationships.

In both equal weighted and implied weighting analyses, tree search was performed with the traditional search option of TNT v. 1.1 [55,57] applying random addition sequence (RAS) and tree bisection reconnection (TBR) through 1000 replicates keeping 10 trees per replicate. TBR was applied to all the trees retained in memory and trees are rooted in ^†^*Pteronisculus stensioi*. Most characters are unordered; three characters are ordered (chs. 32, 289 and 325). Branch support was evaluated also with TNT applying bootstrap expressed as values of GC (groups present/contradicted) through 10 000 replicates and calculating Bremer decay indexes for each node. Support measurements were calculated for implied (*K* = 8 and *K* = 3) and equal weighting analyses.

Considering that, within Teleostei, both Teleocephala (*sensu* de Pinna [53]) and Clupeocephala are well-corroborated groupings, demonstrated in several occasions by molecular and morphological phylogenetic analyses [4,10,11], but our dataset was not specifically designed to test the relationships among recent teleosts, we performed the analyses with constraints enforcing the monophyly of these two main clades of living teleosts. The phylogenetic data are freely available in the supplementary files, in Dryad Digital Repository (https://doi.org/10.5061/dryad.2tp53gr) [58] and in MorphoBank (www.morphobank.org; Project 2196).

The distribution of characters was analysed using the ‘trace character history’ option in Mesquite v. 3.31 [54] and both accelerated (ACCTRAN) and delayed transformation (DELTRAN) methods for character optimization were run with PAUP v.4.0a for Macintosh. Depending on the optimization, some character changes will be synapomorphic for a certain clade under ACCTRAN (opting for the earliest possible transformation and preferring reversal over convergence), but not if DELTRAN is assumed (opting for the latest possible transformation and preferring convergence over reversals), and vice versa [59,60]. These synapomorphies depend on the optimization and are thus ambiguous. Other character changes are unambiguous because they are synapomorphic under both delayed and accelerated transformation, i.e. both optimization methods will set the character change at the same node. Note that unambiguous character transformation only refers to the node in question and does not exclude that character transformations might be homoplastic at other nodes. Agnarsson & Miller [61] thoroughly discussed the advantages and disadvantages of ACCTRAN and DELTRAN optimizations and concluded that there are no theoretical reasons to prefer one or other method and both methods should be applied and considered to achieve the optimal understanding of character evolution. However, for this work, representing the first cladistic analyses of the three main crown-neopterygian lineages taken together, the resulted phylogenetic hypothesis is not robust enough and we do not want to hasten conclusions about character evolution at this early stage of research. Therefore, only the unambiguous synapomorphies were taken into account, discriminating between unique and non-unique synapomorphies.

## 3. Discussion of characters

All 339 characters are listed in the electronic supplementary material, file S2. Unless the definition of a character is self-explanatory or has been explained in some previous work, the characters are accompanied with additional explanatory paragraph and/or figure. Additionally, some characters need thorough discussions, which are included in this section.

### 3.1. Relative position of the dorsal fin

The relative position of the dorsal fin has been included in one or more characters and expressed in different ways by many authors. In many cases, attempts have been made to represent the position of the dorsal fin relative to the body in general (e.g. anterior, in the middle and posterior), which has important biomechanical implications. However, such characters are usually very vaguely defined and they should rather be expressed quantitatively, although this is also problematic. Alternatively, using the pelvic and anal fins as landmarks is a useful tool to represent the relative position of the dorsal fin in the body of actinopterygians, even in those fishes with extremely elongated bodies (e.g. ^†^*Saurichthys*, ^†^*Aspidorhynchus*, Belonidae). Therefore, we adopted the multistate character proposed by López-Arbarello ([26]: ch. 1), which was modified to encompass the variation included in our dataset.

A character used by several cladistic analyses ‘Dorsal and anal fins posteriorly placed’ (e.g. [62]: ch. 77, [63]: ch. 99, [64]: ch. 95, [65]: ch. 94, [66]: ch. 91), is an example of a vaguely defined character, and another character ‘Dorsal fin origin anterior to that of pelvic fin’ (e.g. [62]: ch. 78, [63]: ch. 100, [64]: ch. 96, [65]: ch. 95, [66]: ch. 92) would imply unspecified states [44] for our dataset, both in the presence, embracing the conditions described in our character states 2 and 3 (dorsal fin extending anterior to opposite of insertion of pelvic fins, or originating anterior to insertion of pelvic and extending opposite to anal fins), and the absence, embracing the conditions described in our character states 0 and 1 (dorsal fin originating posterior to insertion of pelvic and extending backwards not beyond middle of anal fin, or originating approximately at the level of the origin of the anal fin and extending opposite to it) and 4 and 5 (dorsal fin originating posterior to the origin of anal fin, or originating posterior to insertion of pelvic and extending backwards up to end of anal fin). Another binary character used in Arratia ([29]: ch. 118), ‘Dorsal fin placed posteriorly, closer to caudal fins than to pelvic fins’, or the reworded version of this character in Arratia ([39]: ch. 136) embraces the conditions described in our states 1 and 4 in one character state and the conditions in our states 0, 2, 3 and 5 in the other character state. Cavin ([67]: ch. 41) ‘Both dorsal and anal fins well developed, ending posteriorly close to the caudal peduncle’ is a compound character [45] involving unspecified absence of the two types discussed by Jenner [44].

### 3.2. Type of scales

Ideally one would like to distinguish the palaeoniscoid and lepisosteoid types of the ganoid scales because both lepisosteoid and elasmoid scales are homologous to palaeoniscoid scales, but it is possible that these two types evolved independently [68,69]. However, the transition from one type to the other is gradual and might occur within a single scale [70]. Even when histological information is available for some taxa, one would need to trace the presence/absence of dentine in scales from different regions of the body of each fish specimen, which would probably result in heterogeneous patterns. Therefore, pending a more rigorous and detailed analysis of the distribution of the different types of scales in Mesozoic actinopterygians, we apply here the simply morphological distinction between ganoid, amioid and cycloid scales (ch. 4).

Other authors use independent a/p characters for each lepisosteoid, amioid and cycloid scale types (e.g. [66]: chs. 140–142), which is methodologically incorrect because these characters are not mutually independent and involve unspecified absence states [44]. Alternatively, the lepisosteoid, amioid and cycloid scale types represent different states of single multistate characters in several recent phylogenetic analyses (e.g. [29]: ch. 156, [35]: ch. 175), which is a valid option, but the scoring of lepisosteoid type of scales for a certain taxon is questionable due to the lack of histological information (e.g. several Triassic teleosts in [29,39]).

**Figure 1.**
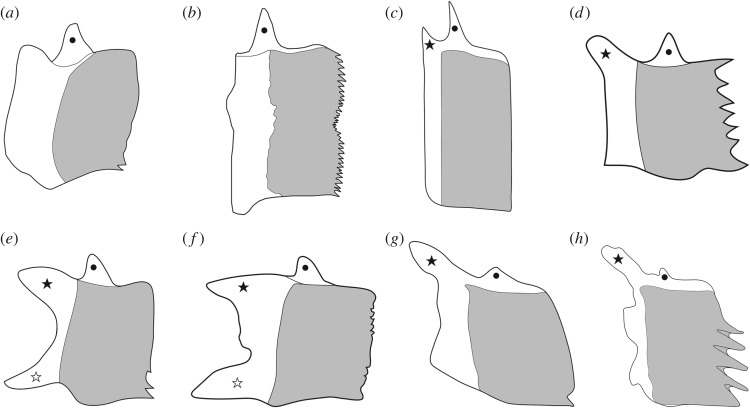
Morphology of ganoid scales. (*a*) ^†^*Sangiorgioichthys sui*, reconstruction based on GMPKU-P-1642; (*b*) ^†^*Siemensichthys macrocephalus* reconstruction based on [60]: fig. 10B; (*c*) ^†^*Australosomus kochi* reconstruction based on [45]: text-fig. 57C; (*d*) ^†^*Semionotus bergeri* reconstruction based on NMC 15128a; (*e*) ^†^*Callipurbeckia minor* reconstruction based on NHMUK PV P8047; (*f*) ^†^*Scheenstia mantelli* reconstruction based on NHMUK PV 2397 and 4916; (*g*) *Lepisosteus* sp. reconstruction based on MB.f.18498; (*h*) ^†^*Dentilepisosteus laevis* reconstruction based on MPSC 901 in [5]: fig. 109B. Black circle, dorsal peg for the peg-and-socket articulation of adjacent scales; black star, anterodorsal process and white star, anteroventral process, both for the longitudinal articulation of adjacent scales.

Among the ganoid scales, we distinguish different morphotypes (figure 1) according to the shape of the posterior margin of the scales (ch. 6) and the presence/absence of different articulatory processes (chs. 7–9). Most ganoid scales of actinopterygians are articulated through the so-called peg-and-socket articulation, consisting of a dorsal spine-like peg protruding from the dorsal border of the scale, which fits in a conical socket excavated in the medial surface of the scale (figure 1*a–e*). In some ginglymodians, the scales have only very reduced pegs and sockets, or this articulating structure is completely absent (figure 1*f*–*h*). The reduction to the complete absence of a peg-and-socket articulation occurs independently in the scales of several lepisosteiform taxa (^†^*Lepidotes*, ^†^*Scheenstia*, ^†^*Dentilepisosteus*, ^†^*Masillosteus janeae*, *Atractosteus spatula*). In addition to the peg-and-socket articulation explained above, the scales of many ginglymodians (e.g. the species of ^†^*Lepidotes* and ^†^*Scheenstia*, the callipurbeckiids) also form a rostro-caudal or longitudinal articulation consisting of two anteriorly oriented processes protruding from the anteroventral and anterodorsal corners of the scale (figure 1*e*,*f*). These processes can be as strong as or stronger than the peg for the dorsoventral articulation, but they do not fit into sockets. In several taxa (e.g. ^†^*Semionotus*, ^†^*Paralepidotus*, ^†^*Pliodetes* and the gars), the ventral anterior process is poorly developed and there is a strong dorsal anterior process (figure 1*d*,*g*,*h*). The scales of several other taxa (^†^*Australosomus*, ^†^*Archaeosemionotus*, ^†^*Boreosomus*, ^†^*Obaichthys*, ^†^*Ophiopsis*, ^†^*Panxianichths*, ^†^*Plesiofuro*) only have a small anterodorsal process (figure 1*c*). It is important to note that many authors (e.g. [71]: ch. 36, [72]: ch. 59, [73]: ch. 38) score the absence of peg-and-socket articulation for taxa with amioid or cycloid scales. However, the peg-and-socket articulation is a feature of the ganoid scales and the character is not applicable (–) for taxa with other scale types.

### 3.3. Endocranial fossae

Two main fossae have been identified in the otico-occipital region of the neopterygian endocranium: the post-temporal fossa and the fossa bridgei. However, according to Bjerring [74], the terms ‘post-temporal fossa’ and ‘fossa bridgei’ have been misunderstood and erroneously applied to different fossae in the braincase of the actinopterygians. After thorough discussions, Bjerring [74] proposes a basic scheme including five pairs of endocranial depressions and discusses hypotheses of homology for each of them. His arguments are sound and clearly presented and we thus adopt his ideas as hypotheses of primary homology for the definition of the following two characters. Understanding the homologies of the endocranial fossae in actinopterygians is difficult and we strongly recommend reading Bjerring [74] for a complete argumentation of his hypotheses of homology, which are being followed in this work.

It is important to note that Allis ([75]: p. 8) distinguishes the ‘*temporal fossa* of fishes is a hole formed by the more or less complete roofing, by dermal bones, of the *temporal groove* on the dorsal surface of the primordial cranium’. More correctly, Bjerring [74] and other authors (e.g. [76,77]) use the term fossa referring to the endocranial depressions.

As defined by Bjerring ([74]: p. 232), the fossa supra-auditiva ‘is a depression in the external surface of the otic region of the endocranium, situated dorsal to the lateral semicircular duct and lateral to the anterior and posterior semicircular ducts'. It has been labelled in different ways by different authors (e.g. in *Amia*: ‘post-temporal fossa’ by Patterson [51,78], or ‘fossa bridgei’ by Jarvik [79]). The fossa supra-auditiva is either present or the condition is unknown in the taxa included in our data matrix, so its presence is uninformative for our analysis.

### 3.4. Foramen for the glossopharyngeal nerve (IX)

The braincase in ^†^*Pteronisculus*, ^†^*Australosomus* and ^†^*Boreosomus* ossifies in a few pieces, which are not directly comparable with the individual bones that form in neopterygians. In these fishes, the glossopharyngeal nerve exits the ventrolateral wall of the otic region within a groove for the vena jugularis, which is called the jugular depression. The IX-foramen in ^†^*Pachycormus macropterus* is in the opisthotic, also in the anterior end of the jugular groove (Mainwaring AJ. 1978 Anatomical and systematic review of the Pachycormidae, a family of Mesozoic fossil fishes. Unpublished: Westfield College: p. 54), and this condition is autapomorphic for this taxon in our data matrix.

The passage of the glossopharyngeal nerve through the otic walls of the neurocranium of *Amia* is described in detail by Allis ([80]: p. 683): ‘It passes above the ramulus papillae lagenae acustici, under the ramulus ampullae posterioris acustici, between the sacculus and the sinus utriculi posterior … and issues from the cranium by its foramen …, which lies immediately behind the hind edge of the petrosal, in the angle between that edge and the ventral edge of the posterior process of the bone. The foramen lies entirely in the cartilage of the cranium, but its front and upper edges are formed by the petrosal.’ The shape of the prootic and intercalar bones in ^†^*Calamopleurus cylindricus* is remarkably similar to the condition in *Amia calva* (compare [23]: figs 24 and 303) and there is no evidence for the IX-foramen in any of the bones at the lateroventral wall of the otic region, which is mostly unossified. Thus, we assume that the glossopharyngeal nerve in ^†^*Calamopleurus cylindricus* exits the braincase in the same way as in *Amia calva*. Similarly, the same condition is interpreted for all those amiine halecomorphs, in which this region of the braincase is well preserved. In *Amia calva* and ^†^*Calamopleurus cylindricus*, there is a foramen in the posterior region of the intercalar ([23]: figs 24C, 28 and 303), which, according to Allis ([80]: pl. 21) corresponds to the exit of the supratemporal branch of the glossopharyngeal nerve.

The foramen for the glossopharyngeal nerve (IX) pierces the prootic bone in ginglymodians, most non-amiinae halecomorphs and early Mesozoic teleosts such as aspidorhynchiforms, ^†^*Siemensichthys macrocephalus*, ^†^*Dorsetichthys bechei* and ^†^*Leptolepis coryphaenoides*. Instead, in more derived teleosts, like ^†^*Tharsis dubius* and teleocephalans, the IX-foramen is in the exoccipital. Among the taxa included in our analysis, ^†^*Ionoscopus cyprinoides* is the only species in which the IX-foramen is enclosed at the anterior end of the intercalar [81].

Patterson & Rosen ([82]: ch. 26) defined an a/p character for the presence of the ‘foramen for the glossopharyngeal nerve (IX) in exoccipital rather than in prootic’, which was later used in Arratia ([62]: ch. 20 App. 3) and, with slightly rephrased definition in several cladistic analyses (e.g. [65]: ch. 20, [66]: ch. 19, [29]: ch. 32, [39]: ch. 37). All these examples include unspecified absences [44] with *Amia* scored equal to *Lepisosteus* and early Mesozoic teleosts with the absence character state.

### 3.5. Foramen for the vagus nerve (X)

The fissura otico-occipitales is open in the braincase of our out-group taxa ^†^*Pteronisculus*, ^†^*Australosomus* and ^†^*Boreosomus*, and in ^†^*Watsonulus eugnathoides*. Therefore, there is no foramen for the vagus nerve (X), which exits through the fissure in these fishes. The otico-occipital fissure is closed in more derived actinopterygians, and the vagus nerve exits the cranium through a foramen through one of the bones of the occipital region. In most halecomorphs and most basal teleosts, the X-foramen is surrounded by the intercalar and the exoccipital bones. In ginglymodians, the halecomorphs ^†^*Calamopleurus cylindricus* and ^†^*Oshunia brevis*, and in the teleosts ^†^*Catervariolus*, ^†^*Varasichthys* and the Teleocephala, the X-foramen is completely enclosed by the exoccipital. ^†^*Pachycormus macropterus* presents a condition unique among the studied taxa, in which the vagus nerve exits the braincase through a foramen between the opisthotic and the intercalar.

The position of the foramen for the vagus nerve has been used for cladistic analyses in variably coded characters. Our coding (character 24) is taken from Gardiner *etal*. ([83]: ch. 5 App. 1) and Hurley *etal*. ([4]: ch. 9), who use a multistate character representing the position of the exit of the vagus nerve. Their character state 0, ‘anterior to exoccipital’ is equivalent to our character state 0 ‘through the fissura otico-occipitalis’, though we modified its formulation to make it more precise and descriptive. Their character state 1 ‘lateral outgrowths from intercalar form lateral margin’ is not represented in our data matrix. Their character state 2, expressed as ventral outgrowths from intercalar lateral margin ‘enclose ventral margin’ in [83], or ‘enclose dorsal margin’ in Hurley *et al*. [4] is equivalent to our character state 1 ‘between intercalar and exoccipital’, which is more flexible because the shapes of the intercalar outgrowths vary intra- and interspecifically and, thus, they contribute in different ways to the rim of the X-foramen. Finally, their character state 3 ‘enclosed by exoccipital’ is the same as our character state 2. The character state 1 of these authors is problematic because it was scored for ^†^*Pachycormus* in Gardiner *et al*. ([83]: ch. 5 App. 1), which does not agree with Mainwaring (Mainwaring AJ. 1978 Anatomical and systematic review of the Pachycormidae, a family of Mesozoic fossil fishes. Unpublished: Westfield College), or for *Polypterus* in Hurley *et al*. ([4]: ch. 9), but the bone enclosing the X-foramen in this fish is not the intercalar but opisthotic according to Allis [84] and Claeson *et al*. [85].

Most other authors (e.g. [86]: ch. 3, [71]: ch. 11, [63]: ch. 25, [65]: ch. 21, [87]: ch. 2, [26]: ch. 3, [29]: ch. 33) only include an a/p character coding the presence of the X-foramen in the exoccipital (our state 2), thus producing an unspecified absence [44] when equally scoring *Amia* (here state 1), ^†^*Watsonulus* (here state 0) and sometimes also ^†^*Pachycormus* (here state 3) with the state ‘absence’.

### 3.6. Intercalar

**Figure 2.**
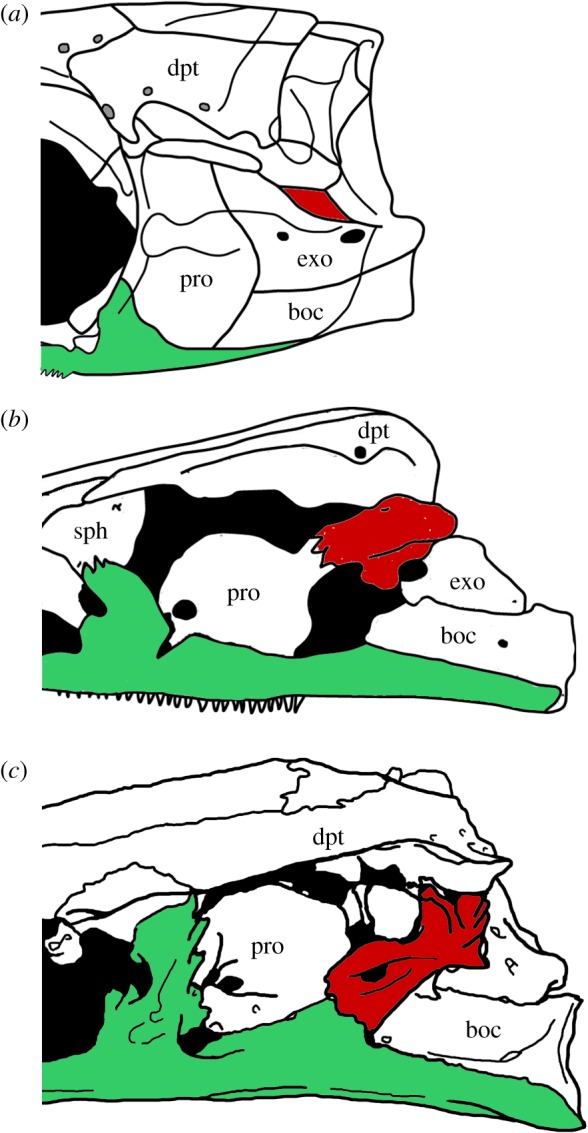
Extent of the dermal outgrowths of the intercalar bone. (*a*) small dermal component without extensive outgrowths in ^†^*Ichthyokentema purbeckensis*, reconstruction based on [90]: fig. 2; (*b*) dermal outgrowths contacting the prootic in *Amia calva*, reconstruction based on AMNH 90970 SD in [23]: fig. 23B; (*c*) extensive outgrowths contacting the prootic and parasphenoid in ^†^*Ionoscopus cyprinoides*, reconstruction based on NHMUK PV 37795a in [78]: fig. 1A. Intercalar bone painted in red, parasphenoid painted in green. Abbreviations: boc, basioccipital; dpt, dermopterotic; exo, exoccipital; pro, prootic; sph, sphenotic.

According to Patterson ([78]: p. 315), the intercalar bone is homologous to the cranio-spinal process present in the out-group taxa ^†^*Pteronisculus*, ^†^*Australosomus* and ^†^*Boreosomus* [47,88], and in the halecomorph ^†^*Watsonulus eugnathoides* [89] and the ginglymodian ^†^*Ticinolepis crassidens* [37]. Patterson further interpreted that a dermal component develops from this endochondral core, which is homologous with the fully dermal intercalar in teleosts above the level of ^†^*Leptolepis coryphaenoides*. Following Patterson's hypothesis of homology, we coded the a/p character 33 for the presence of an intercalar (chondral or dermal), and characters 34 and 35 representing different conditions observed regarding the development and relationships of the dermal component of the intercalar. In our character 33, we distinguish the intercalar without extensive dermal outgrowths (state 0; figure 2*a*) from the intercalar with outgrowths contacting the prootic (state 1; figure 2*b*) or with extensive outgrowths contacting the prootic and parasphenoid (state 2; figure 2*c*). We do not code the disappearance of the chondral component of the intercalar because it is almost impossible to evaluate this feature in fossils.

Olsen & McCune ([86]: chs. 9, 21) and Brito ([71]: chs. 9, 10) score the presence/absence of a chondral intercalar and the presence/absence of an intercalar with dermal outcrops in two independent characters. This way, the complete absence of an intercalar (e.g. *Lepisosteus*) is incorrectly scored the same as the presence of a dermal intercalar (e.g. *Amia*) in their state ‘absence of a chondral intercalar’.

### 3.7. Hyomandibular facet

There is much variation regarding the bones involved in the articulation of the hyomandibula with the neurocranium, so we have defined character 39, including six states summarizing the observed variation. In ^†^*Caturus furcatus*, there is a single bone forming the lateral wall of the endocranium, which has been identified as a large prootic [91], but might be the result of the fusion between the prootic and opisthotic [92]. Owing to this uncertainty, this character is scored as ‘missing data’ (?) for ^†^*Caturus furcatus*. In most other halecomorphs, the facet for articulation of the hyomandibula is formed in cartilage (ch. 39[0]) [23]. The cartilage is not preserved in fossils, but the condition can be inferred. Among the taxa studied, only in ^†^*Macrepistius* the facet for the hyomandibula is completely included in the opisthotic (ch. 39[1]) (interpretation of this bone according to Maisey [81]). The condition in ^†^*Pachycormus macropterus*, in which all the bones of the lateral wall of the neurocranium are involved in the formation of the hyomandibular facet (ch. 39[2]) (Mainwaring AJ. 1978 Anatomical and systematic review of the Pachycormidae, a family of Mesozoic fossil fishes. Unpublished: Westfield College), is unique among the studied taxa. Among teleosts, the facet is formed by the sphenotic, pterotic and prootic bones (ch. 39[3]) in the more basal forms and in *Brycon* and *Hiodon*, but the prootic is not involved in the facet in the other teleost taxa in our data matrix. The facet is formed by the pterotic and sphenotic (ch. 39[5]) in most of these species, or by the pterotic only in ^†^*Notelops* and ^†^*Ebertichthys* (ch. 39[4]).

Several authors have coded the inclination of the hyomandibular facet in variably defined morphological characters. For example, Gardiner & Schaeffer ([93]: ch. 20) coded the presence/absence of horizontal facets and Coates ([94]: ch. 27) coded posteroventrally versus ventrally oriented facets. The common problem with previous attempts to distinguish between different degrees of inclination of the hyomandibular facet is that there is no clear indication of the reference against which the measurement should be taken. To solve this problem, for our character 40 ‘Orientation of hyomandibular facet respect to the parasphenoid axis’, we have taken as reference the orientation of the orbital portion of the parasphenoid, which is the most constant structure reflecting the anteroposterior axis of the head in most actinopterygian braincases. On the contrary, the postorbital portion of the parasphenoid is very variable, not only in extension, but also in orientation.

**Figure 3.**
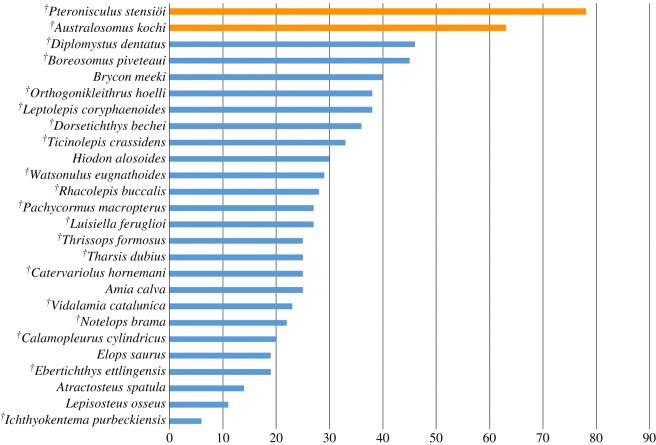
Bar chart representing the values of the angle between the main axis of the hyomandibular facet and the longitudinal axes of the orbital portion of the parasphenoid (horizontal axis) obtained for 26 studied taxa.

To define the character states, we have been able to measure the angle between the main axis of the hyomandibular facet and the longitudinal axes of the orbital portion of the parasphenoid in 26 taxa. This information is represented in figure 3, which shows a gradual change for angles below 50° followed by a significant gap between 50° and 60°. This pattern agrees with previous ideas concerning a significantly inclined versus an almost horizontal hyomandibular facet, the latter only present in neopterygians [93]. Our character states are thus defined around this gap. However, this hypothesis should be tested in a more comprehensive analysis of the variation of this feature among non-neopterygian actinopterygians, which might reveal facets oriented with angles larger than 50°, or they might refute our hypothesis with values filling the gap observed in this study. In such a case, this feature should best be treated as a continuous character.

The hyomandibular facet is not directly observable in several taxa because it is formed in cartilage (see above), or it is hidden by other bones in fossils. In such cases, scoring the variation in the orientation of the hyomandibular facet is still possible through a rough estimation within the ranges proposed in the two character states when the parasphenoid and the head of the hyomandibula are well visible and preserved *in situ*.

An alternative coding proposed by Xu & Wu ([72]: ch. 36) is a character including three states based on ranges of angular values representing the orientation of the suspensorium. However, the orientation of the suspensorium is independent and does not directly reflect the inclination of the hyomandibular facet (see Gardiner & Schaeffer [93]: pp. 145–146). Such a variation would thus represent a separate character, but we think that the inclination of the suspensorium is not an independent feature but a direct consequence of the position of the lower jaw articulation (character 69).

### 3.8. Dermosphenotic and dermal component of the sphenotic

The presence of a dermal component of the sphenotic is discussed by Bartram [95] and this feature has been used in several cladistic analyses (e.g. [5]: ch. 23, [26]: ch. 7, [72]: ch. 16, [29]: ch. 10, [27]: ch. 34, [33]: ch. 75). The character is discussed by Grande ([5]: p. 760), who clearly distinguishes the condition in the ^†^obaichthyids, in which the sphenotic is fused to the dermosphenotic, but scores the presence of a sphenotic dermal component for these taxa. However, the dermal component of the sphenotic in other neopterygians is an ossification independent from the dermosphenotic. The best example of the independence of these two ossifications is found in the living gars, in which the dermosphenotic and the exposed portion of the sphenotic are well separated by suborbital bones (see examples in Grande [5]). Therefore, the two conditions are not homologous and are thus here coded as different characters (chs. 41 and 42, respectively).

The fusion between dermosphenotic and sphenotic is also equated with the dermal component of the sphenotic and incorrectly scoring the presence of this latter feature in ^†^*Obaichthys* and ^†^*Dentilepisosteus* in Arratia ([29]: ch. 10), Cavin *et al*. ([27]: ch. 34), Bermúdez-Rochas & Poyato-Ariza ([96]: ch. 7), and other papers using those data matrices.

### 3.9. Lateral dermethmoids

**Figure 4.**
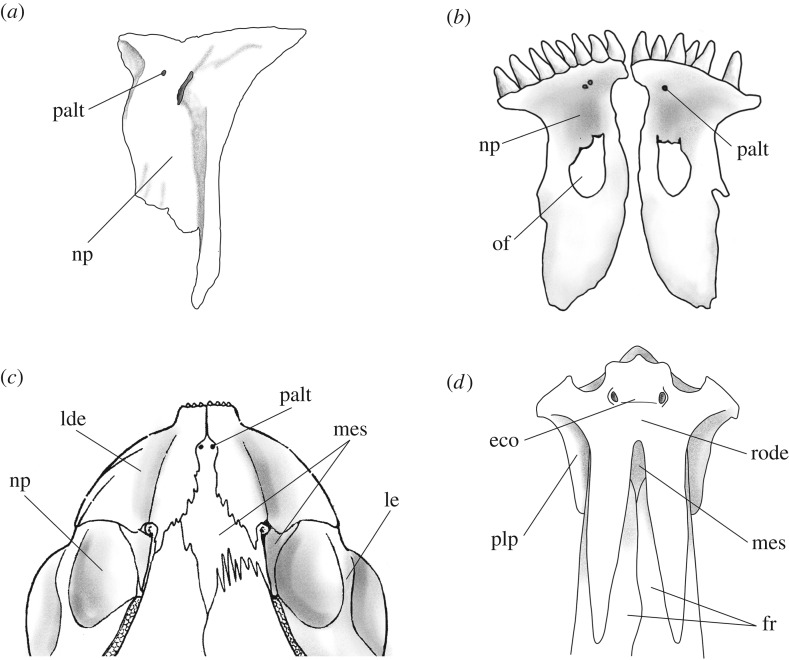
Lateral dermethmoids: (*a*) forming small nasal processes of the premaxilla, only partially surrounding the olfactory foramen, in ^†^*Ticinolepis longaeva*, line drawing of the premaxilla of MCSN 8007; (*b*) forming large nasal processes of the premaxilla, enclosing the olfactory foramen, in *Amia calva*, reconstruction based on AMNH 90970 SD in [23]: fig. 42A; (*c*) forming toothed dermethmoids in ^†^*Siemensichthys macrocephalus*, reconstruction based on [74]: fig. 145; (*d*) forming part of a compound mesethmoid with chondral and dermal components in ^†^*Tharsis dubius*, reconstruction based on [74]: fig. 130a. Abbreviations: eco, ethmoidal commissure; fr, frontal bone; lde, lateral dermethmoid; le, lateral ethmoid; mes, mesethmoid; np, nasal process of the premaxilla; of, olfactory foramen; palt, foramen for terminal branch of palatine nerve; plp, postero-lateral process of lateral dermethmoid; rode, rostrodermethmoid.

Characters 47 and 48 are based on the hypotheses of homology between the rhinal bone, the nasal process of the premaxilla in halecomorphs and ginglymodians (figure 4*a*,*b*), and the lateral dermethmoids of teleosts, which are discussed and summarized in López-Arbarello ([26]: p. 38) and thoroughly discussed in Patterson [78]. According to Patterson ([51]; [78]: p. 510) ‘the pholidophorid premaxilla and lateral dermethmoid are together the exact topographic homologues of the premaxilla and its nasal process’ and the lateral dermethmoids in ^†^*Siemensichthys macrocephalus* (figure 4*c*) or ^†^*Dorsetichthys bechei* are homologous of the lateral dermethmoid component of the compound mesethmoid of ^†^*Leptolepis coryphaenoides* (figure 4*d*) and more derived teleosts.

According to this hypothesis, among the taxa included in this analysis, the lateral dermethmoids might be present or absent (ch. 47). If present, they might be forming the nasal process of a single premaxilla (figure 4*a*,*b*) or they might be toothed and separated from a pair of small premaxillae (figure 4*c*), or they might be forming part of a compound mesethmoid ossification including chondral and dermal components (figure 4*d*) (ch. 48).

### 3.10. Parasphenoid processes

Two ascending processes are identified in early actinopterygians: processus ascendens anterior and posterior (e.g. [46,47,77,79,97–99]). The processus ascendens posterior is more widely present among actinopterygians and it is usually referred to as the ascending process (e.g. [23,52,78,100–102]). The processus ascendens anterior is related to the processus basypterigius of the neurocranium ([46]: p. 273; [47]: pp. 105, 324), and it is usually referred to as the basipterygoid process (e.g. [100]), especially in neopterygians (e.g. [5,23,78]).

The basipterygoid process of the parasphenoid (processus ascendens anterior) is poorly developed in several taxa, including ^†^*Boreosomus* [47,103], and *Amia* and other halecomorphs [23,79], and it is absent in several actinopterygians, including the chondrosteans ^†^*Birgeria* [88], ^†^*Condorlepis* [104], ^†^*Chondrosteus* [105] and ^†^*Acipenser* [106], but also in ^†^*Australosomus* [88] and ^†^*Perleidus* [46,103]. The absence of this process is probably related to the poor development or complete absence of a processus basypterigius in the neurocranium in these taxa.

### 3.11. Quadratojugal

The homologies of the quadratojugal bone of actinopterygians, which is probably not homologous with the quadratojugal bone of sarcopterygians [107], have been debated by several authors. Among non-neopterygian actinopterygians, the quadratojugal is a small dermal bone attached to the palatoquadrate and it contains a pit-line (e.g. see detailed descriptions and illustrations of the quadratojugal in ^†^*Mimipiscis toombsi* and ^†^*Moythomasia durgaringa* in Gardiner [100]). In Ginglymodi, the bone identified as the quadratojugal is a splint-like dermal ossification lying along the dorsal margin of the preopercle, with an anterior head that buttresses the articular process of the quadrate and a posterior spine-like portion (see López-Arbarello [26]: fig. 3). The symplectic articulates between the quadrate and the posterior spine-like portion of the quadratojugal. The homology between the splint-like quadratojugal of ginglymodians and the plate-like quadratojugal of other actinopterygians has been supported by several authors (e.g. [51,108,109]).

In teleosts and in most halecomorphs, there is no independent quadratojugal, which is considered absent in halecomorphs, but fused to the quadrate in teleosts. Among these fishes, no ossification resembling the quadratojugal has been found in halecomorphs (except for those with a distinct plate-like quadratojugal), but the strengthened posteroventral margin of the teleost quadrate has been considered homologous to the quadratojugal (e.g. [26,51,52,75,78,109–116]).

Our characters 70 to 72 are coded based on the hypotheses of homology between the plate-like quadratojugal of non-neopterygian actinopterygians and a few basal neopterygians, the splint-like quadratojugal of ginglymodians and the posteroventral border of the teleost quadrate. Character 70 refers to the complete absence of a quadratojugal, which only occurs in halecomorphs and ^†^*Boreosomus piveteaui* among the taxa included in our analysis. The fusion of the quadratojugal with the quadrate is coded in our character 71, and character 72 distinguishes the plate-like from the splint-like quadratojugal. Therefore, characters 71 and 72 are inapplicable for most halecomorphs because they do not have a quadratojugal (70[0]), and character 72 is also inapplicable for teleosts because the shape of the quadratojugal cannot be established due to its complete fusion with the quadrate.

**Figure 5.**
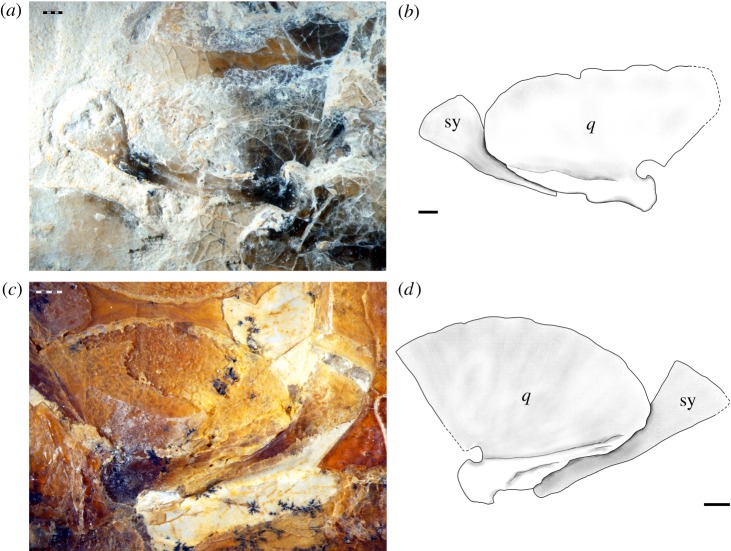
Symplectic-quadrate complex in ^†^*Aspidorhynchus acutirostris.* (*a*) Photograph and (*b*) line drawing of JME 1997.III.6. (*c*) Photograph and (*d*) line drawing of SNSB-BSPG 1964.XXIII.542. Abbreviations: q, quadrate; sy, symplectic. Scale bars, 1 mm.

After a thorough revision of the osteology of ^†^pachycormiforms and ^†^aspidorhynchiforms, Gouiric-Cavalli [117] concluded that in these fishes the quadratojugal is absent and the symplectic participates in a double-jaw articulation resembling the condition in *Amia calva*. We agree with this author in the presence of a double jaw articulation in the specimen SNSB-BSPG AS.VII.1069 of ^†^*Belonostomus speciosus* and the same condition has been reported in ^†^*Vinctifer comptoni* [71]. However, the symplectic does not reach the jaw in JME 1997.III.6 (figure 5*a*,*b*) or other examined specimens of ^†^*Aspidorhynchus acutirostris* (e.g. SNSB-BSPG 1964.XXIII.542; figure 5*c*,*d*), in which these bones are well preserved and exposed. Both in ^†^*Belonostomus speciosus* (SNSB-BSPG AS.VII.1069) and in ^†^*Aspidorhynchus acutirostris* (JME 1997.III.6, SNSB-BSPG 1964.XXIII.542), the bone tissue at the posteroventral border of the quadrate is different and partially separated from the rest of the quadrate, and it is here considered as a quadratojugal (figure 5).

### 3.12. Infraorbital bones

**Figure 6.**
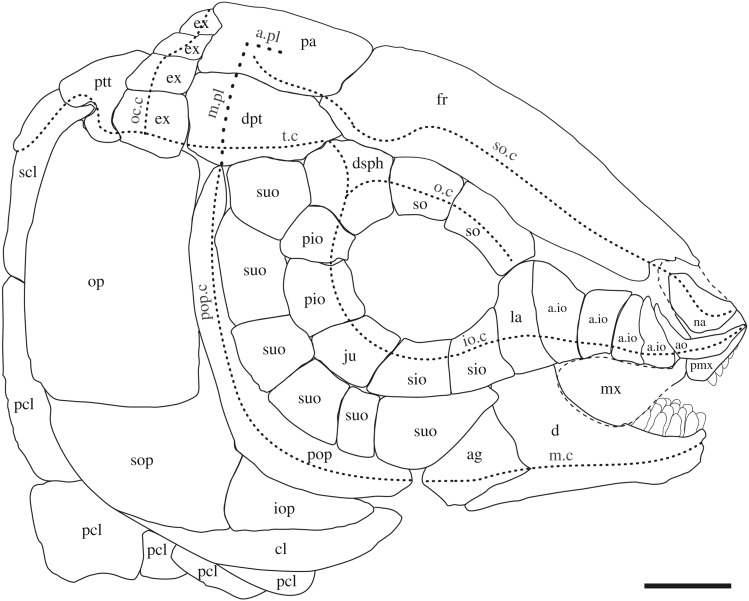
Reconstruction of the skull in ^†^*Scheenstia zappi*, modified from [121]. Abbreviations: a.io, anterior infraorbital bone; ag, angular bone; ao, antorbital bone; a.pl, anterior pit line; cl, cleithrum; d, dentary; dpt, dermopterotic bone; dsph, dermosphenotic bone; ex, extrascapular bone; fr, frontal bone; io, infraorbital bone; io.c, infraorbital sensory canal; iop, interoperculum; ju, jugal bone; la, lachrymal; m.c, mandibular sensory canal; m.pl, middle pit line; mx, maxilla; na, nasal bone; o.c, orbital sensory canal; oc.c, occipital sensory canal; op, operculum; pa, parietal bone; pcl, postcleithrum; pio, posterior infraorbital bone; pmx, premaxilla; pop, preoperculum; pop.c, preopercular sensory canal; ptt, post-temporal bone; scl, supracleithrum; sio, subinfraorbital bone; so.c, supraorbital sensory canal; so, supraorbital bone; sop, suboperculum; suo, suborbital bone; t.c, temporal sensory canal. Scale bar, 1 cm.

López-Arbarello ([26]: p. 11) discusses the homologies between the bones carrying the infraorbital sensory canal. Without exception, these bones develop in connection with one or more neuromasts [108,113,118–120], but the association of each of these bones with particular neuromasts of the infraorbital sensory line is not possible because the number of neuromasts in this sensory line is highly variable [113], even between the left and right sides of the same specimen of an individual [108]. Nonetheless, the rostral, antorbital and dermosphenotic have a different developmental timing with respect to the rest of the series and can be distinguished because of their association with particular segments of the cephalic sensory canals ([26]; figure 6).

The rostral bone is associated with the ethmoidal commissure, and the presence of the ethmoidal commissure in potentially compound bones (states 0, 2 and 3) indicates fusion of the rostral with other ossifications ([122,123]; e.g. ^†^*Leptolepis coryphaenoides*, figure 4*d*). The complete absence of any trace of the ethmoidal commissure or a median ossification separate from the compound bones indicates that the neuromasts involved in the ethmoidal commissure or in the ethmoidal pit line do not induce any ossification and, thus, there is a real absence of a rostral bone.

The antorbital bone is associated with the junction between the infraorbital and supraorbital canals and it is adjacent to the rostral in holosteans and basal teleosts. In more derived teleosts, however, there are no separate bones anterior to the lachrymal or there is only a small bone in the position of the antorbital, but it has no sensory canals or pit-lines. This latter bone is considered homologous to the antorbital bone [62].

The dermosphenotic carries the last portion of the infraorbital sensory canal and it is placed at the posterodorsal corner of the orbit. In most neopterygians, the dermosphenotic is loosely attached to the frontal and dermopterotic, lying on the sphenotic, but it is tightly sutured and incorporated into the skull roof in several halecomorphs. The dermosphenotic might be fused to or separate and distant from the sphenotic in some taxa (see above).

The series of infraorbital bones between the antorbital and the dermosphenotic should be treated as a whole [26]. Among them, four regions can be distinguished for the sake of comparison: the anteroventral, ventral, posteroventral and posterior margins of the orbit. The number and/or shape of the infraorbital bone or bones involved in each of these regions have long been used in actinopterygian systematics and they have received different names according to their position (e.g. [5,23]). To facilitate comparisons, we adopt this terminology, but without direct implication of taxic primary homology for the individual bones. Accordingly, the terms lachrymal, subinfraorbitals, jugal and postinfraorbitals are used in reference to the bones involved in each of the regions previously indicated (figure 6).

### 3.13. Vertebral centra

The composition of the vertebral centrum is still very poorly understood in fossil neopterygians, except for some teleost taxa [29,63,124–126]. Three main tissues, product of different mineralization processes, might be involved in the ossification of the vertebral centrum: arcocentrum, chordacentrum and autocentrum. Other authors (e.g. op. cit.) have attempted to define different vertebral types according to the different combination of tissues involved in the ossified centrum. Here, we proposed a completely different approach based on the general hypothesis that each of these three tissues develops independently from each other, i.e. the presence of one tissue does not imply the presence or absence of any of the other tissues. Consequently, we propose three independent characters (chs. 225–227) scoring the presence/absence of each of these tissues.

The vertebrae in our out-group taxa ^†^*Pteronisculus* and ^†^*Boreosomus*, but also in the basal halecomorph ^†^*Watsonulus* and, as far as know, in most ginglymodians outside Lepisosteoidea (^†^*Semionotus elegans*, ^†^*Paralepidotus ornatus*, ^†^*Isanichthys lertboosi*, ^†^*Thaiichthys buddhabutrensis*, ^†^*Araripelepidotes temnurus*) are only formed by the dorsal and ventral arcual elements, and the centrum does not ossify. Therefore, none of these tissues is present in the vertebral centra of these taxa.

The arcocentrum is an endochondral ossification derived from the dorsal and ventral arcualia. The crescent-shaped hemicentra of the vertebrae of the halecomorph ^†^*Caturus* has been interpreted as hemichordacentra [23,127,128]. However, after close examination of specimens, we agree with Laerm ([125]: p. 195) that the hemicentra of caturids (clearly visible in the specimen NHMUK PV 20578 of ^†^*Caturus furcatus*) are endochondral ossifications (225[1]). Similarly, the ring-like centra of ^†^*Ophiopsiella* (previously ^†^*Ophiopsis*, see [129]) have been thought to be formed by a cylinder of chordacentrum surrounded by autocentrum [95]. Laerm [125] questioned this hypothesis because the inner ring of the vertebral centra of ^†^*Ophiopsiella* is ossified instead of being mineralized as is the case of the chordacentra in other actinopterygians. Gardiner *et al*. [83] consider the drum-like centra of ^†^*Ophiopsiella* to be homologous with the centra in *Amia*. We, therefore, follow the latter authors and scored state (225[2]) for this taxon.

The vertebral centra of *Amia*, state (225[2]), is formed by endochondral replacement of cartilaginous arch base and intercalated anlagen [125,130,131]. In the abdominal region, the centra are monospondylous and result in perichordal ossification of the interdorsal and basiventral arcualia. The diplospondylous centra of the caudal region are formed by the fusion of the interdorsal and interventral, and basidorsal and basiventral arcocentra.

Among gars, the development of the holospondylous vertebrae has been studied in *Lepisosteus* [90,125,132–134], in which the centrum is mainly formed by endochondral replacement of a continuous perichordal tube of cartilage derived from the dorsal and ventral arch anlagen [125,134]. This condition corresponds to our character state (225[2]). The solidly ossified opisthocoelous centra of other fossil and living gars resemble the centra of *Lepisosteus* and there is no reason to suspect a different composition. The hemicentra of ^†^*Scheenstia mantelli* are expanded arcocentra, according to Patterson ([51]: p. 294). Interestingly, the vertebral centra of ^†^*Macrosemius rostratus* are perichordally ossified and continuous with the parapophysis, but not with the neural arches (SNSB-BSPG AS I 770, [95]: p. 157), thus resembling ontogenetic stages of *Amia*.

The vertebral centra of aspidorhynchids and some Jurassic teleosts like ^†^*Siemensichthys*, are formed by annular chordacentra surrounded by the dorsal and ventral arcocentra [128,135].

The chordacentrum is the result of mineralization within the fibrous sheath of the notochord and it is the only component of the vertebral centra in several basal teleosts (226[1]). The chordacentrum in basal teleosts (e.g. ^†^*Annaichthys*, ^†^*Pholidophoretes*; [29]) might form complete rings (^†^*Ichthyokentema*, ^†^*Catervariolus*; [136,137]) or might be represented by dorsal and/or ventral hemichordacentra (^†^*Eurycormus*, ^†^*Parapholidophorus*, ^†^*Pholidoctenus*, ^†^*Pholidorhynchodon*; [29,138]). The hemichordacentra, however, usually fused to form complete rings (^†^*Pholidophorus*; [29]), so we consider this variation to be ontogenetic. Apart from these basal teleosts, the only other taxon with vertebral centra formed by mineralized chordacentrum in our data matrix is ^†^*Australosomus kochi* [46,88].

The centra of living teleosts are mainly formed by direct membranous ossification of the sclerotomal perichordal tube (autocentrum), although chordacentral and arcocentral remnants are variably present [125,128,139,140].

### 3.14. Epurals

The term epurals refers to the series of median rod-like bones placed posterior to the last fully developed neural spine in the caudal skeleton of actinopterygians. Epurals have been interpreted as homologous to detached neural arches [141–144], homologous with radials [145–147], or homologous with the supraneurals [148,149]. Comparing with the series of epurals in the Triassic ^†^*Pteronisculus*, ^†^*Australosomus* and other actinopterygians, and considering the one-to-one relationship between these bones and the neural arches in these fishes, Patterson ([150]: pp. 220–221) concluded that the epurals are detached neural spines, which he considered homologous to the anterior series of supraneurals. However, the homology between supraneurals and neural spines has been questioned[151,152].

Schultze & Arratia [135,140] and Arratia & Schultze [152] restricted the term epurals to the median bones placed dorsal to the neural arches of preural and ural centra in teleosts, interpreting them as detached neural spines, which they did not consider homologous to supraneurals. These authors further distinguished the ‘epurals’ in *Amia* and other neopterygians, rejecting the homology between these elements and the epurals of teleosts.

In *Amia* and other halecomorphs (e.g. ^†^*Ionoscopus cyprinoides*; see examples in Grande & Bemis [23]), the most anterior ‘epurals’ intercalate with the complete neural spines of the last preural vertebrae, but the more posterior epurals (only one in *Amia*, two in ^†^*Ionoscopus cyprinoides*) are placed directly dorsal to short ural neural spines and/or ural neural arches, as is the case of the epurals in teleosts. Both ‘epurals’ and epurals are also present in non-neopterygian actinopterygians like Polyodontidae [153] and Polypteridae [5] and the question of homology between these elements and between them and the supraneurals remains open.

For the present study, we follow Schultze & Arratia [135,140] and code two independent a/p characters for the epurals (ch. 265) corresponding to the median bones posterior to the completely developed neural spine and interpreted as detached neural spines, and ‘epurals’ (ch. 267) corresponding to the median bones intercalating between the complete neural spines, which are attached to their corresponding neural arches. Accordingly, the median elements in the caudal skeleton of ^†^*Australosomus* and ^†^*Pteronisculus* are epurals [47,88]. Among the taxa included in our analysis, ‘epurals’ are only present in halecomorphs and pachycormiforms.

The ‘epurals’ of pachycormiforms need a special discussion. These elements are placed dorsal to a series of median bones named ‘uroneural-like elements’ by Lambers [154], which are interpreted as modified neural arches and spines of several preural and ural vertebrae [155]. Accordingly, because the neural spine is involved in the formation of the ‘uroneural-like elements’, the dorsal series is interpreted as ‘epurals’ in pachycormiforms [155].

### 3.15. Uroneurals, ‘posterior uroneurals’ and urodermals

The name uroneural was given by Regan ([145]: p. 355) to the modified ural neural arches in *Elops* and *Megalops*, which are elongated paired bones spreading along the dorso-lateral surface of the last preural and ural centra. The term was later brought to some confusion with the term urodermal (see below), but Patterson ([150]: pp. 221–231) clarified the use of both terms, fixing the name uroneurals to the series of paired elongated bones flanking the dorsolateral surfaces of preural and/or ural vertebral centra, which represent modified ural neural arches.

A series of uroneural bones is present in ^†^*Eurycormus.* Arratia & Schultze ([138]: p. 32; also in [135]: table 3) distinguished the first element in this series as an uroneural-like bone, assuming that it represents the modified neural arch of the last preural centrum. Although these authors did not explain the reasons for this interpretation, it might be related to the absence of a neural arch on the last preural centrum in this fish. However, although possible, this absence does not necessarily imply that the first element in the series of uroneurals truly corresponds to the first preural centrum, and we thus do not make this distinction.

**Figure 7.**
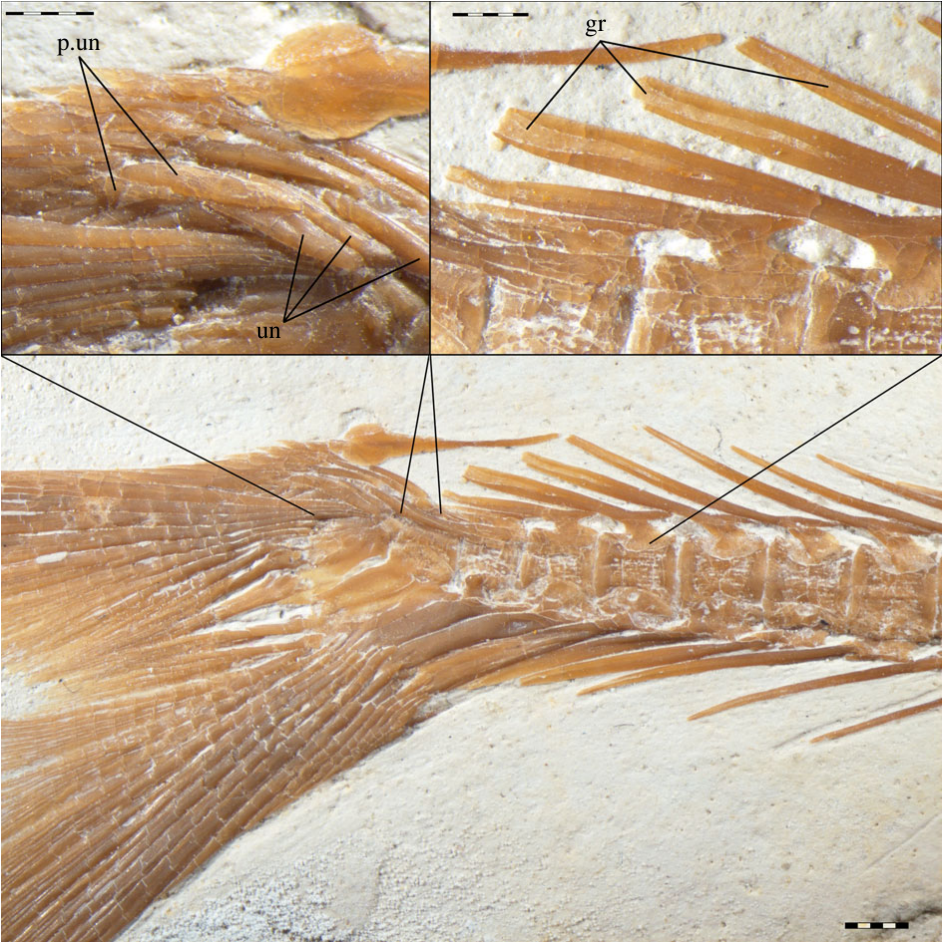
Caudal skeleton in ^†^*Tharsis dubius* (SNSB-BSPG 1964.VIII.280). Detailed photograph showing the uroneurals (un) and ‘posterior uroneurals’ (p.un). Detailed photograph showing the broad neural spines, with a median groove (gr), on the upper right corner.

The uroneurals in ^†^*Tharsis dubius* are described as forming two series (figure 7). The bones forming the anterior series are clearly uroneurals as described in character 273. The second series is formed by three bone splints, which have a different orientation and are placed more laterally than the anterior uroneurals. All or some of these bones are overlapping the bases of the most dorsal principal caudal fin rays in some specimens and, thus, their homology with uroneurals or urodermals (see discussion of character 294) is unclear. Pending a more detailed study to clarify the homology of these bones, we here code an independent character to analyse the presence of such ‘posterior uroneurals’ among the taxa studied.

The homology between the series of reduced rhomboid scales flanking the bases of the uppermost principal caudal fin rays in Jurassic teleosts and the rhomboid scales in the body lobe of the heterocercal tail of some non-neopterygian actinopterygians was first proposed by Nybelin [142]. Nybelin, however, did not distinguish between these elements and the series of modified ural neural arches (i.e. uroneurals), which he also interpreted as derived from rhomboid scales that became (phylogenetically) absorbed and associated to the axial skeleton. Nybelin thus proposed the name urodermalia for the two series. Patterson [150] restricted the term urodermals to the posterior series, which represent modified rhomboid scales, and, following Regan [145], reaffirmed the term uroneurals for the anterior series, which represent modified ural neural arches.

Patterson [150] further discussed the homology between the urodermals and the patches of rhomboid scales in the reduced body lobe of Early Jurassic teleosts and accepted the homology between them and the scales in the body lobe of the heterocercal tail as proposed by Nybelin [142]. Following this hypothesis, we accept the homology between the few modified scales, with or without ganoine layer, which are flanking the base of the most dorsal principal caudal fin rays in several teleosts and halecomorphs, the bodies of which are naked or covered with elasmoid scales, and the scales in the complete body lobe of the heterocercal and abbreviated heterocercal tails of other actinopterygians.

### 3.16. Serrated appendages and clavicles

The term ‘serrated appendages’ was coined by Wilder [156] for the thin, toothed ossifications placed on each side of the isthmus, anterolateral to the cleithrum in *Amia calva*. These appendages develop independently and ‘have no connection with either cranial or postcranial bone or muscles’ ([157]: p. 522). Only the posterior serrated appendage abuts against the cleithrum, which has a similar microscopic composition of cellular bone bearing oblique denticle-bearing ridges ([157]: p. 528). At least, the posterior serrated appendage has been considered homologous with the clavicle of other actinopterygians [76,157–159].

Erroneously, the term ‘serrated appendage’ has been used by Arratia ([29]: p. 125) for the ornamented lateral surface of the cleithrum in several Triassic and other teleosts, although she clearly distinguished this structure from the condition in amiiforms. We agree with Arratia on the equivalence of the condition in the Triassic teleosts and toothed ridges on the cleithrum of ^†^*Watsonulus* and ^†^*Atacamichthys*, but there is no evidence for these structures developing as separate appendages. On the contrary, and in agreement with the observations of Liem & Woods [157], these toothed ridges are part of the cleithrum (our character 325).

## 4. Phylogenetic analyses

**Figure 8.**
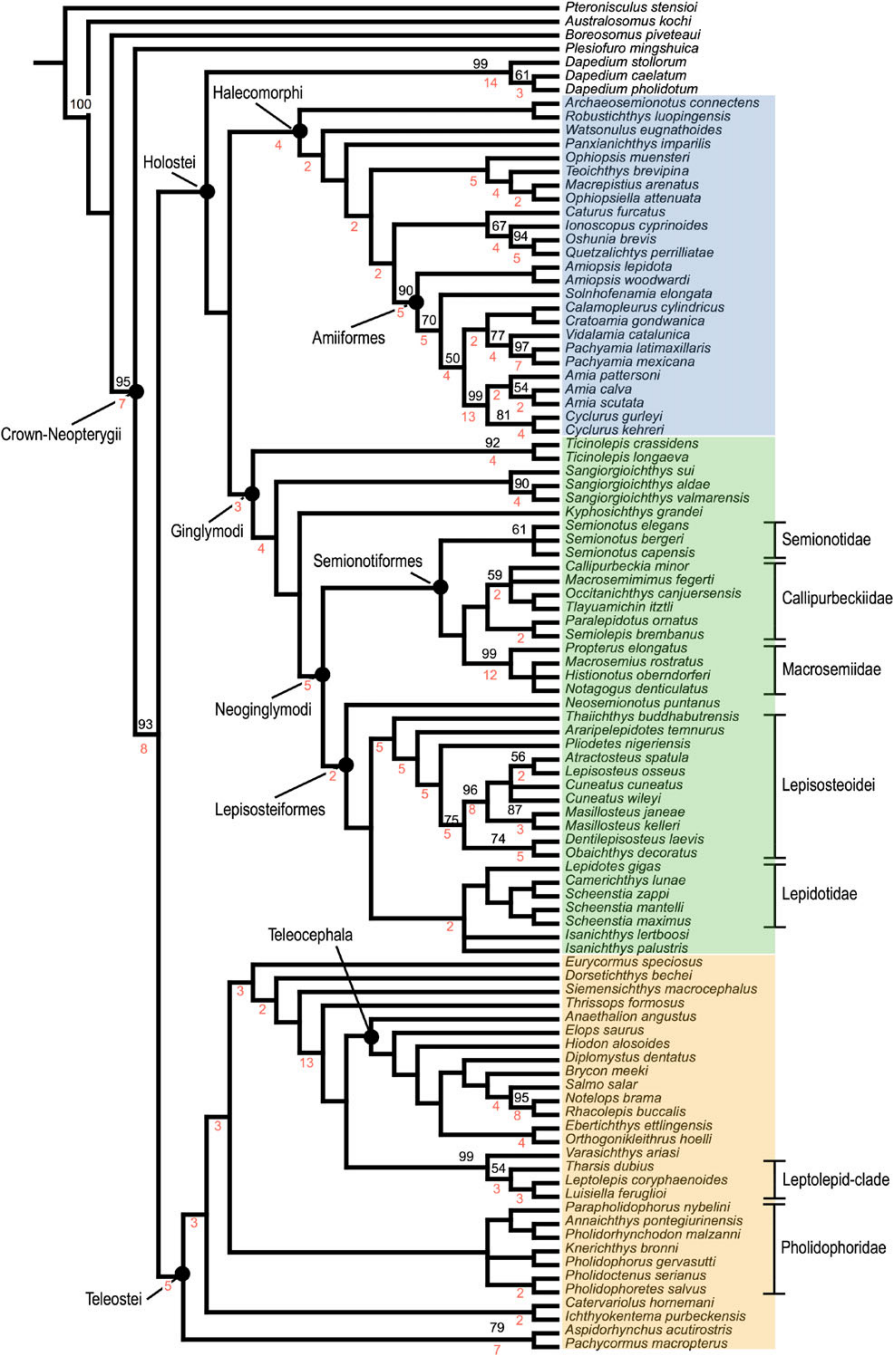
Strict consensus tree of 24 most parsimonious trees, equal weights analysis with constraints. Tree length = 2175 steps, consistency index = 0.268 and retention index = 0.678. Bremer indexes and bootstrap values larger than 50 are indicated with red and black numbers, respectively, at the corresponding nodes. Halecomorphi is highlighted in blue, Ginglymodi in green and Teleostei in orange.

**Figure 9.**
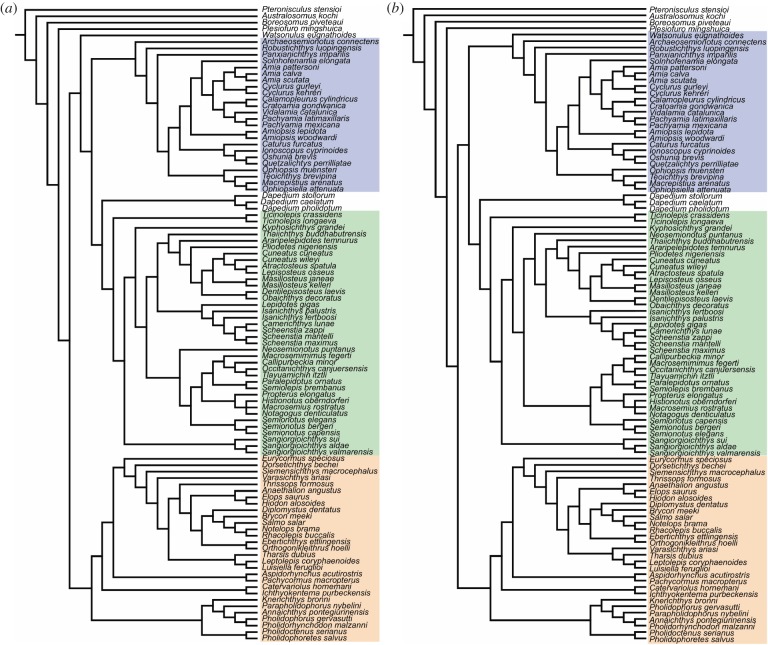
Single most parsimonious trees of the analyses with implied weights with constraints. (*a*) Strong *K*-value of 3; (*b*) moderate down-weighting *K*-value of 8. Halecomorphi is highlighted in blue, Ginglymodi in green and Teleostei in orange. Bremer and bootstrap values for these trees are available in electronic supplementary material, file S3.

The equal weighted cladistic analysis resulted in 24 most parsimonious trees (MPTs) of 2175 steps, with a consistency index (CI) of 0.268 and a retention index (RI) of 0.678. The strict consensus of the 24 most parsimonious trees is represented in figure 8. The consensus shows a generally well-resolved phylogenetic hypothesis with a monophyletic crown-Neopterygii including monophyletic Teleostei and Holostei. Bremer decay indexes and bootstrap supports for all nodes are indicated in figure 8. In this phylogenetic hypothesis, several taxa (e.g. ^†^*Thrissops formosus*, ^†^*Caturus furcatus*) took an unexpected position (see discussions below). We thus run implied weighting analyses to explore the effect of homoplasies in our hypothesis. These analyses were performed with a moderate down-weighting *K*-value of 8 and strong *K*-value of 3 and the corresponding strict consensus trees are represented in figure 9 (Bremer and bootstrap values for these cladograms are included in the electronic supplementary material, file S3). The following discussions are mainly based on the results of the equal weighted analysis (topology of and character optimization on the strict consensus of the 24 MPTs). Different relationships obtained in one or the other of the implied weighted analyses are discussed whenever appropriate, but our conclusions are only based on the equal weighted analysis because recent simulation analysis using equal and applied weighting came to the conclusion that implied weighting performs worse than equal weighting in most cases (e.g. [160]).

### 4.1. Systematic position of ^†^*Ticinolepis*

The monophyly of ^†^*Ticinolepis*, including two species, ^†^*T. longaeva* and ^†^*T. crassidens*, is confirmed with one unique and four homoplastic synapomorphies (electronic supplementary material, file S4) and the genus is recovered as the sister-taxon of all other ginglymodians (figure 8). This phylogenetic position is stable, although in the hypotheses given by implied weighting analyses ^†^*Dapedium* is included at the base of Ginglymodi as the sister-group of ^†^*Ticinolepis* plus all other ginglymodians. The monophyly of Ginglymodi is supported by two unique synapomorphies: the presence of a splint-like quadratojugal (ch. 72[1]) and anterior infraorbital bones (ch. 130[1]). The node is further supported by four homoplastic characters. The ectopterygoid is crescent shaped (ch. 62[1]) in Ginglymodi except lepisosteoids, but also in ^†^*Pachycormus macropterus* and in some teleosts (^†^*Ebertichthys*, ^†^*Tharsis dubius*, ^†^*Luisiella feruglioi*). The antorbital bone being excluded from the margin of the orbit (ch. 139[0]) is the condition shown by all studied ginglymodians, but also several halecomorphs and some teleosts. The subopercle is shallow (ch. 203[1]) in all ginglymodians except ^†^*Pliodetes* and more derived gars, but it is also shallow in most halecomorphs and teleosts. The median gular is absent in all ginglymodians except ^†^*Kyphosichthys* (ch. 208[0]).

López-Arbarello *et al*. [37] discussed several morphological features shared by ^†^*Ticinolepis* and the halecomorphs. Some of these features, such as the relatively large broad nasal bones (ch. 106[0]) or the presence of a well-developed medial wing of the cleithrum (ch. 324[1]), are plesiomorphic conditions also present in other neopterygians and in the out-group taxa, but the presence of a supramaxillary notch (ch. 162[3]) and a branchiopercle (ch. 206[1]) is uniquely derived within Holostei (see below). The series of very shallow scales covering the ventrum in ^†^*Ticinolepis* (ch. 10[1]) is, as indicated by López-Arbarello *et al*. [37] also present in some halecomorphs (^†^*Archaeosemionotus*, ^†^*Ophiopsis*, ^†^*Ophiopsiella*), but it also occurs in ^†^*Aspidorhynchus* [161] and the out-group taxon ^†^*Australosomus* [88].

The resemblances discussed by López-Arbarello *et al*. [37] between the braincase of ^†^*Ticinolepis* and several halecomorphs are mostly plesiomorphic (e.g. the presence of well-ossified lateral ethmoids; ch. 46[0]). Similarly, a large, vertically oriented ascending process of the parasphenoid, which articulates with the sphenotic (ch. 54[0]) is the condition present in all studied halecomorphs, but also in the basal teleost ^†^*Catervariolus* and the out-group taxa ^†^*Australosomos* and ^†^*Pteronisculus*. The facet for the articulation of the hyomandibula in ^†^*Ticinolepis* is formed between the sphenotic, prootic and pterotic bones (ch. 39[3]), which is also the case in the halecomorph ^†^*Caturus furcatus* (NHMUK 20578), but also in the basal teleosts ^†^*Catervariolus* [137], ^†^*Ichthyokentema* [136] and ^†^*Dorsetichthys* [78]. The participation of bones in this facet (ch. 39) is highly variable among the taxa studied, but the state in ^†^*Ticinopelis* is probably the plesiomorphic condition. The condition in the out-group taxa ^†^*Australosomos*, ^†^*Boreosomus* and ^†^*Pteronisculus*, and in ^†^*Watsonulus* is not comparable because there are no separate bones in the braincase of these fishes. Unfortunately, the braincase of other basal taxa like ^†^*Plesiofuro* and the most basal halecomorphs ^†^*Archaeosemionotus*, ^†^*Robustichthys* and ^†^*Panxianichthys* is unknown.

Resembling the amiids, the frontals of ^†^*Ticinolepis* cover the orbital and large part of the temporal regions of the skull, with more than one-third of the length extending behind the orbit (ch. 103[1]). According to our cladistic analysis, this pattern is acquired independently in these two groups and in the halecomorphs ^†^*Caturus furcatus* and ^†^*Watsonulus eugnathoides* and in the teleost ^†^*Rhacolepis buccalis* among the studied taxa. The same state is present in ^†^*Australosomus*, ^†^*Pteronisculus* and ^†^*Plesiofuro*, suggesting these are reversals to a plesiomorphic condition.

López-Arbarello *et al*. [37] noted the presence of a descending lamina in the dermopterotics (ch. 96[1]) of ^†^*Ticinolepis* and discussed the distribution of this feature in other actinopterygians. Although this trait is common among halecomorphs, the condition in most taxa is unknown and the known distribution among the taxa included in our analysis is rather patchy.

### 4.2. Holostei monophyly

Differing from all previous studies, our phylogenetic analysis includes numerous representatives of the main crown-neopterygian clades: Ginglymodians (36 taxa), Halecomorphi (25 taxa) and Teleostei (29 taxa). This more comprehensive dataset was necessary to explore the relationships of ^†^*Ticinolepis* due to its uncertain systematic assessment caused by the mixture of morphological features discussed by López-Arbarello *et al*. [37]. The resulting phylogenetic hypothesis (figure 8) shows a monophyletic Holostei including Halecomorphi, Ginglymodi and ^†^*Dapedium*, which is supported by two homoplastic synapomorphies: the presence of four or more suborbital bones (ch. 144[1]), which is also found in ^†^*Knerichthys browni*; and the presence of a presupracleithrum (ch. 320[1]), which is also present in ^†^*Pteronisculus* and might be a reversal because presupracleithra are known in several Palaeozoic and Triassic non-neopterygians (reversed also in ^†^*Pachycormus macrocephalus*).

The clade formed by Halecomorphi and Ginglymodi, which we could call the crown-Holostei, is well supported by seven synapomorphies in the equal weighted analysis. Among them, the presence of a tapering canal bearing anterior arm of antorbital bone (ch. 137[1]) is uniquely derived in Holostei, which was previously proposed as a synapomorphy of this clade by Hurley *et al*. ([4]: ch. 32[1]), who described it as the shape of the antorbital ‘tapering towards slender anterior process; tri-radiate canal within broader, posterior, portion’, and Grande ([5]: ch. 12[1]). Two other synapomorphies are also uniquely derived in Holostei: a supramaxillary notch (ch. 162[3]) and a branchiopercle (ch. 206[1]). The supramaxillary notch, which was first noted and described by Grande & Bemis [23], first occurs at the base of the crown-Holostei, it is lost in most ginglymodians (present in ^†^*Ticinolepis*, ^†^*Kyphosichthys*) and halecomorphs, but occurs again in and is a synapomorphy of Amiinae. Similarly, the branchiopercle is present in all the studied halecomorph taxa except ^†^*Quetzalichthys*, in the most basal ginglymodians ^†^*Ticinolepis* and ^†^*Sangiorgioichthys*, but it is lost in most ginglymodians and derived again in macrosemiids and the callipurbeckiids ^†^*Occitanichthys* and ^†^*Tlayuamichin*.

The remaining four synapomorphies supporting the crown-Holostei are also homoplastic and independently derived outside Holostei: a relatively long dermopterotic (ch. 93[1]), nasal bones that are not excavated by the posterior nostril (ch. 109[3]), two supraorbital bones (ch. 115[2]), and a maxilla with a straight ventral margin (ch. 159[0]). The shape of the pelvic bones in ^†^*Ticinolepis*, with flat proximal end and widened anteriorly (ch. 336[0]), which is different in the out-group taxa and all studied teleosts except ^†^*Eurycormus*, is another potential synapomorphy of Holostei.

Among previous morphological phylogenetic studies, Hurley *et al*. [4] proposed the monophyly of Holostei based on three synapomorphies. Among them, the shape of the antorbital was already discussed and it is here confirmed. The second synapomorphy relates to the shape of the rostral bone and would be equivalent to our state 0 of character 141 (relatively small approximately rectangular to tube-like), which is not given as a synapomorphy by the algorithm because the ancestral condition cannot be resolved in our data matrix. The third synapomorphy refers to the shape of the brain ‘optic tectum larger than telencephalon’ ([4]: ch. 66), which is not included in our dataset.

Grande [5] proposes 13 synapomorphies for Holostei, including the already discussed features of the antorbital and rostral bones (chs. 137 and 141). Most of the other features mentioned as synapomorphies of Holostei in Grande's analysis are found in more derived positions in our analysis and might have supported this clade in his analysis due to the more restricted sample of taxa. Among these other characters, the lack of a pterotic bone (ch. 37) derives independently in gars and the Triassic teleosts, and the presence of a small dermal component of the sphenotic (ch. 41[1]) derived independently in halecomorphs and some ginglymodians; the distribution of this feature is patchy within this latter clade. Similarly, the presence of large premaxillary nasal processes enclosing the olfactory foramen (ch. 152[1]) and the suture of the nasal processes of the premaxillae with the frontal bones (ch. 153[1,2]) are independently derived in amiiforms and ginglymodians above the level of ^†^*Ticinolepis*. The presence of paired vomers (ch. 59[0]), a compound coronoid process (ch. 169), and the presence of fringing fulcra on the caudal fin (ch. 298[0]) are plesiomorphic for Neopterygii. The presence of serrated appendages (ch. 328[1]) is difficult to evaluate in fossils, and in many cases it is impossible to be certain about their absence. As far as we have been able to evaluate, this feature has a rather patchy distribution, but only within Holostei. Serrated appendages are known in *Amia calva* and ^†^*Caturus furcatus* among halecomorphs, and in living gars, ^†^*Semionotus elegans* and ^†^*Propterus elongatus* among ginglymodians.

Other features proposed by Grande [5] are potential synapomorphies of Holostei, but more data are needed to understand the evolution of these characters because the ancestral condition cannot be resolved in our analysis. Among these, four hypobranchials (ch. 211[1]) are known only in ^†^*Pachycormus macrocephalus* outside Holostei, and the lateral dermethmoids forming the nasal processes of the premaxilla (ch. 48[0]) is apparently unique of holosteans, but the condition of the lateral ethmoids is so far unknown outside Holostei or Teleostei.

The phylogenetic relationships of ^†^*Dapedium* remain controversial: the genus is placed outside the crown-Holostei in the strict consensus tree of the equal weighted analysis (figure 8), but it is the most basal Ginglymodi in the implied weighted analyses (figure 9). These latter results are in agreement with the hypothesis proposed by Gibson [162] in the most recent and comprehensive cladistic analysis of dapediiforms, including four species of ^†^*Dapedium* and seven other dapediiform genera. Gibson's analysis is largely based on the data matrix of López-Arbarello [26], with the addition of several taxa, including three teleosts and three halecomorphs, but little additions to the set of characters (originally meant for ginglymodians only). Consequently, solving the relationships of dapediiforms requires a cladistic analysis including the complete set of dapediiform taxa, as done by Gibson, and a complete set of neopterygian taxa and characters as the one provided in this work.

### 4.3. Relationships among crown-neopterygians

Within Holostei, the patterns of relationships of ginglymodians and halecomorphs mostly agree with previous hypotheses. Above the series of Triassic stem-taxa, the Ginglymodi split in the orders Lepisosteiformes and ^†^Semionotiformes, as proposed by López-Arbarello [26]. Differing from this and the more recent study by López-Arbarello & Wencker [36], and in agreement with Sun & Ni [42], the Middle Triassic ^†^*Kyphosichthys* and ^†^*Sangiorgioichthys* are not semionotiforms but join the tree at the stem to the node (Lepisosteiformes, ^†^Semionotiformes). In our analysis, ^†^*Ticinolepis* also joins the tree at this stem as the most basal Ginglymodi and, thus, we now find it useful to distinguish the clade (Lepisosteiformes, ^†^Semionotiformes) as the Neoginglymodi, defined as the clade including *Lepisosteus* and ^†^*Semionotus*, and all descendants of their most recent common ancestor. Pending clarification of the relationships of ^†^*Dapedium*, the Ginglymodi remain defined as proposed by López-Arbarello ([26]: p. 34): the clade including all taxa more closely related to *Lepisosteus* than to ^†^*Dapedium*, *Amia* or ^†^*Pholidophorus.* This definition might need to be changed if future analyses show that dapediids are nested among more derived ginglymodians.

In the hypothesis of Sun & Ni [42], the stem-Neoginglymodi ^†^*Kyphosichthys* and ^†^*Sangiorgioichthys* form a clade they called Kyphosichthyidae, which is not retrieved in our analysis. Apart from the addition of ^†^*Ticinolepis* in our study, the taxonomic sample is different in the two cladistic analyses. In addition to ^†^*Kyphosichthys* and ^†^*Sangiorgioichthys*, Sun & Ni [42] include in their analysis ^†^*Luoxiongichthys hyperdorsalis* Wen *et al*. [163], a still poorly understood taxon also from the Anisian of South China, which is not included in our dataset. On the other hand, those authors include only two species of ^†^*Sangiorgioichthys*, ^†^*S. aldae* from the Ladinian of the Monte San Giorgio (Swiss and Italian Alps) and ^†^*S. sui* from the Anisian of South China, but we also include ^†^*S. valmarensis* also from the Ladinian of the Monte San Giorgio (Swiss Alps). A fourth species of this genus, ^†^*S. yanjuanensis*, also from the Anisian of South China, is incompletely known and not yet included in any cladistic analysis. Although these taxonomic differences could explain the different results, the putative synapomorphies supporting the Kyphosichthyidae are questionable according to the distribution of characters in our hypothesis. The putative synapomorphies of Kyphosichthyidae are the following: ‘triangular suborbital lateral to quadrate’ ([42]: p. 83) is uniquely derived in ^†^*Sangiorgioichthys* (ch. 150[1]) and absent in ^†^*Kyphosichthys*; ‘infraorbital bones forming the ventral border of the orbit subtriangular, broader ventrally, about 2 times deeper than long’ ([42]: p. 83) is the condition found in ^†^*Kyphosichthys* and many other ginglymodians (ch. 127[0]), but not in the species of ^†^*Sangiorgioichthys* (127[2,3]); ‘nasal process of the premaxilla not pierced by a large foramen for the olfactory nerve’ ([42]: p. 83) is a plesiomorphic holostean condition (ch. 152[0]). This latter feature is further unknown in ^†^*Kyphosichthys* and the species of ^†^*Sangiorgioichthys*, except for ^†^*S. sui*, where the nasal process of the premaxilla encloses a complete olfactory foramen (GMPKU-P-1707), a trait that is a synapomorphy of ginglymodians above the level of ^†^*Ticinolepis* (ch. 152[0]).

Sun & Ni [42] further argue that the genus ^†^*Sangiorgioichthys sensu* López-Arbarello *et al*. [164] is not monophyletic because the sister-taxon of ^†^*S. sui* is ^†^*Kyphosichthys grandei* and not ^†^*S. aldae*. In our analysis, however, the clade formed by the three species of ^†^*Sangiorgioichthys* is well supported by one uniquely derived character, the quadrate being laterally covered by one or two suborbitals forming a triangular plate (ch. 150[1]), and three homoplastic synapomorphies: a single anterior infraorbital bone (ch. 131[0]), otherwise only present in ^†^*Araripelepidotes temnurus*; the dorsal margin of the maxilla being gently concave and allocating supramaxilla (ch. 162[2]), a condition unique to this genus among the studied ginglymodians, but present in most teleosts and a few halecomorphs; and the coronoid process of the lower jaw being completely formed by the surangular (ch. 169[2]), which is a rather rare condition otherwise known in the gars and the Triassic teleost ^†^*Pholidoctenus serianus*. In our hypothesis, ^†^*Kyphosichthys grandei* is the sister-taxon to Neoginglymodi, and this relationship is supported by four synapomorphies: the presence of the longitudinal articulation of the scales of the body (ch. 8[1]), the above discussed shape of the infraorbital bones forming the ventral margin of the orbit (ch. 127[0]), the absence of a branchiopercle (ch. 206[0]), and the relatively low post-temporal bones, which are approximately as high as the dermopterotic (ch. 317[1]).

The monophyly of Neoginglymodi is supported by one uniquely derived synapomorphy: the presence of increasingly large and stout basal fulcra in the dorsal fin (see discussion of chs. 312 and 313 in electronic supplementary material, file S2) and six homoplastic synapomorphies (electronic supplementary material, file S4), and the relationships within this clade generally agree with López-Arbarello & Wencker [36], showing monophyletic Lepisosteidae and ^†^Lepidotidae within Lepisosteiformes, and ^†^Callipurbeckiidae, ^†^Macrosemiidae and ^†^Semionotidae within ^†^Semionotiformes. However, the relationship between some of these clades, or some taxa within these clades, is variable, indicating that more taxa and characters still need to be added to achieve a more robust phylogenetic hypothesis. For example, whereas the relationships of the genus ^†^*Isanichthys* from the Late Jurassic of Thailand remained unresolved in López-Arbarello [26], Cavin *et al*. [27] and Deesri *et al*. [31,32], the species ^†^*Isanichthys palustris* is the most basal lepisosteoid in López-Arbarello and Wencker [36], but the two species of this genus are included in ^†^Lepidotidae in the hypotheses presented here (figures 8 and 9), although the relationships of the taxa within ^†^Lepidotidae are variable in the implied weighting analyses. Similarly, according to Cavin *et al*. [27] and Deesri *et al*. [31,32], the Early Cretaceous ^†^*Thaiichthys buddhabutrensis*, also from Thailand, is the sister-taxon of a clade formed by the Early Cretaceous ^†^*Pliodetes* from Africa and ^†^*Araripelepidotes* from Brazil, which is the sister-group of the superfamily Lepisosteoidea *sensu* López-Arbarello [26]. However, this clade is not resolved in our analysis or in López-Arbarello & Wencker [36], and all those taxa are stem-Lepisosteoidea (figure 8). In this case, the relative position of these stem-taxa remains the same in both the equal and implied weighting analyses (figures 8 and 9). Very interestingly, the Early Cretaceous ^†^*Neosemionotus puntanus* from western Argentina is here retrieved as the most basal lepisosteiform (figure 8) as indicated in 89% of the most parsimonious trees in López-Arbarello [26]. On the contrary, the implied weighting analysis with the strongest constant value of 3 suggests that this taxon is the most basal ^†^semionotiform (figure 9*a*). In any case, as López-Arbarello [26] already stated, the basal position of ^†^*Neosemionotus*, with a ghost lineage going back to the Triassic, indicates that the history of ginglymodians in South America is much longer than currently known. Ginglymodians are well represented in the Late Jurassic–Early Cretaceous of Brazil and Argentina [165–167], but no reliable evidence of their presence has been found before that time [168]. This missing information certainly plays a role regarding the uncertainties about the phylogenetic relationships of ^†^*Neosemionotus*.

Among ^†^semionotiforms, although the clade ^†^Macrosemiidae is very well supported in all cladistic analyses, the relationships of this family vary from a position more basal than ^†^Semionotidae or ^†^Callipurbeckiidae in the earlier studies [26,27,31,32,72,87,96,169], via macrosemiids being the sister-group of ^†^Semionotidae in a clade that is the sister-group of ^†^Callipurbeckiidae in López-Arbarello & Wencker [36], to being the sister-group of ^†^Callipurbeckiidae in our current hypotheses (figures 8 and 9).

In Halecomorphi, the relationships within Amiidae, as well as the basal position of the Triassic taxa generally, agree with previous cladistic analyses. On the contrary, the order ^†^Ionoscopiformes is not monophyletic in our hypotheses, including the cladograms produced by the implied weighting analyses (figures 8 and 9). The name Ionoscopiformes was coined by Grande & Bemis [23] to name a clade including the families ^†^Ionoscopidae, ^†^Ophiopsidae and ^†^Oshunidae. Later authors did not recognize the monotypic family ^†^Oshunidae and placed ^†^*Oshunia brevis* within ^†^Ionoscopidae, together with ^†^*Ionoscopus cyprinoides* and ^†^*Quetzalichthys perilliatae* [24,41,170]. In the hypotheses proposed by these authors [33,171], this clade ^†^Ionoscopidae is the sister-group of ^†^Ophiopsidae, including the genera ^†^*Ophiopsis* (previously ^†^*Furo*) *muensteri*, ^†^*Ophiopsiella* (previously ^†^*Ophiopsis*), ^†^*Macrepistius* and ^†^*Teoichthys*, forming a monophyletic ^†^Ionoscopiformes. Xu *et al*. [33] described the Triassic ^†^*Robustichthys*, retrieved as the oldest ^†^ionoscopiform in a polytomy with ^†^Ionoscopidae and ^†^Ophiopsidae, and Xu & Shen [170] described the slightly younger ^†^*Panxianichthys*, retrieved as the sister-taxon to all other ^†^ionoscopiforms. After adding the Triassic ^†^*Allolepidotus*, ^†^*Asialepidotus* and ^†^*Eoeugnathus* to the analysis, Sun *et al*. [41] proposed very different relationships for ^†^*Panxianichthys* and proposed a clade ^†^Panxianichthyiformes including all these Triassic taxa (except ^†^*Robustichthys*, which is not included in their analysis). On the other hand, López-Arbarello *et al*. [34] redescribed the Triassic ^†^*Archaeosemionotus connectens* and proposed another different phylogenetic hypothesis. In this hypothesis, the ^†^Ionoscopiformes were monophyletic, but the family ^†^Ionoscopidae was not monophyletic and the sister-group of ^†^Ophiopsidae was the clade ^†^Furidae including ^†^*Ophiopsis* (previously ^†^*Furo*) *muensteri*, ^†^*Archaeosemionotus* and ^†^*Robustichthys*. Therefore, in the hypothesis of López-Arbarello *et al*. [34], the latter taxon was retrieved in a significantly more derived position compared to the other studies mentioned before. The disparities between all these studies indicate that the different sets of taxa and characters produce incompatible results for the relationships of the taxa classified in the order ^†^Ionoscopiformes, and the results obtained in our cladistic analysis might be understood as the result of merging those previous datasets. In our hypotheses, the families ^†^Ionoscopidae and ^†^Ophiopsidae are monophyletic, but they are not sister-groups, and the Triassic ^†^*Archaeosemionotus*, ^†^*Robustichthys* and ^†^*Panxianichthys* are retrieved at the base of the Halecomorphi, as well as the Early Triassic ^†^*Watsonulus* (figures 8 and 9). However, the other Triassic taxa ^†^*Allolepidotus*, ^†^*Asialepidotus* and ^†^*Eoeugnathus* are not included in our analysis and, thus, nothing can be said about the monophyly of ^†^Panxianichthyformes *sensu* Sun *et al*. [41], which will be explored in a future study.

Another unexpected result of our analysis is the phylogenetic position of ^†^*Caturus furcatus* outside the Amiiformes and within the ^†^ionoscopid clade (figures 8 and 9). This sister-group relationship between ^†^*Caturus* and ^†^ionoscopids is supported by six homoplastic synapomorphies, and not even in the implied weighted analyses is ^†^*Caturus* retrieved within the amiiform clade, as suggested by previous studies (e.g. [23,34,172]). Including other taxa like ^†^*Heterolepidotes*, ^†^*Osteorachis*, ^†^*Ainia* (=^†^*Callopterus*), which are potentially closely related to ^†^*Caturus* [23,51], in future, cladistic analyses will certainly solve the phylogenetic position of this taxon.

In agreement with the most recent phylogenetic hypothesis for basal teleosts [39], according to our results, the genera ^†^*Aspidorhynchus* and ^†^*Pachycormus* are sister-taxa and together represent the sister-group of all other teleosts (figure 8). Similarly, all Triassic teleosts form a monophyletic clade ^†^Pholidophoridae *sensu* Arratia [29]. The Jurassic ^†^*Eurycormus*, ^†^*Dorsetichthys* and ^†^*Siemensichthys* join the stem Teleocephala, but in different positions relative to each other compared to previous hypotheses [29,35], or ^†^*Eurycormus* is not on this stem, but is the sister-group of ^†^Pholidophoridae in Arratia [39]. The phylogenetic position of these basal teleost taxa is very different when implied weighting is applied (figure 9). In the cladograms obtained from both implied weighted analyses, ^†^Pholidophoridae is the sister-group of all other teleosts, including the clade formed by ^†^*Aspidorhynchus* and ^†^*Pachycormus*. Furthermore, and contrary to previous hypotheses [29,35], this latter clade is well nested within Teleostei, placed above the level of a clade formed by ^†^*Ichthyokentema* and ^†^*Catervariolus* in both implied weighted analyses.

**Figure 10.**
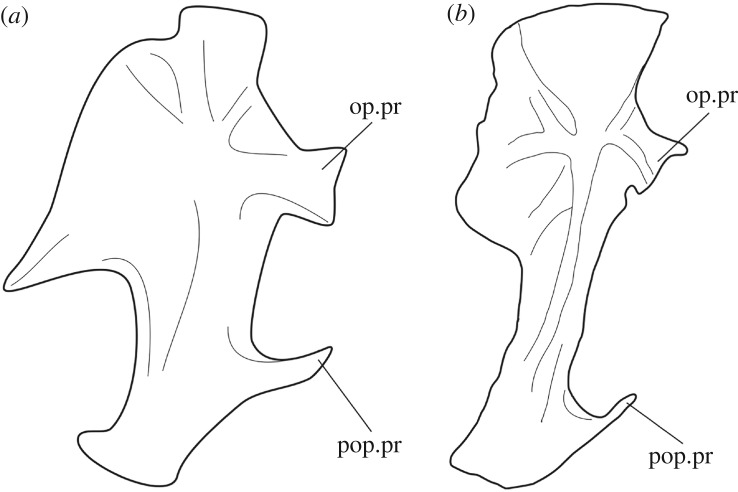
Preopercular process of the hyomandibula in (*a*), ^†^*Tharsis dubius*, reconstruction based on [174]: pl. 12, fig. 7; (*b*) ^†^*Luisiella feruglioi*, reconstruction based on [175]: fig. 7C. Abbreviations: op.pr, opercular process; pop.pro, preopercular process.

Recalling the family ^†^Leptolepidae *sensu* Nybelin [173], ^†^*Leptolepis coryphaenoides* and ^†^*Tharsis dubius* are more closely related to each other than to any other teleost in our analysis (figure 8). The clade including these taxa also includes ^†^*Luisiella feruglioi*, from the Late Jurassic of Argentina, and is supported with three uniquely and four homoplastic synapomorphies and it is stable also in the hypotheses given by the implied weighting analyses (figure 9). The characters uniquely derived in this Leptolepid-clade include the presence of a preopercular process of the hyomandibula (figure 10) and broad neural and haemal spines with a median groove (figure 7). The hyomandibula with a preopercular process was already recognized as a diagnostic of his Leptolepidae s. str. (^†^*Leptolepis*, ^†^*Proleptolepis* and ^†^*Tharsis*) by Nybelin [173], and at least some of these features are present in the other members of the family ^†^Luisiellidae (^†^*Cavenderichthys* [176] and ^†^*Waldmanichthys* [177]). Although several Jurassic teleost taxa are missing in our data matrix compared to previous studies, in particular the other members of the Gondwanan family ^†^Luisiellidae [35], the close phylogenetic relationships between these taxa might be the result of expanded character sampling and a new understanding of character change evolution on the teleost lineage due to the inclusion of numerous non-teleost fossil taxa in our analysis.

Contrary to the hypothesis of Arratia [65], and in agreement with that of Sferco *et al*. [35], the ^†^Varasichthyidae (here represented by ^†^*Varasichthys*) and ^†^Crossognathiformes *sensu* Taverne [178] (here represented by ^†^*Notelops* and ^†^*Rhacolepis*) are not closely related to each other in our analyses. This result supports the proposal of Taverne [178], also supported by Patterson [121] and Cavin [67], that the ^†^Crossognathiformes are teleocephalans. ^†^*Varasichthys* is here instead retrieved outside the crown-group, on the stem Teleocephala. An extended discussion of the relationships between ^†^Varasichthyidae and ^†^Crossognathiformes is provided in Sferco *et al*. [35], where the family ^†^Varasichthydae is represented by all its included taxa.

Further challenging previous ideas (e.g. [35,65]), ^†^*Varasichthys* is retrieved as the sister-taxon of the ^†^Leptolepid-clade in two of the three phylogenetic hypotheses obtained here (equal weighted analysis and implied weighted analysis with *K* = 8, figures 8 and 9*b*). ^†^*Varasichthys* shares four synapomorphies with the Leptolepid-clade: presence of a leptolepid notch in the anterodorsal ascending margin of the dentary (ch 167: 0 → 1); epipleural bones in abdominal and anterior caudal region (ch 242: 0 → 1); absence of hypaxial basal fulcra (ch 292: 0 → 1) and the first anal pterygiophore posterior to first haemal spine or infrahaemal (ch 308: 1 → 0). However, these are all homoplastic characters, which are widely distributed among teleosts, especially among the taxa on the stem-Teleocephala (see for example the distribution of the presence of a leptolepid notch in the anterodorsal ascending margin of the dentary in Sferco *et al*. [35]: fig. 4*a*). Furthermore, the grouping of ^†^*Varasichthys* with the ^†^Leptolepid-clade has a low bootstrap value (below 50). Therefore, although it is an interesting hypothesis, the sister-group relationship between ^†^*Varasichthys* and the ^†^Leptolepid-clade should be taken with caution, considering that the other three ^†^varasichthyid taxa, as well as the ^†^luisiellids ^†^*Cavenderichthys* and ^†^*Waldmanichthys* and other potential members of the ^†^Leptolepid-clade (e.g. several species of ^†^*Leptolepis* [173] are not included in our present analysis.

Among the extinct lineages of Mesozoic teleosts, the ^†^Ichthyodectiformes have been generally accepted as the sister-group of Teleocephala (e.g. [35,66]). ^†^Ichthyodectiformes are only represented by ^†^*Thrissops formosus* in our analysis, and this species is the sister-taxon of Teleocephala only in the strongest implied weighted analysis (*k* = 3; figure 9*a*). In the other two hypotheses, ^†^*Thrissops* is retrieved at a slightly more basal position, and the sister-group of Teleocephala is the clade formed by ^†^*Varasichthys* and the ^†^Leptolepid-clade (see above). Although only one step further from Teleocephala, this is a significant difference because of the broad taxonomic, stratigraphic and geographical range of this potentially alternative sister-group of Teleocephala, which encompasses two so far exclusively Gondwanan clades, the families ^†^Varasichthyidae and ^†^Luisiellidae.

### 4.4. Chronostratigraphic distribution

**Figure 11.**
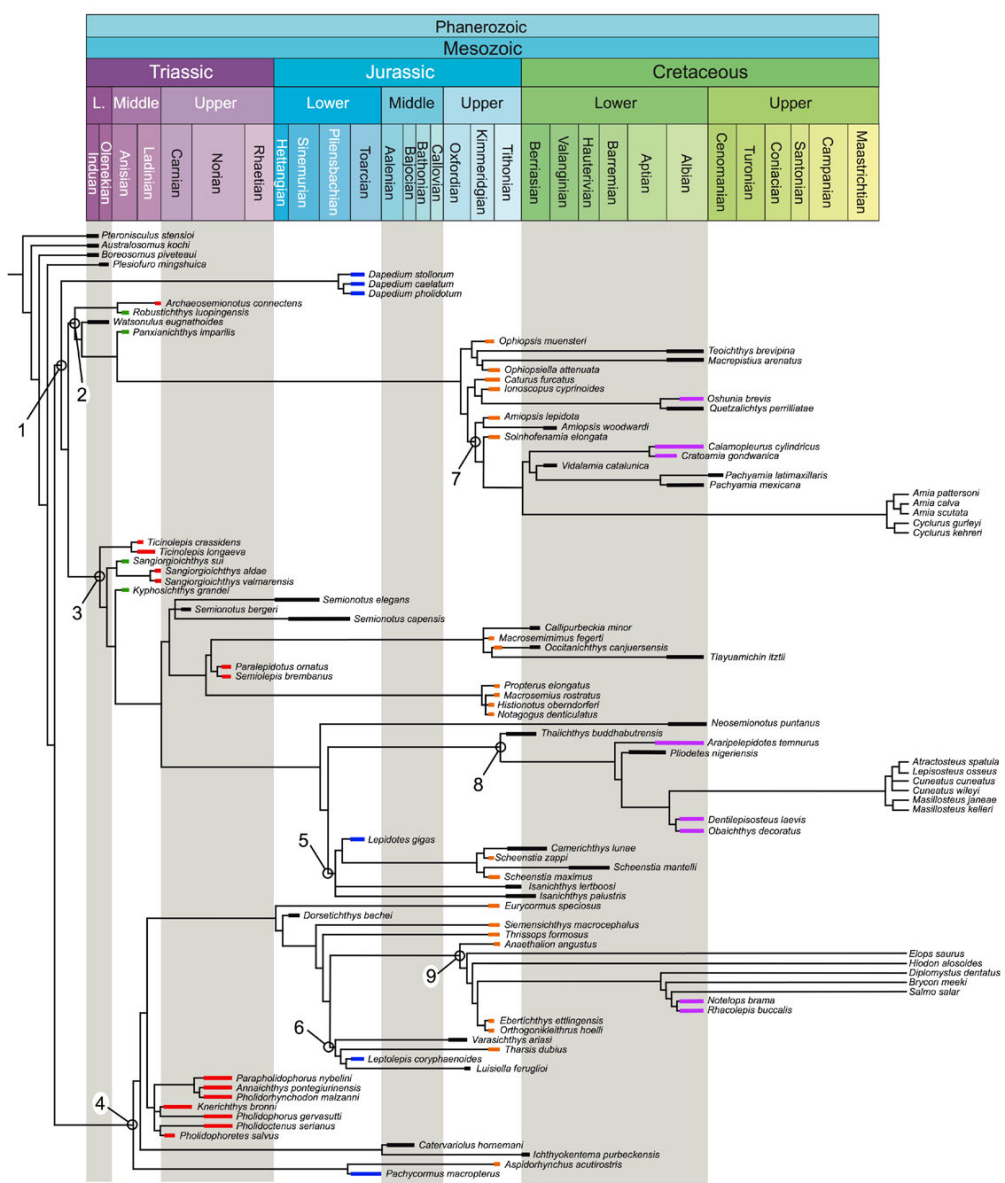
(*Caption overleaf*.)

The pattern of phylogenetic relationships and the chronostratigraphic distribution of the studied taxa indicate a rapid radiation of the holostean clades Halecomorphi (figure 11: Node 2) and Ginglymodi (figure 11: Node 3) during the approximately 15 Myr encompassed by the Early and Middle Triassic, immediately followed by a first rapid radiation of pholidophoriform teleosts (figure 11: Node 4) during the late Middle and Late Triassic. A second important radiation of ginglymodians and teleosts took place independently during the Early Jurassic. The lepidotids within Ginglymodi and the leptolepid-clade within Teleostei represent offshoots of their respective main stems during that period of time (figure 11: Nodes 5 and 6, respectively). In contrast, the crown-group Halecomorphi (figure 11: Node 7) present an enormous ghost lineage throughout half of the Mesozoic from the Middle Triassic to the early Late Jurassic. All three main clades Halecomorphi, Ginglymodi and Teleostei experienced a third important radiation during the Late Jurassic giving rise to their respective crown-groups (figure 11: Nodes 7–9).

**Figure 11.** (*Overleaf*.) Strict consensus tree of 24 most parsimonious trees resulted from the equal weights analysis with constraints, calibrated on a chronostratigraphic chart. Numbers indicate: 1, Holostei; 2, Halecomorphi; 3, Ginglymodi; 4, Teleostei; 5, Lepidotidae; 6, ^†^*Varasichthys* + Leptolepid-clade; 7, crown-Halecomorphi (Amiiformes); 8, crown-Ginglymodi (Lepisosteoidei); 9, crown-Teleostei (Teleocephala). Coloured bars highlight the taxa from the Middle Triassic of South China (green), Middle Triassic of the Alps (red), German Posidonienschiefer (blue), Late Jurassic of Southern Germany (orange) and the Aptian/Albian Araripe Basin of Brazil (purple). Chronostratigraphic provenance of the Mesozoic taxa: ^†^*Pteronisculus stensioi*, ^†^*Boreosomus piveteaui* and ^†^*Australosomus kochi*, Fish Zone 2–5 of Kap Stosch, Early Triassic (Induan) of East Greenland [46,84,179]; ^†^*Plesiofuro mingshuica*, Hongyanjing Formation of Gansu Province, China, Early Triassic (Olenekian) [69,180]; ^†^*Watsonulus eugnathoides*, middle Sakamena Formation of Madagascar, Early Triassic [85,181]; ^†^*Kiphosichthys grandei*, ^†^*Robustichthys luopingensis*, ^†^*Sangiorgioichthys sui*, Luoping biota, Member II of the Guanling Formation, Guizhou, China, Anisian (mid-Pelsonian), Middle Triassic [33,164,182]; ^†^*Panxianichthys imparilis*, Panxian biota, Member II of the Guanling Formation, Guizhou, China, Anisian (mid-Pelsonian), Middle Triassic [164,182]; ^†^*Ticinolepis crassidens*, uppermost Besano Formation, Monte San Giorgio, Switzerland, early Ladinian, Middle Triassic [37]; ^†^*Ticinolepis longaeva*, uppermost Besano Formation and most of the Meride Limestone, Monte San Giorgio, Switzerland, Ladinian, Middle Triassic [37]; ^†^*Sangiorgioichthys aldae* and ^†^*S*. *valmarensis*, Kalkschieferzone, uppermost Meride Limestone, Monte San Giorgio, Switzerland, latest Ladinian, Middle Triassic [37,183,184]; ^†^*Archaeosemionotus connectens*, Perledo Member of the Perledo-Varenna Formation, Italy, late Ladinian, Middle Triassic [34]; ^†^*Pholidophoretes salvus*, Reingraben beds, Austria, early Carnian (Julian), early Late Triassic [29]; ^†^*Knerichthys bronni*, Raild (=Cave del Predil), Friaul, Udine, Italy, Carnian, early Late Triassic [29]; ^†^*Semionotus bergeri*, Hassberge Formation (Coburger Sandstein), Coburg, Germany, late Carnian, early Late Triassic [185]; ^†^*Paralepidotus ornatus*, middle and late Norian localities of Italy and Austria, middle Late Triassic [186]; ^†^*Semiolepis brembanus*, vertebrate level between the Calcare di Zorzino and the Argillite di Riva di Solto at the boundary between middle and late Norian, Italy, middle Late Triassic [187]; ^†^*Annaichthys pontegiurinensis* and ^†^*Pholidophorus gervasuttii*, Ponte Giurino, ^†^*Parapholidophorus nybelini*, ^†^*Pholidoctenus serianus* and ^†^*Pholidorhynchodon malzannii*, Cene, Italy, Norian, middle Late Triassic [29]; ^†^*Semionotus elegans*, Towaco and Boonton formations (Hettangian and Sinemurian), Newark basin, New Jersey, Portland Formation (Sinemurian), Connecticut and Waterfall Formation (Hettangian), Virginia, USA, early Early Jurassic [82]; ^†^*Dorsetichthys bechei*, Lyme Regis, Dorset, England, early Sinemurian, early Early Jurassic [29,188]; ^†^*Semionotus capensis*, Clarens Formation, South Africa, Sinemurian–Pliensbachian, Early Jurassic [189]; ^†^*Dapedium caelatum*, ^†^*D*. *pholidotum* and ^†^*D*. *stollorum*, Early Toarcian localities of southern Germany, Luxembourg and northern France, Early Jurassic [190–192]; ^†^*Leptolepis coryphaenoides*, several early Toarcian localities in the UK, France and Germany, late Early Jurassic [33,193]; ^†^*Pachycormus macropterus*, several localities in the UK, France and Germany, Toarcian, late Early Jurassic [194]; ^†^*Catervariolus hornemani*, Stanleyville Formation, Democratic Republic of Congo, Aalenian–Bathonian, Middle Jurassic [133,195]; ^†^*Varasichthys ariasi*, Cordillera de Domeyko, Chile, Oxfordian, Late Jurassic [196]; ^†^*Luisiella feruglioi*, ‘estratos de Almada’, Cañadón Calcáreo Formation, Argentina, late Oxfordian, Late Jurassic [175,197]; ^†^*Ebertichthys ettlingensis*, ^†^*Histionotus oberndorferi*, ^†^*Macrosemimimus fegerti*, ^†^*Notagogus denticulatus*, ^†^*Ophiopsis muensteri*, ^†^*Orthogonikleithrus hoelli*, ^†^*Scheenstia zappi*, several late Kimmeridgian localities in southern Germany, Late Jurassic [38,121,193,198–200]; ^†^*Amiopsis lepidota*, ^†^*Caturus furcatus*, ^†^*Eurycormus speciosus*, ^†^*Ionoscopus cyprinoides*, ^†^*Ophiopsiella attenuata*, ^†^*Scheenstia maximus*, ^†^*Siemensichthys macrocephalus*, ^†^*Solnhofenamia elongata*, ^†^*Tharsis dubius*, ^†^*Thrissops formosus*, several late Kimmeridgian and early Tithonian localities in southern Germany, Late Jurassic [26,125,194]; ^†^*Anaethalion angustus*, ^†^*Aspidorhynchus acutirostris*, ^†^*Macrosemius rostratus*, ^†^*Propterus elongatus*, several early Tithonian localities in southern Germany, Late Jurassic [57,156,198,201]; ^†^*Occitanichthys canjuerensis*, Canjuers, France, Early Tithonian, Late Jurassic and Dorset, UK, Middle Purbeck Beds, middle Berriasian, Early Cretaceous [36,202]; ^†^*Thaiichthys buddhabutrensis*, ^†^*Isanichthys palustris*, ^†^*I*. *lertboosi*, Phu Kradung Formation, Thailand, Late Jurassic–Early Cretaceous [31,203]; ^†^*Ichthyokentema purbeckensis*, Dorset, UK, Lower Purbeck Beds, early Berriasian, Early Cretaceous [90,202]; ^†^*Callipurbeckia minor*, Dorset, UK, Middle Purbeck Beds, middle Berriasian, Early Cretaceous [202,204]; ^†^*Amiopsis woodwardi*, ^†^Vidalamia catalunica, Montsec Formation, Spain, Berriasian-Valanginian, Early Cretaceous [23]; ^†^*Pliodetes nigeriensis*, Elrhaz Formation, Niger Republic, Aptian, Early Cretaceous [205], ^†^*Pachyamia mexicana*, ^†^*Quetzalichthys perillatae*, ^†^*Teoichthys brevipina*, ^†^*Tlayuamichin itztli*, Tlayúa Formation, Mexico, Albian, Early Cretaceous [23,24,30,161]; ^†^*Neosemionotus puntanus*, Lower Member of the Lagarcito Formation, Argentina, Albian, Early Cretaceous [161]; ^†^*Araripelepidotes temnurus*, ^†^*Calamopleurus cylindricus*, Crato and Santana Formations, Brazil, late Aptian–Albian, Early Cretaceous [206]; ^†^*Cratoamia gondwanica*, Crato Formation, Brazil, late Aptian–early Albian, Early Cretaceous [206]; ^†^*Dentilepisosteus laevis*, ^†^*Notelops brama*, ^†^*Obaichthys decoratus*, ^†^*Oshunia brevis*, ^†^*Rhacolepis buccalis*, Santana Formation, Albian, Early Cretaceous [206]; ^†^*Macrepistius arenatus*, Glen Rose Formation, Texas, USA, Albian, Early Cretaceous [207]; ^†^*Pachyamia latimaxillaris*, Bet-Meir Formation, Ein-Yabrud near Ramallah, Middle East, early Cenomanian, early Late Cretaceous [23].

**Figure 12.**
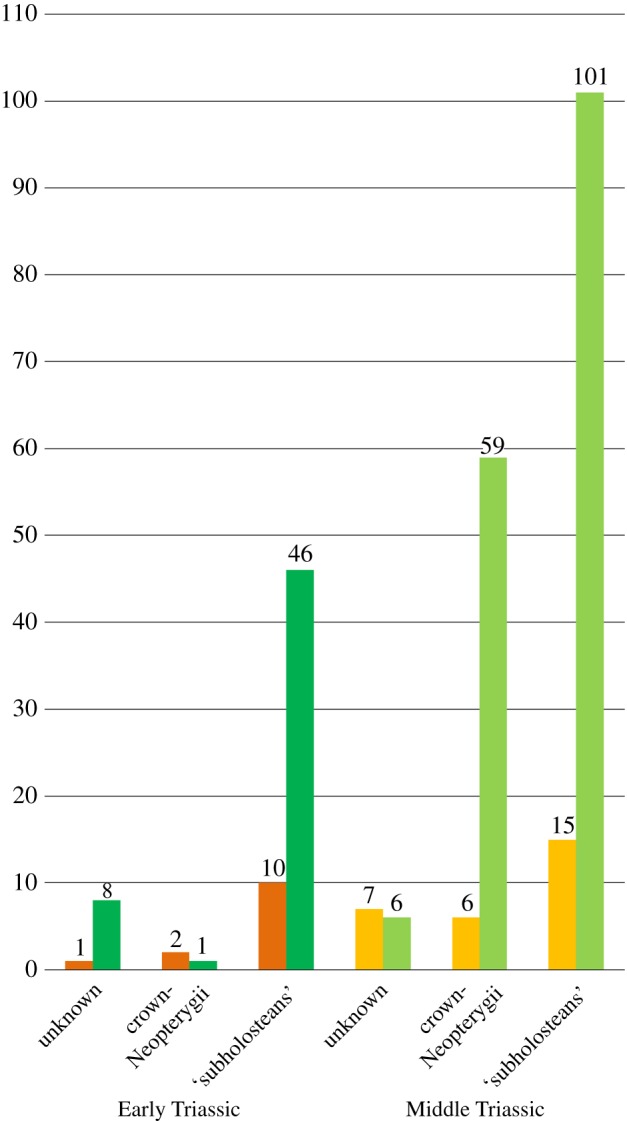
Graphic representation of the number of nominal species from Early and Middle Triassic freshwater (orange) and brackish and marine (green) sediments. Data from Romano *et al*. ([174]: table S3) with the addition of the following recently described taxa: ^†^*Ticinolepis longaeva* and ^†^*T. crassidens* from the Ladinian of the Monte San Giorgio [37]; ^†^*Frodoichthys luopingensis* and ^†^*Gimlichthys dawaziensis* from the Anisian of Yunnan Province [41]; ^†^*Mailingichthys nimaiguensis* from the Ladinian of Guizhou Province [208]; ^†^*Panxianichthys imparilis* from the Anisian of Paxian biota [164]; ^†^*Robustichthys luopingensis* from the Anisian of Luoping Biota [33]; ^†^*Habroichthys dolomiticus* from the Anisian of Monte Prà della Vacca [209]; ^†^*Altisolepis sinensis* from the middle–late Anisian of Luoping [210]; ^†^*Calaichthys tehul* from the Anisian of Cuyo Basin [211]; ^†^*Venusichthys comptus* from the Pelsonian–Anisian of Luoping [212]; ^†^*Wushaichthys exquisitus* [69]; ^†^*Peltopleurus nitidus* from the Anisian of Luoping biota [177]; ^†^*Plesiofuro mingshuica* from the Olenekian of Gansu Province [69].

The Early–Middle Triassic radiation of neopterygians has been thoroughly explored and documented by Romano *et al*. [174] and it is part of what they called the Triassic actinopterygian revolution. This first important radiation of the crown-Neopterygii was apparently facilitated by the demise of several chondrichthyan clades and driven by the successful acquisition of several feeding, locomotory and reproductory innovations in the early members of the Holostei and Teleostei [174,175,179]. Several of these key innovations were also acquired by the so-called subholosteans, allegedly as a consequence of convergent evolution [175,179]. However, the systematic position of the taxa regarded as subholosteans and their phylogenetic relationships with the crown-neopterygians are still unclear, and at least some subholostean groups might be on the stem lineage to crown-Neopterygii [93,180–182]. Therefore, the rapid diversification of the early Mesozoic subholosteans and crown-neopterygians might be part of a single radiation event and should thus not be treated as separate clades, as in most recent diversity studies [174,183]. The hypothesis of a single radiation of Neopterygii including subholosteans is more consistent with published diversity analyses [174], but it should be tested in a phylogenetic framework. Based on the information provided by Romano *et al*. [174] in their table S3 and after the addition of more recently described taxa, we counted 262 species of crown-neopterygian and subholostean taxa in the Early and Middle Triassic only (figure 12). Most of these taxa, however, have never been included in a cladistic analysis and, thus, pending elucidation of their phylogenetic relationships, any hypothesis about the evolution of these groups is largely speculative.

The second radiation of ginglymodians and teleosts indicated by our study during the Early Jurassic might be seen as a part of a faunal recovery after the end-Triassic mass extinction event [184]. However, there is no clear evidence for a significant extinction of actinopterygians around the Triassic–Jurassic boundary [185,186] and these radiations of ginglymodians and teleosts might have started earlier, as is the case of dapediiforms [162], or they might be part of the initial radiation of these groups, which started at the early Triassic. Exploring this topic requires a more complete study of the phylogenetic relationships of dapediiforms (see discussion above) together with the numerous Early Jurassic neopterygians, most of which are in need of revision. It is likewise necessary to complete the apparent long ghost lineage of the crown-Halecomorphi, with several halecomorph taxa from the Late Triassic to Middle Jurassic that have not yet been included in cladistic analyses (e.g. species of the genera ^†^*Caturus*, ^†^*Furo*, ^†^*Heterolepidotus* and ^†^*Osteorhachis* [187]).

**Figure 13.**
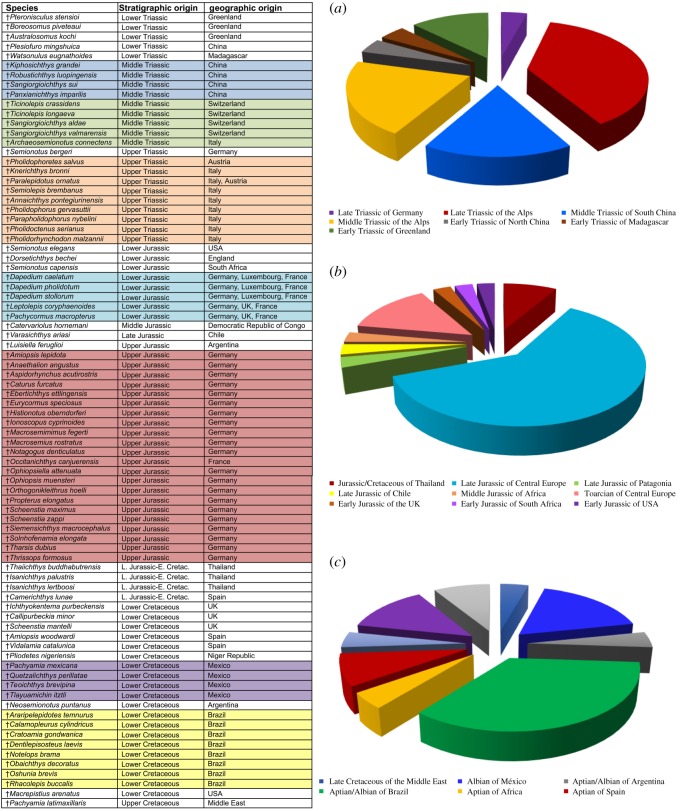
Provenance of the studied taxa highlighting the biases due to the much higher representation of the main Mesozoic Lagerstätte. Table including simplified references to the stratigraphic and geographical provenance of the taxa (left). Pie charts representing the proportions of taxa represented in our data matrix according to their provenance. (*a*) Triassic; (*b*) Jurassic; (*c*) Cretaceous.

Incorporating more Late Triassic to Middle Jurassic neopterygians might also have consequences for the apparent parallel radiation of the three main crown-neopterygian lineages during the Late Jurassic. In our data matrix, these Late Jurassic radiations might be an artefact due to the proportionally high amount of taxa from European Lagerstätten of that age (figure 13).

## 5. Conclusion

The present study was designed and conducted to solve the phylogenetic relationships of the genus ^†^*Ticinolepis*, including two species, ^†^*T. longaeva* and ^†^*T. crassidens*, which is retrieved as the most basal Ginglymodi. ^†^*Ticinolepis* is known from the earliest to late Ladinian, while other more derived taxa are known from the Anisian, thus indicating a ghost lineage for this genus of at least 5 Ma at the base of Ginglymodi (figure 11: Node 3).

Owing to the morphological similarities shared by ^†^*Ticinolepis* with ginglymodians, halecomorphs and teleosts, all crown-neopterygian groups were included in the data matrix, and the cladistic study presented here led to several significant and, partially, novel results. Several cases of homology have been revised and discussed thoroughly.

Some hypotheses of phylogenetic relationships are now confirmed in a more comprehensive taxonomic framework. Among them are the monophyly of Teleostei and Holostei, and its included clades Halecomorphi and Ginglymodi. Within Ginglymodi, the monophyly of Lepisosteiformes and ^†^Semionotiformes is also confirmed, and we propose the name Neoginglymodi for the clade formed by these two orders. At a lower taxonomic level, our analyses retrieve monophyletic Lepisosteoidei and ^†^Lepidotidae within Lepisosteiformes, and monophyletic ^†^Semionotidae, ^†^Callipurbeckiidae and ^†^Macrosemiidae within ^†^Semionotiformes. The sister-group relationship between ^†^Callipurbeckiidae and ^†^Macrosemiidae is here proposed for the first time. Among halecomorphs, the Amiiformes are confirmed to be monophyletic in our hypotheses, but the results of our analyses reject the hypothesis of the monophyly of ^†^Ionoscopiformes. The monophyly of the teleost family ^†^Pholidophoridae is also confirmed, but its systematic position remains unclear. This clade might be more derived than ^†^aspidorhynchiforms, ^†^pachycormiforms and the Jurassic genera ^†^*Catervarioulus* and ^†^*Ichthyokentema*, or it might be the sister-group of all other teleosts.

The relationships of some taxa within the monophyletic clades vary when implied weighting is applied. Most notable is that ^†^*Dapedium* changes from a basal position as the sister-group of all other holosteans, to a position as the sister-group of Ginglymodi. Similarly, the ginglymodian ^†^*Neosemionotus* might be the sister-taxon of Lepisosteiformes or the sister-taxon of ^†^Semionotiformes. The phylogenetic relationships of the Triassic halecomorphs at the base of Halecomorphi are still not clear.

Furthermore, we obtained some challenging results within the Teleostei. The most strikingly of them is probably the ^†^Leptolepid-clade, which is well supported and reiterates the hypothesis of the family Leptolepidae of Nybelin [173]. The controversial results obtained for the phylogenetic relationships of the well-known Jurassic genera ^†^*Varasichthys* and ^†^*Thrissops* should stimulate further research on the corresponding lineages.

Our data matrix, like most previous evolutionary studies of Mesozoic actinopterygians, is strongly biased because most of the included taxa come from a few main Mesozoic Lagerstätten, which might be responsible for the hypothetical pattern of radiations described above. More taxa of other geographical and stratigraphic provenance should be incorporated into the analysis to achieve more robust hypotheses about the evolution of neopterygians during the Mesozoic. One aim of this work has also been to provide a comprehensive dataset to facilitate those utterly needed phylogenetic studies. We have merged previously existing lists of characters, proposed several new characters, discussed several cases of conflicts of homology and have scored ourselves each entry in our data matrix. This has been an enormous effort and is certainly not free of failure, so we hope future scholars will contribute with a critical approach, discussing and improving this dataset with more than the addition of new taxa.

## Data accessibility

Dryad Digital Repository: https://doi.org/10.5061/dryad.2tp53gr [58].

Phylogenetic data: MorphoBank, Project 2196 (https://morphobank.org/index.php/MyProjects/List/select/project_id/2196).

The datasets supporting this article have been uploaded as part of the electronic supplementary material:

Electronic supplementary material file S1: Material examined and data matrix; file S2: List of phenotypic characters; file S3: Branch support values obtained for the cladistic analyses; file S4: List of synapomorphies.

## Supplementary Material

Data

## Supplementary Material

Characters

## Supplementary Material

Branch support

## Supplementary Material

Optimization
