# Supplementary material for: Neopterygian phylogeny: the merger assay
Source: R Soc Open Sci. 2018 Mar 21;5(3):172337. doi: 10.1098/rsos.172337 (PMC5882744; doi:10.1098/rsos.172337)
Supplement: Data [file rsos172337supp1.pdf]

Supplementary File S1: Material examined and data matrix to

Neopterygian Phylogeny: the merger assay

Adriana López-Arbarello[1]*, and Emilia Sferco[2]

[1]Department of Earth- and Environmental Sciences, Palaeontology & Geobiology and GeoBio-Center, Ludwig Maximilian University, Richard-Wagner-Strasse 10, D-80333 Munich, Germany (a.Lopez-Arbarello@lrz.uni-muenchen.de)

[2]CICTERRA-CONICET-UNC. Av. Velez Sarsfield 1611, X0516GCA, Córdoba, Argentina.

Institutional abbreviations.

AMNH, American Museum of Natural History, New York, USA; **BGS.GSM,** British Geological Survey, London, UK; **SNSB-BSPG,** Bayerische Staatssammlung für Paläontologie un Geologie, München, Germany; **GMPKU,** Geological Museum of Peking University, Beijing, China; **GZG.V,** Geowissenschaftliches Zentrum der Georg-August-Universität, Göttingen, Germany; **IGM,** Instituto de Geología, Universidad Nacional Autónoma de México; **JM,** Jura-Museum Eichstätt, Germany; **MB,** Museum für Naturkunde, Leibniz-Institut für Evolutions- und Biodiversitätsforschung an der Humboldt-Universität, Berlin, Germany; **MHIN-UNSL-GEO,** Museo de Historia Natural de la Universidad Nacional de San Luis, San Luis, Argentina; **MHNH,** Muséum d'histoire naturelle, Le Havre, France; **MMP,** Museo Municipal de Ciencias Naturales de Mar del Plata, Argentina; **MNHN,** Muséum National d'Histoire Naturelle, Paris, France; **NHMUK,** Natural History Museum, London, UK; **NMB,** National Museum, Bloemfontein, South Africa; **NMC,** Naturkunde-Museum Coburg, Coburg, Germany; **SMF,** Senckenberg Forschungsinstitut und Naturmuseum, Frankfurt am Main, Germany; **SMNS,** Staatliches Museum für Naturkunde, Stuttgart, Germany; **TRF,** Helmut Tischlinger, private collection, Germany; **UBA,** Universidad de Buenos Aires, Vertebrate Collection, Buenos Aires, Argentina.

Taxa included in the cladistic analyses

Amia calva: UBA P.670 and Grande and Bemis (1).
†*Amia pattersoni*: based on Grande and Bemis (1).
†*Amia scutata*: based on Grande and Bemis (1).
†*Amiopsis lepidota*: JME SOS.2966, JME ETT.852, SMNS 59759, SMNS 80251, SNSB-BSPG AS.I.676, SNSB-BSPG AS.VI, SNSB-BSPG AS.VII.327, SNSB-BSPG 1964.XXIII.550, SNSB-BSPG 1964.XXIII.551, SNSB-BSPG 1966.XXX.19, and Grande and Bemis (1).
†*Amiopsis woodwardi*: based on Grande and Bemis (1).
†*Anaethalion angustus*: based on Gaudant (2), Patterson and Rosen (3), Arratia (4-7), and Poyato-Ariza (8).
†*Annaichthys pontegiurinensis*: based on Arratia (9, 10).
†*Araripelepidotes temnurus*: MNHN BCE.334, MNHN BCE.335, MNHN BCE.336, MNHN BCE.399, MNHN BCE.340, SNSB-BSPG 1965.I.132 and detailed high quality photographs of AMNH 11813, AMNH 11832, AMNH 11833, AMNH 12716, and AMNH 11614.
†*Archaeosemionotus connectens*: SMF-P1238 (holotype), PIMUZ A/I552.
†*Aspidorhynchus acutirostris*: SNSB-BSPG 2012.I.1 (neotype), SNSB-BSPG AS.V.509, SNSB-BSPG AS.VII.1107-1109, SNSB-BSPG 1903.I.501, SNSB-BSPG 1959.I.71, SNSB-BSPG 1964.XXIII.529-532, SNSB-BSPG 1964.XXIII.539, SNSB-BSPG 1964.XXIII.542, SNSB-BSPG 1972.XX.141, SNSB-BSPG 1977.IX.3, SNSB-BSPG 1985.I.3, JME SOS.440, JME SOS.1396, JME SOS.2255, JME 197 III 6, JME SOS.2734, JME SOS.2804, JME SOS.2827, JME SOS.2829,

JME SOS.2833, JME SOS.3260, JME SOS.3465, JME SOS.3920, JME SOS.3948, JME SOS.3949, JME SOS.3953, JME SOS.4118, JME SOS.4297, JME SOS.4377, JME SOS.4609, JME SOS.8292, MB.f. 1376, MB.f. 2858, MB.f. 3526, MB.f. 3527, MB.f.3529, MB.f.3530, MB.f.18104, NHMUK PV 37068, NHMUK PV 37802, NHMUK PV P7810, SMNS 80253, SMNS 86705.

†*Atractosteus spatula*: based on Grande (11).

†*Australosomus kochi*: based on Stensiö (12) and Nielsen (13).

†*Boreosomus piveteaui*: based on Nielsen (14).

Brycon meeki: based on Weitzman (15), Mirande (16).

†*Calamopleurus cylindricus*: based on Grande and Bemis (1, 17).

†*Callipurbeckia minor*: BGS.GSM 27975 (neotype), MB.f.1617–9, NHMUK PV P. 6343, NHMUK PV P. 8047, NHMUK PV P. 29398, NHMUK PV P. 28399, NHMUK PV 19006, NHMUK PV 21349, NHMUK PV 21974, NHMUK PV 36080, NHMUK PV 44848, NHMUK PV 48255, NHMUK PV 48341.

†*Camerichthys lunae*: based on Bermúdez-Rochas and Poyato-Ariza (18).

†*Catervariolus hornemani*: based on Taverne (19) and Arratia (9, 10).

†*Caturus furcatus*: JME ETT.3366, NHMUK PV 20578, NHMUK PV 37906, SNSB-BSPG AS.VII.263, SNSB-BSPG 1950.XXXV.31.

†*Cratoamia gondwanica*: based on Brito et al. (17).

†*Cuneatus cuneatus*: based on Grande (11).

†*Cuneatus wileyi*: based on Grande (11).

†*Cyclurus gurleyi*: based on Grande and Bemis (1).

†*Cyclurus kehreri*: based on Grande and Bemis (1).

†*Dapedium caelatum*: SMNS 56226, and Thies and Hauff (20).

†*Dapedium pholidotum*: based on Thies and Waschkewitz (21).

†*Dapedium stollorum*: SNSB-BSPG 1949.XV.22 and Thies and Hauff (22).

†*Dentilepisosteus laevis*: based on Wenz and Brito (23) and Grande (11).

†*Diplomystus dentatus*: SNSB-BSPG 1889.I.18, SNSB-BSPG 1889.I.503, SNSB-BSPG 1957.I.128, SNSB-BSPG 2008.I.81, Patterson and Rosen (3), Grande (24, 25) and Arratia (5-7).

†*Dorsetichthys bechei*: based on Nybelin (26) and Patterson (27, 28).

†*Ebertichthys ettlingensis*: JME ETT.2940, JME ETT.3119, and Arratia (29).

Elops saurus: Nybelin (30), Forey (31), Taverne (32) and Arratia (4, 6, 7).

†*Eurycormus speciosus*: JME SOS.2339, JME SOS.2341, JME SOS.4614a-b, JME SOS.4615, SMNS 80144/37, SMNS 87848/21, SMNS 88987/3, SMNS 95445/11, SMNS 95445/12, SNSB-BSPG AS.I.1275, SNSB-BSPG AS.V.510, SNSB-BSPG 1960.XVIII.61, SNSB-BSPG 1960.XVIII.106, Arratia and Schultze (33) and Arratia (9, 10).

Hiodon alosoides: based on Greenwood (34), Li and Wilson (35, 36) and Hilton (37, 38).

†*Histionotus oberndorferi*: SNSB-BSPG AS.XIX.1 and Bartram (39).

†*Ichthyokentema purbeckensis*: based on Patterson and Griffith (40) and Patterson (28).

†*Ionoscopus cyprinoides*: SNSB-BSPG AS.I.50 (holotype), SNSB-BSPG 1903.I.64, SNSB-BSPG 1960.XVIII.53, SNSB-BSPG 1960.XVIII.62, SNSB-BSPG 1964.XXIII.141, Grande and Bemis (1), and Maisey (41).

†*Isanichthys lertboosi*: based on Deesri et al. (43).

†*Isanichthys palustris*: based on Cavin and Suteethorn (44).

†*Knerichthys bronni*: based on Arratia (9, 10).

†*Kyphosichthys grandei*: based on Xu and Wu (45) and Sun and Ni (46).

†*Lepidotes gigas*: MB.f.1372, MB.f.1495, MB.f.1519, MNHN HLZ.32, NHMUK PV P.14539, SNSB-BSPG AS.I.625, SNSB-BSPG 1940.I.8, and SNSB-BSPG 2015.I.82.

Lepisosteus osseus: UBA P.671 and Grande (11).

†*Leptolepis coryphaenoides*: SMNS 87371, SMNS 87356, Nybelin (42), Patterson (28), Patterson and Rosen (3), and Arratia (5-7).

†*Luisiella feruglioi*: MACN 13026-13056, MACN 13058-13069, MACN 14430, MACN 14431, 14435, MLP 35.III.1-4, MLP 67.III.1-1 a MLP 67.III.1-15, MLP 67.III.1-17 to MLP 67.III.1-21, MLP 67.III.1-23 to MLP 67.III.1-26, MLP 67.III.1-29, MLP 67.III.1-30, MLP 67.III.1-32 to MLP 67.III.1-35, MLP 67.III.1-37, MLP 67.III.1-40 to MLP 67.III.1-45, MLP 67.III.1-49 to MLP

67.III.1-53, MLP 67.III.1-56 to MLP 67.III.1-60, MLP 67.III.1-63, MLP 67.III.1-65, MLP 67.III.1-66, MLP 67.III.1-68, MLP 67.III.1-70 a MLP 67.III.1-74, MLP 76- X.7-16/2, MLP 92.XI.30-1 to MLP 92.XI.30-8, 89.XI.30-1, MPEF-PV 1354, MPEF-PV 1385, MPEF-PV 1388, MPEF-PV 1389, MPEF-PV 1469#3-5, MPEF-PV 1469#7-10, MPEF-PV 1470#2, MPEF-PV 1470#3, MPEF-PV 1470#5, MPEF-PV 1471, MPEF-PV 1472#3-4, MPEF-PV 1472#6, MPEF-PV 1472#8-10, MPEF-PV 1473, MPEF-PV 1474#1-8, MPEF-PV 1475#5, MPEF-PV 1476#1-4, MPEF-PV 1476#6-8, MPEF-PV 1476#11, MPEF-PV 1477#4, MPEF-PV 1477#6-7, MPEF-PV 1478#1, MPEF-PV 1478#3-6, MPEF-PV 1478#8, MPEF-PV 1480-1483, MPEF-PV 1484#1-2, MPEF-PV 1484#5, MPEF-PV 1484#7-9, MPEF-PV 1484#11, MPEF-PV 1485#3-5, MPEF-PV 1485#7-8, MPEF-PV 1486 A-B, MPEF-PV 1487#1-2, 1487#4-7, 1487#10, MPEF-PV 1488#1-8, MPEF-PV 1489#2-5, MPEF-PV 1489#9, MPEF-PV 1491#1-2, MPEF-PV 1491#4, MPEF-PV 1491#6, MPEF-PV 1492#3, MPEF-PV 1493#5, 1494#1, MPEF-PV 1494#3, MPEF-PV 1608, MPEF-PV 1611, MPEF-PV 1654, MPEF-PV 1768-1769, MPEF-PV 3096-3097, MPEF-PV 3170, MPEF-PV 3185-3186, MPEF-PV 3192-3193, MPEF-PV 3195-3196, MPEF-PV 3198, MPEF-PV 3200, MPEF-PV 3202, MPEF-PV 3411-3415, MPEF-PV 3417- MPEF-PV 3421, MPEF-PV 3426, MPEF-PV 3428-3429, MPEF-PV 3431-3432, MPEF-PV 3837, MPEF-PV 3904-3905, MPEF-PV 3908-3909, MPEF-PV 3911-3912, MPEF-PV 3930-3939, MPEF-PV 3942-3949, MPEF-PV 3950-3953, MPEF-PV 3956-3957, MPEF-PV 3960, MPEF-PV 3962-3967, MPEF-PV 3977, MPEF-PV 3979-3981, MPEF-PV 3983-3987, MPEF-PV 3422-3425, MPEF-PV 3427, MPEF-PV 3846-3849, MPEF-PV 3850-3856, MPEF-PV 3858-3861, MPEF-PV 3864-3873, MPEF-PV 3875-3877, MPEF-PV 3879-3885, MPEF-PV 3887-3888, MPEF-PV 3891, MPEF-PV 3906, MPEF-PV 3910, MPEF-PV 3927-3928, MPEF-PV 3940-3941, MPEF-PV 3954-3955, MPEF-PV 3959, MPEF-PV 3961, MPEF-PV 3968-3969, MPEF-PV 3974, MPEF-PV 3982, MPEF-PV 3986, MPEF-PV 3988-3989, MPEF-PV 3433, MPEF-PV 10534-10535.

†*Macrepistius arenatus*: based on Schaeffer (43, 44).

†*Macrosemimimus fegerti*: JME ETT.854 (holotype), JME ETT.67, JME ETT.77, JME ETT.172, JME ETT.244, JME ETT.853, JME ETT.888, JME ETT.978, JME ETT.1351.

†*Macrosemius rostratus*: SNSB-BSPG AS.I.770, SNSB-BSPG 1986.XV.119, Bartram (39) and Ebert et al. (45).

†*Masillosteus janeae*: based on Grande (11).

†*Masillosteus kelleri*: based on Micklich and Klappert (46) and Grande (11).

†*Neosemionotus puntanus*: MMP 1114 (holotype), MHIN-UNSL-GEO.V8, MHIN-UNSL-GEO.V64, MHIN-UNSL-GEO.V68, MHIN-UNSL-GEO.V183–185, MHIN-UNSL-GEO.V187–191.

†*Notagogus denticulatus*: SNSB-BSPG AS.I.768 (holotype), SNSB-BSPG 1967.I.303, SNSB-BSPG 1986.XV.120 and Bartram (39).

†*Notelops brama*: based on Forey (47), Maisey (48).

†*Obaichthys decoratus*: based on Wenz and Brito (23) and Grande (11).

†*Occitanichthys canjuersensis*: MNHN CNJ89 (holotype), NHMUK PV P.1118, PV 41157.

†*Ophiopsiella attenuata*: JME ETT.1896 (neotype), JME ETT.3365, JME ETT.3370, and Lane and Ebert (49).

†*Ophiopsis muensteri*: JME ETT.977, JME ETT.2077, SNSB-BSPG AS.VII.1135 (holotype), SNSB-BSPG AS.VII.267, SNSB-BSPG 1870.IV.2, SNSB-BSPG 1993.VXIII.12, and Lane and Ebert (50).

†*Orthogonikleithrus hoelli*: JME SOS.3954, JME SOS.3955, JME SOS.3957, JME SOS.3961, JME SOS.3964, Arratia (6) and Konwert (51).

†*Oshunia brevis*: based on Maisey (41, 48), and Grande and Bemis (1).

†*Pachyamia latimaxillaris*: based on Grande and Bemis (1).

†*Pachyamia mexicana*: based on Grande and Bemis (1).

†*Pachycormus macropterus*: based on Patterson (52), Mainwaring (53), and Arratia and Lambers (54).

†*Panxianichthys imparilis*: based on Xu and Shen (55) and Sun et al. (56).

†*Paralepidotus ornatus*: SNSB-BSPG 2003.XXIX.218, SNSB-BSPG 2003.XXIX.632, SNSB-BSPG 2007.I.62 (several specimens) and Tintori (57).

†*Parapholidophorus nybelini*: based on Arratia (9, 10).

†*Pholidoctenus serianus*: based on Arratia (9, 10).

†*Pholidophoretes salvus*: based on Arratia (9, 10).

†*Pholidophorus gervasuttii*: based on Arratia (9, 10).
†*Pholidorhynchodon malzanni*: based on Arratia (9, 10).
†*Plesiofuro mingshuica*: based on Xu et al. (58, 59).
†*Pliodetes nigeriensis*: MNHN GDF.1275 (holotype), MNHN GDF.1274, MNHN GDF.1276–78, MNHN GDF.1285, MNHN GDF.1288 and Wenz (59).
†*Propterus elongatus*: SNSB-BSPG AS.I.767 (holotype), SNSB-BSPG 1963.I.173, SNSB-BSPG 1964.XXIII.145, SNSB-BSPG 1986.XV.121, SNSB-BSPG 2011.I.138 and Bartram (39).
†*Pteronisculus stensioi*: based on Nielsen (14).
†*Quetzalichthys perrilliatae*: based on Alvarado-Ortega and Espinosa-Arrubarrena (60).
†*Rhacolepis buccalis*: SNSB-BSPG 1967.I.159, SNSB-BSPG 1972.I.16, SNSB-BSPG 1972.I.17, SNSB-BSPG 1975.I.167, SNSB-BSPG 1989.XII.209, Forey (47), and Maisey (48) (1991).
†*Robustichthys luopingensis*: based on Xu et al. (61).
Salmo salar: high quality images of MNHN-D-17 through OsteoBase (62), and NHMUK 1880.2.16, NHMUK 2017.4.4.2, and NHMUK 2017.4.4.3 through the Natural History Museum Data Portal (data.nhm.ac.uk), and Sanford (63).
†*Sangiorgioichthys aldae*: MCSN 5050 (holotype) and Tintori and Lombardo (64).
†*Sangiorgioichthys sui*: GMPKU P.1568 (holotype), GMPKU P.1357–9, GMPKU P.1472, GMPKU P.1558, GMPKU P.1567-8, GMPKU P.1609, GMPKU P.1636, GMPKU P.1642, GMPKU P.1656, GMPKU P.1661, GMPKU P.1695, GMPKU P.1707, GMPKU P.1973, GMPKU P.1995.
†*Sangiorgioichthys valmarensis*: MCSN 8425 (holotype)
†*Scheenstia mantelli*: NHMUK PV 2397, NHMUK PV 2401, NHMUK PV 2456 (holotype), NHMUK PV 3036–7, NHMUK PV 3048–49, NHMUK PV 3517–8, NHMUK PV 4916, NHMUK PV P.6336, NHMUK PV P.6344–5, NHMUK PV P.6348c, NHMUK PV P.6362, NHMUK PV P.6933, NHMUK PV P.7333, NHMUK PV P.11832–3, NHMUK PV P.14451.
†*Scheenstia maximus*: SMF P.2386 (neotype), P.325.
†*Scheenstia zappi*: JME Scha.80 (holotype).
†*Semiolepis brembanus*: based on Lombardo and Tintori (65) and *†Semiolepis* sp. NHMUK PV P.10943.
†*Semionotus bergeri*: SNSB-BSPG AS.I.512, 1960.I.25, 1960.XVI.2, GZG.V.010.001 (lectotype), GZG.V.010.002-4, MB f. 1518 (4 specimens), NMC 1264, 15128a–b, 15129, SMF P6108.
†*Semionotus capensis*: MB f. 12240 (9 specimens), NHMUK PV P4089 (4 specimens), P56020 (7 specimens), SNSB-BSPG 1908.I.502, photographs of several specimens in the NMB.
†*Semionotus elegans*: based on Olsen and McCune (66).
†*Siemensichthys macrocephalus*: SNSB-BSPG AS.I.1134 (holotype), SNSB-BSPG AS.7-873, SNSB-BSPG 1929.I.25, SMNS 80144/3, SMNS 80440/43, Patterson (28) and Arratia (9, 10, 67).
†*Solnhofenamia elongata*: SNSB-BSPG AS.VII.324 (holotype), SNSB-BSPG AS.VI.27, SNSB-BSPG AS.VI.28, SNSB-BSPG AS.VII.326, SNSB-BSPG 1875.XIV 332, and Grande and Bemis (1).
†*Teoichthys brevipinna*: based on Machado et al. (68).
†*Thaiichthys buddhabutrensis*: based on Cavin et al. (69).
†*Tharsis dubius*: CPBA V.1383, CPBA V.1502, CPBA V.1505, JME SOS.3899, MB.f.15685, MB.f.2831, SNSB-BSPG 1986.XV.125, SNSB-BSPG 1953.I.9, SNSB-BSPG 1953.I.97, SNSB-BSPG 1954.I.226, SNSB-BSPG 1964.XXIII.395, SNSB-BSPG 1964.XXIII.399, SNSB-BSPG 1964.XXIII.407, SNSB-BSPG 1964.XXIII.415, SNSB-BSPG 1964.XXIII.417, SNSB-BSPG 1964.XXIII.414, SNSB-BSPG 1964.XXIII.430, SNSB-BSPG 1964.XXIII.280, SNSB-BSPG 1964.XXIII.160, SNSB-BSPG 1986.I.41, SNSB-BSPG 1986.I.37, SNSB-BSPG 1960.XVIII.90, SNSB-BSPG 1960 XVIII 91, SNSB-BSPG 1960.XVIII.97, SNSB-BSPG 1960.XVIII.98, SNSB-BSPG 1961.III.23, SNSB-BSPG 1961.I.466, BSPG 1964.XXIII.384, SNSB-BSPG 1968.XV.29, SNSB-BSPG 1969.XVI.32, SNSB-BSPG 1969.XVI.39, SNSB-BSPG 1994.V.5, SNSB-BSPG AS.VI.25, Nybelin (42), Patterson (28), Patterson and Rosen (3), and Arratia (5).
†*Thrissops formosus*: JME ETT.46–47, JME ETT.73–76, JME ETT.87, JME ETT.126, JME ETT.211, ETT.283, ETT.564, ETT.867, JME ETT.879, JME ETT.887, JME ETT.972, JME ETT.1350, JME ETT.1805, JME SOS.7849, MNHN-CRN 61, SNSB-BSPG AS.VII.2 (holotype), SNSB-BSPG AS.VII.175, SNSB-BSPG AS.VII.178, SNSB-BSPG 1993.XVIII.P15, Nybelin (70), and Patterson and Rosen (3).

†*Ticinolepis crassidens*: PIMUZ T273 (holotype), PIMUZ T237, PIMUZ T240, PIMUZ T248, PIMUZ T256-257, PIMUZ T265, PIMUZ T277-278, PIMUZ T315, PIMUZ T331, PIMUZ T341, PIMUZ T358, PIMUZ T401, PIMUZ T413, PIMUZ T438, PIMUZ T638, and PIMUZ T4350.

†*Ticinolepis longaeva*: MCSN 8072 (holotype), MCSN 8001, MCSN 8007–9, MCSN 8011, MCSN 8073, MCSN 8077, MCSN 8083–4, MCSN 8086, MCSN 8412–13, MCSN 8415, PIMUZ T1789, PIMUZ T4785, PIMUZ T4788-4790, PIMUZ T4987.

†*Tlayuamichin itztli*: IGM 6716 (holotype), IGM 6717–20.

†*Varasicthys ariasi*: Arratia (71-73).

†*Vidalamia catalunica*: based on Grande and Bemis (1).

†*Watsonulus eugnathoides*: based on Olsen (74) and Grande and Bemis (1).

Additional taxa not included in the analyses

†*Anaethalion knorri*: JME SOS.2267a, JME SOS.2282, JME SOS.2270a, JME SOS.2281, JME SOS.2280, SNSB-BSPG 1960.XVIII.93, SNSB-BSPG 1964.XXIII.394, SNSB-BSPG 1969.XVI.31, SNSB-BSPG 1969.XI.39, SNSB-BSPG 1968.XV.129, SNSB-BSPG 1991.I.122, SNSB-BSPG 1968.XV.3, SNSB-BSPG AS.I.1473.

†*Belonostomus tenuirostris*: SNSB-BSPG AS.I.1207, SNSB-BSPG 1961.I.212.

†*Belonostomus speciosus*: SNSB-BSPG AS.VII.1069.

†*Cavenderichthys talbragarensis*: MB.f.4917, MB.f.5001, MB.f.5006, MB.f.5004, MB.f.5005, MB.f.5002, MB.f.5003. NHMUK PV 12424-12439, NHMUK PV 12441-12449, NHMUK PV 12450-12451, NHMUK PV 37973-37977, NHMUK PV 18113, SNSB-BSPG 1939.X.321, TRF 68, TRF 60, TRF 61, TRF 64.

†*Lepidotes elvensis*: MNHN JRE.250, MNHN JRE.251, MNHN JRE.254, MNHN JRE.545 (holotype).

†*Lepidotes semiserratus*: NHMUK PV P.3528, NHMUK PV P.7410, NHMUK PV 35556, NHMUK PV 62939.

†*Leptolepides haertesi*: JME SOS.2473, JME SOS.2471, JME SOS.2554.

†*Leptolepides sprattiformis*: CPBA V.282, JME SOS.2305, JME SOS.2422, JME SOS.2463, JME SOS.2472, JME SOS.2956.

†*Macrosemimimus lennieri*: MHNH PV 7267 (holotype of *Lepidotes lennieri*).

†*Lepidotes toombsi*: NHMUK PV P.2518 (holotype), NHMUK PV P.34511.

Lepisosteus sp.: MB.f.18498.

†*Macrosemius fourneti*: SNSB-BSPG AS.I.768 (holotype).

†*Notagogus decoratus*: SNSB-BSPG 1986.XV.119.

†*Obaichthys africanus*: SNSB-BSPG 1993.IX.320 (scales).

†*Orthogonikleithrus leichi*: JME SOS.2301, JME SOS.2632.

†*Propterus microstomus*: SNSB-BSPG AS.VII.268 (holotype), SNSB-BSPG AS.I.637, SNSB-BSPG 2011.I.139.

†*Scheenstia decoratus*: SNSB-BSPG AS.VI.3 (holotype).

†*Scheenstia degenhardti* : MB.f.1499 (holotype).

†*Scheenstia laevis*: MNHN CRN.61.

†*Siemensichthys* sp.: SMNS 80144/3, SMNS 86214/20, SMNS 87316/10, SMNS 95398/6.

†*Vinctifer comptoni*: SNSB-BSPG 1971.I.169a-b, SNSB-BSPG 1972.I.14, SNSB-BSPG 1972.I.28, CPBA V.14066.

References

1. Grande L, Bemis WE. A comprehensive phylogenetic study of amiid fishes (Amiidae) based on comparative skeletal anatomy: An empirical search for interconnected patterns of natural history. Memoirs of the Society of Vertebrate Paleontology. 1998;4:I-X, 1-690.
2. Gaudant J. Contribution à une révision des Anaethalion de Cérin (Ain). Bulletin Bureau de Recherche Geologique et mineralogique. 1968;2:5-15.
3. Patterson C, Rosen DE. Review of ichthyodectiform and other Mesozoic teleost fishes and the theory and practice of classifying fossils. Bulletin of the American Museum of Natural History. 1977;158(2):81-172.
4. Arratia G. *Anaethalion* and similar Teleosts (Actinopterygii, Pisces) from the Late Jurassic (Tithonian) of Southern Germany and their relationships. Palaeontographica Abt A. 1987;200(1-3):1-44.
5. Arratia G. The cuadal skeleton of Jurassic teleosts: a phylogenetic analysis. In: Chang M-M, Liu H, Zhang G, editors. Early vertebrates and related problems in evolutionary biology: Beiging: Science Press; 1991. p. 249-340.
6. Arratia G. Basal teleosts and teleostean phylogeny. Palaeo Ichthyologica. 1997;7:5-168.
7. Arratia G. The monophyly of Teleostei and stem-group teleosts. Consensus and disagreements. In: Arratia G, Schultze H-P, editors. Mesozoic Fishes 2 Systematics and Fossil Record. München: Verlag Dr. Friedrich Pfeil; 1999. p. 265-334.
8. Poyato-Ariza FJ. The elopiform fish *Anaethalion* a*ngustus* restored, with comments on individual variation. In: Arratia G, Schultze H-P, editors. Mesozoic Fishes 2 – Systematics and Fossil Record. Munich, Germany: Verlag Dr Friedrich Pfeil; 1999. p. 361-8.
9. Arratia G. Morphology, taxonomy, and phylogeny of Triassic pholidophorid fishes (Actinopterygii, Teleostei). Journal of Vertebrate Paleontology. 2013;33:1-138.
10. Arratia G. NEW TRIASSIC TELEOSTS (ACTINOPTERYGII, TELEOSTEOMORPHA) FROM NORTHERN ITALY AND THEIR PHYLOGENETIC RELATIONSHIPS AMONG THE MOST BASAL TELEOSTS. Journal of Vertebrate Paleontology. 2017;37(2):24.
11. Grande L. An empirical synthetic pattern study of gars (Lepisosteiformes) and closely related species, based mostly on skeletal anatomy. The resurrection of Holostei. American Society of Ichthyologists and Herpetologists, Special Publication. 2010(6):1-863.
12. Stensiö E. Triassic fishes from east Greenland collected by the Danish Expeditions in 1929-1931. Meddelelser om Grønland. 1932;83(3):1-305.
13. Nielsen E. Studies on Triassic Fishes II: *Australosomus* and *Birgeria*. Meddelelser om Grønland. 1949;146(1):1-309.
14. Nielsen E. Studies on Triassic Fishes I: *Glaucolepis* and *Boreosomus*. Meddelelser om Grønland. 1942;138:1-394.
15. Weitzman SH. The osteology of *Brycon meeki*, a generalized characid fish, with an osteological definition of the family: Division of Systematic Biology, Stanford University; 1962.
16. Mirande JM. Phylogeny of the family Characidae (Teleostei: Characiformes): from characters to taxonomy. Neotropical Ichthyology. 2010;8(3):385-568.
17. Brito PM, Yabumoto Y, Grande L. New Amiid fish (Halecomorphi: Amiiformes) from the lower Cretaceous Crato Formation, Araripe Basin, northeast Brazil. Journal of Vertebrate Paleontology. 2008;28(4):1007-14.
18. Bermudez-Rochas DD, Poyato-Ariza FJ. A new semionotiform actinopterygian fish from the Mesozoic of Spain and its phylogenetic implications. Journal of Systematic Palaeontology. 2015;13(4):265-85.
19. Taverne L. Ostéologie et relations de *Catervariolus* (Teleostei," Pholidophoriformes") du Jurassique moyen de Kisangani (Formation de Stanleyville) en République Démocratique du Congo. Bulletin de l'Institut Royal des Sciences Naturelles de Belgique Sciences de la Terre. 2011;81:175-212.
20. Thies D, Hauff RB. A neotype for Dapedium caelatum Quenstedt, 1858 (Actinpoterygii, Neopterygii, Semionotiformes) from the Early Jurassic (Early Toarcian) of South Germany. Geologica et Palaeontologica. 2008;42:23-38.

21. Thies D, Waschkewitz J. Redescription of Dapedium pholidotum (Agassiz, 1832) (Actinopterygii, Neopterygii) from the Lower Jurassic Posidonia Shale, with comments on the phylogenetic position of Dapedium Leach, 1822. Journal of Systematic Palaeontology. 2016;14(4):339-64.
22. Thies D, Hauff R. A new species of Dapedium Leach, 1822 (Actinopterygii, Neopterygii, Semionotiformes) from the Early Jurassic of South Germany. Palaeodiversity. 2011;4:185-221.
23. Wenz S, Brito PM. First occurrence of lepisosteids (Pisces, Actinopterygii) from the Lower Cretaceous in the Chapada-do-Araripe (N-E Brazil) - implications for the phylogeny of Ginglymodi. Comptes Rendus De L Academie Des Sciences Serie Ii. 1992;314(13):1519-25.
24. Grande L. A revision of the fossil genus *Diplomystus* , with comments on the Interrelationships of Clupeomorph Fishes. American Museum Novitates. 1982;2728:1-34.
25. Grande L. Recent and fossil Clupeomorph fishes with materials for revision of the subgroups of Clupeoids. Bulletin of the American Museum of Natural History. 1985;181(2):231-372.
26. Nybelin O. On certain Triassic and Liassic representatives of the family Pholidophoridae s. str. Bulletin of the British Museum (Natural History),Geology. 1966;11(8):351-432.
27. Patterson C. The caudal skeleton in Lower Liassic Pholidophorid fishes. Bulletin of the British Museum (Natural History), Geology. 1968;16(5):203-39.
28. Patterson C. The braincase of pholidophorid and leptolepid fishes, with a review of the actinopterygian braincase. Philosophical Transactions of the Royal Society of London. 1975;269(899):275-579.
29. Arratia G. New remarkable Late Jurassic teleosts from southern Germany: Ascalaboidae n. fam., its content, morphology, and phylogenetic relationships. Fossil Record. 2016;19(1):31-59.
30. Nybelin O. On the Caudel Skeleton in Elops: With Remarks on Other Teleostean Fishes: Almqvist & Wiksell (distr.); 1971.
31. Forey PL. Relationships of elopomorphs. In: PH G, RS M, C P, editors. Interrelationships of Fishes. London, UK: Academic Press for the Linnean Society of London.; 1973. p. 351-68.
32. Taverne L. L'ostéologie d'Elops Linné, c., 1766 (Pisces Elopiformes) et son intérêt phylogénétique. Mém Acad Roy Cl Sci Collect. 1974;8:1-96.
33. Arratia G, Schultze H-P. *Eurycormus – Eurypoma*: two Jurassic actinopterygian genera with mixed identity. Fossil Record. 2007;10(1):17-37.
34. Greenwood P. Interrelationships of osteoglossomorphs. In: PH G, RS M, C P, editors. Interrelationships of Fishes. London, UK: Academic Press for the Linnean Society of London; 1973. p. 307–32.
35. GQ L, MVH W. Phylogeny of Osteoglossomorpha. In: MLJ S, LD P, GD J, editors. Interrelationships of fishes. San Diego, USA: Academic Press; 1996. p. 163-74.
36. LI G-Q, WILSON MV, editors. Early divergence of Hiodontiformes sensu stricto in East Asia and phylogeny of some Late Mesozoic teleosts from China. Mesozoic fishes 2: systematics and fossil record; 1999; Munich, Germany: Verlag Dr Friedrich Pfeil.
37. Hilton EJ. Osteology of the Extant North American Fishes of the Genus *Hiodon* Lesueur, 1818 (Teleostei: Osteoglossomorpha: Hiodontiformes). Fieldiana: Zoology, New Series. 2002;100:1-142.
38. Hilton EJ. Comparative osteology and phylogenetic systematics of fossil and living bony-tongue fishes (Actinopterygii, Teleostei, Osteoglossomorpha). Zoological Journal of the Linnean Society. 2003;137:1-100.
39. Bartram AWH. The Macrosemiidae, a Mesozoic family of holostean fishes. Bulletin of the British Museum (Natural History), Geology. 1977;29(2):137-234.
40. Griffith J, Patterson C. The structure and relationships of the Jurassic fish *Ichthyokentema purbeckensis*. Bulletin of the British Museum (Natural History), Geology. 1963;8(1):3-43.
41. Maisey JG. The supraotic bone in neopterygian fishes (Osteichthyes, Actinopterygii). American Museum Novitates. 1999;3267:1-52.
42. Nybelin O. A revision of the leptolepid fishes. Acta Regiae Societatis Scientiarum et Litterarum Gothoburgensis, Zoologica. 1974;9:1-202.
43. Schaeffer B. A new actinopterygian fish from the Cretaceous of North America. American Museum Novitates. 1968;2344:1-10.

44. Schaeffer B. The Braincase of the Holostean Fish *Macrepistius*, with Comments on Neurocranial Ossification in the Actinopterygii. American Museum Novitates. 1971;2459:1-34.
45. Ebert M, Lane JA, Kolbl-Ebert M. *Palaeomacrosemius thiollieri*, gen. et sp nov., a new Macrosemiidae (Neopterygii) from the Upper Jurassic of the Solnhofen Archipelago (Germany) and Cerin (France), with a revision of the genus *Macrosemius*. Journal of Vertebrate Paleontology. 2016;36(5):22.
46. Micklich N, Klappert G. *Masillosteus kelleri*, a New Gar (Actinopterygii, Lepisosteidae) from the Middle Eocene of Grube Messel (Hessen, Germany). Kaupia. 2001;11:73-81.
47. Forey PL. The osteology of *Notelops* Woodward, *Rhacolepis* Agassiz and *Pachyrhizodus* Dixon (Pisces: Teleostei). Bulletin of the British Museum (Natural History) - Geology. 1977;28(2):123-204.
48. Maisey JG. Santana Fossils: An Illustrated Atlas. Maisey JG, editor. Neptune City: T.F.H. Publications, Inc.; 1991 //. 1-455 p.
49. Lane JA, Ebert M. A taxonomic reassessment of ophiopsis (Halecomorphi, Ionoscopiformes), with a revision of Upper Jurassic species from the Solnhofen Archipelago, and a new genus of Ophiopsidae. Journal of Vertebrate Paleontology. 2015;35(1).
50. Lane JA, Ebert M. Revision of *Furo muensteri* (Halecomorphi, Ophiopsidae) from the Upper Jurassic of Western Europe, with comments on the genus. Journal of Vertebrate Paleontology. 2012;32(4):799-819.
51. Konwert M. *Orthogonikleithrus francogalliensis*, sp nov (Teleostei, Orthogonikleithridae) from the Late Jurassic Plattenkalks of Cerin (France). Journal of Vertebrate Paleontology. 2016;36(3):10.
52. Patterson C. Interrelationships of holosteans. In: Greenwood PH, Miles RS, Patterson C, editors. Interrelationships of fishes. London: Academic Press; 1973. p. 233-305.
53. Mainwaring AJ. Anatomical and systematic review of the Pachycormidae, a family of Mesozoic fossil fishes. Unpublished: Westfield College; 1978.
54. Arratia G, Lambers P. The caudal skeleton of pachycormiforms: Parallel evolution? In: Arratia G, Viohl G, editors. Mesozoic Fishes - Systematics and Paleoecology. München, Germany: Verlag Dr. Friedrich Pfeil; 1996. p. 191-218.
55. Xu G-H, Shen C-C. *Panxianichthys imparilis* gen. et sp. nov., a new ionoscopiform (Halecomorphi) from the Middle Triassic of Guizhou, China. Vertebrata PalAsiatica. 2015;53(1):1-15.
56. Sun ZY, Tintori A, Xu YZ, Lombardo C, Ni PG, Jiang DY. A new non-parasemionotiform order of the Halecomorphi (Neopterygii, Actinopterygii) from the Middle Triassic of Tethys. Journal of Systematic Palaeontology. 2017;15(3):223-40.
57. Tintori A. *Paralepidotus ornatus* (Agassiz 1833-43): A semionotid from the Norian (Late Triassic) of Europe. In: Arratia G, Viohl G, editors. Mesozoic Fishes: Systematics and Paleoecology. M∞nchen: Verlag Dr. Friedrich Pfeil; 1996. p. 167-79.
58. Xu GH, Gao KQ, Coates MI. Taxonomic revision of *Plesiofuro mingshuica* from the Lower Triassic of Northern Gansu, China, and the relationships of early neopterygian clades. Journal of Vertebrate Paleontology. 2015;35(6):14.
59. Wenz S. †*Pliodetes nigeriensis*, gen. nov. et. sp. nov., a new semionotid fish from the Lower Cretaceous of Gadoufaoua (Niger Republic): phylogenetic comments. In: Arratia G, Schultze H-P, editors. Mesozoic Fishes 2 – Systematics and Fossil Record. München, Germany: Verlag Dr. Friederich Pfeil; 1999. p. 107-20.
60. Alvarado-Ortega J, Espinosa-Arrubarrena L. A new genus of ionoscopiform fish (Halecomorphi) from the Lower Cretaceous (Albian) lithographic limestones of the Tlayua quarry, Puebla, Mexico. Journal of Paleontology. 2008;82(1):163-75.
61. Xu G-H, Zhao L-J, Coates MI. The oldest ionoscopiform from China sheds new light on the early evolution of halecomorph fishes. Biology Letters. 2014;10(5).
62. Osteobase [Internet]. 2016.
63. Sanford CPJ. Salmonoid fish osteology and phylogeny (Teleostei: Salmonoidei). Theses Zoologicae. 2000;33:264 p.
64. Tintori A, Lombardo C. A new early Semionotidae (Semionotiformes, Actinopterygii) from the Upper Ladinian of the Monte San Giorgio area (Southern Switzerland and Northern Italy). Rivista Italiana di Paleontologia e Stratigrafia. 2007;113(3):369-81.

65. Lombardo C, Tintori A. A new semionotid fish (Actinopterygii, Osteichthyes) from the Late Triassic of the Northern Italy. In: Arratia G, Schultze H-P, Wilson MVH, editors. Mesozoic Fishes 4 – Homology and phylogeny. München: Verlag Dr. Friedrich Pfeil; 2008. p. 129-42.
66. Olsen PE, McCune AR. Morphology of the *Semionotus elegans* species group from the Early Jurassic part of the Newark Supergroup of eastern North America with comments on the Family Semionotidae (Neopterygii). Journal of Vertebrate Paleontology. 1991;11(3):269-92.
67. Arratia G. New teleostean fishes from the Jurassic of southern Germany and the systematic problems concerning the 'pholidophoriforms'. Paläontologische Zeitschrift. 2000;74(1/2):113-43.
68. Machado GP, Alvarado-Ortega J, Machado LP, Brito PM. *Teoichthys brevipina*, sp nov., a new ophiopsid fish (Halecomorphi, Ionoscopiformes) from the Lower Cretaceous Tlayua Formation, Central Mexico. Journal of Vertebrate Paleontology. 2013;33(2):482-7.
69. Cavin L, Deesri U, Suteethorn V. Osteology and relationships of *Thaiichthys* nov gen.: a Ginglymodi from the Late Jurassic - Early Cretaceous of Thailand. Palaeontology. 2013;56(1):183-208.
70. Nybelin O. Versuch einer taxonomischen Revision der jurassichen Fischgattung *Thrissops* Agassiz: Meddeland Från Götemborgs. Museui Zoologiska Avdeling. 1964;135:1-44.
71. Arratia G. *Varasichthys ariasi* n. gen. et sp. from the Upper Jurassic of Chile (Pisces, Teleostei, Varasichthyidae n. fam.). Palaeontographica Abteilung A. 1981;175:107-39.
72. Arratia G. Phylogenetic and paleogeographic relationships of the varasichthyid group (Teleostei) from the Late Jurassic of Central and South America. Revista Geológica de Chile. 1994;21(1):119-65.
73. Arratia G. The varasichthyid and other crossognathiform fishes, and the Break-up of Pangaea. In: Longbottom C, L., Richter M, editors. Fishes and the Break-up of Pangaea. 295: Geological Society Special Publications; 2008. p. 71-92.
74. Olsen PE. The skull and pectoral girdle of the parasemionotid fish *Watsonulus eugnathoides* from the Early Triassic Sakamena Group of Madagascar, with comments on the relationships of the holostean fishes. Journal of Vertebrate Paleontology. 1984;4(3):481-99.

Data matrix

†*Pteronisculus stensioi*

1	0	0	0	-	1	0	0	-	0	0	-
1	-	-	?	0	-	-	0	?	?	0	0
-	0	-	-	0	1	0	0	0	0	-	0
0	-	-	0	0	0	0	0	0	0	0	-
0	0	1	0	0	0	0	1	1	0	-	0
0	0	0	0	0	0	0	-	0	0	0	0
?	-	0	?	?	?	0	0	1	0	-	-
-	0	0	0	0	1	0	0	0	1	0	1
0	0	0	0	0	0	1	0	0	0	2	0
0	0	0	0	1	-	-	-	-	-	0	0
0	1	0	0	1	0	2	0	0	0	-	-
-	0	0	1	0	0	1	0	-	0	0	1
0	0	0	0	0	0	0	-	-	0	0	-
0	0	3	0	0	0	0	-	-	0	0	0
-	1	0	0	0	0	-	0	0	0	0	0
0	0	0	0	0	-	0	0	-	-	-	0
-	0	0	0	0	0	0	0	-	-	0	0
-	0	2	1	-	?	0	?	0	0	-	0
1	0	0	0	2	0	0	-	0	0	0	1
-	-	0	?	?	-	0	1	1	1	0	0
0	-	-	?	0	0	?	0	0	?	?	?

?	?	?	?	-	-	-	-	-	?	0	?
1	0	0	0	0	-	0	0	0	-	-	-
-	0	0	0	?	?	-	?	?	0	0	0
?	3	0	1	?	0	?	-	-	-	?	0
2	0	-	-	?	1	0	0	?	?	0	-
-	0	0	?	0	0	1	1	0	1	0	0
0	1	0	0	0	0	0	0	0	0	0	?
0	0	0									

†*Australosomus kochi*

1	0	1	0	-	0	0	1	0	1	0	-
1	-	0	0	0	-	-	0	0	-	0	0
-	0	-	-	0	1	0	0	0	0	-	0
0	-	-	0	0	?	0	0	0	0	0	-
0	0	0	0	2	0	-	0	-	0	-	1
-	0	0	0	0	0	0	-	0	0	0	0
?	-	0	0	?	?	0	0	?	0	-	-
-	0	0	0	0	0	0	0	-	1	-	0
0	0	1	2	1	0	1	0	0	0	2	0
?	?	0	0	1	-	-	-	-	-	-	-
-	-	0	0	1	1	-	-	-	0	-	-
-	0	-	-	-	-	-	?	-	0	1	-
-	-	-	-	-	-	-	-	-	0	0	-
0	0	3	0	0	0	0	-	-	0	0	0
-	1	0	0	0	0	-	1	0	-	-	0
0	0	0	0	-	-	2	1	0	-	-	0
-	0	0	0	0	0	4	0	-	-	0	0
-	0	3	1	-	0	0	0	1	0	-	1
3	0	0	0	-	1	0	-	0	1	0	1
-	-	0	-	0	0	0	0	0	-	-	0
0	-	0	0	0	0	0	0	0	0	0	0
0	0	0	0	-	-	1	0	0	?	0	4
1	0	0	1	0	0	0	0	0	-	-	-
-	0	0	0	0	0	0	0	?	0	0	0
?	3	?	1	?	0	?	0	0	2	-	0
2	0	0	-	0	2	0	1	-	0	0	-
-	0	1	-	0	0	0	0	0	0	2	0
0	1	0	0	0	0	0	0	?	1	0	2
1	1	0									

†*Boreosomus piveteaui*

2	0	0	0	-	?	0	1	0	0	0	-
1	-	0	0	0	-	-	0	0	-	0	0
-	0	-	-	1	1	0	0	0	0	-	0
0	-	-	1	0	0	0	0	0	0	0	-
0	0	0	0	2	1	-	1	1	1	0	0
0	?	0	0	0	0	0	-	0	1	-	-
?	-	0	0	?	?	0	0	1	0	-	-
-	0	1	0	0	1	0	0	3	1	0	0
0	0	0	0	0	0	0	0	0	0	2	0
0	0	0	0	1	-	-	-	-	-	0	0
0	1	0	0	1	1	2	0	0	0	-	-
-	0	0	1	0	0	1	0	-	0	0	?

0	0	0	0	0	0	-	-	-	0	0	-
0	0	3	0	0	0	0	-	-	0	0	0
-	1	0	0	0	0	-	0	0	-	-	0
0	0	0	0	0	0	2	1	0	-	-	0
-	0	0	0	0	0	0	0	-	-	-	0
-	0	3	1	-	?	?	?	?	0	-	0
0	0	1	0	0	0	0	-	0	0	0	1
-	-	0	?	?	?	?	?	?	?	?	?
?	?	?	?	?	?	?	?	?	?	?	?
?	?	?	?	-	-	?	0	?	?	?	?
?	?	?	?	?	?	?	?	?	?	?	?
?	0	0	?	?	0	?	0	?	0	?	?
?	3	0	1	?	0	?	-	-	0	-	0
1	0	0	-	?	0	0	?	-	?	0	-
-	0	0	?	1	0	0	0	0	0	3	1
0	0	?	?	0	?	?	0	?	0	0	2
0	?	0									

Amia calva

3	0	0	1	-	-	-	-	-	0	-	-
0	-	-	0	0	-	-	0	1	0	2	1
1	1	0	0	0	1	1	2	0	1	1	1
1	1	0	1	1	0	0	1	0	0	1	0
1	2	0	0	1	0	-	1	0	1	0	0
0	0	1	0	0	0	0	1	0	1	-	-
0	0	0	0	0	-	0	0	2	1	1	0
2	0	0	0	2	0	0	0	2	0	0	1
0	0	0	1	0	0	1	0	0	0	0	0
2	1	1	0	1	-	-	-	-	-	1	0
0	1	3	0&1	2	0	2	0	1	0	-	-
-	0	0	2	1	0	0	1	4	1	1	-
-	-	-	-	-	-	0	1	1	0	1	1
0	1	0	0	1	3	1	0	0	0	0	1
0	1	2	0	0	1	0	0	0	0	0	0
0	0	0	0	0	0	2	0	0	-	0	0
-	0	0	0	0	1	2	1	1	1	1	1
0	1	2	1	0	1	1	2	1	1	0	1
0	0	0	0	1	0	1	0	2	0	0	1
2	-	2	0	0	0	0	1	1	1	0	0
0	-	0	0	0	0	0	0	0	0	1	0
0	0	0	0	0	0	1	0	0	0	2	3
1	3	1	0	0	1	0	0	0	-	-	-
-	1	1	0	0	3	2	0	2	0	0	0
0	2	1	0	-	2	0	0	0	2	-	1
3	2	0	0	1	0	2	2	1	1	1	3
3	0	1	-	0	0	0	0	0	0	1	0
1	1	0	1	1	0	0	0	0	1	0	0
1	0	0									

†*Amia pattersoni*

3	0	0	1	-	-	-	-	-	0	-	-
?	?	?	?	0	?	?	?	?	?	2	?
1	?	?	0	0	?	?	2	0	1	1	1

1	1	?	?	1	0	?	?	?	0	1	0
1	2	0	0	1	?	-	1	0	1	0	0
0	?	1	0	0	?	0	1	0	1	-	-
0	0	?	?	?	?	?	?	?	1	1	0
?	?	?	0	2	0	0	0	1	0	0	?
0	0	0	0	0	0	1	0	0	0	0	0
2	1	1	0	1	-	-	-	-	-	1	0
0	1	2	0	1	0	2	0	1	0	-	-
-	0	0	2	1	0	0	1	4	1	1	-
-	-	-	-	-	-	0	?	1	0	1	1
0	1	0	0	1	3	1	0	0	0	0	1
0	1	?	0	0	1	0	?	0	0	0	0
0	0	0	0	?	?	2	0	0	-	0	0
-	0	0	0	0	0	2	1	1	1	1	1
0	1	2	1	1	?	?	?	?	?	?	?
0	?	0	0	?	?	0	0	2	0	0	1
2	-	2	0	0	0	0	1	1	?	?	0
0	-	0	0	0	0	0	0	0	0	1	?
?	?	?	-	0	0	1	0	0	?	4	4
?	?	?	0	0	1	0	0	0	-	-	-
-	1	1	0	0	1	2	?	0	0	0	0
0	2	1	0	-	2	0	0	0	2	-	1
3	2	0	?	?	0	2	?	?	?	?	3
3	0	1	-	0	0	0	0	0	0	1	0
0	1	?	?	1	?	0	?	?	?	0	0
?	0	0									

†*Amia scutata*

3	0	0	1	-	-	-	-	-	0	-	-
?	?	?	?	0	?	?	?	?	?	2	?
1	?	?	?	?	?	?	?	0	1	1	1
1	1	0	?	1	0	?	?	?	0	1	0
1	?	?	?	?	?	?	?	?	1	0	?
0	?	1	0	0	?	?	?	0	1	-	-
0	?	?	?	?	?	?	?	2	1	1	0
?	?	?	?	?	0	0	0	2	0	0	1
0	0	0	0	0	0	1	0	0	0	0	0
2	1	1	0	1	-	-	-	-	-	1	0
0	1	3	0	2	0	2	0	1	0	-	-
-	0	0	2	1	0	0	1	4	1	1	-
-	-	-	-	-	-	0	?	1	0	1	1
1	1	0	0	1	3	1	0	0	0	0	1
0	1	2	0	0	1	0	0	0	0	0	0
0	0	0	0	0	0	2	0	0	-	0	0
-	0	0	0	0	1	2	1	1	1	1	1
0	1	2	1	1	?	?	?	?	?	?	?
0	?	0	0	?	0	0	0	2	0	0	1
2	-	2	0	0	0	0	1	1	1	0	0
0	-	0	0	0	0	0	0	0	0	1	0
0	0	0	0	0	0	1	0	0	?	4	4
1	3	1	0	0	1	0	0	0	-	-	-
-	1	1	0	0	3	2	?	2	0	0	0

0	2	1	0	-	2	0	0	0	2	-	1
3	2	0	0	1	0	2	2	0&1	1	1	3
3	0	1	-	0	0	0	0	0	0	1	0
1	1	?	?	1	?	0	0	0	1	0	0
1	0	0									

†*Amiopsis lepidota*

0	0	1	1	-	-	-	-	-	0	-	-
?	?	?	?	0	?	?	?	?	?	?	?
?	?	?	?	?	?	?	?	?	?	?	?
?	?	?	?	?	0	?	?	?	?	1	?
?	?	?	?	?	?	?	?	?	?	?	?
?	?	?	0	0	?	0	?	0	1	-	-
0	?	?	?	?	?	0	0	2	1	1	0
?	?	0	?	?	0	0	0	1	0	0	?
?	0	0	2	1	1	0	0	0	3	1	0
?	1	0	0	0	1	2	0	0	0	3	0
0	1	?	1	1	0	3	0	1	0	-	-
-	0	0	2	1	0	1	1	4	1	0	0
-	-	0	0	-	0	0	1	?	0	1	1
1	1	0	1	1	1	1	0	0	0	0	1
0	1	1	0	?	1	0	0	0	?	0	0
0	0	0	0	0	0	2	0	0	-	0	0
-	0	0	0	0	1	2	1	1	1	1	1
0	1	2	1	1	?	?	?	?	?	?	?
3	?	0	0	1	?	1	0	2	0	0	1
1	-	0	0	0	0	0	1	1	1	0	0
0	-	0	0	0	0	0	0	0	1	1	0
0	0	0	0	0	0	1	0&1	0	0	?	4
1	3	1	0	0	0	0	0	0	-	-	-
-	1	1	0	0	3	3	?	3	0	0	0
0	2	1	0	-	1	0	0	0	0	1	1
0	0	0	1	1	0	?	1	1	1	1	3
3	0	1	1	0	0	0	0	0	0	?	0
1	1	?	?	0	?	0	?	0	1	0	0
1	0	0									

†*Amiopsis woodwardi*

0	0	1	1	-	-	-	-	-	0	-	-
?	?	?	?	0	?	?	?	?	?	?	?
?	?	?	?	?	?	?	?	?	?	?	?
1	?	?	?	?	0	?	?	?	?	1	?
?	?	?	?	?	?	?	?	?	1	0	?
?	?	1	0	0	0	0	1	0	1	-	-
0	0	?	?	?	?	?	?	?	1	1	0
?	?	?	?	?	0	0	0	1	0	0	1
?	0	1	2	1	1	0	0	?	?	1	0
?	?	0	0	0	?	1	0	0	0	3	1
0	1	1	1	1	0	?	?	1	0	-	-
-	0	0	2	1	0	?	?	?	1	?	?
?	?	?	?	?	?	0	1	?	0	1	1
0	1	0	1	1	1	1	0	0	0	0	1
0	1	?	0	?	1	0	0	0	?	0	0

0	0	0	0	0	0	2	0	0	-	0	0
-	0	0	0	0	0	2	1	?	?	1	1
0	1	2	1	1	?	?	?	?	?	?	?
3	?	0	0	1	?	0	0	2	0	0	1
1	-	0	0	0	0	0	1	1	1	0	0
0	-	0	0	0	0	0	0	0	0	1	0
0	0	0	0	0	0	1	0	0&1	?	4	4
1	3	1	0	0	0	0	0	0	-	-	-
-	1	1	0	0	3	?	?	3	0	0	0
0	?	1	0	-	1	0	0	0	?	?	1
0	?	0	1	1	0	?	1	1	1	1	3
?	0	1	-	0	0	0	?	?	0	1	0
0	1	?	?	0	?	0	?	?	1	0	0
1	0	0									

†*Anaethalion angustus*

0	0	1	2	0	-	-	-	-	0	-	-
?	?	?	1	1	?	?	?	?	?	?	?
?	?	?	?	?	1	1	?	?	?	?	?
0	?	?	1	0	0	0	0	1	?	1	2
?	1	0	?	?	?	?	1	?	1	1	0
0	2	1	0	0	0	0	0	1	0	1	-
1	3	1	?	?	?	1	?	4	1	0	2
0	?	0	0	?	0	?	0	?	1	1	?
?	0	?	2	?	?	0	0	0	?	2	?
?	1	?	0	0	0	3	-	0	0	?	0
0	0	?	1	?	0	?	?	0	0	-	-
-	0	0	0	0	0	1	2	?	1	1	-
-	-	-	-	-	-	0	-	-	0	1	0
1	1	1	0	0	2	2	-	0	1	0	1
4	0	3	1	1	4	-	1	0	1	1	0
0	2	0	0	-	-	3	0	0	0	1	0
-	0	1	0	0	2	4	1	1	0	1	1
0	0	1	1	2	?	?	?	?	?	?	0
?	?	0	0	1	0	0	0	1	1	1	0
-	1	?	?	0	0	0	?	1	1	1	1
1	1	0	0	0	0	0&1	0	0	0	1	?
?	?	0	1	0	0	2	1	0	?	0	3
1	1	0	1	0	0	1	0	1	1	0	1
1	0	0	?	1	2	3	?	?	2	0	0
1	?	1	?	-	2	1	1	1	1	1	1
0	0	1	0	1	0	0	?	?	1	1	3
?	0	1	?	0	?	?	0	?	?	0	0
0	2	1	-	0	0	1	?	0	1	0	2
1	?	0									

†*Annaichthys pontegiurinensis*

0	0	1	0	-	0	0	?	?	0	0	-
?	?	?	?	0	-	-	?	?	?	?	?
?	?	?	?	?	?	?	?	?	?	?	?
1	?	?	?	?	?	?	?	?	?	0	-
?	?	?	?	?	?	?	?	?	?	?	?
?	?	?	0	0	?	0	?	2	?	?	?

?	0	0	?	?	?	?	?	?	1	?	?
?	?	?	?	?	0	0	0	-	?	-	?
0	0	-	-	-	1	?	?	0	0	2	1
1	0	0	0	?	?	?	?	?	?	?	?
?	?	1	1	1	1	2	0	0	0	-	-
-	0	0	0	0	?	?	1	2	1	0	1
?	1	0	0	-	0	0	-	-	0	1	?
2	1	1	0	0	2	2	-	0	1	?	1
0	1	3	0	?	4	-	?	?	0	?	0
?	?	?	?	-	?	2	0	0	-	0	1
1	1	?	0	0	2	1	1	1	1	0	1
?	0	2	?	?	?	?	?	?	?	?	?
?	?	?	0	?	?	0	-	0	1	0	?
-	-	?	?	?	?	?	?	?	?	?	0
?	?	-	?	?	?	?	?	?	?	?	?
?	?	?	?	-	-	?	?	?	?	?	?
?	?	?	?	?	?	?	?	?	?	?	?
?	0	?	?	?	1	?	0	?	0	0	?
0	1	0	0	1	0	0	1	1	0	0	?
?	0	?	?	?	0	?	?	?	?	?	?
?	?	?	2	?	?	?	0	?	?	?	?
1	?	?	?	?	?	?	?	?	?	?	?
?	?	1									

†*Araripelepidotes temnurus*

2	0	1	0	-	1	0	1	2	0	0	-
?	?	?	?	?	?	?	0	1	1	?	2
1	0	-	2	?	1	0	?	?	?	?	1
1	0	?	?	0	0	0	0	0	0	?	?
?	3	?	?	?	?	?	?	?	?	?	?
?	2	0	0	0	?	?	?	0	?	?	?
?	0	0	?	?	?	?	?	2	?	?	?
?	?	0	0	0	1	0	0	4	0	0	?
0	0	0	1	0	0	0	0	0	5	1	0
?	1	0	?	0	1	2	0	1	3	4	0
0	0	2	1	1	1	0	0	0	1	0	?
0	0	?	?	?	?	?	?	?	?	0	2
0	0	0	0	0	0	?	?	?	0	1	2
2	1	0	0	0	1	0	-	-	0	0	0
-	?	?	?	?	?	?	?	?	?	?	1
1	-	-	-	?	?	-	0	-	-	-	0
-	0	0	0	0	0	4	1	1	2	1	1
0	0	?	0	-	?	?	?	?	1	2	1
3	0	?	0	?	0	0	-	0	0	0	?
-	-	?	?	?	?	?	?	?	?	?	?
?	?	-	?	?	?	?	?	?	?	?	?
?	?	?	?	-	-	?	-	?	?	?	?
?	?	?	?	0	?	?	?	?	?	?	?
?	?	1	0	?	3	2	2	?	1	0	0
0	0	0	0	2	0	0	-	-	0	0	?
0	?	?	?	?	0	?	?	?	0	1	2
1	2	0	2	1	1	0	0	1	?	?	?

0	?	?	?	?	?	0	?	?	0	0	?
0	0	0									
†*Archaeosemionotus connectens*											
0	0	0	0	-	1	0	1	0	1	0	-
?	?	?	?	?	?	?	?	?	?	?	?
?	?	?	?	?	?	?	?	?	?	?	?
?	?	?	?	?	?	?	?	?	?	1	0
?	?	?	?	?	?	?	?	?	?	?	?
?	?	?	0	0	?	?	?	0	1	-	-
0	0	?	?	?	?	?	?	?	?	?	?
?	?	?	?	?	?	?	?	?	?	0	?
0	?	?	?	0	0	0	?	?	?	?	?
?	?	?	0	0	?	?	0	0	0	?	?
?	?	0	?	8	0	3	0	1	0	-	-
-	0	?	?	?	?	?	?	?	?	0	2
0	0	0	0	0	0	?	0	0	0	1	1
1	1	0	0	0	2	1	0	0	0	0	1
0	1	?	?	?	0	-	?	0	0	1	0
0	0	0	0	0	?	2	0	0	-	0	0
-	0	0	0	0	1	1	1	1	1	0	1
0	?	?	1	0	?	?	?	?	?	?	?
?	?	?	?	?	?	?	?	?	?	?	?
?	?	?	?	?	?	?	?	?	?	?	?
?	?	?	?	?	?	?	?	?	?	?	?
?	?	?	?	?	?	?	?	?	?	?	?
?	?	?	?	?	?	?	?	?	?	?	?
?	0	?	?	?	1	?	0	?	0	?	?
?	2	0	0	1	0	?	-	-	0	0	?
0	?	?	?	?	?	?	?	?	?	1	0
0	1	0	0	?	?	?	?	?	?	?	?
1	?	?	?	?	?	?	?	?	?	0	?
0	0	?									
†*Aspidorhynchus acutirostris*											
4	1	1	0	-	0	0	0	-	1	0	-
?	?	?	?	0	0	-	?	?	?	1	1
?	?	?	?	?	1	0	?	0	?	?	?
0	?	?	1	0	0	0	0	1	?	?	?
?	1	0	0	?	?	-	1	1	1	1	0
0	0	1	0	0	0	0	0	0	0	1	-
0	0	0	?	?	?	0	0	?	1	0	2
1	0	1	0	2	0	0	0	-	-	-	?
?	0	-	-	-	-	-	0	0	3	2	0
3	?	0	1	0	1	3	-	0	0	2	0
0	0	1	1	6	0	2	0	0	0	-	-
-	0	0	4	0	0	0	1	?	1	0	1
0	0	1	0	-	0	0	?	?	0	1	1
1	2	2	0	0	1	1	1	1	1	0	1
0	1	1	0	0	3	-	0	0	0	0	0
0	2	?	0	0	0	3	0	0	0	0	0
-	0	0	0	0	0	4	1	1	1	1	1
0	0	3	0	-	?	?	?	?	?	?	?

0	0	0	?	?	?	0	0	1	1	0	?
?	-	?	?	?	0	?	?	?	?	?	0
?	?	?	0	0	0	0	0	0	0	0	?
?	?	1	-	?	0	1	0	0	0	4	4
0	-	0	2	0	0	0	0	1	2	1	2&3
0	0	0	?	1	3	0	0	?	0	0	0
1	2	1	0	-	2	0	1	?	0	0	1
0	0	?	?	0	0	0	?	?	0	1	0
0	0	0	0	?	?	?	0	?	1	2	?
1	?	?	?	0	0	0	?	?	0	0	2
0	3	0									

Atractosteus spatula

4	0	0	0	-	1	1	1	1	0	0	-
0	-	-	0	0	-	-	0	1	0	?	2
1	0	-	1	0	0	-	1	1	-	-	1
1	0	6	1	1	-	1	0	0	1	1	0
2	2	1	1	-	-	1	1	1	1	0	1
-	4	0	1	1	0	1	3	4	0	0	1
-	0	0	0	1	1	0	0	1	1	0	3
3	0	0	0	2	2	0	0	4	0	0	0
1	0	0	0	1	1	0	1	0	4	1	0
0	1	1	1	0	1	2	0	1	1	4	1
1	0	2	0	3	1	0	0	0	1	3	0
1	1	0	2	1	0	0	1	0	1	0	3
0	2	0	0	0	0	0	1	2	1	-	-
-	-	-	-	-	-	0	-	-	0	0	1
2	1	1	0	0	1	1	0	1	1	0	1
0	1	0	0	0	4	3	0	0	1	0	0
-	1	0	0	0	0	0	1	0	3	0	0
-	0	4	0	-	0	1	1	1	1	2	1
0	0	1	-	?	1	0	1	2	0	0	0
0	-	2	0	?	1	0	0	1	1	0	0
0	-	1	0	0	0	0	0	0	0	0	0
0	0	0	0	0	0	1	0	0	0	0	0
1	0	0	0	0	0	0	0	0	-	-	-
-	1	1	0	0	3	2	3	2	1	0	0
0	0	0	0	2	0	0	-	?	0	0	1
0	2	0	0	0	0	2	1	0	0	1	1
1	0	0	2	2	0	0	0	1	1	2	1
0	6	0	1	0	0	0	0	0	0	0	0
0	0	0									

Brycon meeki

0	0	1	2	0	-	-	-	-	0	-	-
1	2	2	1	1	1	1	?	0	-	3	2
1	0	-	0	1	1	1	2	0	1	0	1
0	0	3	1	0	0	1	0	1	0	?	?
2	1	?	0	0	1	?	0	-	1	1	0
1	5	0	0	0	?	0	0	2	0	1	-
0	4	1	1	0	-	1	?	3	1	0	2
0	0	0	0	2	0	1	0	3	0	0	?
1	1	0	2	1	0	0	0	1	2	2	?

?	1	?	0	0	1	3	-	0	0	0	0
0	0	1	1	0	1	2	0	0	0	-	-
-	0	0	4	0	1	1	4	-	1	1	-
-	-	-	-	-	-	0	-	-	0	1	-
2	1	0	0	0	1	0	-	-	1	0	1
4	0	3	0	1	1	0	1	0	1	2	0
0	0	0	-	-	?	3	0	0	0	0	?
?	1	?	0	0	1	4	0	-	-	1	1
0	0	4	0	-	0	0	0	0	1	0	0
3	2	0	0	0	0	0	0	?	?	1	0
-	1	0	0	0	1	1	?	1	1	?	1
1	1	1	0	0	0	0	0	0	0	0	1
1	1	0	1	1	1	?	2	1	0	3	4
1	1	0	1	0	2	0	0	1	2	1	2
3	0	0	1	0	2	3	?	4	2	0	0
0	0	1	0	-	2	0	1	?	2	-	1
0	0	0	1	1	0	0	1	0	1	1	3
3	0	1	-	2	0	-	0	0	1	?	0
0	3	1	-	0	0	1	1	?	1	0	2
1	?	?									

†*Calamopleurus cylindricus*

2	0	0	1	-	-	-	-	-	0	-	-
?	?	?	?	0	?	?	1	1	0	2	2
1	1	0	0	0	1	?	1	0	1	1	1
1	1	0	1	1	0	0	0	0	0	1	0
1	3	0	0	1	0	-	1	1	1	0	0
0	0	1	0	0	0	0	1	0	1	-	-
0	3	0	?	?	?	0	0	2	1	1	0
2	0	1	0	2	0	0	0	1	0	0	1
2	0	0	0	1	1	1	0	0	2	2	0
3	1	0	0	0	1	0	1	0	0	3	0
0	0	4	0	2	0	2	0	0	0	-	-
-	0	0	2	1	0	0	1	4	1	1	-
-	-	-	-	-	-	0	1	1	0	1	1
0	1	3	1	1	1	1	0	0	1	0	1
0	1	2	0	0	1	0	0	0	0	0	0
0	0	0	4	0	0	2	0	0	-	0	0
-	0	0	0	0	1	2	1	?	1	1	1
0	1	1	1	0	?	?	?	?	?	?	?
?	?	?	0	?	0	1	0	2	0	0	1
2	-	0	0	0	0	0	0	1	1	?	0
0	-	0	0	0	0	0	0	0	1	1	0
0	0	0	0	0	0	0	0	1	?	3	3
1	3	1	0	0	0	0	0	0	-	-	-
-	1	1	0	0	3	2	?	3	1	0	0
0	1	1	0	-	?	0	2	2	2	-	1
0	?	0	1	1	0	2	?	?	?	1	3
3	0	1	-	0	0	1	0	0	1	1	0
0	1	?	?	0	?	0	0	?	1	0	0
1	0	0									

†*Callipurbeckia minor*

0	0	0	0	-	2	0	1	2	0	1	1
?	?	?	?	0	?	?	?	?	?	?	2
?	?	?	?	?	?	?	?	?	?	?	?
?	?	?	?	?	?	0	?	?	?	1	0
1	?	1	0	0	?	0	?	?	?	?	?
?	1	0	0	0	?	0	0	3	0	0	1
-	0	0	?	?	?	?	?	?	?	0	4
?	?	?	?	?	0	0	0	4	0	0	?
?	0	0	2	1	1	0	1	0	5	1	0
3	1	1	0	0	1	1	0	0	0	3	0
0	0	0	1	7	0	0	0	0	1	2	2
0	0	0	3	1	0	0	1	0	1	0	1
0	0	1	0	-	0	0	1	1	0	1	1
4	3	2	0	0	1	1	0	0	1	0	1
0	1	?	0	?	1	1	?	0	?	0	0
0	0	0	1	?	?	2	0	1	-	0	0
-	0	0	0	0	0	1	1	1	3	1	1
0	?	?	0	-	?	?	?	?	1	1	0
0	0	?	0	?	?	0	?	?	?	?	?
?	?	?	0	?	?	?	?	?	?	?	?
?	?	?	?	?	?	?	?	?	?	?	?
?	?	?	?	?	?	?	?	?	?	?	?
?	?	?	?	?	?	?	?	?	?	?	?
?	?	?	0	0	3	?	1	?	1	0	0
0	1	0	0	1	0	0	-	-	0	0	1
0	0	0	0	0	0	?	?	1	0	1	2
?	2	0	2	1	1	0	0	1	0	2	?
1	4	?	?	?	?	0	?	0	0	0	?
0	0	0									

†*Camerichthys lunae*

0	0	?	0	-	1	?	?	?	?	1	0
?	?	?	?	?	?	?	?	?	?	?	?
?	?	?	?	?	?	?	?	?	?	?	?
?	?	?	?	?	?	?	?	?	?	?	?
?	?	?	?	?	?	?	?	?	?	?	?
?	?	?	0	0	?	?	?	1	?	?	?
?	?	?	?	?	?	?	?	?	?	?	?
?	?	?	?	?	?	?	?	?	?	?	?
?	?	?	?	?	1	0	1	?	?	?	?
?	1	?	0	0	1	2	0	0	1	?	0
?	?	2	1	7	0	0	0	0	1	3	2
1	0	0	3	1	0	0	?	?	?	0	3
1	0	0	0	1	0	?	?	?	0	1	1
4	3	0	?	0	1	?	?	?	1	0	1
0	1	?	?	?	1	?	?	0	?	?	?
0	0	0	1	2	?	2	0	0	-	0	0
-	0	-	0	1	0	?	1	1	1	0	1
0	0	?	0	-	?	?	?	?	?	?	?
?	?	?	0	?	?	?	?	?	?	?	?
?	?	?	?	?	?	?	?	?	?	?	?
?	?	?	?	?	?	?	?	?	?	?	?

?	?	?	?	?	?	?	?	?	?	?	?
?	?	?	?	?	?	?	?	?	?	?	?
?	?	?	?	?	?	?	?	?	?	?	?
?	?	?	?	?	?	?	?	?	?	?	?
?	?	?	?	?	?	?	?	?	?	?	?
?	?	?	?	?	?	?	?	?	?	?	?
?	?	?	?	?	?	?	?	?	?	?	?
?	?	?									

†*Catervariolus hornemani*

0	0	0	0	-	0	0	0	-	0	0	-
1	2	4	?	1	?	-	?	0	-	?	2
0	?	?	0	?	?	?	1	0	?	?	0
0	0	3	1	0	?	0	0	0	0	1	1
?	1	1	0	0	0	0	1	1	1	1	?
0	0	0	0	0	0	0	?	1	0	1	-
0	3	?	?	?	?	0	?	0	1	?	?
0	0	0	0	?	0	0	0	4	0	0	?
0	0	0	3	0	0	0	0	0	0	0&1	0
?	1	0	0	0	1	1	0	0	0	3	0
0	0	-	3	0	0	2	0	0	0	-	-
-	0	0	?	0	0	1	1	2	1	0	2
0	0	0	0	0	0	1	0	-	0	1	1
?	1	2	0	0	1	1	1	0	1	?	1
0	1	1	0	0	1	0	0	0	1	0	0
0	0	0	0	0	0	2	0	0	-	0	0
-	0	0	0	0	0	4	1	1	1	1	1
?	?	3	1	0	?	?	?	1	0	-	0
0	0	1	0	?	0	0	-	0	1	0	1
-	-	0	?	?	-	1	?	1	1	?	1
0	-	?	0	0	0	0	0	0	0	0	0
0	0	0	0	-	-	1	0	0	?	4	4
1	0	0	0	0	0	0	0	1	1	1	3
1	0	0	0	?	2	2	?	?	0	0	0
0	2	0	0	0	0	0	1	1	0	0	1
0	0	?	?	0	0	0	?	1	0	1	0
?	?	0	0	0	0	0	0	0	0	1	0
?	2	?	?	0	0	1	?	?	0	0	?
0	0	?									

†*Caturus furcatus*

2	0	1	1	-	-	-	-	-	0	-	-
?	?	?	?	0	?	?	?	?	?	1	1
1	?	?	0	0	1	0	?	0	1	2	?
1	-	3	1	?	?	0	0	0	0	1	0
1	3	0	0	2	0	-	1	1	1	0	0
0	?	?	0	0	0	0	1	0	1	-	-
0	0	0	?	?	?	0	?	2	?	1	0
2	0	1	0	1	0	0	0	1	?	2	1
2	0	1	2	0	0	1	0	0	3	1	0
2	1	0	0	0	1	0	1	0	0	3	1
0	1	4	0	1	0	2	0	0	0	-	-
-	0	0	2	1	0	1	1	4	1	0	1

0	0	1	0	-	0	0	0	0	0	1	1
1	2	3	1	1	1	1	0	0	1	0	1
0	1	1	0	0	1	0	0	0	0	0	0
0	0	0	4	0	0	2	0	0	-	0	0
-	0	0	0	0	0	4	1	2	0	1	1
1	1	0	1	0	?	?	?	?	?	?	1
3	0	?	0	0	0	0	-	1	0	0	1
-	-	0	0	0	0	0	?	1	0	0	0
0	-	-	0	0	1	0	1	1	0	0	0
0	0	0	0	1	-	?	0	0	?	?	4
1	1	1	0	0	-	0	0	0	-	-	-
-	0	0	0	1	1	1	0	4	0	0	0
1	3	1	0	-	1	?	1	1	0	0	1
0	0	?	?	0	0	0	?	?	0	1	0
?	1	0	0	0	0	0	1	0	1	1	0
1	?	0	1	0	?	?	?	1	0	0	0
?	3	0									

†*Cratoamia gondwanica*

3	0	0	1	-	-	-	-	-	0	-	-
?	?	?	?	?	?	?	?	?	?	?	?
?	?	?	?	?	?	?	1	0	?	?	1
1	?	?	?	?	?	?	?	?	?	1	0
?	1	?	?	?	?	?	?	?	1	0	?
?	?	?	0	0	?	?	?	?	?	?	?
?	?	?	?	?	?	?	?	?	1	1	0
?	?	?	?	?	0	0	0	1	0	0	?
?	0	0	0	0	0	1	0	?	?	1	0
?	?	1	0	0	0	?	?	0	?	?	0
0	1	2	0	1	0	2	0	0	0	-	-
-	0	0	0	0	0	1	1	?	1	1	-
-	-	-	-	-	-	0	1	?	0	1	0
0	2	0	1	1	1	1	0	0	?	0	?
?	1	2	0	?	1	0	0	0	0	0	0
0	0	0	4	0	0	2	0	0	-	0	0
-	0	0	0	0	0	2	?	?	?	?	1
?	?	1	1	?	?	1	?	?	?	?	?
?	?	?	0	?	0	?	0	2	0	0	1
2	-	?	0	0	0	?	?	?	?	?	?
?	?	0	0	0	0	0	0	0	1	1	?
?	?	0	0	0	0	0	0	1	?	2	2
1	3	1	0	0	0	0	0	0	-	-	-
-	1	1	0	0	3	?	?	3	0	0	0
0	2	1	0	-	1	0	2	?	2	-	1
1	?	?	?	?	0	?	?	?	?	?	?
?	0	?	-	?	?	1	0	?	?	?	0
?	?	1	-	0	?	?	?	?	?	?	?
?	?	?									

†*Cuneatus cuneatus*

4	0	0	0	-	0	0	?	?	0	0	-
?	?	?	?	0	?	?	0	?	?	?	2
1	?	?	1	0	?	?	1	1	-	-	1

1	?	?	?	?	?	1	?	?	1	1	0
2	2	1	1	-	-	1	0	-	1	0	1
-	4	0	1	1	?	1	3	3	0	0	1
-	?	0	?	1	1	0	0	1	?	0	3
?	?	?	?	?	1	0	0	4	0	0	?
?	0	0	3	0	1	0	1	0	4	1	0
0	1	1	1	0	1	2	0	1	1	4	0
?	0	2	0	3	1	2	0	0	1	1	0
1	1	0	?	?	?	0	1	?	1	0	3
0	2	0	0	0	0	0	1	2	1	-	-
-	-	-	-	-	-	?	?	?	0	0	1
2	1	?	0	0	1	1	?	?	?	2	?
0	0	0	0	0	?	3	0	0	1	0	0
-	1	?	0	0	1	0	1	?	2	0	0
-	0	4	0	-	?	?	?	?	?	?	?
?	?	?	-	?	1	0	1	2	0	0	0
?	-	?	?	?	1	?	?	?	?	?	?
?	?	?	?	?	?	?	?	?	?	?	?
?	?	?	?	?	?	?	?	?	?	?	?
?	?	?	?	?	?	?	?	?	?	?	?
?	1	?	?	?	3	2	3	?	1	0	0
0	0	0	0	2	0	0	-	?	0	0	?
0	?	?	?	0	0	?	?	?	?	1	1
1	1	0	2	2	?	0	0	?	?	?	?
?	?	1	-	0	0	?	?	0	0	0	?
0	0	0									

†*Cuneatus wileyi*

4	0	0	0	-	0	?	?	?	0	0	-
?	?	?	?	0	?	?	0	?	?	?	2
1	?	?	1	0	?	?	1	1	-	-	1
?	?	?	?	1	?	?	?	?	1	1	0
2	2	1	1	-	-	1	0	-	1	0	1
-	?	0	1	1	?	?	?	3	0	0	1
-	?	0	?	1	1	0	0	1	?	0	3
?	?	?	?	?	1	0	0	4	0	0	?
?	0	0	3	0	1	0	1	0	4	1	0
?	1	1	1	0	1	2	0	1	1	4	0
?	0	2	0	3	1	2	0	0	1	1	0
1	1	0	?	?	?	0	1	0	1	0	3
0	2	0	0	0	0	0	1	2	1	-	-
-	-	-	-	-	-	?	?	?	0	0	1
2	1	1	0	0	1	1	?	1	?	?	?
?	?	?	?	?	?	3	0	0	1	0	0
-	1	?	?	?	?	?	?	?	2	0	0
-	0	4	0	-	?	?	?	?	?	?	?
?	?	?	-	?	1	0	1	2	0	0	0
?	-	?	?	?	1	?	?	?	?	?	?
?	?	?	?	?	?	?	?	?	?	?	?
?	?	?	?	?	?	?	?	?	?	?	?
?	?	?	?	?	?	?	?	?	?	?	?
?	1	?	?	?	3	2	?	?	1	0	0

0	0	0	0	?	0	0	-	?	0	?	?
0	?	?	?	0	0	?	?	?	?	1	1
1	0	0	2	2	?	0	0	?	0	2	1
?	?	1	-	0	0	?	?	0	0	0	?
0	0	0									

†*Cyclurus gurleyi*

3	0	0	1	-	-	-	-	-	0	-	-
?	?	?	?	0	?	?	?	?	?	?	?
1	1	0	?	0	?	?	1	0	1	1	1
1	1	?	?	0	0	?	?	?	0	1	0
1	2	0	0	0	?	-	1	2	1	0	?
0	0	1	0	0	0	0	?	0	1	-	-
0	?	?	?	?	?	?	?	2	1	1	0
2	?	?	?	?	0	0	0	?	1	0	1
0	0	1	1	0	0	1	0	0	0	0	0
2	1	1	0	1	-	-	-	-	-	1	0
0	1	3	0	2	0	2	0	1	0	-	-
-	0	0	2	1	0	0	1	4	1	1	-
-	-	-	-	-	-	0	1	?	0	1	1
1	1	0	0	1	3	1	0	0	0	0	1
0	1	?	0	0	1	0	0	0	0	0	0
0	0	0	0	2	2	2	0	0	-	0	0
-	0	0	0	0	0	2	1	1	1	1	1
0	1	2	1	1	?	?	?	?	?	?	?
0	?	0	0	?	?	0	0	2	0	0	1
2	-	2	0	0	0	0	1	1	1	0	0
0	-	0	0	0	0	0	0	0	0	0	0
0	0	0	0	0	0	1	0	0	?	3	3
1	3	1	0	0	1	0	0	0	-	-	-
-	1	1	0	0	3	2	?	3	0	0	0
0	2	1	0	-	2	0	2	?	2	-	1
3	2	0	1	1	0	2	2	1	1	1	3
3	0	1	-	0	0	0	0	0	1	1	0
1	1	?	?	1	?	0	?	?	?	0	0
?	0	0									

†*Cyclurus kehreri*

3	0	0	1	-	-	-	-	-	0	-	-
?	?	?	?	0	?	?	?	?	?	2	1
1	?	?	0	0	?	?	2	0	1	1	1
1	1	0	?	1	0	?	1	?	0	1	0
1	2	0	0	0	0	-	1	2	1	0	0
0	0	1	0	0	0	0	1	0	1	-	-
0	1	0	?	?	?	0	0	2	1	1	0
2	0	0	0	2	0	0	0	1	1	0	1
0	0	1	0	0	0	1	0	0	0	0	0
2	1	1	0	1	-	-	-	-	-	1	0
0	1	3	0	2	0	2	0	1	0	-	-
-	0	0	2	1	0	0	1	4	1	1	-
-	-	-	-	-	-	0	1	?	0	1	1
1	1	0	0	1	3	1	0	0	0	0	1
0	1	2	0	0	1	0	0	0	0	2	0

0	0	0	0	2	2	2	0	0	-	0	0
-	0	0	0	0	0	2	1	1	1	1	1
0	1	2	1	0	?	?	?	?	?	?	1
0	?	0	0	1	0	0	0	2	0	0	1
2	-	2	0	0	0	0	1	1	?	0	0
0	-	0	0	0	0	0	0	0	0	1	0
0	0	0	0	0	0	1	0	0	?	3	4
1	3	1	0	0	1	0	0	0	-	-	-
-	1	1	0	0	3	2	?	2	0	0	0
0	2	1	0	-	2	0	2	?	2	-	1
2	2	0	1	1	0	2	2	0	1	1	3
3	0	1	?	0	0	0	0	0	1	1	0
1	1	?	?	1	?	0	?	0	1	0	0
1	0	0									

†*Dapedium caelatum*

5	0	0	0	-	0	0	0	-	0	0	-
?	?	?	?	0	?	?	?	?	?	?	?
-	-	-	-	?	?	?	?	?	?	?	-
-	-	-	1	?	?	?	?	-	0	?	?
?	2	?	?	?	?	?	0	-	1	0	?
?	?	?	0	0	?	?	?	2	?	?	?
?	?	?	?	?	?	?	?	?	?	0	4
?	?	?	?	?	2	0	0	3	0	0	?
?	0	?	0	0	0	0	0	?	0	0	0
?	0	?	0	0	0	3	-	1	0	?	?
?	?	?	?	?	?	0	0	0	?	?	?
-	?	0	1	0	?	?	1	1	1	0	3
?	0	0	0	?	?	0	?	?	0	1	1
2	1	0	0	0	1	?	?	?	1	0	?
?	1	1	0	0	0	-	0	0	0	0	1
0	0	1	2	0	?	3	0	0	?	0	?
?	1	?	0	0	0	0	?	?	?	0	1
0	0	?	1	0	?	?	?	?	?	?	?
0	?	0	0	?	?	0	?	?	?	?	?
?	?	?	?	?	?	?	?	?	?	?	?
?	?	?	?	?	?	?	?	?	?	?	?
?	?	?	?	?	?	?	?	?	?	?	?
?	?	?	?	?	?	?	?	?	?	?	?
?	1	?	?	?	1	?	0	?	?	?	?
0	1	0	0	?	0	0	-	?	0	0	?
1	?	?	?	?	1	2	?	?	?	1	?
?	?	0	?	1	1	0	?	1	?	?	?
?	?	?	?	?	?	?	?	?	?	0	?
0	0	0									

†*Dapedium pholidotum*

5	0	0	0	-	0	0	0	-	0	0	-
?	?	?	?	0	?	?	?	?	?	?	?
?	?	?	?	?	?	?	?	?	?	?	?
?	?	?	?	?	?	?	?	?	?	?	?
?	?	?	?	?	?	?	?	?	?	?	?
?	?	?	0	0	?	?	?	2	?	?	?

?	?	?	?	?	?	?	?	?	?	0	?
?	?	?	?	?	2	0	0	-	-	-	?
?	?	?	?	?	?	?	?	?	0	0	0
0	0	?	?	0	0	3	-	?	0	4	0
?	0	?	?	1	0	2	0	?	0	-	?
-	0	0	1	0	?	?	1	1	1	0	3
1	0	0	0	0	0	0	?	?	0	1	1
2	1	2	0	0	1	?	?	?	1	0	1
0	1	1	0	?	0	-	0	0	0	0	1
0	0	1	2	?	?	3	0	0	?	0	?
?	1	?	0	0	0	0	?	?	?	0	1
0	0	3	1	0	?	?	?	?	?	?	?
?	?	0	0	?	?	0	?	?	?	?	?
?	?	?	?	?	?	?	?	?	?	?	?
?	?	?	?	?	?	?	?	?	?	?	?
?	?	?	?	?	?	?	?	?	?	?	?
?	?	?	?	?	?	?	?	?	?	?	?
?	1	?	?	?	1	3	0	?	2	?	?
0	1	0	0	?	0	0	-	-	0	0	?
1	?	?	?	?	1	2	?	?	?	1	?
?	?	0	?	?	?	0	1	?	?	1	?
?	?	?	?	?	?	?	?	?	0	0	?
?	0	0									

†*Dapedium stollorum*

5	0	0	0	-	1	0	0	-	0	0	-
?	?	?	?	0	?	?	?	?	?	?	?
?	?	?	?	?	?	?	?	?	?	?	?
?	?	?	?	?	?	?	?	?	?	1	0
?	?	?	?	?	?	?	?	?	?	?	?
?	?	?	0	0	?	?	?	3	?	?	?
?	?	?	?	?	?	?	?	?	?	0	?
?	?	?	?	?	2	0	0	4	0	0	?
?	0	1	0	0	0	0	0	?	0	0	0
0	0	?	0	0	0	3	-	1	0	4	0
?	0	2	2	0	0	2	0	0	0	-	-
-	0	0	1	0	0	1	1	?	1	0	3
1	0	0	0	0	0	0	0	0	0	1	1
2	1	2	0	0	1	1	0	0	?	0	?
?	?	?	0	?	?	?	?	0	0	0	1
0	0	1	2	?	?	3	0	0	?	0	?
?	1	?	0	0	0	0	1	1	1	0	1
0	0	3	1	0	?	?	?	?	?	?	0
0	0	0	0	?	0	0	?	?	?	?	?
?	?	?	?	?	?	?	?	?	?	?	?
?	?	?	?	?	?	?	?	?	?	?	?
?	?	?	?	?	?	?	?	?	?	?	?
?	?	?	?	?	?	?	?	?	?	?	?
?	1	?	?	?	1	0	0	?	2	?	?
0	2	0	0	0	0	0	-	-	0	0	?
1	2	?	?	?	0	2	?	?	?	1	?
?	?	0	?	1	1	0	1	1	?	?	?

?	?	?	?	?	?	?	?	?	0	0	?
0	0	0									

†*Dentilepisosteus laevis*

1	1	2	0	-	1	1	1	1	0	0	-
?	?	?	?	0	?	?	0	1	0	?	2
1	0	-	2	0	0	-	1	0	0	-	1
1	0	?	1	-	1	1	?	?	1	1	0
1	2	1	1	-	-	1	1	1	1	0	?
?	3	0	1	0	?	1	0	4	0	0	1
-	0	0	?	1	1	0	0	1	?	0	3
?	0	0	0	2	2	0	0	4	0	0	1
?	0	0	0	1	1	0	1	0	?	?	?
?	1	1	0	0	1	1	0	0	1	3	1
0	0	1	1	4	1	2	0	0	1	4	?
1	0	?	?	?	?	0	1	?	1	0	?
0	?	0	0	0	0	0	1	2	0	1	2
4	1	2	0	0	1	?	?	?	0	0	1
0	1	1	0	?	1	?	?	0	0	0	0
0	0	1	0	?	0	3	0	1	1	0	0
-	0	0	0	0	1	3	1	1	2	0	1
1	0	4	0	-	?	?	?	?	?	?	?
0	?	?	0	?	?	0	1	2	0	0	0
0	-	2	?	?	1	0	?	0	?	?	0
0	?	1	0	0	0	0	0	0	0	0	0
0	0	1	0	0&1	-	?	?	?	?	1	1
1	1	0	2	0	-	0	0	0	-	-	-
-	?	1	0	?	3	2	3	3	1	0	0
?	0	0	0	2	0	0	?	?	0	0	?
0	?	?	?	?	0	?	?	?	?	1	?
?	2	0	2	2	0	0	0	0	?	2	?
?	?	?	?	0	?	?	?	?	0	0	?
0	0	0									

†*Diplomystus dentatus*

0	0	1	2	0	-	-	-	-	?	-	-
1	?	?	?	1	0	-	0	0	-	3	2
0	?	?	0	1	1	1	2	0	?	?	1
0	?	5	1	0	0	?	0	1	0	?	?
?	1	1	?	?	?	0	1	3	1	1	0
?	?	?	0	0	0	0	0	1	0	1	-
1	0	1	?	?	?	?	?	4	1	0	2
0	0	0	0	3	0	1	0	?	1	?	?
0	0	?	2	?	1	0	0	0	2	2	?
?	1	0	0	0	0	3	-	0	0	0	0
0	0	0	1	6	0	2	0	0	0	-	-
-	0	0	6	0	1	1	4	-	1	1	-
-	-	-	-	-	-	0	-	-	0	1	0
2	1	1	0	0	2	2	-	0	1	0	1
1	0	3	1	1	1	0	1	0	0	0	0
0	0	1	0	-	?	3	0	0	0	0	?
?	0	?	0	0	2	4	1	2	1	1	1
0	0	2	0	-	2	?	?	?	1	?	0&1

1	1	0	0	0	0	0	0	1	1	1	0
-	1	0	0	0	1	0	?	1	1	1	1
1	1	?	0	0	0	0	0	0	0	0	0
?	0	0	1	1	1	2	1	0	0	3	4
1	1	0	2	0	2	1	0	1	2	0	2
2	0	0	0	1	2	3	?	2	2	1	0
0	0	1	0	-	2	0	2	2	2	-	1
0	0	0	0	1	2	0	2	0	1	1	3
?	0	1	-	0	?	1	0	?	1	2	0
0	2	1	-	0	0	1	1	0	1	0	?
1	0	0									

†*Dorsetichthys bechei*

?	0	1	0	-	?	0	0	-	?	0	-
1	2	4	0	1	0	-	0	0	-	1	1
0	1	1	0	0	1	1	0	0	1	1	0
0	0	3	1	0	0	0	0	1	?	1	1
1	1	1	0	2	1	0	1	?	1	1	0
?	?	?	0	0	?	?	?	1	0	1	-
?	0	1	?	?	?	?	?	?	1	0	?
?	0	?	0	?	0	0	0	0	0	1	1
1	0	0	2	0	1	0	0	0	0	2	1
?	0	0	0	0	0	?	0	0	0	3	0
0	0	1	1	1	1	2	0	0	0	-	-
-	0	0	0	0	0	1	1	3	1	0	0
0	-	1	0	-	0	?	0	-	0	1	0
1	1	1	0	0	2	2	-	0	1	0	1
3	1	1	0	1	0	-	1	0	0	?	0
0	0	1	0	-	?	3	0	0	0	1	1
0	0	1	1	0	2	4	1	1	0	0	1
0	0	2	1	2	?	?	?	?	0	-	0
0	0	0	0	2	0	?	-	0	1	0	1
-	-	0	0	0	-	0	1	1	?	?	1
0	-	-	0	0	0	1	1	1	0	1	0
0	0	1	-	-	-	0	0	0	0	0	0
1	3	0	0	0	0	0	0	1	1	?	3
3	0	0	0	1	1	2	0	3	0	1	1
1	2	0	0	?	0	0	1	1	0	0	1
0	?	?	?	?	0	?	?	?	?	1	0
?	?	0	2	0	0	0	0	?	?	?	?
?	?	1	-	0	0	?	?	0	0	0	?
0	0	0									

†*Ebertichthys ettlingensis*

0	0	1	2	?	-	-	-	-	?	-	-
?	?	?	?	1	0	-	?	?	?	?	?
?	?	?	?	?	?	?	?	?	?	?	?
0	?	4	1	0	0	?	0	1	0	1	2
?	?	?	0	0	?	?	1	1	1	1	0
0	1	1	0	0	0	0	0	1	0	1	-
1	0	1	?	?	?	1	?	4	1	0	2
0	?	0	0	?	0	0	0	?	?	?	?
0	0	0	?	1	1	0	0	0	2	2	1

?	1	0	0	0	0	3	-	0	0	?	0
0	0	1	1	0	1	2	0	0	0	-	-
-	0	0	0	0	1	1	4	-	1	1	-
-	-	-	-	-	-	0	-	-	0	1	0
2	1	1	0	0	2	2	-	0	?	0	1
4	0	3	1	1	4	?	1	0	?	0	0
0	?	?	0	-	?	3	0	0	0	0	0
-	0	0	0	0	2	4	1	2	1	1	1
0	0	2	0	-	?	?	?	?	?	?	1
?	?	0	0	2	0	0	0	1	1	1	0
-	0	0	?	1	0	0	1	1	1	1	1
1	1	1	2	2	0	1	0	0	1	1	0
0	0	0	1	1	1	2	1	0	0	2	2
1	1	0	0	1	3	0	0	1	2	0	1
1	0	0	1	0	2	3	?	4	2	0	1
1	2	1	0	-	2	1	1	1	1	1	1
0	0	1	1	1	0	0	1	0&1	1	1	3
3	0	1	2	0	0	0	0	0	1	3	1
0	3	1	-	0	0	1	1	0	1	?	2
1	0	?									

Elops saurus

0	0	1	2	1	-	-	-	-	0	-	-
1	2	3	1	1	0	-	0	0	-	3	2
0	0	-	0	1	1	1	2	0	1	1	1
0	0	5	1	0	0	0	0	1	0	1	2
2	1	0	0	0	1	-	1	1	1	1	0
0	2	0	0	0	0	0	0	1	0	1	-
0	2	1	1	1	0	1	1	1	1	0	2
0	0	0	1	3	0	0	0	0	1	1	?
0	0	0	2	1	1	0	0	0	2	2	?
?	1	0	0	0	1	3	-	0	-	0	0
0	0	1	1	1	0	2	0	0	0	-	-
-	0	0	0	0	0	1	2	-	1	1	-
-	-	-	-	-	-	0	-	-	0	1	0
1	1	1	0	0	2	2	-	0	1	0	1
?	0	3	1	1	?	?	1	0	0	0	0
0	2	0	0	-	0	3	0	0	0	1	?
?	0	?	0	0	1&2	4	1	1	1	1	1
0	0	0	1	2	2	0	2	0	?	?	1
3	0	0	0	1	0	0	0	1	1	1	0
-	1	?	0	0	0	0	?	1	0	1	1
1	1	?	0	0	0	1	0	0	1	1	1
1	0	0	1	0	0	2	1	0	1	3	4
1	1	0	1	1	0	1	0	1	2	0	1
1	0	0	1	0	2	3	?	3	2	0	1
1	2	1	0	-	2	1	1	1	2	-	1
1	0	0	1	?	0	0	1	1	?	1	3
?	?	1	-	0	0	1	0	0	0	0	0
0	5	1	-	0	0	1	1	0	1	1	2
1	0	1									

†*Eurycormus speciosus*

0	0	1	1	-	-	-	-	-	0	-	-
?	?	?	?	0	-	-	?	0	-	?	?
?	?	?	?	?	?	?	?	?	?	?	?
?	?	?	?	0	0	?	0	1	0	1	1
?	1	1	0	2	1	?	0	?	1	1	?
?	0	?	0	0	?	0	0	2	0	1	-
0	0	0	?	?	?	0	?	0	1	0	?
0	0	0	0	2	0	?	0	?	?	0	?
?	?	?	2	0	0	?	0	0	?	?	?
?	1	0	0	0	?	?	0	0	0	?	0
0	0	1	1	1	1	2	0	0	0	-	-
-	0	0	?	?	0	0	1	2	1	0	2
0	1	1	0	-	0	1	-	-	0	1	0
1	1	1	0	0	2	2	-	0	1	1	1
0	1	?	0	0	?	?	?	0	0	0	0
0	0	1	0	-	?	3	0	0	0	1	1
0	1	1	0	0	0	4	1	1	0	1	1
?	0	?	1	2	?	?	?	?	1	1	?
0	0	0	0	2	0	?	-	0	1	0	1
-	-	0	0	1	-	0	1	1	0	1	1
0	-	-	0	0	0	1	0	0	0	0	0
0	0	1	-	-	-	1	0	0&1	0	?	0
1	?	0	0	0	0	0	0	1	0	1	0&1
0	0	0	1	1	2	1	0	3	0	1	0
1	2	0	0	-	1	0	0	0	0	0	1
0	0	?	?	?	0	0	?	?	?	1	?
?	0	0	2	0	0	0	0	0	1	2	1
1	3	0	0	0	0	1	1	?	1	?	0
1	3	0									

Hiodon alosoides

1	0	1	2	0	-	-	-	-	0	-	-
?	?	?	1	1	1	1	0	1	0	3	2
0	0	-	0	1	1	1	1	0	1	0	1
0	0	3	1	0	0	0	0	1	0	1	2
2	1	0	0	0	1	-	1	0	1	1	1
-	5	1	0	0	1	0	0	1	0	1	-
0	3	1	1	1	0	1	1	3	1	0	2
0	0	1	1	2	0	3	0	?	0	?	?
0	0	1	0	0	0	0	0	0	2	2	0
3	1	0	0	1	0	-	-	-	-	0	0
0	0	2	0	2	1	2	0	0	0	-	-
-	0	1	-	-	-	-	4	-	1	1	-
-	-	-	-	-	-	0	-	-	0	1	1
1	2	2	0	0	1	0	-	-	1	0	1
1	0	1	1	1	2	-	1	0	0	0	0
0	2	0	0	-	-	3	0	0	0	1	0
-	0	0	0	0	0	4	0	-	-	1	1
0	0	3	0	-	2	0	3	1	0	-	0
3	0	-	0	1	0	0	0	1	1	1	0
-	1	-	0	1	0	1	1	1	1	1	1
1	?	0	0	0	0	0	1	1	0	0	0

0	0	0	0	1	0	2	1	0	0	3	4
1	3	0	1	0	0	1	0	1	2	1	1
1	0	0	1	0	3	0	?	3	0	0	0
1	2	1	1	-	2	0	2	2	2	-	1
0	0	0	1	1	1	0	2	1	1	1	3
3	0	1	-	0	0	0	0	0	1	0	0
0	1	1	-	0	1	1	1	0	1	1	2
1	0	1									

†*Histionotus oberndorferi*

3	0	0	0	-	1	?	?	?	0	0	-
?	?	?	?	0	?	?	?	?	?	?	?
?	?	?	?	?	?	?	?	?	?	?	?
?	?	?	?	?	0	?	0	0	1	1	0
?	?	1	?	?	?	0	?	?	?	?	?
?	1	1	0	0	?	0	0	4	0	0	1
-	0	?	?	?	?	?	?	?	?	0	?
?	?	?	?	?	0	1	1	-	-	0	?
?	0	1	2	1	3	0	1	?	?	?	?
?	1	1	0	0	0	0	0	0	0	?	0
0	0	0	?	?	0	4	-	?	1	?	?
0	0	0	5	1	0	0	1	?	1	1	-
-	-	-	-	-	-	0	1	?	0	1	1
4	1	2	0	0	?	0	-	-	0	0	1
?	1	?	?	?	1	?	?	0	?	0	0
0	0	0	0	?	?	2	0	0	-	0	?
?	0	?	1	0	0	?	1	1	1	1	1
1	?	?	?	?	?	?	?	?	1	1	1
3	1	0	0	?	0	?	?	?	?	?	?
?	?	?	?	?	?	?	?	?	?	?	?
?	?	?	?	?	?	?	?	?	?	?	?
?	?	?	?	?	?	?	?	?	?	?	?
?	?	?	?	?	?	?	?	?	?	?	?
?	0	?	?	?	3	2	1	?	?	?	?
0	0	0	0	0	0	?	?	?	0	0	?
1	3	?	?	0	0	?	?	?	?	1	0
0	?	0	2	1	1	0	?	?	?	?	?
?	?	?	?	?	?	?	?	?	?	?	?
0	?	?									

†*Ichthyokentema purbeckensis*

0	0	1	0	-	0	0	0	-	0	0	-
1	2	4	1	1	0	-	0	0	-	?	1
0	1	1	0	1	1	0	2	0	1	0	0
0	0	3	1	0	0	0	0	1	0	1	1
3	1	1	0	0	1	0	1	1	1	1	0
?	2	0	0	0	0	0	2	2	0	1	-
?	1	?	?	?	?	?	?	0	1	0	0
0	0	0	0	1	0	0	0	3	0	0	0
0	0	0	1	0	2	0	0	0	0	0	0
0	1	0	0	0	1	1	0	0	0	3	0
0	0	2	0	1	1	2	0	0	0	-	-
-	0	0	0	0	0	1	1	?	1	0	0

0	-	0	0	-	0	1	0	-	0	1	1
2	1	1	0	0	2	1	0	0	1	1	1
4	0	0	0	1	0	-	0	0	0	0	0
0	2	1	0	0	0	2	0	0	-	0	0
-	0	0	0	0	0	4	1	1	2	1	1
0	0	?	1	?	?	?	?	?	0	-	0
0	0	0	0	1	0	0	-	0	1	0	?
-	-	0	?	0	-	1	?	?	?	?	0
0	-	?	?	?	?	?	?	?	?	?	?
?	?	?	?	-	-	?	?	?	?	?	?
?	?	?	?	?	?	?	?	?	?	?	?
?	0	?	?	?	2	3	?	?	?	?	?
?	?	0	0	?	0	0	0	0	0	0	1
0	0	?	?	0	0	0	?	?	?	1	?
?	2	0	2	0	0	0	0	0	0	2	0
?	2	1	-	0	0	?	?	0	0	?	?
0	0	0									

†*Ionoscopus cyprinoides*

0	0	0	1	-	-	-	-	-	0	-	-
?	?	?	?	0	-	-	1	1	0	4	1
1	1	0	0	?	1	0	?	0	1	2	0
0	0	0	1	1	0	0	?	?	0	1	0
?	3	0	0	2	0	-	?	?	1	0	?
?	0	?	0	0	0	?	?	0	1	-	-
0	0	0	?	?	?	0	0	2	1	1	0
2	0	0	0	2	0	0	0	1	1	2	?
1	0	-	2	?	2	0	0	0	3	1	0
?	1	1	0	0	0	1	0	0	0	3	0
0	1	0	1	8	0	3	1	1	0	-	-
-	0	0	3	1	0	0	1	?	1	0	2
0	0	1	0	0	0	0	0	0	0	1	1
0	1	0	1	1	1	1	0	0	0	0	1
0	1	1	0	0	1	0	0	0	0	0	0
0	0	0	0	2	0	2	0	0	-	0	0
-	0	0	0	0	0	4	1	1	1	1	1
0	1	2	1	?	?	?	?	?	?	?	1
3	?	?	1	?	0	0	0	2	0	0	0
0	-	1	0	0	0	0	1	1	0	0	0
0	-	0	0	0	1	0	0	0	0	0	?
?	?	0	0	0	0	1	1	0	0	0	0
1	-	1	0	0	0	0	0	0	-	-	-
-	0	0	0	1	1	2	?	0	0	1	0
0	2	0	1	-	1	0	2	1	0	0	1
0	0	0	1	0	0	0	1	1	0	1	0
0	0	0	0	0	0	0	0	?	?	2	0
?	2	1	-	0	-	0	?	0	?	0	0
0	0	0									

†*Isanichthys lertboosi*

0	0	0	0	-	0	?	1	2	0	1	0
?	?	?	?	0	?	?	?	1	0	1	2
1	0	-	0	0	?	?	?	1	-	-	1

1	0	?	?	0	0	0	0	0	?	1	0
?	?	?	?	?	?	?	?	?	?	?	?
?	?	?	0	0	?	?	?	1	?	?	?
?	?	?	?	?	?	?	?	?	?	?	?
?	?	?	?	?	0	0	0	3	?	0	?
?	0	0	0	0	0	0	1	0	1	1	0
3	?	1	0	0	1	2	0	1	1	4	0
0	0	2	1	3	0	0	0	0	1	1	1
0	0	?	?	?	?	?	?	?	?	0	3
1	0	0	0	1	0	0	1	1	0	1	1
3	1	2	0	0	1	1	0	0	1	0	1
3	1	?	?	?	?	?	?	0	?	0	1
0	0	0	0	1	?	2	0	0	-	0	0
-	0	0	0	0	0	4	1	?	?	?	1
0	0	2	0	-	?	?	?	?	?	?	0
0	0	0	0	-	0	?	-	0	0	0	?
-	-	?	?	?	-	?	?	?	?	?	?
?	?	?	?	?	?	?	?	?	?	?	?
?	?	?	?	-	-	?	?	?	?	?	?
?	?	?	?	?	?	?	?	?	?	?	?
?	?	?	?	?	1	2	?	?	?	0	0
0	0	0	0	?	0	0	-	?	?	0	?
0	?	?	?	?	?	?	?	?	?	1	1
?	?	0	2	1	1	0	0	?	?	2	?
?	?	?	?	?	?	?	?	?	0	0	?
0	0	0									

†Isanichthys palustris

0	0	0	0	-	0	?	?	?	0	?	?
?	?	?	?	0	?	?	?	1	0	?	?
?	?	?	?	?	?	?	?	?	?	?	?
?	?	?	?	?	0	?	?	?	?	?	?
?	?	?	?	?	?	?	?	?	?	?	?
?	?	?	0	0	?	?	?	1	?	?	?
?	?	?	?	?	?	?	?	?	?	?	?
?	?	?	?	?	1	0	0	4	0	0	?
?	0	0	0	0	0	0	1	?	?	?	?
?	?	1	0	0	1	2	0	1	1	4	0
0	0	2	1	3	0	?	0	0	1	1	?
0	0	?	?	?	?	?	?	?	?	0	3
1	2	0	0	0	0	?	?	?	0	1	1
2	1	3	0	0	1	1	0	0	1	0	1
?	1	?	?	?	?	?	?	0	?	?	0
0	0	0	0	?	?	2	0	0	-	0	0
-	0	0	0	0	0	0	1	1	?	1	1
0	0	?	0	-	?	?	?	?	?	?	?
?	?	?	?	?	0	?	?	?	?	?	?
?	?	?	?	?	?	?	?	?	?	?	?
?	?	?	?	?	?	?	?	?	?	?	?
?	?	?	?	?	?	?	?	?	?	?	?
?	?	?	?	?	?	?	?	?	?	?	?
?	?	?	?	?	?	?	?	?	?	?	?

?	?	?	?	?	?	?	?	?	?	?	?
?	?	?	?	?	?	?	?	?	?	?	?
?	?	?	?	?	?	?	?	?	?	?	?
?	?	?	?	?	?	?	?	?	?	?	?
?	?	?									

†*Knerichthys bronni*

?	?	1	0	-	1	0	0	-	?	?	?
?	?	?	?	0	-	-	?	?	?	?	?
?	?	?	?	?	?	?	?	?	?	?	?
1	?	?	?	?	0	?	?	?	?	?	?
?	?	?	?	?	?	?	?	?	?	?	?
?	?	?	0	0	?	?	?	1	?	?	?
?	?	?	?	?	?	?	?	?	1	?	?
0	?	?	?	?	0	?	?	?	?	?	?
?	0	?	?	?	1	?	?	0	0	2	0
?	?	0	0	?	?	?	?	?	?	?	0
0	0	1	1	1	1	?	?	0	0	-	-
-	0	?	?	?	?	?	1	?	?	0	3
0	1	1	0	?	0	?	?	?	0	1	0
0	1	?	0	0	2	1	0	0	?	1	?
?	?	?	0	?	?	?	?	?	?	?	0
?	?	?	?	?	?	2	0	0	-	0	?
?	?	1	0	0	2	0	1	2	?	0	1
?	0	?	?	?	?	?	?	?	?	?	?
0	?	?	?	?	0	0	?	?	?	?	?
?	?	?	?	?	?	?	?	?	?	?	?
?	?	-	?	?	?	?	?	?	?	?	?
?	?	?	?	-	-	?	?	?	?	?	?
?	?	?	?	?	?	?	?	?	?	?	?
?	0	?	?	?	1	?	0	?	0	0	?
0	2	0	0	?	0	0	1	1	0	0	?
?	?	?	?	?	?	?	?	?	?	?	?
?	?	?	1	0	?	1	?	?	?	?	?
?	?	?	?	?	?	?	?	0	?	?	?
?	?	?									

†*Kyphosichthys grandei*

0	0	0	0	-	1	0	1	1	0	1	0
?	?	?	?	0	?	?	?	?	?	?	?
?	?	?	?	?	?	?	?	?	?	?	?
?	?	?	?	0	0	?	?	?	?	?	?
?	?	0	0	2	?	-	?	?	?	?	?
?	?	?	0	0	?	?	?	1	0	0	1
-	?	?	?	?	?	?	?	?	?	0	?
?	?	?	?	?	0	0	1	4	0	0	?
?	0	0	0	0	0	0	0	?	5	?	0
?	1	0	1	0	0	1	0	0	0	3	0
?	0	0	1	1	1	0	0	0	1	1	0
0	0	0	3	1	0	0	1	0	1	0	2
0	0	1	1	0	0	0	?	?	0	1	1
3	1	0	1	0	3	1	0	0	0	0	1
?	?	?	0	?	0	-	0	?	?	2	0

0	0	0	0	?	?	2	0	0	-	0	0
-	0	1	0	0	0	0	1	1	1	1	1
0	0	3	1	0	?	?	?	?	?	?	0
3	1	?	0	?	0	0	?	?	?	?	?
?	?	?	?	?	?	?	?	?	?	?	?
?	?	?	?	?	?	?	?	?	?	?	?
?	?	?	?	?	?	?	?	?	?	?	?
?	?	?	?	?	?	?	?	?	?	?	?
?	0	?	?	?	1	?	0	?	?	0	0
0	2	0	0	0	0	0	-	?	0	0	1
0	?	?	1	1	0	?	?	1	1	1	0
0	?	0	?	1	1	0	0	1	?	2	1
1	5	?	?	?	?	0	?	?	0	0	?
0	0	0									

†Lepidotes gigas

0	0	1	0	-	1	1	1	2	0	0	-
?	?	?	?	0	?	?	?	1	0	1	2
1	?	?	0	?	1	1	?	0	?	?	1
1	0	?	?	0	0	0	0	0	0	1	0
?	2	1	0	2	1	0	?	?	1	1	0
0	1	1	0	0	?	?	?	1	0	0	1
-	?	0	?	?	?	0	0	3	?	0	?
?	?	?	?	?	0	0	0	4	0	0	?
?	0	0&1	0	0	1	0	1	0	1	1	0
3	1	0	0	0	1	2	0	1	1	4	0
0	0	1	1	3	0	0	0	0	1	2	1
1	0	0	3	1	0	0	1	0	1	0	3
1	0	1	0	0	0	0	1	1	0	1	1
3	1	0	0	0	1	1	0	0	1	0	1
0	1	?	0	0	1	0	?	0	0	0	0
0	0	0	1	1	?	2	0	0	-	0	0
-	0	0	0	0	0	4	1	1	2	1	1
0	?	3	0	-	?	?	?	?	1	1	0
0	?	?	0	?	0	0	?	?	?	?	?
?	?	?	?	?	?	?	?	?	?	?	?
?	?	?	?	?	?	?	?	?	?	?	?
?	?	?	?	?	?	?	?	?	?	?	?
?	?	?	?	?	?	?	?	?	?	?	?
?	2	?	?	?	1	2	0	?	1	?	?
0	0	0	0	2	0	0	-	?	0	0	1
0	0	?	?	?	0	0	?	?	1	1	1
1	2	0	2	1	2	0	0	1	0	?	?
1	4	?	?	?	?	0	?	?	0	0	?
0	0	0									

Lepisosteus osseus

4	0	0	0	-	0	0	1	1	0	0	-
0	-	-	0	0	-	-	0	1	0	1	2
1	0	-	1	0	0	-	1	1	-	-	1
1	0	6	1	0	0	1	0	0	1	1	0
2	2	1	1	-	-	1	1	1	1	0	1
-	4	0	1	1	0	1	3	4	0	0	1

-	0	0	0	1	1	0	0	1	1	0	3
3	0	0	0	2	1&2	0	0	4	0	0	0
1	0	0	0	1	1	0	1	0	4	1	0
0	1	1	1	0	1	2	0	1	1	4	0
1	0	2	0	3	1	0	0	0	1	2	0
1	1	0	2	1	0	0	1	0	1	0	3
0	2	0	0	0	0	0	1	2	1	-	-
-	-	-	-	-	-	?	?	?	0	0	1
2	1	1	0	0	1	1	0	1	1	0	0
0	1	0	0	0	0	3	0	0	1	0	0
-	1	0	0	0	1	0	1	0	2	0	0
-	0	4	0	-	0	1	1	1	1	2	1
0	0	1	-	?	1	0	1	2	0	0	0
0	-	2	0	?	1	0	0	1	1	0	0
0	-	1	0	0	0	0	0	0	0	0	0
0	0	1	-	0	0	1	0	0	0	0	4
1	0	0	0	0	0	0	0	0	-	-	-
-	1	1	0	0	3	2	3	3	1	0	0
0	0	0	0	2	0	0	-	?	0	0	1
0	2	0	1	0	0	2	1	0	0	1	1
1	0	0	2	2	0	0	0	1	1	2	1
0	6	0	1	0	0	0	0	0	0	0	0
0	0	0									

†*Leptolepis coryphaenoides*

?	0	?	2	0	-	-	-	-	0	-	-
1	0	?	?	1	0	-	0	0	-	1	1
0	?	?	0	0	1	1	0&1	0	1	1	1
0	?	?	1	0	0	0	0	1	0	1	2
2	1	1	0	0	1	0	1	?	1	1	0
?	?	?	0	0	?	?	?	2	0	1	-
1	0&1	1	?	?	?	1	?	3&4	1	0	2
0	1	0	0	1	0	0	0	1	0	1	?
0	0	0	2	1	1	0	0	0	2	2	?
?	1	0	0	0	0	2	0	0	0	3	0
0	0	1	1	1	1	2	0	0	0	-	-
-	0	?	?	?	?	?	2	0	1	0	0
0	-	1	0	-	0	?	-	-	0	1	0
1	1	1	0	0	2	2	-	0	1	1	1
1	0	1	1	1	1&3	1	1	0	0	0	0
0	0	1	0	-	?	3	0	0	0	0	0
-	1	?	0	0	2	4	1	2	0	0	1
0	0	2	1	2	?	?	?	?	1	1	0
0	0	0	0	2	0	?	-	1	1	1	0
-	0	0	0	0	0	0	?	1	?	0	1
0	-	1	1	1	0	1	?	?	1	?	0
0	0	0	1	1	1	2	1	0	0	3	2
1	1	0	0	1	?	0	0	1	2	0	0
0	0	0	1	1	2	3	?	3	2	1	1
1	2	1	1	-	2	1	1	1	1	1	1
0	0	1	0	1	0	0	0	?	1	1	3
?	0	1	2	0	0	0	0	0	0	3	1

1	2	1	-	0	0	1	1	0	1	0	2
1	0	1									
†*Luisiella feruglioi*											
0	0	1	2	0	-	-	-	-	0	-	-
?	?	?	?	1	0	-	?	?	?	?	?
?	?	?	?	?	?	?	?	?	?	?	?
0	?	?	1	0	0	?	?	?	0	1	2
2	?	?	?	?	?	?	0	-	1	1	?
?	1	0	0	0	1	0	0	1	0	1	-
1	0	1	?	?	?	1	1	4	1	0	?
0	1	0	0	3	0	0	0	3	0	1	?
0	0	0	2	1	1	0	0	0	2	2	1
3	1	0	0	0	0	3	-	0	0	3	0
0	0	1	1	0	1	2	0	0	0	-	-
-	0	0	0	0	0	1	4	-	1	1	-
-	-	-	-	-	-	0	-	-	0	1	0
1	1	1	0	0	2	2	-	0	1	0	1
1	0	3	1	1	4	?	1	0	0	-	0
1	-	-	-	-	?	3	0	0	0	0	0
-	0	1	0	0	2	4	1	2	0	0	1
0	0	1	1	2	?	?	?	?	?	?	0
0	0	0	0	1	0	0	?	?	?	1	0
-	0	0	0	1	0	0	1	1	1	0	1
0	-	0	1	1	0	1	0	1	1	1	0
0	0	0	1	0	0	2	1	0	0	3	3
1	1	0	0	0&1	0	0	0	1	1	0	1
0	0	0	1	1	2	3	?	3	2	1	1
1	2	1	1	-	2	1	1	1	1	1	1
0	0	1	1	1	0	0	0&2	0	1	1	3
3	0	1	2	0	0	?	0	0	1	3	1
0	2	1	-	0	0	1	1	0	1	0	2
1	0	?									
†*Macrepistius arenatus*											
?	0	0	0	-	1	?	?	?	?	?	?
1	0	0	?	0	-	-	?	1	0	1	1
0	?	?	0	?	1	0	?	0	1	2	0
0	0	1	1	1	0	0	0	0	0	?	?
3	?	0	0	2	0	-	0	-	?	?	?
?	?	?	0	0	?	?	?	0	?	?	?
?	?	?	?	?	?	?	?	?	?	?	?
?	?	?	?	?	0	0	0	4	-	0	1
0	0	0	2	1	0	0	1	0	?	?	?
?	?	?	0	0	1	0	1	2	-	3	1
0	1	5	0	8	0	1	0	2	0	-	-
-	0	?	?	?	?	?	?	?	?	0	3
0	0	0	0	0	0	0	?	?	0	1	1
1	1	0	1	0	?	1	0	0	0	0	1
0	1	?	?	?	0	-	?	0	0	0	0
0	0	0	2	?	?	2	0	0	-	0	0
-	0	0	0	0	0	0	1	?	?	1	1
0	?	?	?	?	?	?	?	?	?	?	?

?	?	?	1	?	?	?	0	2	0	0	?
2	-	?	?	?	0	?	?	?	?	?	?
?	?	?	?	?	?	?	?	?	?	?	?
?	?	?	?	?	?	?	?	?	?	?	?
?	?	?	?	?	?	?	?	?	?	?	?
?	0	?	?	?	1	?	?	?	?	?	?
0	?	0	?	2	0	?	-	?	?	?	?
1	?	?	?	?	?	?	?	?	?	?	?
?	?	?	?	?	?	?	?	?	?	?	?
?	?	?	?	?	?	?	?	?	?	?	?
?	?	?									
†*Macrosemimimus fegerti*											
0	0	1	0	-	2	0	1	2	0	0	-
?	?	?	?	0	?	?	?	?	?	?	?
?	?	?	?	?	?	?	?	?	?	?	?
?	?	?	?	0	0	0	?	?	0	1	0
?	?	1	0	?	?	0	?	?	1	1	?
?	1	?	0	0	?	0	0	2	0	0	1
-	0	?	?	?	?	0	0	3	?	0	4
?	?	?	?	0	0	0	1	4	0	0	?
?	0	0	0	1	1	0	1	0	?	1	0
?	1	0	0	0	0	1&2	0	0	0	3	0
0	0	?	?	7	0	-	0	0	1	3	2
0	0	0	3	1	0	0	1	?	1	0	1
0	0	1	0	-	0	0	1	1	0	1	1
4	3	?	0	0	1	1	0	0	1	?	1
0	1	?	0	0	1	1	?	0	0	0	?
0	0	?	2	2&3	1&2	2	0	1	-	0	0
-	0	0	0	0	0	4	1	1	3	1	1
0	0	3	0	-	?	?	?	?	1	1	0
0	0	1	0	?	0	0	?	?	?	?	?
?	?	?	?	?	?	?	?	?	?	?	?
?	?	?	?	?	?	?	?	?	?	?	?
?	?	?	?	?	?	?	?	?	?	?	?
?	?	?	?	?	?	?	?	?	?	?	?
?	0	?	?	?	3	3	1	?	2	?	?
0	0	0	0	1	0	0	-	?	0	0	?
0	0	?	?	0	0	0	?	?	0	1	2
0	2	0	2	1	1	0	0	1	0	2	1
1	4	?	?	?	?	0	0	0	0	0	?
0	0	0									
†*Macrosemius rostratus*											
3	0	1	0	-	1	0	0	-	0	-	-
?	?	?	?	0	?	?	?	?	?	?	2
1	0	-	0	?	1	0	?	1	-	-	1
0	?	?	1	?	0	?	0	0	1	1	0
?	2	1	0	0	1	0	?	?	1	0	?
0	1	1	0	0	?	0	0	4	0	0	1
-	0	0	?	?	?	0	?	3	1	0	?
?	0	0	0	2	0	1	1	-	0	-	?
?	0	0	2	1	3	0	1	0	2	2	0

3	1	1	0	1	0	?	?	?	?	3	0
0	0	0	1	7	0	4	-	0	1	3	0
0	0	0	5	1	0	0	1	0	1	1	-
-	-	-	-	-	-	0	1	0	0	1	1
4	1	0	0	0	?	0	-	-	0	0	1
0	1	1	0	0	1	1	0	0	0	0	0
0	0	0	0	3	3	2	0	0	-	0	0
-	0	1	1	0	0	4	1	1	1	1	1
1	1	3	0	-	?	?	?	1	?	0	1
3	1	-	0	?	0	0	-	1	0	0	?
?	-	0	?	?	?	?	?	?	?	?	?
?	?	?	?	?	?	?	?	?	?	?	?
?	?	?	?	?	?	?	?	?	?	?	?
?	?	?	?	?	?	?	?	?	?	?	?
?	1	?	0	?	3	2	1	?	1	0	0
0	0	0	0	0	0	0	-	?	0	?	1
2	2	0	1	0	0	?	?	1	1	1	3
?	1	1	2	1	1	0	0	0	0	?	?
1	?	1	-	0	?	0	0	0	1	0	0
1	0	0									

†*Masillosteus janeae*

1	0	0	0	-	0	1	1	2	0	0	-
?	?	?	?	0	?	?	?	1	0	?	?
?	0	-	?	?	?	?	0	?	?	?	1
?	?	?	?	1	-	?	?	?	1	1	0
?	?	?	?	?	?	?	?	?	1	0	?
?	3	0	1	0	?	?	?	3	0	0	1
-	?	?	?	?	?	?	?	?	?	0	3
?	?	?	?	?	1	0	0	4	0	0	?
?	0	0	1	0	1	0	1	0	4	1	0
?	1	1	1	0	1	2	0	1	1	4	1
1	0	2	0	3	1	2	0	0	1	4	3
1	0	0	5	1	0	0	1	?	1	0	3
0	2	0	0	0	0	0	1	2	1	-	-
-	-	-	-	-	-	0	-	-	0	0	1
2	1	?	0	?	1	1	0	1	?	2	-
0	0	1	0	2	2	3	0	0	1	0	0
-	1	0	0	0	0	0	1	0	2	0	0
-	0	?	0	-	?	?	?	?	?	?	?
0	?	?	-	?	1	0	1	2	0	0	0
?	-	2	?	?	1	?	?	?	?	?	?
?	?	?	?	?	?	?	?	?	?	?	?
?	?	?	?	?	?	?	?	?	?	?	?
?	?	?	?	?	?	?	?	?	?	?	?
?	1	?	?	?	3	2	3	?	1	?	?
0	0	0	0	-	0	0	-	?	0	0	?
0	2	?	?	0	0	2	?	?	0	1	1
1	1	0	2	2	0	0	0	1	1	2	1
0	6	1	-	0	?	?	?	0	0	0	?
0	0	0									

†*Masillosteus kelleri*

1	0	0	0	-	0	?	?	?	0	0	-
?	?	?	?	0	?	?	?	?	?	?	?
?	?	?	?	?	?	?	?	?	?	?	?
?	?	?	?	1	-	?	?	?	?	1	0
?	?	?	?	?	?	?	?	?	?	?	?
?	?	?	?	0	?	?	?	3	0	0	1
-	?	?	?	?	?	?	?	?	?	0	?
?	?	?	?	?	1	0	0	4	0	0	?
?	0	0	1	0	1	0	1	0	4	?	0
2	1	1	1	0	1	2	0	1	1	4	0
1	0	2	0	3	1	2	0	0	1	5	3
1	0	0	5	1	0	0	1	?	1	0	3
0	2	0	0	0	0	0	1	2	1	-	-
-	-	-	-	-	-	0	-	-	0	0	1
2	1	?	0	?	1	1	0	?	?	2	-
0	0	1	0	2	2	3	0	0	1	0	?
?	1	1	0	0	1	4	1	0	?	0	0
-	0	4	0	-	?	?	?	?	?	?	?
?	?	?	-	?	1	0	1	2	0	0	0
?	-	2	?	?	1	?	?	?	?	?	?
?	?	?	?	?	?	?	?	?	?	?	?
?	?	?	?	?	?	?	?	?	?	?	?
?	?	?	?	?	?	?	?	?	?	?	?
?	1	?	?	?	3	2	3	?	1	?	?
0	0	0	0	-	0	0	-	?	0	0	?
0	?	?	?	0	0	?	?	?	0	1	1
1	1	0	2	2	0	0	0	?	?	?	?
0	5	1	-	?	?	?	?	?	0	0	?
0	0	0									

†*Neosemionotus puntanus*

0	0	1	0	-	0	?	?	?	0	1	1
?	?	?	?	0	?	?	?	?	?	?	?
?	?	?	?	?	?	?	?	?	?	?	?
?	?	?	?	0	0	?	?	?	?	1	0
?	?	?	?	?	?	?	?	?	1	0	?
?	?	?	0	0	?	?	?	?	0	0	1
-	?	0	?	?	?	?	?	3	?	?	?
?	0	0	?	3	2	0	0	4	0	0	?
?	0	0	0	0	0	0	0	?	5	1	0
?	1	?	0	0	1	2	0	1	0	4	0
?	0	1	1	4	0	2	0	0	1	1	1
1	0	0	3	1	0	0	1	?	1	0	2
0	0	0&1	0	0	0	0	1	2	0	1	1
2	1	0	0	0	?	?	?	?	1	0	1
?	1	?	?	?	?	?	?	?	0	0	0
0	0	0	0	?	0	2	0	0	-	0	0
-	0	0	0	0	0	4	1	1	?	1	1
1	?	3	0	-	?	?	?	?	?	?	0
0	0	0	?	?	0	0	?	?	?	?	?
?	?	?	?	?	?	?	?	?	?	?	?
?	?	?	?	?	?	?	?	?	?	?	?

?	?	?	?	?	?	?	?	?	?	?	?
?	?	?	?	?	?	?	?	?	?	?	?
?	2	?	?	?	1	2	0	?	1	?	?
0	0	0	0	1	0	0	-	?	0	1	1
0	0	?	1	1	0	0	?	?	?	1	2
2	2	1	2	1	1	0	?	1	1	2	1
?	?	1	-	?	?	?	?	?	1	0	?
0	0	0									
†*Notagogus denticulatos*											
3	0	1	0	-	1	0	0	-	0	0	-
?	?	?	?	0	?	?	?	?	?	?	?
?	?	?	?	?	?	?	?	?	?	?	?
?	?	?	?	?	0	?	?	?	1	?	?
?	?	?	?	?	?	?	?	?	?	?	?
?	?	?	0	0	?	?	?	?	?	?	?
?	?	?	?	?	?	?	?	?	?	?	?
?	?	?	?	?	0	?	?	?	-	0	?
?	0	?	2	1	3	0	1	?	?	?	?
?	1	1	0	0	?	?	?	?	?	?	0
0	?	0	?	?	0	4	-	?	1	?	?
0	0	0	5	1	0	0	?	?	?	1	-
-	-	-	-	-	-	0	?	?	0	1	1
4	1	2	0	0	?	0	-	-	0	0	1
?	1	?	?	?	?	?	?	0	?	0	0
0	0	0	0	?	?	2	0	0	-	0	0
-	0	1	1	0	0	4	1	?	?	1	1
1	1	3	0	-	?	?	?	?	?	?	?
?	?	?	?	?	?	?	?	?	?	?	?
?	?	?	?	?	?	?	?	?	?	?	?
?	?	?	?	?	?	?	?	?	?	?	?
?	?	?	?	?	?	?	?	?	?	?	?
?	?	?	?	?	?	?	?	?	?	?	?
?	0	?	?	?	3	2	1	?	1	?	?
?	0	0	0	?	0	?	-	?	0	?	1
1	3	?	?	?	0	2	?	?	?	1	3
?	?	?	?	?	?	?	?	?	?	?	?
?	?	?	?	?	?	1	?	?	?	0	?
?	0	0									
†*Notelops brama*											
2	0	1	2	0&1	-	-	-	-	0	-	-
1	0	3	?	1	1	0	0	1	0	3	2
0	?	?	0	?	1	1	2	0	1	1	1
0	0	4	1	0	0	0	0	1	0	1	1
?	2	0	0	0	1	-	?	?	1	1	0
1	2	0	0	0	0	0	0	0	0	1	-
?	2	1	?	?	?	1	?	4	1	0	?
0	0	0	0	3	0	2	0	1	1	1	-
0	0	0	2	1	1	0	0	0	2	2	1
3	1	?	0	0	1	3	-	0	0	2	0
0	0	2	0	1	1	-	0	0	0	-	-
-	0	1	-	-	-	-	3	-	1	1	-

-	-	-	-	-	-	0	-	-	0	1	0
0	2	0	1	0	1	1	1	0	1	0	1
4	0	1	1	1	1	0	1	0	2	1	0
0	2	0	0	-	?	3	0	0	0	0	0
-	0	0	0	0	0	4	?	?	?	0	1
0	0	?	1	?	?	?	?	?	0	-	1
3	0	0	0	0	0	0	0	1	1	1	0
-	1	0	0	1	0	1	?	1	1	?	1
0	-	1	0	0	0	1	0	0	1	0	0
0	0	0	1	0	1	2	1	0	1	4	4
1	2	0	2	2	3	0	0	1	2	1	2
1	0	0	?	1	2	3	?	4	2	?	?
?	2	?	?	-	2	0	1	?	2	-	1
0	0	?	?	0	0	0	0	?	0	1	3
3	?	1	-	1	0	0	0	0	1	0	0
0	2	1	-	0	0	?	1	?	1	?	2
1	0	0									

†*Obaichthys decoratus*

1	1	2	0	-	1	0	1	1	0	0	-
?	-	-	?	0	?	?	0	?	?	?	2
1	0	-	2	0	0	-	1	?	?	?	1
1	0	?	1	-	1	1	0	0	1	1	0
?	2	1	1	-	-	1	1	0	1	?	?
?	3	0	1	0	?	1	0	4	0	0	1
-	0	0	?	1	1	0	0	1	?	0	3
?	0	0	0	2	2	0	0	4	0	0	0
1	0	0	0	1	1	0	1	0	?	?	?
?	1	1	0	0	1	?	0	0	-	3	1
0	0	1	1	7	0	0	0	0	1	5	-
1	0	?	?	?	?	0	?	?	?	0	1
0	0	0	0	-	0	0	1	2	0	1	2
4	1	0	0	0	1	?	?	?	0	0	1
2	1	1	0	0	1	1	0	0	0	?	0
0	0	1	0	0	?	3	0	1	1	0	0
-	0	0	0	0	1	3	1	1	2	1	1
1	0	4	0	-	?	?	?	?	?	?	?
?	?	?	?	?	1	0	1	2	0	0	0
0	-	2	?	?	1	?	?	1	?	?	0
0	-	?	?	?	?	?	?	?	?	?	?
?	?	?	?	?	?	1	0	0	?	?	?
?	?	?	?	?	?	0	0	0	-	-	-
-	?	?	?	?	3	2	?	?	1	?	?
0	0	0	0	?	?	?	?	?	0	0	?
0	?	?	?	?	0	?	?	?	?	1	1
1	0	0	2	2	0	0	0	1	?	-	?
-	?	?	?	0	?	0	?	?	0	0	?
0	0	0									

†*Occitanichthys canjuersensis*

0	0	0	0	-	1	0	1	2	0	1	0
?	?	?	?	0	?	?	?	?	?	?	?
?	?	?	?	?	?	?	?	?	?	?	?

?	?	?	?	1	0	0	0	0	0	1	0
?	?	?	?	?	?	?	?	?	?	?	?
?	1	0	0	0	?	0	0	3	0	0	1
-	0	?	?	?	?	?	?	3	1	0	?
?	?	?	?	?	0	0	0	4	-	0	?
?	0	0	0	0	1	0	1	0	5	1	0
3	1	1	0	0	?	1	0	0	0	3	0
0	0	0	1	-	0	0	0	0	1	4	2
0	0	?	?	?	?	0	?	?	?	0	2
0	0	0	0	0	0	0	1	1	0	1	1
4	3	0	0	0	1	1	0	0	1	0	1
0	1	?	0	0	1	1	?	0	?	?	?
0	0	0	2	2	2	2	0	1	-	0	0
-	0	0	0	0	0	1	1	1	3	1	1
0	1	3	0	-	?	?	?	?	1	1	0
?	0	?	0	?	0	0	?	?	?	?	?
?	?	?	0	?	?	?	?	?	?	?	?
?	?	?	?	?	?	?	?	?	?	?	?
?	?	?	?	?	?	?	?	?	?	?	?
?	?	?	?	?	?	?	?	?	?	?	?
?	?	?	?	?	3	?	1	?	2	?	?
0	1	0	0	?	0	0	-	?	0	0	1
0	?	?	?	0	0	?	?	1	?	1	2
0	?	0	2	1	1	0	0	1	0	2	1
1	4	?	?	?	?	0	?	?	0	0	?
0	0	0									

†*Ophiopsiella attenuata*

3	0	1	0	-	1	0	0	-	1	0	-
?	?	?	?	0	?	?	?	?	?	?	?
0	?	?	?	?	?	?	?	?	?	?	?
?	?	?	1	1	0	?	0	0	?	1	0
?	?	?	?	?	?	?	?	?	?	?	?
?	?	?	0	0	?	?	?	0	1	-	-
0	?	0	?	?	?	?	?	2	1	1	0
?	?	?	?	?	0	0	0	4	0	-	?
?	0	1	2	1	0	0	1	0	3	1	0
3	1	?	0	0	1	1	0	2	0	3	1
0	1	5	0	8	0	3	0	2	0	-	-
-	0	0	3	1	0	0	1	4	1	0	3
0	2	0	0	0	0	0	1	0	0	1	1
1	1	0	1	0	3	1	0	0	1	0	1
0	1	?	0	?	1	0	0	0	0	0	0
0	0	0	0	0	0	2	0	0	-	0	0
-	0	0	0	0	1	1	1	1	1	1	1
0	1	2	1	?	?	?	?	?	1	1	0
2	0	1	1	1	0	1	-	2	0	0	?
2	-	?	?	?	?	?	?	1	?	?	?
?	?	?	?	?	?	?	?	?	?	?	?
?	?	?	?	?	?	?	?	?	?	?	?
?	?	?	?	?	?	?	?	?	?	?	?
?	0	?	?	?	3	3	2	?	0	?	?

0	3	0	1	2	0	?	-	?	0	0	?
1	1	?	?	1	0	?	?	?	?	1	0
0	0	0	0	0	0	0	1	1	0	1	0
1	3	?	?	0	?	0	0	0	0	0	?
0	0	0									

†*Ophiopsis muensteri*

0	0	0	0	-	1	0	1	0	1	0	-
?	?	?	?	0	?	?	?	?	?	?	?
?	?	?	?	?	?	?	?	?	?	?	?
?	?	?	1	?	0	?	?	?	?	1	0
?	?	?	?	?	?	?	?	?	1	0	?
?	?	1	0	0	0	?	?	0	?	?	?
?	?	?	?	?	?	?	?	?	1	1	0
?	?	?	0	?	0	0	0	4	0	0	?
?	0	?	2	1	1	0	1	0	5	1	0
3	1	?	0	0	1	0	0	2	0	3	0
0	0	0	0	8	0	3	0	2	0	-	-
-	0	0	2	1	0	1	1	4	1	0	1
0	0	0	0	-	0	0	?	0	0	1	1
1	1	0	1	1	2	1	0	0	1	0	1
?	1	?	0	0	1	?	0	0	0	0	0
0	0	0	0	?	0	2	0	0	-	0	0
-	0	1	0	0	1	1	1	1	?	1	1
0	1	2	1	?	?	?	?	?	?	?	?
2	0	1	1	1	0	0	?	?	?	?	?
?	?	0	?	?	?	?	?	?	?	?	?
?	?	?	?	?	?	?	?	?	?	?	?
?	?	?	?	?	?	?	?	?	?	?	?
?	?	?	?	?	?	?	?	?	?	?	?
?	0	?	?	?	3	3	1	?	0	0	0
0	2	0	0	2	0	0	-	?	0	0	1
0	0	?	?	1	0	0	?	?	?	1	0
0	0	0	0	1	0	0	0	0	0	1	0
1	3	?	?	0	?	0	0	0	1	0	0
0	0	0									

†*Orthogonikleithrus hoelli*

2	0	1	2	?	-	-	-	-	?	-	-
?	?	?	?	1	?	?	?	?	?	?	?
?	?	?	?	?	?	?	?	?	?	?	?
0	?	5	1	0	0	0	0	0	0	1	2
?	1	?	0	?	1	?	0	-	1	1	?
?	?	?	0	0	?	0	0	2	0	1	-
1	0	1	?	?	?	1	?	4	1	0	2
0	0	?	0	3	?	?	0	?	?	?	?
?	0	?	2	1	1	0	0	0	?	?	?
?	?	0	0	0	0	3	0	0	0	?	?
0	0	?	?	?	?	0	0	0	0	-	-
-	0	?	?	?	-	?	4	-	1	1	-
-	-	-	-	-	-	0	-	-	0	1	0
2	1	1	0	0	2	2	-	0	0	0	1
1&4	0	3	0	1	1	?	1	0	-	-	0

	1	-	-	-	-	?	3	0	0	0	0	0
	-	0	0	0	0	2	4	1	2	0	1	1
	0	0	2	0	-	?	?	?	?	?	?	?
	?	?	?	0	0	0	?	0	1	1	1	0
	-	1	0	0	1	0	0	1	1	1	0	1
	1	?	1	2	2	0	0&1	0	0	1	1	0
	0	0	0	1	0	0	2	1	0	0	?	3
	1	1	0	2	1	0	0	1	1	2	?	1
	2	0	0	1	1	2	3	?	4	2	1	1
	0	2	1	0	-	2	1	1	1	2	-	1
	0	0	1	1	1	0	?	0	?	?	1	3
	3	0	1	-	0	?	1	0	0	1	3	1
	0	?	1	-	0	0	1	1	?	1	?	2
	1	0	?									
†*Oshunia brevis*												
	0	0	1	1	-	-	-	-	-	?	-	-
	1	1	0	?	0	-	-	1	1	0	1	2
	0	1	0	0	?	?	?	?	0	1	2	0
	1	0	0	?	1	0	0	0	0	0	?	?
	1	2	0	0	0	0	-	1	1	1	0	?
	?	?	?	0	0	0	?	?	0	1	-	-
	?	?	?	?	?	?	0	?	?	1	1	0
	?	?	?	?	?	0	0	0	?	?	0	1
	2	0	0	2	1	1	0	0	0	6	2	1
	?	1	?	0	1	-	-	-	-	-	3	0
	0	1	?	?	?	?	?	1	?	?	?	?
	?	?	?	?	?	?	?	?	?	?	0	?
	?	?	?	?	?	?	?	?	?	0	1	1
	1	1	0	?	?	?	?	?	?	?	?	?
	?	?	?	?	?	?	?	?	?	?	?	0
	?	?	?	?	?	?	?	?	?	?	0	0
	-	?	0	?	?	0	?	1	?	?	?	?
	?	?	?	1	?	?	?	?	?	?	?	?
	?	0	?	1	?	0	?	0	2	0	0	0
	0	-	1	?	?	0	0	?	1	0	?	0
	0	-	0	0	0	0	?	0	0	0	0	?
	?	?	?	?	?	0	0	0	1	?	0	?
	?	-	?	0	0	0	0	0	0	-	-	-
	-	0	0	0	?	1	?	?	?	?	?	?
	?	?	?	?	?	1	?	?	?	0	0	1
	1	?	?	?	?	0	?	?	?	?	1	?
	?	?	?	?	0	1	0	?	1	?	2	?
	?	?	?	?	?	?	?	?	?	?	0	0
	?	0	0									
†*Pachyamia latimaxillaris*												
	3	0	0	1	-	-	-	-	-	?	-	-
	?	?	?	?	0	?	?	?	?	?	?	?
	1	?	?	?	?	?	?	1	?	?	?	?
	1	?	?	?	?	0	?	?	?	?	?	?
	?	2	?	?	?	?	?	?	?	?	?	?
	0	?	1	0	0	0	0	?	0	1	-	-

0	?	?	?	?	?	0	?	2	1	1	0
?	?	?	?	?	?	0	0	0	0	0	0
?	0	0	2	1	0	1	0	1	0	0	0
?	1	?	0	0	?	?	0	0	0	1	1
0	1	3	0	2	0	2	0	0	0	-	-
-	0	0	2	1	0	?	1	?	1	1	-
-	-	-	-	-	-	0	?	?	0	1	1
0	1	0	1	2	1	1	1	0	0	0	1
0	1	?	0	?	1	0	0	0	0	0	0
0	?	0	4	0	0	2	0	0	-	0	0
-	0	0	0	0	3	2	1	?	?	1	1
1	1	1	1	0	?	1	?	?	?	?	?
?	?	?	0	?	?	?	0	2	0	0	1
2	-	0	1	0	0	0	1	?	?	?	0
0	-	0	0	0	0	?	0	0	1	1	0
0	0	?	?	0	0	?	0	0	?	4	3
?	?	1	?	0	0	0	0	0	-	-	-
-	?	?	?	?	?	?	?	?	?	?	?
?	?	?	?	?	?	?	?	?	?	?	1
1	?	?	?	?	0	?	?	?	?	1	3
?	?	?	?	0	0	1	?	0	?	1	0
?	?	?	?	0	?	0	?	?	?	0	1
1	0	0									

†*Pachyamia mexicana*

3	0	0	1	-	-	-	-	-	?	-	-
?	?	?	?	0	?	?	?	?	?	?	?
1	?	?	?	?	?	?	?	?	?	?	?
?	?	?	?	1	0	?	?	?	?	?	?
?	2	?	?	?	?	?	?	?	1	0	?
0	?	1	0	0	?	0	?	0	1	-	-
0	?	?	?	?	?	?	?	?	1	1	0
?	?	?	?	?	0	0	0	0	0	0	?
?	0	0	2	1	0	1	0	1	0	0	0
2	1	0	0	0	?	?	?	0	0	1	1
0	1	?	0	2	0	2	0	0	0	-	-
-	0	0	2	1	0	1	1	4	1	1	-
-	-	-	-	-	-	0	?	?	0	1	1
0	0	1	0	2	1	1	1	0	0	0	1
0	1	2	0	0	1	0	0	0	0	0	0
0	0	0	4	0	0	2	0	0	-	0	0
-	0	0	0	0	3	2	1	?	1	1	1
1	1	1	1	0	?	1	?	?	?	?	?
3	?	?	0	?	0	0	0	2	0	0	1
2	-	0	1	0	0	0	1	?	?	?	0
0	-	0	0	0	0	0	0	0	1	1	0
0	0	0	0	0	0	0	0	0	?	4	2
1	3	1	0	0	0	0	0	0	-	-	-
-	1	1	0	0	3	0	?	3	0	0	0
0	2	1	0	-	1	0	2	?	2	-	1
1	?	0	1	1	0	?	?	?	?	1	?
3	0	1	-	0	0	1	0	0	?	1	0

?	?	?	?	0	?	0	0	?	1	0	1
1	0	0									

†*Pachycormus macropterus*

0	0	1	?	-	-	-	-	-	0	-	-
1	0	0	?	0	-	-	0	?	?	5	3
?	0	-	?	0	1	0	1	0	1	0	0
0	0	2	1	0	0	0	0	1	0	1	1
1	1	0	0	2	1	-	1	1	1	0	?
0	1	1	0	0	0	0	1	0	?	?	?
0	0	0	0	?	?	0	?	2	1	0	1
1	0	1	0	2	-	-	-	-	1	-	?
0	1	0	2	0	0	0	0	0	0	2	0
?	1	0	0	1	0	-	-	-	-	0	0
0	0	1	?	6	0	2	0	0	0	-	-
-	0	0	1	0	0	0	3	-	1	0	1
0	0	1	0	-	0	1	0	-	0	1	1
0	2	0&2	1	0	?	1	1	1	0	0	1
0	1	1	0	0	3	-	0	0	0	0	0
0	0	0	0	0	?	?	0	?	?	?	0
-	0	0	0	0	3	4	1	?	?	?	1
0	0	0	1	?	2	1	?	1	?	?	0
0	3	-	0	-	0	?	-	1	1	0	?
-	-	?	?	0	-	0	?	1	?	?	0
?	?	0	0	0	0	0	0	0	0	0	0
0	0	0	0	0	0	-	-	-	0	0	0
0	-	1	?	2	0	0	0	0	-	-	?
-	0	-	?	-	1	0	?	?	0	1	0
1	?	1	0	-	0	0	0	?	?	0	?
1	0	0	1	?	?	0	?	?	?	1	0
0	?	?	?	?	?	?	1	?	1	2	?
?	2	?	?	0	0	1	1	1	0	0	-
-	-	-									

†*Panxianichthys imparilis*

0	0	0	0	-	1	0	1	0	0	0	-
?	?	?	?	0	?	?	?	?	?	?	?
?	?	?	?	?	?	?	?	?	?	?	?
?	?	?	?	1	0	?	?	?	?	?	?
?	?	0	0	?	0	-	?	?	?	?	?
?	?	?	0	0	0	?	?	?	0	0	0
-	?	?	?	?	?	?	?	?	1	1	0
?	0	0	0	1	0	0	0	?	0	0	?
0	0	0	0	1	1	0	0	0	3	1	0
3	1	0	0	0	1	2	0	0	0	3	0
0	0	0	1	8	0	2	0	1	0	-	-
-	0	0	2	1	0	1	1	4	1	0	1&2
0	0	1	0	0	0	0	?	?	0	1	1
1	1	0	1	1	3	1	0	0	1	0	1
?	1	?	0	0	0	-	?	0	0	0	0
0	0	0	0	?	?	2	0	0	-	0	0
-	0	0	0	0	0	1	1	2	0	0	1
0	1	?	1	?	?	?	?	?	?	?	?

0	0	?	1	?	0	0	?	?	?	?	?
?	?	?	?	?	?	?	?	?	?	?	?
?	?	?	?	?	?	?	?	?	?	?	?
?	?	?	?	?	?	?	?	?	?	?	?
?	?	?	?	?	?	?	?	?	?	?	?
?	0	?	?	?	1	3	0	?	0	?	?
0	2	0	0	1	0	0	2	2	0	0	?
0	0	?	?	1	0	?	?	?	1	1	0
0	2	0	0	0	0	0	1	0	?	1	?
1	3	?	?	?	?	?	?	?	0	0	?
0	0	0									

†*Paralepidotus ornatus*

3	0	0	0	-	1	0	1	1	0	0	-
?	?	?	?	0	?	?	?	?	?	?	?
?	?	?	?	?	?	?	?	?	?	?	?
?	?	?	?	?	?	?	?	?	?	?	?
?	?	?	?	?	?	?	?	?	1	0	?
?	1	0	0	0	?	0	0	3	0	0	1
-	?	?	?	?	?	?	?	?	?	?	?
?	?	?	?	?	0	0	0	4	0	0	?
?	0	0	2	1	3	0	1	0	?	?	?
?	1	?	0	0	0	0	0	0	0	3	0
?	0	?	?	0	0	2	?	0	1	?	?
?	0	?	?	?	?	?	?	?	1	0	0
-	-	1	0	-	0	?	?	?	0	1	?
?	?	?	?	?	?	?	?	?	?	?	1
?	?	?	?	?	?	?	?	?	?	?	?
0	0	?	?	3	?	2	0	1	-	0	0
-	0	0	0	0	0	?	1	1	2	1	1
0	?	?	0	-	?	?	?	?	?	?	?
?	?	?	?	?	?	0	-	0	0	0	?
?	-	?	?	?	-	?	?	?	?	?	?
?	?	?	?	?	?	?	?	?	?	?	?
?	?	?	?	-	-	?	?	?	?	?	?
?	?	?	?	?	?	?	?	?	?	?	?
?	0	?	?	?	1	3	0	?	2	?	0
0	2	0	0	1	0	0	-	?	0	0	?
1	2	?	?	?	0	?	?	?	?	1	0
?	?	?	?	?	?	?	?	?	?	2	1
1	?	?	?	?	?	?	?	0	0	0	?
0	0	0									

†*Parapholidophorus nybelini*

0	0	1	0	-	0	?	?	?	0	0	-
?	?	?	?	0	-	-	?	?	?	?	?
?	?	?	?	?	?	?	?	?	?	?	?
1	?	?	?	?	?	?	?	?	?	0	-
?	?	?	?	?	?	?	0	?	?	?	?
?	?	?	0	0	?	0	?	2	?	?	?
0	?	0	?	?	?	?	?	1	1	1	0
1	0	0	0	2	0	0	0	-	-	-	?
0	0	-	-	-	1	-	?	0	0	2	1

0	1	0	0	0	0	1&2	0	0	0	0	0
0	0	1	1	0	1	2	0	0	0	-	-
-	0	0	0	0	0	1	1	2	1	0	0
-	-	0	0	-	0	0	-	-	0	1	0
2	1	1	0	0	2	2	-	0	1	1	1
0	1	?	0	?	?	-	?	?	?	1	0
0	?	1	0	?	?	2	0	0	-	0	1
1	0	1	0	0	2	4	1	1	1	0	1
?	0	?	1	?	?	?	?	?	?	?	1
3	0	0	0	1	?	0	-	0	1	0	1
-	-	?	?	?	-	?	?	?	?	?	?
?	?	-	?	?	?	?	?	?	0	0	?
?	?	?	?	-	-	2	0	0	?	?	?
?	?	?	?	0	0	0	?	?	?	?	?
?	0	?	?	?	1	3	0	?	2	0	?
0	2	0	0	0	0	0	1	1	0	0	?
0	0	?	?	1	0	?	?	?	?	1	0
?	0	0	2	?	?	?	?	?	0	2	0
1	2	0	0	?	?	0	?	?	0	1	?
0	?	1									

†*Pholidoctenus serianus*

0	0	1	0	-	1	0	0	-	0	0	-
?	?	?	?	0	-	-	?	?	?	?	?
?	?	?	?	?	?	?	?	?	?	?	?
1	?	?	?	0	0	?	?	?	?	0	-
?	?	?	?	?	?	?	0	?	?	?	?
?	?	?	0	0	0	?	?	1	?	?	?
0	0	0	?	?	?	?	?	?	?	?	?
?	?	?	?	?	0	2	0	-	-	-	?
?	0	?	?	?	1	?	0	0	3	1	0
0	1	0	?	0	0	2	0	0	0	0	0
0	0	1	1	1	1	2	0	0	0	-	-
-	0	0	0	0	0	1	1	2	1	0	1
0	0	0	0	-	0	0	-	-	0	1	0
1	1	1	1	0&1	1	2	-	0	1	?	1
2	1	?	0	?	?	?	?	0	0	?	0
0	0	1	0	?	?	2	0	0	-	0	0
-	1	1	0	0	2	4	1	1	1	0	1
0	0	2	1	?	?	?	?	?	?	?	1
0	0	0	0	?	0	0	-	0	1	0	1
-	-	?	?	0	-	0	?	?	?	?	0
0	-	-	0	0	0	0	0	0	0	0	0
0	0	0	0	-	-	2	0	0	0	3	3
1	?	0	1&2	0	?	1	0	0	-	-	-
-	0	?	?	?	1	3	0	?	0	0	?
0	2	0	0	?	0	0	1	1	0	0	?
0	0	?	?	1	0	0	?	?	?	1	0
0	?	0	0&2	0	?	0	0	?	0	2	?
1	2	0	0	?	?	0	?	?	0	1	?
0	0	1									

†*Pholidophoretes salvus*

4	0	1	0	-	0	?	?	?	0	?	?
?	?	?	?	0	-	-	?	?	?	?	?
?	?	?	?	?	?	?	?	?	?	?	?
1	?	?	?	?	0	?	?	?	?	?	?
?	?	?	?	?	?	?	?	?	?	?	?
?	?	?	0	0	?	?	?	1	?	?	?
?	?	?	?	?	?	?	?	?	?	?	?
?	?	?	?	?	0	2	0	-	-	-	?
0	0	-	-	-	1	-	?	0	3	1	0
1	0	0	0	0	?	2	0	0	0	?	0
0	0	1	1	1	1	?	?	?	0	-	-
-	0	?	?	?	?	?	?	?	1	0	0
-	-	0	0	-	0	?	?	?	0	1	0
0	1	1	1	?	2	1	0	0	?	?	?
?	1	?	0	?	?	?	?	?	0	?	0
0	?	?	0	?	?	2	0	0	-	0	1
0	0	1	0	0	2	4	1	1	1	0	1
?	0	2	1	?	?	?	?	?	?	?	?
?	0	0	0	1	?	?	-	?	1	0	?
-	-	?	?	?	?	?	?	?	?	?	?
?	?	-	?	?	?	?	?	?	?	?	?
?	?	?	?	-	-	?	?	?	?	?	?
?	?	?	?	?	?	?	?	?	?	?	?
?	0	?	?	?	1	?	0	?	?	?	?
0	?	?	0	?	0	0	?	?	0	0	?
0	?	?	?	?	0	?	?	?	?	1	?
?	?	0	2	?	?	?	?	?	?	?	?
1	2	?	?	?	?	?	?	?	0	?	?
0	?	?									

†*Pholidophorus gervasutti*

?	0	0	0	-	0	?	?	?	0	0	-
?	?	?	?	0	-	-	?	?	?	?	?
?	?	?	?	?	?	?	?	?	?	?	?
?	?	?	?	?	0	?	?	?	?	0	-
?	?	?	?	?	?	?	?	?	?	?	?
?	?	?	0	0	?	?	?	1	?	?	?
0	0	0	?	?	?	?	?	2	1	1	0
0	?	0	0	3	0	0	0	-	-	-	?
?	0	?	?	?	1	?	0	0	0	1	1
1	?	0	0	0	?	2&3	0	0	0	3	0
0	0	1	1	6	?	2	0	?	0	-	-
-	0	0	?	?	0	?	1	2	1	0	0
-	-	1	0	-	0	?	-	-	0	1	0
0	1	1	0&1	0	2	2	-	0	1	1	1
0	1	3	0	?	3	-	?	0	?	0	0
0	0	?	0	?	?	2	0	0	-	0	0
-	0	1	1	?	2	0	1	2	1	1	1
?	0	2	1	2	?	?	?	?	?	?	?
0	0	0	0	?	?	?	-	0	1	0	1
?	-	?	?	?	-	?	?	?	?	?	0
0	?	-	?	?	?	?	?	?	?	?	?

?	?	?	?	-	-	?	?	?	?	?	?
?	?	?	?	?	?	?	?	?	?	?	?
?	0	?	?	?	1	3	0	?	0	0	?
0	2	0	1	0	2	0	1	1	0	0	1
0	0	?	?	1	0	?	?	?	?	1	0
?	?	0	2	2	?	?	?	?	1	3	0
1	2	0	0	?	?	0	?	?	0	1	?
0	0	1									
†*Pholidorhynchodon malzanni*											
0	0	0	0	-	0	?	?	?	0	0	-
?	?	?	?	0	-	-	?	?	?	?	?
?	?	?	?	?	?	?	?	?	?	?	?
1	?	?	?	?	0	?	?	?	?	1	1
1	?	1	0	2	1	?	0	-	?	?	?
?	?	?	0	0	0	?	?	0	?	?	?
?	?	0	?	?	?	?	?	?	?	?	?
?	0	0	0	2	0	0	0	-	0	-	?
0	0	1	2	1	1	0	0	0	0&3	2	1
1	1	0	0	0	0	1&2	0	0	0	0	0
0	0	1	1	1	1	2	0	?	0	-	-
-	0	0	0	0	0	1	1	2	1	0	0&1
0	0	?	0	-	0	1	0	-	0	1	0
0	1	1	0	0	2	2	-	0	1	1	1
0	1	3	0	0	4	-	0	?	1	1	0
0	0	0	0	0	?	2	0	0	-	0	1
1	0	1	1	0	2	1	1	1	0	0	1
?	0	2	1	2	?	?	?	?	?	?	1
2	0	0	0	?	?	?	-	0	1	0	1
-	-	?	?	?	-	0	?	?	?	?	?
?	?	-	?	?	?	?	?	?	?	?	?
?	?	?	?	-	-	?	?	?	?	?	?
?	?	?	?	?	?	?	?	?	?	?	?
?	0	?	?	?	1	?	0	?	?	0	?
0	2	0	?	1	0	0	?	?	0	0	?
0	0	0	?	1	0	0	?	?	?	1	0
?	0	0	0	0	0	1	0	?	0	2	0
1	2	0	0	?	?	0	0	0	0	1	?
0	3	1									
†*Plesiofuro mingshuica*											
0	0	?	0	-	1	0	1	0	0	0	-
?	?	?	?	0	?	?	?	?	?	?	?
?	?	?	?	?	?	?	?	?	?	?	?
?	?	?	?	?	?	?	?	?	?	?	?
?	?	?	?	?	?	?	?	?	?	?	?
?	?	?	0	0	?	?	?	0	?	?	?
?	?	?	?	?	?	?	?	?	?	?	?
?	0	0	0	0	0	0	0	0	0	0	?
?	0	1	0	0	1	1	0	0	0	2	0
0	0	0	0	0	1	0	0	0	?	3	0
?	0	0	3	0	1	2	?	0	0	-	-
-	0	0	1	0	0	1	?	-	0	0	0

- - 0 0 - 0 1 ? ? 0 0 -
0 0 3 0 0 0 0 - - 0 0 0
- 1 ? 0 ? 0 - ? 0 0 0 0
0 0 0 0 ? ? 1 0 - - - 0
- 0 0 0 0 0 ? 0 - - 2 0
- 0 - 1 0 ? ? ? ? 1 2 0
1 0 0 0 ? 0 0 ? ? ? ? ?
? ? ? ? ? ? ? ? ? ? ? ?
? ? ? ? ? ? ? ? ? ? ? ?
? ? ? ? ? ? ? ? ? ? ? ?
? ? ? ? ? ? ? ? ? ? ? ?
? 0 ? ? ? 1 1 ? ? 0 ? ?
0 2 0 0 ? 0 0 - ? 0 0 1
0 0 ? ? 1 0 0 ? ? 1 1 0
0 1 0 2 0 0 0 0 0 0 2 1
? ? 1 - ? ? 0 ? 0 0 0 ?
? 0 0

†*Pliodetes nigeriensis*

2 0 2 0 - 1 0 1 1 0 0 -
? ? ? ? 0 ? ? ? ? ? ? ?
? ? ? ? ? ? ? ? ? ? ? ?
? ? ? ? 0 ? ? ? ? ? 1 0
? ? ? ? ? ? ? ? ? 1 0 ?
? ? ? 0 0 ? ? ? ? ? ? ?
? ? ? ? ? ? ? ? 3 ? ? ?
? ? ? ? ? 1 0 0 4 0 0 ?
? 0 0 1 0 0 0 0 0 1 1 0
3 1 ? 1 0 1 2 0 1 1 4 0
? 0 1 0 3 0 0 0 0 1 1 0
1 0 0 3 1 0 0 1 0 1 0 3
0 2 0 0 0 0 0 1 2 0 1 2
4 1 0 ? ? ? ? ? ? 0 0 1
4 0 ? ? ? 1 ? ? ? 0 0 1
0 0 1 0 ? 0 3 0 0 0 0 0
- 0 0 0 0 1 3 1 1 1 0 1
0 ? ? 0 - ? ? ? ? ? ? 1
? ? ? ? ? ? ? ? ? ? ? ?
? ? 0 ? ? 0 ? ? ? ? ? ?
? ? ? ? ? ? ? ? ? ? ? ?
? ? ? ? ? ? ? ? ? ? ? ?
? ? ? ? ? ? ? ? ? ? ? ?
? ? ? ? ? ? ? ? ? ? ? ?
? ? ? ? ? ? ? ? ? ? ? 1
0 ? ? 0 ? 0 ? ? 1 ? 1 1
? ? 0 2 1 ? 0 ? 1 ? 2 1
? ? ? ? ? ? ? ? ? 0 0 ?
0 0 ?

†*Propterus elongatus*

3 0 1 0 - 1 0 0 - 0 0 -
? ? ? ? 0 ? ? ? ? ? ? ?
? ? ? ? ? ? ? ? ? ? ? ?

?	?	?	1	?	0	?	?	?	1	?	?
?	?	1	0	?	?	0	?	?	1	0	?
?	?	?	0	0	?	0	?	1	0	0	1
-	0	?	?	?	?	0	0	3	?	0	?
?	?	?	?	?	0	1	1	-	-	0	?
?	0	0	2	1	3	0	1	?	2	2	?
?	1	1	0	0	0	1	0	0	0	3	0
0	0	0	1	7	0	4	-	0	1	3	0
0	0	0	5	1	0	0	1	0	1	1	-
-	-	-	-	-	-	0	?	?	0	1	1
4	1	2	0	0	?	0	-	-	0	0	?
?	?	?	?	?	?	?	?	0	0	0	0
0	0	0	0	?	0	2	0	0	-	0	0
-	0	0	1	0	0	4	1	1	1	1	1
1	1	3	0	-	?	?	?	?	?	?	1
3	0	0	0	?	0	0	?	?	?	?	?
?	?	?	?	?	?	0	?	1	0	?	?
?	?	?	?	?	?	?	?	?	?	?	?
?	?	?	?	?	?	?	?	?	?	?	?
?	?	?	?	?	?	?	?	?	?	?	?
?	0	?	0	?	3	2	2	?	1	0	0
0	2	0	0	0	0	0	-	?	0	1	1
1	3	0	1	0	0	2	?	?	0	1	3
3	2	0	0	1	1	0	0	?	?	2	1
1	?	0	1	0	?	0	0	0	1	0	?
0	0	0									

†*Quetzalichtys perrilliatae*

0	0	1	1	-	-	-	-	-	?	-	-
?	?	?	?	0	?	?	?	?	?	?	?
?	?	?	?	?	?	?	?	?	?	?	?
?	?	?	?	1	?	?	?	?	?	?	?
?	?	?	?	?	?	?	?	?	?	?	?
?	?	?	0	0	?	?	?	0	1	-	-
0	?	?	?	?	?	?	?	?	1	1	0
?	?	?	?	?	0	0	0	2	1	0	?
2	0	0	2	1	1	0	1	0	6	2	1
?	1	1	0	0	1	2	0	2	0	3	0
?	1	0	1	8	0	3	1	1	0	-	-
-	0	0	3	1	0	?	1	4	1	0	2
0	0	0	0	0	1	?	?	?	0	1	1
0	1	0	1	1	1	1	0	0	0	?	1
?	1	?	0	0	?	?	0	0	0	0	0
0	0	0	0	0	?	2	0	0	-	0	0
-	0	0	0	0	0	4	1	1	0	1	1
?	0	2	1	0	?	?	?	?	?	?	?
0	0	?	1	?	?	?	0	2	0	0	0
0	-	1	0	?	0	0	0	1	0	0	0
0	-	0	0	0	0	0	0	0	0	0	?
?	?	0	0	0	0	0	0	0	?	0	0
1	-	1	0	0	0	0	0	0	-	-	-
-	?	0	0	1	1	?	?	2	?	?	0

0	2	0	1	-	1	0	2	?	0	0	1
1	?	0	1	?	0	?	1	0	?	1	0
0	0	0	0	0	1	?	0	1	?	1	?
?	?	?	?	0	?	?	?	?	1	0	?
0	0	0									
†*Rhacolepis buccalis*											
2	0	1	2	0&1	-	-	-	-	0	-	-
1	0	3	?	1	1	0	0	1	0	3	2
1	0	-	1	?	1	1	2	0	?	?	1
0	0	5	1	0	0	0	?	1	0	1	1
2	2	0	0	-	1	-	?	?	1	1	0
?	2	1	0	0	0	0	2	0	0	1	-
?	1	1	?	?	?	1	?	4	1	0	?
0	0	0	0	3	0	2	0	1	1	1	-
2	1	0	2	1	1	1	0	0	2	2	1
3	1	?	0	0	1	3	-	0	0	2	0
0	0	3	1	0	1	-	0	0	0	-	-
-	0	1	-	-	-	-	3	-	1	1	-
-	-	-	-	-	-	0	-	-	0	1	0
0	2	0	1	0	1	1	0	0	1	0	1
4	0	3	1	1	1	0	1	0	2	1	0
0	0	0	0	-	0	3	0	0	0	0	0
-	0	0	0	0	0	4	1	1	1	1	1
0	0	0	0	-	2	0	?	?	0	-	1
3	0	0	0	1	0	0	0	1	1	1	0
-	1	0	0	0	0	1	?	?	?	?	1
0	-	1	0	0	0	1	0	0	0	0	1
1	1	0	1	1	1	2	1	0	0	?	4
1	2	0	2	2	3	0	0	1	2	1	2
2	0	0	1	1	2	3	?	4	2	?	1
?	2	?	?	-	2	0	1	1	2	-	1
0	?	?	?	?	0	0	0	?	?	?	?
?	?	1	-	?	0	0	0	0	2	0	0
0	2	1	-	0	0	?	1	?	1	?	2
1	0	0									
†*Robustichthys luopingensis*											
3	0	0	0	-	?	?	?	?	0	0	-
?	?	?	?	0	?	?	?	?	?	?	?
?	?	?	?	?	?	?	?	?	?	?	?
?	?	?	?	?	?	?	?	?	?	1	0
?	?	?	?	?	?	?	?	?	?	?	?
?	?	?	0	0	?	?	?	0	1	-	-
?	?	?	?	?	?	?	?	0	1	1	0
1	?	?	?	?	0	0	0	1	0	0	?
?	?	0	0	0	?	0	0	0	5	1	0
3	1	?	0	0	1	2	0	0	0	3	0
?	?	0	1	8	0	2	0	1	0	-	-
-	0	0	2	1	0	1	1	0	1	0	3
0	2	0	0	0	0	0	1	1	0	1	1
1	1	0	0	0	3	1	0	0	1	0	1
?	1	?	0	?	0	-	?	0	0	0	0

0	0	0	0	0	0	2	0	0	-	0	0
-	0	0	0	0	0	1	1	1	2	1	1
0	?	?	1	?	?	?	?	?	?	?	?
0	?	?	1	?	?	0	?	?	?	?	?
?	?	?	?	?	?	?	?	?	?	?	?
?	?	?	?	?	?	?	?	?	?	?	?
?	?	?	?	?	?	?	?	?	?	?	?
?	?	?	?	?	?	?	?	?	?	?	?
?	2	?	?	?	1	1	0	?	0	?	0
0	2	0	0	2	0	0	0	2	0	0	?
1	0	?	?	1	0	0	?	?	1	1	0
0	?	0	0	0	?	0	1	?	?	2	1
?	4	?	?	?	?	?	?	?	0	0	?
0	0	0									

Salmo salar

2	0	1	2	0	-	-	-	-	0	-	-
1	3	3	1	1	1	0	0	0	-	3	2
0	1	0	0	?	1	1	?	0	1	0	1
0	0	3	1	0	0	0	0	1	0	1	1
2	2	0	0	0	1	-	0	-	1	1	0
1	5	0	0	0	0	0	0	0	0	1	-
0	0	1	1	1	0	1	?	3	1	0	2
0	0	0	0	3	1	1	0	2	1	?	?
1	1	0	?	?	1	0	?	0	2	2	1
3	1	?	?	0	0	3	-	0	0	2	0
0	0	1	0	2	0	2	0	0	0	-	-
-	0	0	6	-	1	1	3	-	1	1	-
-	-	-	-	-	-	0	-	-	0	1	?
3	1	?	0	0	2	1	0	0	1	0	1
4	0	3	?	1	1	0	1	0	0	0	0
0	?	0	0	-	0	3	0	0	0	0	0
-	0	1	0	1	0	4	1	1	1	1	1
?	0	2	0	-	2	0	3	1	0	?	1
3	0	0	0	0	0	?	0	1	1	1	0
-	1	0	?	1	0	0	?	0	?	1	0
0	-	0	0	0	0	0	0	0	0	1	?
?	?	?	?	?	0	2	1	0	0	1	4
1	1&2	0	2	0	0	0	1	1	2	1	1
2	0	?	?	?	2	3	?	4	2	0	0
0	?	1	?	-	2	0	?	?	2	-	1
0	0	0	?	?	0	?	?	?	?	1	3
?	0	1	?	0	0	0	0	?	0	1	1
0	4	1	-	0	0	1	1	?	1	0	2
1	?	0									

†*Sangiorgioichthys aldae*

0	0	0	0	-	2	0	0	-	0	1	0
?	?	?	?	0	?	?	?	?	?	?	?
?	?	?	?	?	?	?	?	?	?	?	?
?	?	?	?	0	?	?	?	?	?	?	?
?	?	?	?	?	?	?	?	?	?	?	?
?	?	?	0	0	?	?	?	2	?	?	?

?	?	?	?	?	?	0	?	3	?	0	?
?	?	?	?	?	0	0	0	4	0	0	?
?	0	0	0	1	1	0	0	0	5	2	0
?	1	?	0	0	0	2	0	0	0	3	0
?	0	0	1	1	0	3	0	0	1	0	-
-	0	0	?	?	0	?	1	?	1	0	3
1	0&1	0	0	0	1	0	?	?	0	1	1
3	1	0	0	0	2	1	0	0	1	0	1
2	1	?	0	?	0	-	?	0	?	0	1
0	0	1	0	?	?	2	0	0	-	0	0
-	0	0	0	0	0	0	1	1	1	1	1
0	1	?	0	-	?	?	?	?	?	?	0
?	0	?	?	?	0	0	?	?	?	?	?
?	?	?	?	?	?	?	?	?	?	?	?
?	?	?	?	?	?	?	?	?	?	?	?
?	?	?	?	?	?	?	?	?	?	?	?
?	?	?	?	?	?	?	?	?	?	?	?
?	?	?	?	?	?	?	?	?	?	?	?
?	?	0	?	0	0	?	-	?	0	?	?
0	0	?	?	?	0	?	?	?	?	1	0
?	?	0	0	0	?	0	0	1	?	2	1
1	3	1	-	?	?	?	?	?	0	0	?
?	0	?									

†*Sangiorgioichthys sui*

0	0	0	0	-	1	0	0	-	0	1	0
?	?	?	?	0	?	?	?	?	?	?	?
?	?	?	?	?	?	?	?	?	?	?	?
?	?	?	?	0	?	?	?	?	?	1	0
?	?	?	?	?	?	?	?	?	1	0	0
?	1	1	0	0	0	0	0	1	0	0	1
-	0	0	?	?	?	0	0	3	?	0	?
?	0	0	0	1	0	0	0&1	4	0	0	?
?	0	0	0	0	0	0	0	0	5	1	0
3	1	?	0	0	0	2	0	0	0	3	0
?	0	0	1	1	0&1	2	0	0	1	0	-
-	0	0	3	1	0	0	1	0	1	0	2&3
1	0	1	1	0	1	0	1	?	0	1	1
2	1	0	0	0	2	1	0	0	1	0	1
2	1	1	0	0	0	-	0	0	0	0	0
0	0	0	0	?	?	2	0	0	-	0	0
-	0	1	0	0	0	0	1	1	1	1	1
0	1	2	0	-	?	?	?	?	1	0	0
3	1	1	?	?	0	0	?	?	?	?	?
?	?	?	?	?	?	?	?	?	?	?	?
?	?	?	?	?	?	?	?	?	?	?	?
?	?	?	?	?	?	?	?	?	?	?	?
?	?	?	?	?	?	?	?	?	?	?	?
?	0	?	0	?	1	3	0	?	0	0	0
0	2	0	0	0	0	0	-	?	0	0	?
0	0	?	?	1	0	0	?	?	1	1	0
0	2	0	0	0	?	0	0	1	0	2	1

1	?	1	-	?	?	0	?	?	0	0	?
0	0	0									

†*Sangiorgioichthys valmarensis*

2	0	0	0	-	1	?	?	?	0	1	0
?	?	?	?	0	?	?	?	?	?	?	?
?	?	?	?	?	?	?	?	?	?	?	?
?	?	?	?	?	?	?	?	?	?	?	?
?	?	?	?	?	?	?	?	?	?	?	?
?	?	?	0	0	?	?	?	?	?	?	?
?	?	?	?	?	?	?	?	?	?	?	?
?	?	?	?	?	0	0	0	?	0	0	?
?	0	0	0	1	1	0	0	0	?	?	?
?	?	?	0	0	0	1&2	0	0	0	3	0
?	0	0	?	1	0	3	0	?	?	?	?
?	0	?	?	?	?	?	?	?	?	?	3
1	0	0	0	0	1	0	?	?	0	1	?
?	1	0	0	0	2	?	?	?	?	?	?
?	?	?	?	?	?	?	?	0	?	?	0
0	0	1	0	?	?	2	0	0	-	0	0
-	0	0	0	0	0	0	1	1	1	1	?
?	1	?	0	-	?	?	?	?	?	?	0
?	0	?	?	?	0	?	?	?	?	?	?
?	?	?	?	?	?	?	?	?	?	?	?
?	?	?	?	?	?	?	?	?	?	?	?
?	?	?	?	?	?	?	?	?	?	?	?
?	?	?	?	?	?	?	?	?	?	?	?
?	?	?	?	?	1	?	?	?	?	?	?
?	?	0	?	0	0	?	-	?	0	?	?
0	?	?	?	?	0	?	?	?	?	1	0
?	?	0	0	0	?	0	0	1	?	2	1
1	?	1	-	?	?	?	?	?	0	0	?
?	0	0									

†*Scheenstia mantelli*

0	0	0	0	-	1	1	1	2	0	0	-
?	?	?	?	0	?	?	?	1	0	?	2
1	?	?	0	?	?	?	?	0	?	?	1
1	0	?	?	0	0	0	0	0	0	1	0
?	2	1	0	2	1	0	?	?	?	?	0
0	?	?	0	0	?	0	?	2	?	?	?
?	?	?	?	?	?	?	?	3	?	?	?
?	?	0	0	0	2	0	0	4	0	0	?
?	0	0	0&2	0&1	1	0	1	0	?	?	?
?	1	?	0	0	1	2	0	1	1	4	0
0	0	1	1	3	0	0	0	0	1	3	?
1	0	?	?	?	?	?	?	?	1	0	3
1	0	0	0	1	0	0	1	1	0	1	2
4	3	?	0	0	1	1	0	0	1	0	1
0	1	?	0	?	1	?	?	0	?	?	?
0	0	1	2	3	?	2	0	0	-	0	0
-	0	0	0	0	0	1	1	1	1	1	1
0	0	?	0	-	?	?	?	?	?	?	0

0	0	?	0	?	?	0	-	1	0	0	?
?	-	?	?	?	?	?	?	?	?	?	?
?	?	?	?	?	?	?	?	?	?	?	?
?	?	?	?	?	?	?	?	?	?	?	?
?	?	?	?	?	?	?	?	?	?	?	?
?	?	?	?	?	?	?	0	?	?	?	?
0	0	0	0	2	0	0	-	?	0	0	?
?	?	?	?	?	0	?	?	?	?	1	1
?	?	0	2	1	2	0	0	1	?	2	?
1	4	?	?	?	?	0	?	?	0	?	?
?	0	?									

†*Scheenstia maximus*

0	0	0	0	-	1	1	1	2	0	1	0
?	?	?	?	0	?	?	?	?	?	?	?
?	?	?	?	?	?	?	?	?	?	?	?
?	?	?	?	?	?	?	?	?	?	?	?
?	?	?	?	?	?	?	?	?	1	1	?
?	?	?	?	0	?	?	?	1	?	?	?
?	?	?	?	?	?	?	?	?	?	?	?
?	?	?	?	?	2	0	0	4	0	0	?
?	0	?	?	?	?	0	1	0	?	?	?
?	1	?	0	0	1	2	0	1	0	4	0
?	0	2	1	3	0	0	0	0	1	3	?
1	0	?	?	?	?	?	?	?	1	0	3
1	0	0	0	1	0	0	?	?	?	?	?
?	?	?	?	?	?	?	?	?	?	?	1
?	?	?	?	?	?	?	?	?	0	0	?
0	0	1	3	3	3	2	0	0	-	0	0
-	0	0	0	0	0	1	1	1	1	1	1
0	?	?	?	?	?	?	?	?	?	?	?
?	?	?	?	?	?	0	?	?	?	?	?
?	?	?	?	?	?	?	?	?	?	?	?
?	?	?	?	?	?	?	?	?	?	?	?
?	?	?	?	?	?	?	?	?	?	?	?
?	?	?	?	?	?	?	?	?	?	?	?
?	1	?	?	?	?	?	0	?	?	?	?
0	0	0	0	2	0	0	-	?	0	0	1
0	2	?	?	0	0	2	?	?	0	1	1
1	?	0	2	1	2	0	0	1	?	2	?
?	?	?	?	?	?	0	?	?	0	0	?
0	0	0									

†*Scheenstia zappi*

0	0	0	0	-	1	?	?	?	0	1	0
?	?	?	?	0	?	?	?	?	?	?	?
?	?	?	?	?	?	?	?	?	?	?	?
?	?	?	?	?	?	?	?	?	0	1	0
?	?	?	?	?	?	?	?	?	?	?	0
0	?	?	0	0	?	?	?	?	0	0	1
-	?	?	?	?	?	?	?	?	?	0	?
?	?	?	?	?	2	0	0	4	0	0	?
?	0	0	0	0	1	0	1	?	1	1	0

3	1	1	0	0	1	2	0	1	0	3	0
?	0	1	1	3	0	2	0	0	1	3	?
1	0	0	3	1	0	0	1	?	1	0	3
1	0	0	0	1	0	0	1	1	0	1	?
?	?	?	?	?	?	?	?	?	1	0	1
0	1	?	0	?	1	?	?	0	?	0	?
0	0	0	2	2	?	2	0	0	-	0	0
-	0	0	0	1	0	1	1	1	?	0	1
0	?	?	0	-	?	?	?	?	1	1	0
0	0	1	0	?	0	?	?	?	?	?	?
?	?	?	?	?	?	?	?	?	?	?	?
?	?	?	?	?	?	?	?	?	?	?	?
?	?	?	?	?	?	?	?	?	?	?	?
?	?	?	?	?	?	?	?	?	?	?	?
?	2	?	?	?	1	?	0	?	?	?	?
0	0	0	0	2	0	0	-	?	0	0	?
0	0	?	?	1	0	?	?	?	?	1	0
0	1	0	2	1	2	0	0	1	1	?	?
1	5	?	?	0	?	0	?	?	?	0	?
0	0	0									
†*Semiolepis brembanus*											
0	0	0	0	-	0&1	0	1	1	0	1	1
?	?	?	?	0	?	?	?	?	?	?	?
?	?	?	?	?	?	?	?	?	?	?	?
?	?	?	?	?	?	?	?	?	?	?	?
?	?	?	?	?	?	?	?	?	?	?	?
?	?	?	0	0	?	?	?	3	0	0	1
-	?	?	?	?	?	?	?	?	?	?	?
?	?	?	?	?	2	0	0	4	0	0	?
?	0	0	2	1	1	0	1	0	1	1	0
?	1	?	0	0	0	0	0	0	0	3	0
?	0	0	?	1	0	0	?	0	1	2	0
0	0	0	3	1	0	0	1	?	1	0	0
-	-	1	0	-	0	?	?	?	0	1	1
4	1	0	0	0	?	?	?	?	1	?	1
?	?	?	?	?	?	?	?	?	?	?	0
0	0	?	?	2	?	2	0	1	-	0	0
-	0	0	0	0	0	4	1	1	2	1	1
1	?	?	0	-	?	?	?	?	?	?	?
?	?	?	?	?	?	0	?	?	?	?	?
?	?	?	?	?	?	?	?	?	?	?	?
?	?	?	?	?	?	?	?	?	?	?	?
?	?	?	?	?	?	?	?	?	?	?	?
?	?	?	?	?	?	?	?	?	?	?	?
?	2	?	?	?	1	3	0	?	2	?	?
0	2	0	0	1	0	0	-	?	0	0	?
0	0	?	?	?	0	?	?	?	?	1	0
0	2	?	?	?	?	?	?	?	?	2	1
1	?	?	?	?	?	?	?	0	0	0	?
0	0	0									
†*Semionotus bergeri*											

0	0	1	0	-	1	0	1	1	0	1	1
?	?	?	?	0	?	?	?	?	?	?	?
?	?	?	?	?	?	?	?	?	?	?	?
?	?	?	?	1	0	?	?	?	?	1	0
?	?	?	?	?	?	?	?	?	?	?	?
?	?	?	0	0	?	?	?	3	?	?	?
?	?	?	?	?	?	?	?	?	?	0	?
?	?	?	?	?	0	0	0	3	0	0	?
?	0	0	0	1	1	0	1	?	?	?	?
?	1	1	0	0	1	1	0	0	0	3	0
0	0	0	2	7	0	0	0	0	1	2	0
0	0	0	?	?	0	0	?	?	?	0	0
-	-	1	0	-	0	0	1	?	0	1	?
?	?	?	?	?	?	?	?	?	?	?	1
?	?	?	?	?	?	?	?	?	?	?	0
0	0	1	0	?	?	2	0	0	-	0	0
-	0	0	0	0	0	0	1	1	1	1	1
0	0	?	0	-	?	?	?	?	?	?	0
?	0	0	?	?	0	0	?	?	?	?	?
?	?	?	?	?	?	?	?	?	?	?	?
?	?	?	?	?	?	?	?	?	?	?	?
?	?	?	?	?	?	?	?	?	?	?	?
?	?	?	?	?	?	?	?	?	?	?	?
?	0	?	?	?	3	?	1	?	?	0	0
0	0	0	0	?	0	?	-	?	0	0	?
0	0	?	?	?	0	?	?	?	?	1	2
?	1	0	2	?	?	0	0	?	?	2	1
1	?	?	?	?	?	?	0	?	0	0	?
0	0	0									

†*Semionotus capensis*

0	0	1	0	-	?	0	1	1	0	1	1
?	?	?	?	0	?	?	?	?	?	?	?
?	?	?	?	?	?	?	?	?	?	?	?
?	?	?	?	1	0	?	?	?	?	1	0
?	?	?	?	?	?	?	?	?	?	?	0
?	1	?	0	0	?	0	0	3	0	0	1
-	0	?	?	?	?	?	?	3	?	0	?
?	?	?	?	?	1	0	0	3	0	0	?
?	0	0	0	1	1	0	1	0	5	1	0
?	1	1	0	0	0	1	0	0	0	3	0
0	0	0	0	7	0	2	?	0	1	2	?
0	0	0	3	1	0	0	?	?	?	0	0
-	-	1	0	-	0	0	1	1	0	1	?
?	?	?	?	?	?	?	?	?	1	0	1
?	?	?	?	?	?	?	?	?	?	0	?
0	0	1	0	?	?	2	0	0	-	0	0
-	0	0	0	0	0	0	1	1	1	1	1
0	0	?	0	-	?	?	?	?	?	?	0
?	0	?	?	?	0	0	?	?	?	?	?
?	?	?	?	?	?	?	?	?	?	?	?
?	?	?	?	?	?	?	?	?	?	?	?

	?	?	?	?	?	?	?	?	?	?	?	?
	?	?	?	?	?	?	?	?	?	?	?	?
	?	0	?	0	?	3	?	1	?	?	0	0
	0	0	0	0	1	0	?	-	?	0	0	1
	0	0	0	1	0	0	0	?	?	?	1	2
	2	0	0	2	1	?	0	0	?	?	2	?
	?	?	?	?	?	?	?	?	?	0	0	?
	0	0	0									
†*Semionotus elegans*												
	0	0	1	0	-	0	0	?	?	0	1	1
	?	?	?	?	0	?	?	?	1	1	?	2
	0	0	-	0	0	1	1	?	1	-	-	?
	?	?	?	?	1	0	?	?	?	?	1	0
	1	?	1	0	2	?	?	1	3	1	0	0
	0	1	?	0	0	?	0	0	2	0	0	1
	-	0	0	?	?	?	0	?	2	1	0	4
	1	?	0	0	0	0	0	0	3	0	0	1
	0	0	1	0	1	1	0	1	0	5	1	0
	3	1	1	0	0	1	1	0	0	0	3	0
	0	0	0	0	6	0	0	0	0	1	1	0
	0	0	0	3	1	0	0	1	0	1	0	0
	-	-	1	0	-	0	0	1	1	0	1	2
	4	1	?	0	0	1	1	0	0	1	0	1
	0	1	1	0	0	1	0	?	0	0	0	0
	0	0	1	0	0	0	2	0	0	-	0	0
	-	0	0	0	0	0	4	1	1	1	1	1
	0	0	3	0	-	?	?	?	1	1	1	0
	?	0	0	0	?	0	0	-	0	0	0	?
	?	-	?	?	?	-	0	?	1	0	?	?
	?	?	?	0	0	0	?	0	0	0	0	?
	?	?	?	?	-	-	?	?	?	?	?	?
	?	?	0	?	0	?	0	0	0	-	-	-
	-	0	?	0	?	3	2	1	3	1	?	?
	0	0	0	0	?	0	?	-	?	0	0	1
	0	0	0	1	?	0	?	?	1	?	1	2
	0	?	0	2	1	?	0	0	?	0	2	1
	1	?	0	1	?	?	0	0	?	0	0	?
	0	0	0									
†*Siemensichthys macrocephalus*												
	2	0	0	0	-	1	0	0	-	0	0	-
	1	?	0	?	1	?	?	0	0	-	1	1
	0	?	?	?	0	?	?	1	0	1	1	0
	0	0	?	?	0	?	0	?	?	0	1	1
	1	1	1	0	?	?	?	0	-	1	1	0
	0	?	?	0	0	?	?	?	0	0	1	-
	?	?	?	?	?	?	1	?	?	?	0	?
	0	?	?	0	?	0	?	0	2	0	?	1
	1	0	1	2	1	1	0	0	0	3	1	1
	1	1	0	?	0	0	3	-	0	?	0	?
	?	?	3	1	0&1	1	2	0	0	0	-	-
	-	0	?	?	?	?	?	1	3	1	1	-

-	-	-	-	-	-	1	0	-	0	1	1
1	1	0	1	0	1	1	0	0	1	0	1
0	1	3	0	0	4	-	?	0	?	?	0
0	?	?	?	?	?	3	0	0	0	1	1
0	0	1	0	?	2	0	1	1	0	0	1
1	?	2	1	2	?	?	?	?	?	?	0
?	0	0	0	2	?	?	-	1	1	0	0
?	-	?	?	?	?	?	?	?	?	?	0
?	?	?	0	0	1	?	1	1	?	?	?
?	?	?	?	?	?	?	?	?	?	?	?
?	?	?	?	?	?	?	?	?	?	?	?
?	?	?	?	?	?	1	?	?	0	?	?
1	?	1	?	?	?	?	?	?	?	?	?
0	?	?	?	?	0	?	?	?	?	1	?
?	?	0	?	?	?	?	?	?	?	2	1
1	?	0	0	?	?	1	?	?	0	0	?
0	2	?									

†*Solnhofenamia elongata*

3	0	1	1	-	-	-	-	-	0	-	-
?	?	?	?	0	?	?	?	?	?	?	?
1	?	?	?	?	?	?	2	0	?	?	1
1	?	?	1	?	0	?	?	?	0	1	0
1	2	?	0	2	?	?	1	1	1	0	?
0	?	1	0	0	0	0	1	0	1	-	-
-	0	0	0	0	-	0	?	2	1	1	0
?	?	?	?	2	0	0	0	2	?	0	?
0	0	1	0	1	2	0	0	0	3	1	0
3	1	0	0	0	1	2	0	2	0	3	0
0	1	2	0&1	1	0	2	0	0	0	-	-
-	0	0	2	1	0	1	1	4	1	0	0
-	-	0	0	-	0	0	1	1	0	1	1
0	1	0	1	1	1	1	0	0	0	0	1
0	1	1	0	?	1	0	0	0	0	0	0
0	0	0	0	0	0	2	0	0	-	0	0
-	0	0	0	0	3	2	1	1	1	1	1
0	1	1	1	1	0	1	?	?	1	1	0
3	0	?	0	1	0	0	0	2	0	0	1
2	-	1	0	0	0	0	1	1	1	0	0
0	?	0	0	0	0	0	0	0	1	1	0
0	0	1	-	0	0	1	1	0	0	3	4
1	2	1	0	0	0	0	0	0	-	-	-
-	1	1	0	0	3	0	?	2	0	0	0
0	2	1	0	-	1	0	2	2	2	-	1
1	2	0	1	1	0	2	1	0	1	1	3
3	0	1	-	0	0	0	0	0	1	1	0
1	1	0	1	0	?	0	?	0	1	0	0
1	0	0									

†*Teoichthys brevipina*

3	0	-	0	-	0	?	?	?	0	0	-
?	?	?	?	0	?	?	?	?	?	?	?
?	?	?	?	?	?	?	?	?	?	?	?

?	?	?	?	?	0	?	0	0	0	?	?
?	?	?	?	?	?	?	?	?	?	?	?
?	?	?	0	0	?	?	?	0	1	-	-
-	?	?	?	?	?	?	?	?	?	1	0
?	?	?	?	?	0	0	0	4	0	0	?
?	0	0	0	1	0	0	1	0	0	1	0
3	1	1	?	0	1	0	0	2	0	3	0
0	1	5	0	6	0	2	0	1	0	-	-
-	0	0	3	1	0	?	1	4	1	0	3
0	2	0	0	0	0	?	?	?	0	1	1
2	1	0	0	0	1	1	0	0	?	0	1
?	0	?	0	?	1	0	?	0	0	0	0
0	0	0	0	?	?	2	0	0	-	0	0
-	0	0	0	0	0	4	?	?	?	?	1
0	?	1	?	?	?	?	?	?	?	?	?
?	0	?	1	?	?	1	0	2	0	0	?
0	-	?	?	?	?	?	?	?	?	?	?
?	?	?	?	?	?	?	?	?	?	?	?
?	?	?	?	?	?	?	?	?	?	?	?
?	?	?	?	?	?	?	?	?	?	?	?
?	0	?	?	?	3	?	1	?	?	?	?
0	2	0	0	2	0	0	-	?	0	0	?
0	0	?	?	?	0	?	?	?	?	1	0
?	?	?	?	?	?	0	0	?	?	1	?
?	?	?	?	0	?	?	?	?	?	0	?
?	0	0									

†*Thaiichthys buddhabutrensis*

0	0	?	0	-	0	?	1	2	0	1	0
?	?	?	?	0	?	?	?	1	0	1	?
1	0	-	0	?	?	?	?	0	?	?	1
1	0	?	?	0	0	0	?	?	?	1	0
1	?	0	?	?	?	-	?	?	1	?	?
?	?	?	0	0	?	?	?	2	0	0	1
-	?	?	?	?	?	?	?	?	1	0	?
1	?	?	0	?	2	0	0	3	0	0	?
?	0	0	1	0	0	0	1	0	1	1	0
3	1	1	1	0	1	2	0	1	1	4	0
0	0	2	1	1	1	0	0	0	1	2	?
0	0	?	?	?	?	?	?	?	?	0	1
0	0	1	0	-	0	0	1	1	0	1	2
4	1	0	0	0	1	0	-	-	1	0	1
?	?	?	?	?	?	?	?	0	1	1	0
0	0	1	0	?	?	3	0	0	1	0	0
-	0	0	0	0	0	4	1	1	2	1	1
1	0	?	0	-	?	?	?	?	1	2	0
0	0	0	0	0	0	?	-	0	0	0	?
?	-	?	?	?	?	?	?	?	?	?	?
?	?	?	?	?	?	?	?	?	?	?	?
?	?	?	?	-	-	?	?	?	?	?	?
?	?	?	?	?	?	?	?	?	?	?	?
?	?	?	?	?	1	2	0	?	1	0	0

	0	0	0	0	?	0	0	-	?	0	0	1
	0	0	0	1	0	0	?	?	?	0	1	0
	0	2	0	2	1	?	0	0	?	?	2	1
	?	4	1	-	0	?	?	?	?	0	0	0
	0	0	0									
†*Tharsis dubius*												
	0	0	1	2	0	-	-	-	-	0	-	-
	1	0	?	?	1	0	-	0	?	?	3	1
	0	?	?	?	1	1	1	2	0	?	?	1
	0	?	5	1	0	0	0	0	1	0	1	2
	2	1	1	0	?	1	0	0	?	1	1	0
	0	1	0	0	0	1	0	0	2	0	1	-
	1	0	1	?	?	?	1	1	4	1	0	2
	0	1	0	0	3	0	0	0	2	1	1	?
	?	0	0	2	1	1	0	0	?	2	2	1
	?	1	0	0	0	0	3	-	0	0	3	0
	0	0	1	1	2	1	2	0	0	0	-	-
	-	0	0	0	0	0	0	2	0	1	1	-
	-	-	-	-	-	-	0	-	-	0	1	0
	2	1	1	0	0	2	2	-	0	1	1	1
	1	0	1	1	1	2	1	1	0	0	0	0
	0	0	1	0	-	?	3	0	0	0	1	0
	-	0	1	0	0	2	4	1	1	1	1	1
	1	0	1	1	2	?	?	?	?	?	?	0
	1	0	0	0	2	0	0	0	1	1	1	0
	-	1	0	0	0	0	0	1	1	1	1	1
	1	0	1	1	1	0	1	1	1	1	1	0
	0	0	0	1	1	0	2	1	0	0	3	3
	1	1	0	0	1	0	0	0	1	1	0	0
	0	0	0	1	1	2	3	?	4	2	1	1
	1	3	1	1	-	2	1	1	1	1	1	1
	0	0	1	1	1	0	0	0	?	1	1	3
	3	0	1	2	0	0	0	0	0	1	3	?
	0	2	1	-	0	0	1	1	0	1	0	2
	1	0	0									
†*Thrissops formosus*												
	4	0	1	2	?	-	-	-	-	0	-	-
	1	2	1	?	1	1	1	?	1	0	?	?
	0	0	-	0	?	?	?	2	0	1	1	1
	0	0	5	1	0	0	0	0	1	0	1	2
	?	1	1	0	0	1	0	0	-	1	1	0
	?	2	1	0	0	0	0	0	2	0	1	-
	1	0	1	?	?	?	1	?	?	1	0	2
	0	?	0	0	3	0	0	0	1	1	-	?
	1	0	?	2	0	1	0	0	0	2	2	?
	?	1	0	0	0	1	3	-	0	0	3	0
	0	0	1	1	1	1	3	0	0	0	-	-
	-	0	0	0	0	0	1	?	?	1	1	-
	-	-	-	-	-	-	0	-	-	0	1	0
	1	1	0&1	0	0	2	2	-	0	1	0	1
	?	0	?	1	1	1	0	1	0	0	0	0

0	0	0	0	-	0	3	0	0	0	1	0
-	0	0	0	0	0	4	1	1	0	1	1
0	0	0	0	-	?	?	?	?	?	?	0
1	0	0	0	1	0	0	0	?	1	1	0
-	1	0	0	0	0	0	0	1	1	1	1
0	-	0	0	0	0	0&1	0	0	0	0	0
0	0	0	1	0	0	2	1	0	0	3	3
1	1	0	0&1	0	0	1	0	1	0	1	0
0	0	0	1	1	2	1	?	3	0	1	1
1	2	1	0	-	2	1	1	1	2	-	1
0	1	1	1	1	1	1	2	1	1	1	3
3	0	1	-	0	0	0	0	0	1	3	?
0	1	1	-	0	1	1	?	0	1	0	?
1	?	0									
†*Ticinolepis crassidens*											
0	0	0	0	-	0	0	0	-	1	0	-
?	?	?	?	0	?	?	?	1	0	?	0
?	?	?	?	?	?	?	?	0	0	-	0
0	0	3	1	0	0	0	0	0	0	1	0
?	?	1	0	0	0	?	?	?	1	?	?
?	1	-	0	0	?	0	0	2	0	0	1
-	0	?	?	?	?	?	?	0	?	?	?
?	?	?	0	?	0	0	0	?	0	0	?
?	0	?	?	?	?	?	0	0	0	0	0
3	1	?	0	0	0	1	0	0	0	3	0
0	0	0	2	5	0	2	0	0	1	1	0
0	0	0	3	1	0	0	1	0	1	0	3
1	2	0	0	0	0	0	?	?	0	1	1
3	1	0	0	0	3	1	0	0	1	0	1
?	?	?	?	?	?	?	?	0	?	2	0
0	0	0	2	3	3	2	0	0	-	0	0
-	0	0	0	0	0	0	1	1	1	1	1
0	1	3	0	-	?	?	?	?	?	?	?
?	?	?	0	?	0	0	?	?	?	?	?
?	?	?	?	?	?	?	?	?	?	?	?
?	?	?	?	?	?	1	?	?	?	?	?
?	?	?	?	?	?	?	?	?	?	?	?
?	?	?	?	?	?	?	?	?	?	?	?
?	0	?	?	?	2	1	0	?	0	1	0
0	2	0	0	1	0	0	-	?	0	0	?
0	?	?	?	?	0	?	?	?	?	1	0
0	0	0	0	0	0	0	1	1	1	2	1
1	?	?	?	?	?	0	?	?	0	0	?
0	0	0									
†*Ticinolepis longaeva*											
0	0	0	0	-	1	0	0	-	1	0	-
?	?	?	?	0	?	?	?	1	0	?	?
?	?	?	?	?	?	?	?	?	?	?	?
?	0	?	?	0	0	?	0	0	?	1	0
1	?	1	0	?	?	?	0	-	1	0	0
?	1	1	0	0	?	?	?	2	0	0	1

-	0	0	?	?	?	?	?	0	1	0	1
1	?	0	0	1	0	0	0	1	0	0	1
0	0	1	2	0	0	1	0	0	0	0	0
3	1	0	0	0	0	2	0	0	0	3	0
0	0	0	0&1	5	0	2	0	0	1	1	0
0	0	0	3	1	0	0	1	0	1	0	3
1	2	0	0	0	0	0	0	0	0	1	1
3	1	0	0	0	3	1	0	0	1	0	1
0	1	1	0	0	1	0	0	0	0	2	0
0	0	0	1	1	1	2	0	0	-	0	0
-	0	0	0	0	0	0	1	1	1	1	1
0	1	3	0	-	?	?	?	?	1	1	1
0	?	?	0	?	0	0	?	?	?	?	?
?	?	?	?	?	?	?	?	?	?	?	?
?	?	?	?	?	?	?	?	?	?	?	?
?	?	?	?	?	?	?	?	?	?	?	?
?	?	?	?	?	?	?	?	?	?	?	?
?	0	?	?	0	1	1	0	?	0	1	0
0	2	0	0	1	0	0	-	?	0	0	?
0	0	?	?	1	0	0	?	?	1	1	0
0	2	0	0	0	0	0	1	1	0	2	1
1	4	1	-	?	?	0	0	0	0	0	0
0	0	0									

†*Tlayuamichin itztli*

0	0	0	0	-	2	0	1	2	0	1	0
?	?	?	?	0	?	?	?	?	?	?	?
?	?	?	?	?	?	?	?	?	?	?	?
?	?	?	?	1	0	?	?	?	?	1	0
?	1	1	?	?	?	?	0	-	1	0	?
0	1	0	0	0	1	0	0	4	0	0	1
-	0	?	?	?	?	?	?	3	?	0	?
?	?	?	?	?	1&2	0	0	4	0	0	?
?	0	0	0	1	1	0	1	?	5	1	0
?	1	1	0	0	0	0	0	0	2	3	0
0	0	0	2	7	0	0	0	0	1	6	2
0	0	0	3	1	0	0	1	?	1	0	2
0	0	0	1	0	0	0	1	1	0	1	1
4	3	0	0	0	1	1	0	0	1	0	1
0	1	?	0	?	1	1	?	0	0	2	1
0	0	0	2	?	2	2	0	1	-	0	0
-	0	1	0	0	0	1	1	1	3	1	1
0	1	3	0	-	?	?	?	?	?	?	0
3	0	1	0	?	0	0	?	?	?	?	?
?	?	?	?	?	?	?	?	?	?	?	?
?	?	?	?	?	?	?	?	?	?	?	?
?	?	?	?	?	?	?	?	?	?	?	?
?	?	?	?	?	?	?	?	?	?	?	?
?	?	?	?	?	3	3	1	?	2	?	?
0	0	0	0	1	0	0	-	?	0	0	?
0	?	?	?	0	0	?	?	?	?	1	2
1	2	0	2	1	1	0	0	1	?	2	?

1	4	?	?	?	?	0	?	0	0	0	?
0	0	0									

†*Varasichthys ariasi*

0	0	1	2	1	-	-	-	-	0	-	-
?	?	?	?	1	0	-	?	0	-	3	2
0	?	?	0	1	?	?	2	0	?	?	?
0	0	?	?	0	?	0	0	1	?	?	?
?	3	?	0	0	1	?	0	-	?	?	?
?	?	?	0	0	?	?	?	1	0	1	-
?	?	1	?	?	?	1	?	?	1	0	2
0	0	?	?	?	0	2	0	3	1	?	?
?	0	0	2	?	?	0	0	0	2	2	?
?	1	?	0	0	0	3	-	0	-	?	?
?	?	1	1	8	1	2	0	0	0	-	-
-	0	0	0	0	0	0	?	?	1	0	0
-	-	0	0	-	0	0	-	-	0	1	0
1	1	1	0	0	?	2	-	0	1	1	1
1	0	3	0	1	1	0	1	0	?	0	0
0	?	1	0	-	?	3	0	0	0	1	0
-	0	0	0	0	2	4	?	?	?	1	1
0	0	1	1	2	?	?	?	?	?	?	0
0	0	0	0	2	0	?	0	1	1	1	?
-	1	?	0	?	0	0	?	?	?	?	1
1	0	1	0	0	0	0	1	1	0	0	?
?	?	0	0	0	0	2	1	0	0	3	?
1	3	0	0	0	0	1	0	1	1	0	0
0	0	0	?	1	1	3	?	3	2	?	1
1	2	1	1	-	2	1	?	?	1	1	1
1	1	1	1	?	1	1	0	1	1	1	3
?	?	1	2	?	?	?	0	0	2	3	?
?	5	?	?	0	0	0	?	0	1	1	2
1	0	1									

†*Vidalamia catalunica*

3	0	0	1	-	-	-	-	-	0	-	-
?	?	?	?	0	?	?	?	?	?	?	?
?	1	0	0	?	?	?	?	?	?	?	1
?	?	?	1	1	0	?	0	0	0	?	?
1	2	0	0	?	?	-	1	1	?	?	?
0	?	1	0	0	?	?	?	0	1	-	-
0	?	?	?	?	?	0	?	2	1	1	0
1	?	0	0	?	0	0	0	1	0	0	?
?	0	0	0	1	2	1	0	0	?	1	0
?	1	0	0	0	?	?	0	0	?	3	0
0	1	3	0	2	?	2	?	0	0	-	-
-	0	0	?	?	0	0	1	4	1	1	-
-	-	-	-	-	-	0	?	?	0	1	1
?	1	0	1	2	1	1	1	0	0	0	1
0	1	?	0	0	1	0	0	0	0	0	0
0	0	0	4	0	0	2	0	0	-	0	0
-	0	0	0	0	3	2	1	?	?	1	1
?	1	1	1	0	?	1	?	?	1	0	1

?	0	?	0	1	0	0	0	2	0	0	1
2	-	0	1	0	0	0	1	?	?	?	0
0	-	0	0	0	0	0	0	0	1	1	0
0	0	0	0	0	0	0	0	0	?	3	3
1	3	1	0	0	0	0	0	0	-	-	?
-	1	1	0	0	3	0	?	2	0	0	0
0	2	1	0	-	1	0	2	?	2	?	1
1	2	0	1	1	0	?	0	?	?	1	3
?	0	1	-	0	0	1	?	?	?	1	0
1	?	1	-	0	?	0	0	?	1	0	1
1	0	0									
†*Watsonulus eugnathoides*											
1	0	0	0	-	1	0	?	?	0	0	-
1	3	0	?	0	-	-	?	0	-	?	0
0	0	-	0	0	1	0	?	0	0	-	0
?	?	?	1	1	0	0	0	0	0	1	0
1	1	0	0	0	0	0	1	1	1	0	0
0	?	1	0	0	0	0	1	0	0	0	0
-	?	0	?	?	?	0	?	2	?	1	0
1	0	0	0	0	0	0	0	0	0	0	?
0	0	1	2	0	1	1	0	0	0	1	0
?	1	0	0	0	1	2	0	0	0	3	0
0	0	0	0	8	0	2	0	0	0	-	-
-	0	0	2	1	0	?	1	0	1	0	-
-	-	-	-	-	-	0	0	0	0	1	1
0	1	0	1	1	2	1	0	0	1	0	1
0	1	1	0	0	1	0	0	0	0	0	0
0	0	0	0	0	0	1	0	-	-	0	0
-	0	0	0	0	0	?	1	2	?	0	1
0	1	2	1	0	?	?	?	?	1	0	0
0	0	?	1	?	0	0	-	0	0	0	-
-	-	?	?	?	?	?	?	1	?	?	?
?	?	?	?	?	?	?	?	?	?	?	?
?	?	?	?	-	-	?	?	?	?	?	?
?	?	?	?	0	?	0	?	?	?	?	?
?	0	?	?	?	1	?	0	?	0	?	0
0	2	0	0	?	0	?	-	?	0	0	1
0	?	?	?	?	0	?	?	?	?	1	0
0	?	0	0	0	?	0	1	?	?	2	1
1	3	0	0	0	?	?	?	?	0	0	?
0	0	0									
