# Supplementary material for: Neopterygian phylogeny: the merger assay
Source: R Soc Open Sci. 2018 Mar 21;5(3):172337. doi: 10.1098/rsos.172337 (PMC5882744; doi:10.1098/rsos.172337)
Supplement: Characters [file rsos172337supp2.pdf]

Supplementary File S2: List of phenotypic characters to

Neopterygian Phylogeny: the merger assay

Adriana López-Arbarello[1]*, and Emilia Sferco[2]

[1]Department of Earth- and Environmental Sciences, Palaeontology and Geobiology and GeoBio-Center, Ludwig Maximilian University, Richard-Wagner-Strasse 10, D-80333 Munich, Germany (a.Lopez-Arbarello@lrz.uni-muenchen.de)

[2]CICTERRA-CONICET-UNC. Av. Velez Sarsfield 1611, X0516GCA, Córdoba, Argentina.

The 339 characters listed below have been used for the cladistic analysis to explore the phylogenetic relationships of the Middle Triassic genus †*Ticinolepis*. Among them, 76 characters are new (indicated in blue), but the remaining characters have been emended from previous authors. In most cases, corrections were necessary because the original characters have been proposed either for ginglymodians, halecomorphs, or teleosts and they thus failed to represent the complete morphological disparity encompassed in our data matrix. For this reason, although the emended characters refer to the same trait, many of the newly defined character states are incompatible with those originally included by the source authors.

Many character definitions have been corrected to avoid problematic coding as discussed by Jenner (1) and Brazeau (2). In many cases, tracing back the author who first discussed a certain morphological feature for taxonomic/systematic comparisons is a hopeless task and we might unfairly end up acknowledging the wrong source. Therefore, the cited references correspond to the sources we have used for the corresponding characters, which is not necessarily the first mention of the systematic value of the morphological trait. We thus limit our references to the sources from which a given character coding was taken and refer the readers to the literature cited therein. Several characters are here proposed for the first time in a cladistic analysis, but not all of them are being used for the first time in actinopterygian systematics. In these and many other cases, additional literature is cited in the discussion.

As explained in the Methods section of the manuscript, autapomorphic character states are not deleted because such states, though not informative for the tree search, are informative concerning the amount of homoplasy.

Abbreviations: App., appendix; ch., character; chs., characters; fig., figure; figs., figures; p., page; pl. plate.

General features of the body and squamation

1. Relative position of the dorsal fin respect to the pelvic and anal fins
- (0) originating posterior to insertion of pelvic and extending backwards not beyond middle of anal fin
- (1) originating approximately at the level of the origin of the anal fin and extending opposite to it
- (2) extending anterior to opposite of insertion of pelvic fins
- (3) originating anterior to insertion of pelvic and extending opposite to anal fins
- (4) originating posterior to the origin of anal fin
- (5) originating posterior to insertion of pelvic and extending backwards up to end of anal fin

As presented here, this character is modified from López-Arbarello ((3): ch. 1). Character state 5 was added by Thies and Waschkewitz ((4): ch. 1) to represent the condition in the species of †*Dapedium*.

López-Arbarello (3) scores character state 1 "dorsal fin opposite to anal fin" for the gars, but the dorsal fin in living gars is placed a little backwards respect to the anal fin. Therefore, we now added character state 4 to represent this condition more accurately.

2. Elongation of the rostral region anterior to the lower jaw symphysis
   (0) does not extend anterior to the dentary symphysis significantly
   (1) extends well anterior to the dentary symphysis by more than 50% of mandibular length

This character is taken from Grande ((5): ch. 4) to distinguish the remarkable condition of the head of †obaichthyid gars.

We are aware that this character encompasses some variability from normal prognathism to hypognathism within character state 0. However, the condition in †obaichthyids, which is also found in most species of †*Aspidorhynchus* (6, 7) is so divergent from most other actinopterygians that it blunts the comparably not significant degree of variation included in the other character state. For this reason, we do not include the third character state "does not extend anteriorly beyond the lower jaw symphysis" proposed by Thies and Waschkewitz ((4): ch. 9), which falls within the range of variation accepted for our character state 0.

3. Ornamentation of the dermal bones of the skull
   (0) ornamented with tubercles or ridges
   (1) smooth or very slightly ornamented
   (2) ornamented with sharp tubercles resembling conical teeth

This character is taken from López-Arbarello ((3): ch. 19) and summarizes the main three types of ornamentation recognized for most actinopterygians. The variation from tubercles to ridges is usually present within a single bone and whether one or the other type of ornamentation is dominant varies between regions of the same skull and between individuals of the same species. It is thus impossible to distinguish between tubercles and ridges in different character states. The very distinct kind of ornamentation described in our character state 2 is found in obaichthyid gars and in †*Pliodetes* among the taxa included in our data matrix. It has been compared with the dermal denticles in the clupeomorph teleost *Denticeps*, though these might not be homologous with the odontodes of other vertebrates (8). Resembling the case of the question of homology among the different types of scales (see main text), histological studies are needed to propose more robust hypotheses of primary homology for the different ornamental structures the skull bones of actinopterygians.

Grande and Bemis ((9): ch. 8) is equivalent to our character states 0 and 1, without distinguishing our state 2, which is not present in their data set. Grande ((5): ch. 2) is equivalent to the presence/absence of the condition described in our character state 2 and, thus, implies an unspecified absence (1).

4. Type of scales
   (0) ganoid
   (1) elasmoid of amioid type
   (2) elasmoid of cycloid type

From Brito ((6): ch. 38).

5. Shape of posterior margin of cycloid scales
   (0) smooth
   (1) crenulated

From Sferco et al. ((10): ch. 176). Among basal teleosts, the posterior margin of the cycloid scales is generally smooth. However, in the Late Jurassic varasichthyids, in †*Pachythrissops*, basal elopomorphs (e.g. *Elops*), and a few fossil euteleosts (e.g. †*Leptolepides sprattiformis*) the posterior margins of the scales are crenulated.

6. Posterior margin of ganoid anterior flank scales
   (0) Smooth (Fig. 1C, G)
   (1) serrate or dentate (Fig. 1B, D, E, H)

(2) spiny, with a few small spines (Fig. 1A, F)

Modified from Arratia ((11): ch. 159) by adding character state 2 to represent the conditions found in several ginglymodian taxa. The characteristic of the scales with a strong posteriorly directed spine was proposed in Grande ((5): ch. 38) as an independent a/p character, which involves an unspecified absence state.

7. Vertical peg-and-socket articulation

(0) well developed (Fig. 1A–D, F)
(1) reduced (Fig. 1E, G–H)

From Gardiner and Schaeffer ((12): ch. 3). Xu et al. (13) erroneously score the presence of peg-and-socket articulation in †*Leptolepis coryphaenoides*, which not possible because this taxon has cycloid scales.

8. Longitudinal articulation of the scales of the body

(0) absent (Fig. 1A–B)
(1) present (Fig. 1C–H)

9. Mode of longitudinal articulation of the scales

(0) small anterodorsal process (Fig. 1C)
(1) strong anterior dorsal process (Fig. 1D, G–H)
(2) double, anterior dorsal and ventral processes (Fig. 1E–F)

This character is modified from López-Arbarello ((3): ch. 86) by splitting her character state 1 "only a strong dorsal anterior peg for the longitudinal articulation", to distinguish it from the presence of a small anterodorsal process in some of the studied taxa (†*Australosomus*, †*Archaeosemionotus*, †*Boreosomus*, †*Obaichthys*, †*Ophiopsis*, †*Panxianichthys*, †*Plesiofuro*).

10. Squamation of the ventrum

(0) not significantly different from the rest of the body
(1) numerous horizontal rows of distinctly shallow scales (Fig. S2-1)

First notice among halecomorphs (14), the rhomboid scales covering the ventrum are notably shallower and clearly distinct from the other scales covering the rest of the body in several of the studied taxa (†*Australosomus*, †*Archaeosemionotus*, †*Aspidorhynchus*, †*Ophiopsiella*, †*Ophiopsis*, †*Ticinolepis*).

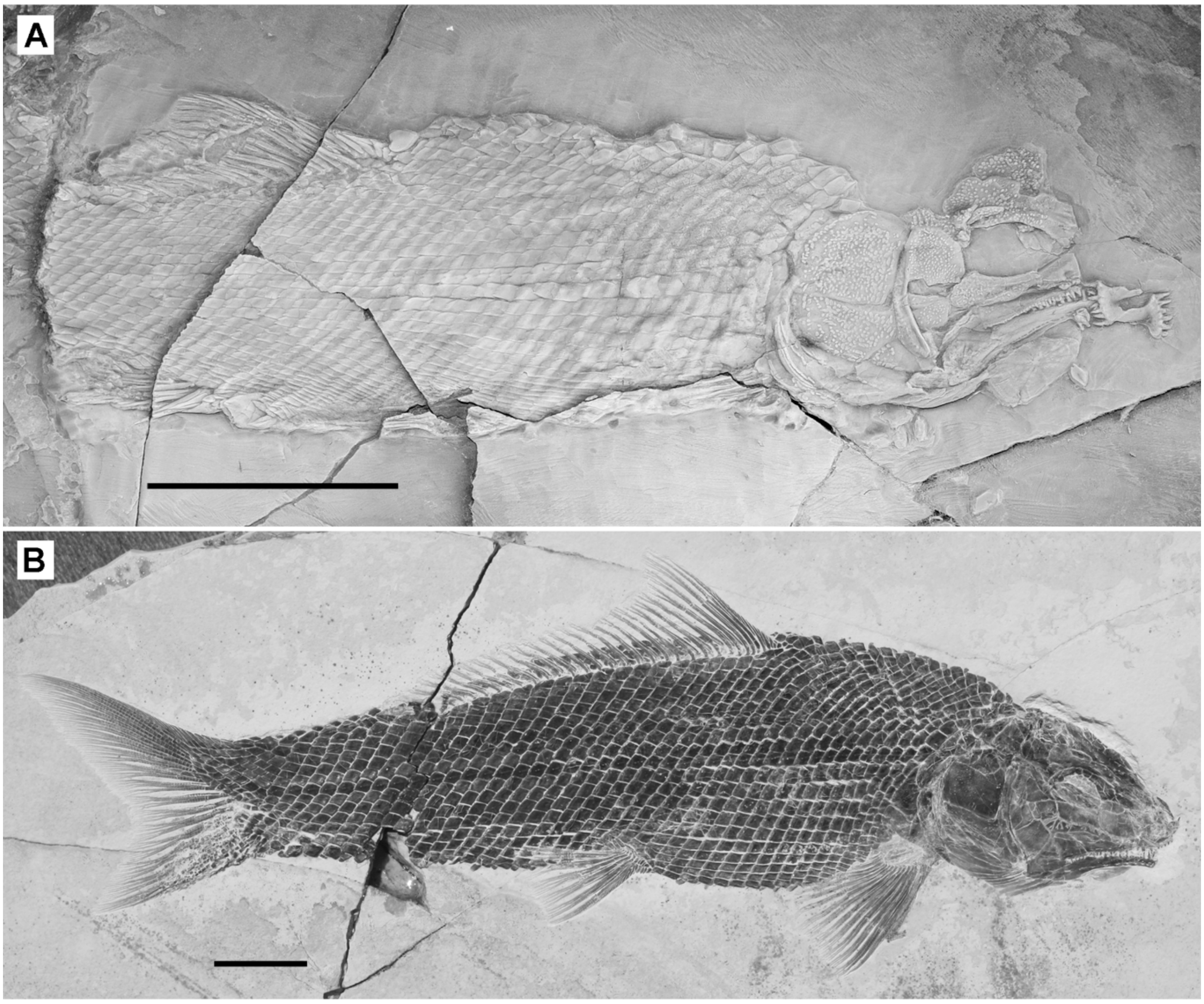

Figure S2-1: Ventral squamation in A, †*Archaeosemionotus connectens* (PIMUZ A/I 552 dusted with ammonium chloride) and B, †*Ophiopsiella attenuata* (JME ETT.1896). Scale bars = 2 cm.

11. Dorsal ridge of scales between the skull and the dorsal fin
- (0) absent
- (1) present

12. Shape of the dorsal ridge scales
- (0) with a low spine
- (1) with a high spine

The presence of a dorsal ridge of scales was proposed by Olsen and McCune ((15): ch. 17) as a distinct feature for "semionotids". López-Arbarello ((3): ch. 83) added the variation in the shape of the dorsal ridge scales forming a multistate character, which we here split in the characters 11 and 12 following the recommendations of Brazeau (2).

Variations in the shape of dorsal ridge scales of several species of †*Semionotus* are thoroughly analysed by McCune (16), but these are all different morphologies of scales with high spines thus corresponding to our character state "with a high spine".

Endocranium, parasphenoid and vomer

13. Fossa parampullaris of Bjerring (17)
- (0) absent
- (1) present

Defined by Bjerring ((17): p. 234) the fossa parampullaris is a depression in the endocranium of the Devonian coelacanthiform †*Nesides schmidti* "situated lateral to the ampulla of the posterior semicircular duct and dorsal to the posterior part of the lateral semicircular duct", and it is placed "dorsal to the parampullar process". According to this author, the depression identified as the 'post-temporal fossa' by Patterson (18) in †*Dorsetichthys bechei* is not homologous with the 'Temporalgrube' of Sagemehl ((19): p. 200, fig. 5). Instead, based on its topographic relationships and the presence of a foramen for the supratemporal nerve of the lateralis system, this depression is homologous with the fossa parampullaris observed in †*Nesides schmidti*. As indicated above, we refer the readers to the work of Bjerring (17) for a more complete explanation of his hypotheses of homology.

14. Bones participating in the posterior end of the fossa parampullaris of Bjerring (17)
- (0) intercalar
- (1) exoccipital
- (2) autopterotic
- (3) epioccipital

Sferco et al. ((10): ch. 12) is a multistate character coding the bones surrounding the posttemporal fossa. We here distinguish between the bones involved in the posterior end of the fossa parampullaris (this character) from those roofing this portion of the fossa (next character), which according to Bjerring (17) hypotheses of homology should be treated independently. The fossa parampullaris is an endocranial depression, the development of the bones that ossify in the fossa is independent from the bones that might growth covering it.

In our data matrix, character states 1 and 3 (posterior end of the fossa parampullaris in the exoccipital or epioccipital) are autapomorphic for †*Oshunia brevis* and *Salmo salar*, respectively.

15. Bones roofing of the posterior opening of the fossa parampullaris of Bjerring (17)
- (0) dermopterotic (or dermopterotic portion of pterotic)
- (1) epioccipital
- (2) parietal
- (3) epioccipital and pterotic
- (4) extrascapular and dermopterotic

Character state 2 is autapomorphic of †*Thrissops formosus* in our data matrix.

16. Spiracular canal
- (0) present
- (1) absent

Taken from Gardiner and Schaeffer ((12): ch. 32). Arratia ((20): ch. 14 App. 3), which includes a "greatly reduced" intermediate state. However, this intermediate state is only scored in †*Leptolepis coryphaenoides* in Arratia (20, 21), or in †*L. coryphaenoides* and †*Tharsis dubius* in Arratia (22), or in †*L. coryphaenoides* and is polymorphic (greatly reduced or absent) in †*Tharsis dubius* in Arratia (11, 23-26) and Arratia and Tischlinger (27). The intraspecific variation in †*Tharsis dubius* indicates that the intermediate condition is probably due to ontogenetic variation and, thus, pending a more detailed study of this issue, we excluded the intermediate character state and scored both †*L. coryphaenoides* and †*Tharsis dubius* with missing data (?).

17. Supraoccipital bone
- (0) absent
- (1) present

From Arratia ((21): ch. 5).

18. Supraoccipital crest
- (0) absent or poorly developed
- (1) present, well developed

19. Shape of the supraoccipital crest
(0) spine-like
(1) high and triangular

The supraoccipital is a small median bone with a poorly developed crest in Triassic and Jurassic teleosts and in certain teleocephalan groups, like clupeomorphs, elopomorphs, euteleosts, and most osteoglossomorphs (10, 11). A well-developed, spine-like supraoccipital crest (state 1) is present in most ostariophysans (e.g. *Brycon* (28)) and in the Cretaceous †*Notelops* and †*Rhacolepis* (29, 30). A high and triangular supraoccipital crest has appeared independently in *Hiodon* (31) and the †Ichthyodectoidei, which are derived ichthyodectiforms (e.g. †*Thrissops*, †*Cladocyclus*), although it is absent in other ichthyodectiform taxa, like †*Allothrissops* (32, 33).

Previous authors have proposed different characters representing the absence/presence, relative size and shape of the supraoccipital crest ((30): chs. 6, 17, (34): ch. 3, (35): ch. 2, (33): chs. 9, 33, 35, 47, (36): ch. 2, (37): ch. 13, (10): ch. 6). Among them, Sferco et al. ((10): ch. 6) gives the optimal representation of the variation observed in the taxa included in our data matrix. However, to avoid pseudo-ordering (2), we distinguish the presence of well-developed supraoccipital crest from its different morphologies in two different characters.

Although our character state 19(0), supraoccipital crest "absent or poorly developed" technically implies an unspecified absence (1), the distinction between the complete absence of the crest and the presence of a poorly developed crest in fossils is usually not possible in the praxis, because in most cases only a well-developed supraoccipital crest is discernible.

20. Supraotic bone
(0) absent
(1) present

From Gardiner et al. ((38): ch. 7 App. 2), but a thorough analysis of the homologies of the supraotic bone and its distribution is done by Maisey (39).

21. Posterior process of epioccipital
(0) absent
(1) present

22. Development of posterior process of epioccipital
(0) small
(1) large

The "presence of a large posteriorly directed process on the "epiotic" …" was proposed by Olsen and McCune ((15): p. 270) as a diagnostic character for their family "Semionotidae" including †*Semionotus* and †*Lepidotes*. Current research (3, 40) indicates that these two genera do not form a monophyletic group and this feature has a much broader distribution among neopterygians. A large epiotic process is found in the semionotiforms †*Semionotus elegans* and †*Macrosemimimus toombsi* ((18): figs. 108–111), the lepisosteiform †*Araripelepidotes temnurus*, and in †"*Lepidotes*" *gloriae* ((41): fig. 6) among ginglymodians, but it is absent in other ginglymodians. A similar process is present in other actinopterygians (e.g. *Polypterus ornatipinnis* (5): fig. 541). A small and blunt process is present in all ginglymodians in which the feature is known, but it is also present in some halecomorphs and teleosts.

23. Foramen for the glossopharyngeal nerve (IX) placed in
(0) jugular depression
(1) prootic
(2) in cartilage, bordered by the prootic anteriorly and dorsally
(3) exoccipital
(4) intercalar
(5) opisthotic

Modified from Sferco et al. ((10): ch. 13). We added character states 0, 2, 4 and 5 to represent the condition observed in the taxa included in our analysis, and excluded their character state 2 (IX-foramen in the basioccipital bone) because it is not represented in our data matrix.

24. Exit of the vagus nerve (X) placed
- (0) through the fissura otico-occipitalis
- (1) between intercalar and exoccipital
- (2) in exoccipital
- (3) between intercalar and opisthotic

From Sferco et al. ((10): ch. 14), though their character states 2 and 3 (exit of the vagus nerve in the basioccipital or between the intercalar and basioccipital, respectively) are not included because they do not occur among the taxa included in this analysis.

25. Posterior extent of exoccipitals in adult-sized individuals
- (0) reach posterior margin of occiput
- (1) do not reach posterior margin of occiput

From Grande and Bemis ((9): ch. 2).

26. Exoccipital forming concave articular facets
- (0) absent
- (1) present

In halecomorphs and in the teleosts †*Dorsetichthys bechei* ((18): fig. 55) and †*Ichthyokentema purbeckensis* ((18): fig. 151), the posterior end of the exoccipital bones form two concave articular facets, which in *Amia calva* articulate with the neural arch of the first vertebral centrum fused to the basioccipital ((9): figs. 25, 37).

27. Exoccipital articular facets
- (0) separated from basioccipital
- (1) attached to basioccipital

The articular facets of the exoccipitals are clearly separated from the basioccipital in the halecomorphs (e.g. (9): figs. 25, 37; (39): fig. 1), but they are tightly attached to this bone in the teleosts †*Dorsetichthys bechei* ((18): fig. 55) and †*Ichthyokentema purbeckensis* ((18): fig. 151).

28. Exoccipital position relative to basioccipital
- (0) dorsal
- (1) anterodorsal
- (2) anterior

The exoccipitals lay on the dorsal surface of the basioccipital in most taxa examined (e.g. (18): figs. 46, 95, 99, 108; (9): fig. 37). Within Ginglymodi however, the exoccipitals are placed anterodorsal (Lepidosteidae) or anterior (†Obaichthyidae and †*Araripelepidotes temnurus*) to the basioccipital ((5): 41, 237, 476, 492–493; (42): figs. 1, 4). †*Rhacolepis* is the only teleost in our data matrix, in which the exoccipital is not dorsal, but anterodorsal to the basioccipital ((29): fig. 13).

29. Foramina in basioccipital for occipital or spinal arteries
- (0) present
- (1) absent

From Sferco et al. ((10): ch. 15).

30. Posterior myodome
- (0) absent
- (1) present

From Gardiner and Schaeffer ((12): ch. 10). Other authors (e.g. (38): ch. 6 App. 1, (43): ch. 12) include the absence of the posterior myodome within multistate characters dealing with different characteristics of the posterior myodome.

31. Relative size of posterior myodome
- (0) not extending into basioccipital
- (1) extending into basioccipital

Modified from Brito ((6): ch. 4). These authors include the absence of a posterior myodome within this character, which is here coded as an independent character (ch. 30) following the recommendations of Brazeau (2).

32. Trajectory of the dorsal aorta in relation to the basioccipital
- (0) passing through an ossified canal formed by the basioccipital
- (1) passing through a median groove in the basioccipital
- (2) canal or groove in the surface of the basioccipital absent

Taken from Hurley et al. ((43): ch. 16) (see discussion in (10): ch. 16), which is modified from Patterson and Rosen ((32): ch. 27).

33. Intercalar
- (0) present
- (1) absent

From Hurley et al. ((43): ch. 7).

34. Dermal component of intercalar
- (0) absent
- (1) present

35. Extent outgrowths of dermal component of intercalar
- (0) without extensive outgrowths (Fig. 2A)
- (1) with outgrowths contacting prootic (Fig. 2B)
- (2) with outgrowths contacting prootic and parasphenoid (Fig. 2C)

36. Opisthotic
- (0) present
- (1) absent

From Olsen and McCune ((15): ch. 5).

37. Pterotic
- (0) present
- (1) absent

Taken from Gardiner et al. ((38): ch. 13 App. 2). Gardiner et al. ((38): ch. 2 App. 1) is a multistate character including the fusion of the pterotic with the dermopterotic in addition to the presence/absence of the pterotic. We agree with Hurley et al. (43) on that the presence of the pterotic and its fusion with the dermopterotic are two independent characters, but evaluating this fusion is very difficult in most fossils so we did not include Hurley et al. ((43): ch. 4).

Grande and Bemis (9) score the presence of a pterotic in †*Oshunia*, but according to Maisey ((39): p. 24) the pterotic is absent in this taxon and the bone interpreted as the pterotic by other authors is the opisthotic. Similarly, according to Rayner (44), Jollie (45), Grande (5) a pterotic is absent in the gars and the bone interpreted as the pterotic by Patterson (18) and Wiley (46) represents the epiotic.

38. Size of prootic
- (0) large, well expanded above the VII-foramen (Fig. 2C)
- (1) small, the dorsal border is close to the VII-foramen (Fig. 2B)

The prootic normally forms most of the anterior half of the lateral wall of the endocranium (state 0), but in *Amia calva* and other amiiforms, the prootic is limited to the anteroventral quarter of the lateral wall of the braincase.

39. Hyomandibular facet formed in

(0) cartilage
(1) the opisthotic
(2) the sphenotic, pterotic, prootic and opisthotic
(3) the sphenotic, prootic and pterotic
(4) the pterotic
(5) the sphenotic and pterotic
(6) the ventral surface of the dermopterotic

40. Orientation of hyomandibular facet respect to the parasphenoid axis

(0) forming an angle of more than 60°
(1) forming an angle of less than 50°

The character states are defined according to the measurements of the angle between the main axis of the hyomandibular facet and the longitudinal axes of the orbital portion of the parasphenoid in 26 taxa (Fig. 3). Among the taxa examined, there is a gradual change for angles below 50° followed by a significant gap between 50° and 60°. Our character states are thus defined around this gap.

41. Sphenotic with small dermal component

(0) absent
(1) present

This feature is explained by Bartram (47) and used in several cladistic analyses [e.g. (5): ch. 23, (3): ch. 7, (48): ch. 16, (11): ch. 10, (49): ch. 34, (50): ch. 75).

42. Sphenotic fused to dermosphenotic

(0) absent, the two bones are separate
(1) present

Among the taxa studied, the dermosphenotic is fused to the sphenotic only in †obaichthyid gars (5). This character is related, though independent from character 121 because the fusion between the bones is not possible in those taxa in which the dermosphenotic and sphenotic are not in contact with each other (*Atractosteus*, *Lepisosteus* and †*Masillosteus*). Thus, character 42 is logically inapplicable for those taxa.

43. Basisphenoid

(0) present
(1) absent

From Wiley ((46): ch. 17).

44. Orbitosphenoid

(0) median
(1) paired

In most actinopterygians the orbitosphenoid is a median bone, usually bifurcating posteriorly to articulate with the paired pterosphenoids. In *Amia calva* and †*Cyclurus kehreri*, the orbitosphenoid is paired (9).

45. Ventral extension of orbitosphenoid

(0) reaching the parasphenoid
(1) confined to the dorsal portion of the orbit

Modified from Arratia ((20): ch. 9 App. 3).

46. Lateral ethmoid ossifications

(0) present
(1) absent

The lateral ethmoid ossifications are widely distributed among actinopterygians (18). In the outgroup taxa the ethmoid regions consists of a median nasal septum and the planum antorbitale (51), which separates the orbit and nasal cavities. In agreement with Patterson ((18): p. 35), we think that the lateral ethmoids are homologous to at least the lateral region of the planum antorbitale. A fully ossified ethmoidal region with a planum antorbitale is also present in †*Watsonulus eugnathoides*.

The ethmoidal ossifications are absent in the gars and macrosemiids, but at least the lateral ethmoids are present in †*Araripelepidotes temnurus* (42), †*Lepidotes semiserratus* (52), †*Scheenstia mantelli* (18) and †*Scheenstia zappi* (53). Ethmoidal ossifications are also reduced in macrosemiids ((54), ALA pers. obs.).

47. Lateral dermethmoid ossifications

(0) absent
(1) present

48. Lateral dermethmoids relationship to other bones

(0) forming the nasal processes of a single paired premaxilla (Fig. 4A, B)
(1) forming toothed dermethmoids (Fig. 4C)
(2) forming part of a compound mesethmoid with chondral and dermal components (Fig. 4D)

López-Arbarello ((3): ch. 47) scores the absence of a nasal process in †*Dapedium*. However, after reviewing the homologies of this feature, we now agree with Thies and Waschkewitz ((4): ch. 47) and interpret the relatively short process in †*D. stollorum* as a small nasal process and, thus, homologous with the lateral dermethmoids.

49. Width of parasphenoid anterior to the ascending processes

(0) the parasphenoid narrows towards its anterior end, but it is equally broad directly anterior and posterior to the ascending processes (e.g. †*Boreosomus piveteaui* (51): fig. 67)
(1) approximately uniformly broad, as wide or wider than posterior to the processes (e.g. †*Siemensichthys macrocephalus* (18): fig. 84)
(2) distinctly narrower than posterior to the processes (e.g. †*Rhacolepis buccalis* (29): fig. 15)
(3) the parasphenoid bar narrows directly in front of the ascending processes, but it broadens distinctly anteriorly (e.g. †*Macrepistius arenatus* (55): fig. 7)

This character summarizes four main shapes of the anterior portion of the parasphenoid. State 0 represents the condition observed in the outgroup taxa †*Pteronisculus*, †*Australosomus* and †*Boreosomus*. Most halecomorphs (e.g. *Amia calva*), several teleosts (e.g. †*Pholidorhynchodon malzanni*, †*Siemensichthys macrocephalus*) present the condition described in state 1, whereas state 2 is found in lepisosteids and several teleosts. The condition described in state 3 has only been observed in †*Macrepistius* and †*Ichthyokentema* among the taxa included in our data matrix, and seems to have evolved independently in the two taxa.

50. Posterior extent of parasphenoid

(0) short, does not extend beyond the fissura oticalis-ventralis
(1) short, but extending beyond the fissura oticalis-ventralis
(2) long, reaching the occipital condyle or close to it
(3) very long, extending posterior to the occipital condyle

Modified from Gardiner et al. ((38): ch. 12 App. 1). We added character states 0 and 1 to represent the conditions found in the outgroup taxa (†*Australosomus*, †*Boreosomus*, and †*Pteronisculus*).

Other authors also coded this feature. Brito ((6): ch. 14) and Xu and Wu ((48): ch. 7) include our states 0–2, but not state 3. Coates ((56): ch. 36) state 0 is equivalent to our state 0, but his state 1 includes our states 1, 2, and 3. Arratia ((20): ch. 11 App. 3) and Arratia ((11): ch. 26) only distinguishes a parasphenoid “extending posterior to basioccipital” (our state 3) and Arratia ((26): ch. 31) only distinguishes between the parasphenoid “short, reaching anterior margin of basioccipital” or

“long, extending posterior to basioccipital”. The second state of Arratia ((26): ch. 31) corresponds to our state 3 and the scorings of the first state agree with our state 1, but we think that the anterior margin of the basioccipital is not a good landmark due to the variation in the extent of the parasphenoid along the ventral surface of the basioccipital.

51. Basipterygoid processes of parasphenoid
(0) almost or totally absent
(1) well developed

From Gardiner and Schaeffer ((12): ch. 30). Character state 0 includes both the poorly developed processus ascendens anterior of †*Boreosomus*, or *Amia*, and other halecomorphs, and the complete absence of the process in †*Australosomus* and †*Perleidus* because it might be impossible to distinguish between the two conditions in many fossils and they might be functionally equivalent.

Gardiner and Schaeffer (12) scored the presence of a dermal basipterygoid process in †*Boreosomus*, but as explained above, the process in this taxon is very poorly developed.

52. Ascending processes of parasphenoid
(0) present
(1) absent

From Patterson (57). The ascending processes or processus ascendens posterior are present in most actinopterygian taxa and their absence in a few fishes like †*Mimipiscis* or the gars is interpreted as secondarily derived (58, 59).

53. Orientation of the ascending processes of parasphenoid
(0) extend perpendicularly
(1) extend anteriorly
(2) extend posteriorly

This character representing the different directions in which the parasphenoid ascending process might be oriented is taken from Hilton and Forey ((60): ch. 16).

54. Extent of the ascending processes of parasphenoid
(0) high, reaching the sphenotic (Fig. 2B–C)
(1) low, sutured to the prootic only (Fig. 2A)

From Gardiner et al. ((38): ch. 5 App. 2).

55. Laterally sliding articulation between the metapterygoid and the parasphenoid basipterygoid process in adults
(0) absent
(1) present

From Grande ((5): ch. 59).

56. Parasphenoid tooth patch
(0) absent
(1) present

57. Extent of parasphenoid tooth patch
(0) extends anterior and posterior to the ascending processes
(1) extends only anterior to the ascending processes
(2) extends only posterior to the ascending processes
(3) limited to the area between the ascending processes

Modified from Gardiner et al. ((38): ch. 11 App. 1). We have added character states to represent all the observed variation.

58. Vomer(s)

(0) absent
(1) present

From Olsen and McCune ((15): ch. 26). Brito ((6): ch. 13) includes this and the following character in a single multistate character, which is an alternative to our coding, though not recommended by Brazeau (2).

59. Vomer in adults
(0) pair
(1) co-ossified

From Patterson ((61): ch. 8).

Palatoquadrate

60. Autopalatine bone
(0) present
(1) absent

From Grande ((5): ch. 64).

61. Dermopalatine(s) bone(s)
(0) separate
(1) fused to autopalatine

From Sferco et al. ((10): ch. 20). The character is inapplicable for those taxa in which the autopalatine does not ossify (e.g. *Hiodon*).

62. Shape of ectopterygoid
(0) elongate, triangular, straight ventral border, deepest mid length (e.g. *Amia calva* (9): fig. 50)
(1) approximately crescent shape, convex dorsally, concave ventrally (e.g. †*Macrosemimimus fegerti* (62): fig. 4)
(2) approximately boomerang shape (e.g. †*Rhacolepis buccalis* (29): fig. 16)
(3) approximately triangular, deepest posteriorly, tapering anteriorly (e.g. †*Masillosteus janeae* (5): fig. 462, †*Dentilepisosteus laevis* (5): fig. 492)
(4) elongate, with bar-like anterior portion and deeply expanded posterior portion (e.g. *Lepisosteus osseus* (5): figs. 60–61, *Atractosteus spatula* (5): figs. 254–255)
(5) bar-like (e.g. *Hiodon alosoides* (31): figs. 45–48)

Modified from López-Arbarello and Wencker ((40): ch. 11). We have reformulated the character states to improve descriptive resolution and accommodate the morphologies observed in the taxa included in our data matrix.

63. Marginal row of teeth on ectopterygoid
(0) absent
(1) present

The pterygoid bones of actinopterygians are usually paved with minor teeth. In addition, several neopterygians have a marginal row of larger teeth, which are normally aligned with the teeth of the dermopalatine bone or bones.

64. Ectopterygoid participation in palatal surface area
(0) ectopterygoid forms half or less of the palatal region
(1) ectopterygoid forms most of the palatal region

Taken from Grande ((5): ch. 63).

65. Part of dorsal surface of ectopterygoid ornamented and forming part of skull roof

(0) absent
(1) present

From Grande ((5): ch. 61).

66. Endopterygoid dentition
(0) present
(1) absent

From López-Arbarello ((3): ch. 15).

67. Metapterygoid/quadrate relationship
(0) metapterygoid sutured or closely associated with the quadrate
(1) ectopterygoid separating metapterygoid from quadrate

From Grande ((5): ch. 60).

68. Metapterygoid/ectopterygoid contact
(0) absent, bones separated by the endopterygoid (e.g. †*Macrosemimimus fegerti* (62): fig. 4)
(1) present, metapterygoid placed anteriorly (e.g. *Amia calva* (9): fig. 50)
(2) present, metapterygoid expanded anteriorly (e.g. †*Rhacolepis buccalis* (29): fig. 16)
(3) present, ectopterygoid enlarged posteriorly (e.g. *Lepisosteus osseus* (5): figs. 60–61, *Atractosteus spatula* (5): figs. 254–255)

69. Relative position of the lower jaw articulation at the level of
(0) posterior to the orbit
(1) around the posterior border of the orbit
(2) around the centre of the orbit
(3) around the anterior border of the orbit
(4) in front of the orbit

The relative position of the lower jaw articulation has been scored in different ways by several authors (e.g. (20): ch. 41 App. 3, (5): ch. 48, (3): ch. 16, (11): ch. 62, (63): ch. 45, (50): ch. 59, (10): ch. 66). Compared to previous authors, we have here defined more character states aiming to improve and facilitate the scoring of this character.

70. Quadratojugal
(0) present
(1) absent

From Olsen and McCune ((15): ch. 22).

71. Quadratojugal fused to quadrate
(0) absent
(1) present

72. Quadratojugal
(0) plate-like
(1) splint-like

From Gardiner et al. ((38): ch. 24 App. 1), Hurley et al. ((43): ch. 43), and López-Arbarello and Wencker ((40): ch. 15).

73. Ventral articular facet of quadratojugal portion of the teleost quadrate
(0) absent (Fig. 5)
(1) present (Fig. 6)

López-Arbarello and Sferco (53) noticed the presence of a well-defined concave articular facet in the posteroventral margin of the quadrate, adjacent to the quadrate condyle, in several Jurassic teleosts. They discuss the possible function of this articular facet, which articulates with the postarticular

process of the lower jaw probably preventing an extreme downward rotation of the lower jaw. They also argue that this facet is formed within the area of the quadrate that might be homologous with the quadratojugal (3, 45, 46, 54, 57, 61, 64-69), and discuss the probably analogue saddle-like articular facet in the anteroventral margin of the quadratojugal of the lepisosteiform †*Scheenstia zappi*. We have now traced the distribution of the ventral articular facet of the quadrate, which is present in most of the examined Jurassic teleosts.

74. Shape of the dorsal margin of the quadrate
(0) convex
(1) straight
(2) concave
(3) sinuous or sigmoid
(4) notched

From Sferco et al. ((10): ch. 44). State 4 is autapomorphic for *Brycon meeki* in our data matrix.

Hyoid arch

75. Urohyal
(0) absent
(1) present

From Sferco et al. ((10): ch. 51).

76. Endoskeletal basihyal
(0) absent
(1) present

From Patterson ((61): ch. 42).

77. Basihyal tooth plates
(0) absent
(1) present

78. Organization of basihyal tooth plates
(0) simple
(1) consisting of a mosaic of entoglossals forming a "tongue bone"

Characters 77 and 78 are modified from Cavin ((70): ch. 32) and Grande ((5): ch. 77) to avoid “pseudo-ordering” according to Brazeau (2).

79. Number of hypohyal ossifications
(0) one pair
(1) two pairs, dorsal and ventral hypohyals

From Sferco et al. ((10): ch. 49).

80. Trajectory of the hyoidean artery piercing the hypohyals
(0) absent
(1) present

Modified from Arratia ((21): ch. 61).

81. Shape of anterior ceratohyal
(0) deep, subrectangular (e.g. †*Ticinolepis longaeva* (71): fig. 10)
(1) elongate hourglass shape (e.g. *Lepisosteus osseus* (5): fig. 67)
(2) narrow anteriorly, more expansive and laterally compressed posteriorly (e.g. *Amia calva* (9): fig. 55)

(3) deeply hourglass shape without Beryciform foramen (e.g. †*Neosemionotus puntanus* (72): fig. 5)
(4) deeply hourglass shape with Beryciform foramen (e.g. †*Luisiella feruglioi* (73): fig. 7C)

Modified from Gardiner et al. ((38): ch. 30 App. 1) adapting to the morphological variation represented in our data matrix. Stewart ((34): ch. 13), Alvarado-Ortega ((33): ch. 32), Cavin et al. ((36): ch. 40), Sferco et al. ((10): ch. 48) only include the presence of a Beryciform foramen and, thus, using those codings would produce an unspecified absence (1).

82. Symplectic bone
(0) absent
(1) present

From Gardiner and Schaeffer (12: ch. 23).

83. Symplectic relationship to lower jaw
(0) is not directly involved in the jaw joint
(1) participates directly in the jaw joint

From Olsen and McCune ((15): ch. 19). According to our observations (Fig. 5), the symplectic does not reach the lower jaw in †*Aspidorhynchus acutirostris*.

84. Relation between symplectic and quadrate
(0) symplectic posteroventral to quadrate, the two bones are separate
(1) symplectic articulates with the inner, medial surface of quadrate
(2) symplectic attached into a groove formed by the posteroventral border of the quadrate
(3) symplectic posterodorsal and well separate from quadrate
(4) symplectic attached between the quadrate and quadratojugal

Modified from Hurley et al. ((43): ch. 45).

85. Shape of symplectic
(0) slightly curved tube or splint
(1) bar-like shaped
(2) hatchet shaped
(3) L-shaped

Modified from Grande ((5): ch. 68).

86. Preopercular process of hyomandibula (Fig. 10)
(0) absent
(1) present

From Arratia ((20): ch. 36 App. 3).

87. Opercular process of hyomandibula notably elongated
(0) absent
(1) present

From Grande and Bemis ((9): ch. 24). The observed variation embraced in our absence character state is so gradual and noticeably different from the condition in the present state, that this is not a case of unspecified absence (1).

88. Hyomandibular articulation with the neurocranium
(0) by means of one articular surface
(1) by means of two distinct articular surfaces

From Sferco et al. ((10): ch. 42).

89. Shape of hyomandibula

(0) rod like
(1) hourglass shaped, with a constriction between the shaft and the articular head
(2) shaft approximately as broad as hyomandibular head
(3) shaft notably narrower than hyomandibular head

Dermal braincase

90. Number of extrascapular bones
(0) one pair
(1) two pairs
(2) more than four extrascapulars

From López-Arbarello ((3): ch. 20). Brito ((6): ch. 20) distinguish only two states corresponding to our states (0), and (1) plus (2) together. The distinction between our states (1) and (2) is based on our own observations. Besides the numerous taxa with a single pair of extrascapular bones, several other taxa present only two pairs of extrascapulars and this number is stable even between closely related species. Differently, taxa with more than two pairs of extrascapulars usually present intraspecific variation in the total number of extrascapulars, including uneven counts. Similarly, Grande and Bemis ((9): ch. 49) includes only two states for this character: presence of only one vs. three pairs.

91. Shape of the extrascapular bone/s
(0) plate like, quadrangular to triangular
(1) tubular
(2) semicircular, expanded caudolaterally
(3) expanded rostrally

Modified from Li and Wilson ((74): ch. 27) and Sferco et al. ((10): ch. 8). State 3 is autapomorfic for *Hiodon alosoides* in our data matrix.

92. Posterior extension of parietals median to the single pair of laterally placed extrascapular bones
(0) absent
(1) present

From López-Arbarello ((3): ch. 21).

93. Dermopterotic (or pterotic) exposed surface extending anterior to parietal
(0) by more than 50% of its total length
(1) by more than 40% but less than 50% of its total length
(2) by more than 30% but less than 40% of its total length
(3) by less than 30% of its total length
(4) not extending anterior to parietal

Modified from Gardiner et al. ((38): ch. 15 App. 2). The ranges of the different character states proposed here are based on measurements taken on 32 taxa (Fig. S2-2).

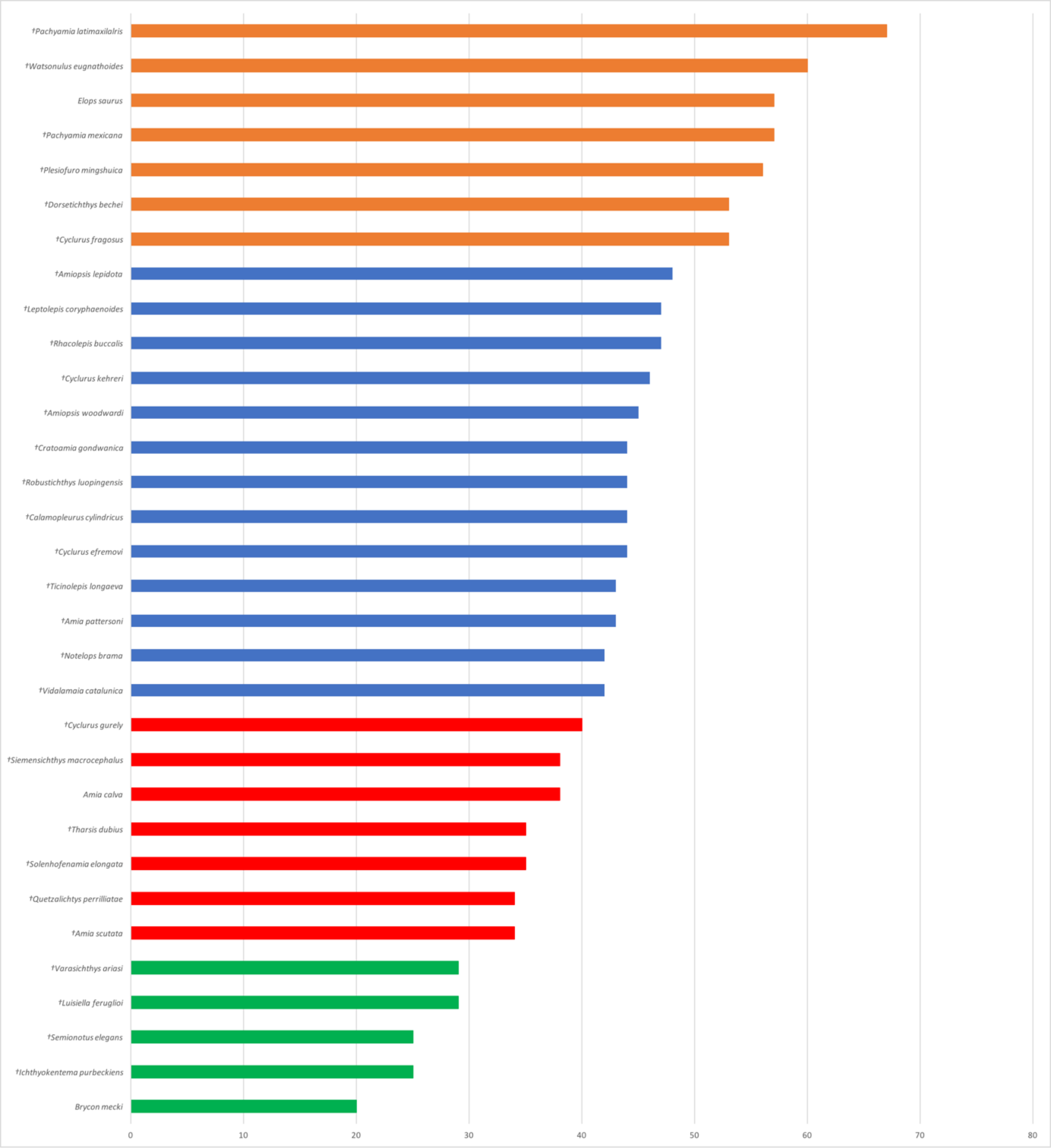

Figure S2-2: Relative length of the anterior portion of the exposed surface of the dermopterotic in 32 studied taxa, expressed as ratios: length of the exposed surface of the dermopterotic anterior to parietal / total length of the exposed surface of the dermopterotic. The different colors represent different character states: orange (0), blue (1), red (2), green (3).

94. Dermopterotic (or pterotic) exposed surface extending posterior to parietal

(0) absent
(1) present

95. Shape of exposed surface of dermopterotic

(0) roughly rectangular (e.g. †*Ticinolepis longaeva* (71): figs. 7, 9-10, 12)
(1) resembling an inverted L (e.g. †*Luisiella feruglioi* (73): fig. 4)
(2) roughly triangular (e.g. †*Caturus furcatus* (9): fig. 401A)

Modified from Grande and Bemis ((9): ch. 35).

96. Dermopterotic descending lamina

(0) absent
(1) present

97. Dermopterotic posterior projection(s)
(0) absent
(1) present and simple
(2) present, one or more, and notably large

Modified from Grande and Bemis ((9): ch. 27), which is equivalent to our state (2).

98. Condition of the skull roof
(0) medioparietal
(1) lateroparietal

From Cavin ((35): ch. 1).

99. Parietal width to length ratio
(0) not exceeding 0.90
(1) well exceeding 0.90

From Grande and Bemis ((9): ch. 18). We differ from Grande and Bemis (9) in the scoring of this character for †*Dorsetichthys bechei*.

100. Relative length of parietals respect to the length of the frontals
(0) less than one half but more than one third the length of frontals
(1) about half the length of frontals
(2) less than one third the length of frontals
(3) more than half the length of frontals

From López-Arbarello ((3): ch. 22). Other authors use different character states (e.g. Cavin et al. ((49): ch. 20).

101. Length to width ratio of frontals in adult sized individuals
(0) lower than three
(1) equal or larger than three

From López-Arbarello ((3): ch. 23). Other authors (e.g. (9): ch. 34, (70): ch. 12, (75): ch. 34, (14): ch. 29, (50): ch. 34) use different ranges for their character states.

102. General shape of frontal
(0) subrectangular not significantly narrower anteriorly than posteriorly
(1) tapering gradually
(2) strongly constricted at the orbit; minimal interorbital width less than half of maximal width in the temporal region
(3) with tubular antorbital portion

This character merges Arratia ((21): ch. 188) and López-Arbarello ((3): ch. 24) with López-Arbarello ((3): ch. 25) because they represent different versions of the same feature, i.e. the general shape of the frontal, and are thus not mutually independent. Additionally, we added the character state 2, which is not represented in the data matrix of López-Arbarello (3).

103. Postorbital portion of frontal
(0) not significantly large (e.g. †*Semionotus bergeri* (76): figs. 4-5)
(1) large, more than 1/3 of total length of frontal (e.g. †*Ticinolepis longaeva* (71): figs. 9-10)

104. Preorbital portion of frontal
(0) not significantly large (e.g. †*Ticinolepis longaeva* (71): figs. 9-10)
(1) large, more than 1/3 of total length of frontal (e.g. †*Occitanichthys canjuerensis* (40): fig. 5)

Characters 103 and 104 are proposed here to represent the variation observed in the position or extension of the frontal relative to the orbit. Typically in amiids, but also in †*Caturus furcatus*, †*Watsonulus eugnathoides* among halecomorphs, the ginglymodian †*Ticinolepis longeva*, the teleost †*Rhacolepis buccalis* and the outgroup taxa †*Plesiofuro mingshuica*, †*Australosomus kochi*, and †*Pteronisculus stensioi*, the frontal has a relatively large postorbital portion (103[1]). Contrary, in the most common condition among ginglymodians, the frontal extends anterior to the orbit (character (104[1]). This condition is also found among halecomorphs in †ophiopsids and †*Quetzalichthys perrillae*.

105. Interfrontal fontanelle in adults
(0) absent
(1) present

From Grande and Bemis ((9): ch. 37).

106. Shape of nasal bones
(0) broad, approximately rectangular to oval or trapezoidal (e.g. †*Ticinolepis longaeva* (71): figs. 9-10)
(1) broad kidney shaped (e.g. †*Lepidotes elvensis* (40): fig. 12C)
(2) approximately tubular (e.g. *Hiodon alosoides* (31): figs. 28-29)
(3) approximately tear-shape, broadest anteriorly, narrowing posteriorly (e.g. †*Solnhofenamia elongate* (9): figs. 330-333)
(4) small, approximately kidney shaped (e.g. *Atractosteus spatula* (5): figs. 216-217)
(5) long and narrow, anterior end incurved laterally (e.g, †*Sangiorgioichthys sui* (77): fig. 4)
(6) inverted Y-shaped (e.g. †*Quetzalichthys perrilliatae* (78): figs. 4-5)

The shape of the nasal bones is highly variable among neopterygians and several authors have code a character with different states to encompass the variation observed in their corresponding sample of taxa (e.g. (74): ch. 12, (79): ch. 2, (80): ch. 7, (3): ch. 28, (10): ch. 3). Similarly, the character states proposed herein represent the numerous morphotypes found among the taxa included in our data matrix.

107. Relationship between left and right nasal bones
(0) sutured to each other (e.g. †*Ticinolepis longaeva* (71): figs. 9-10)
(1) close to each other (e.g. †*Lepidotes elvensis* (40): fig. 12C)
(2) widely separated (e.g. *Hiodon alosoides* (31): figs. 28-29)

Modified from Sferco et al. ((10): ch. 4).

108. Position of nasals relative to frontals
(0) nasals placed anterior to frontals (e.g. †*Ticinolepis longaeva* (71): figs. 9-10)
(1) nasals placed lateral to frontals (e.g. †*Quetzalichthys perrilliatae* (78): figs. 4-5)

Independently of the extension of the preorbital portion of the frontal bones, the nasals of most neopterygian taxa are placed anterior to the frontals either directly articulated with them or loosely attached in the soft tissue. In many teleosts, the nasals are placed lateral to the frontals, which is the most common condition among Jurassic teleosts.

Arratia ((26): ch. 24) use a character coding “nasals bones: joined in midline [0]; separate from each other by parietal bones [= frontals] [1]”. This is a case of “compound character” (2), in which two mutually independent features are taken together: the relationship between the two nasals (coded in our character 107) and the relative position of the nasals respect to the frontals (our character 108). To exemplify, the nasals in *Hiodon alosoides* are well separated from each other, but they are placed anterior and not lateral to the frontals.

The nasals in the halecomorphs †*Oshunia brevis* and †*Quetzalichthys perrilliatae* are also lateral to the frontals.

109. Posterior nostril relationship to nasal bone
(0) excavating the lateral margin of the nasals

(1) completely included in the posterior portion of the nasals
(2) cutting the posterolateral border of the nasals
(3) not indicated by the nasals

Modified from Arratia ((11): ch. 21). In many ginglymodians and halecomorphs the anterior end of the nasal bones is laterally incurved or the lateral border of the bone is deeply concave, in both cases probably surrounding the posterior nare, but the bone itself is not excavated or perforated as in character states 0 to 2. Our state 1 is equivalent to the a/p character Arratia ((11): ch. 21).

110. Nasal bones participate in the orbital margin
(0) present
(1) absent

From Xu and Wu ((48): ch. 22).

Cheek and circumborbital bones

111. Ossified sclerotic ring
(0) present
(1) absent

From Grande and Bemis ((9): ch. 14). Grande and Bemis (9) score the presence of a sclerotic ring in †*Ionoscopus cyprinoides*, but we haven't found evidence for these ossifications in any specimen. According to Hilton ((80)) there are sclerotic bones in *Hiodon alosoides*, which is thus here scored with 111(0), differing from Arratia ((11): ch. 50, (26): ch. 56), who score the absence of sclerotic bones for this genus.

112. Quadrate laterally covered by infraorbital bones
(0) absent
(1) present

From López-Arbarello ((3): ch. 40) and it is important to read the corresponding discussion there to avoid double scoring if using Cavin ((70): ch. 19).

113. Supraorbital bones
(0) present
(1) absent

From Gardiner and Schaeffer ((12): ch. 14).

114. Circumborbital ring
(0) most anterior supraorbital does not contact infraorbitals
(1) most anterior supraorbital contacts infraorbital series

From Hurley et al. ((43): ch. 29).

115. Number of supraorbital bones
(0) more than four
(1) three or four
(2) two
(3) one

The number of supraorbital bones has been used as a character, with different character states, by several authors (e.g. (12): ch. 14, (9): ch. 12). Other authors (e.g. (11): ch. 49, (10): ch. 33, (13): ch. 17) code the absence of supraorbitals together with the different amounts of supraorbitals in a single multistate character, which would lead to "pseudo-ordering" (2).

116. Arrangement of supraorbital bones
(0) in one row

(1) in two rows

117. Size of supraorbital bones relative to orbit
(0) small
(1) large
(2) all supraorbitals small except the first, most anterior supraorbital, which is large, expanded anteriorly

From López-Arbarello ((3): ch. 31).

118. Shape of most anterior supraorbital bone
(0) subrectangular to triangular, if the ring is closed, contacting only the antorbital or one infraorbital bone
(1) trapezoidal, longest ventrally, contacting more than one infraorbital bone
(2) pentagonal
(3) expanded anteroventrally, making the anterodorsal corner of the orbit

Modified from López-Arbarello ((3): ch. 32), which only considers our state 1, i.e. the presence/absence of a large supraorbital with expanded anteroventral portion. In our data matrix, states 2 and 3 are autapomorphies of †*Tlayuamichin itzli* and †*Araripelepidotes temnurus*, respectively.

119. Size and shape of dermosphenotic
(0) extended and tapering rostrally (e.g. *Hiodon alosoides* (31): fig. 36)
(1) extended and tapering caudally, ending at the level of the anterior margin of the dermopterotic (or pterotic) (e.g. *Amia calva* (9): fig. 18-19)
(2) very large, extended and tapering caudally, reaching close to the level of the preopercular canal (e.g. †*Aspidorhynchus acutirostris* Brito (6): fig. 31)
(3) small, not distinctly extended rostrally or caudally (e.g. †*Ticinolepis longaeva* (71): fig. 6)
(4) large, keystone shaped (e.g. *Lepisosteus osseus* (5): figs. 27-28)

The size and shape of the dermosphenotic have been expressed in different ways and included as a character by several authors (e.g. (10): ch. 35, (13): ch. 12). An anteriorly expanded dermosphenotic (state 0) is found in the outgroup taxa †*Pteronisculus* and †*Boreosomus*, and in several teleosts (†*Pachycormus*, †*Parapholidophorus*, †*Pholidorhynchodon*, †*Diplomystus*, and the living *Elops*, *Brycon* and *Hiodon*). The opposite, a posteriorly extended dermosphenotic (state 1) is only found among amiiforms (*Amia*, †*Cyclurus* and †*Pachyamia*). In *Salmo*, †*Notelops*, †*Rhacolepis* and †*Aspidorhynchus*, the dermosphenotic is also expanded posteriorly, but it is distinctly very large in these taxa (state 2). Also relatively very large is the keystone shaped dermosphenotic (state 4) of most lepisosteiforms. However, in the most common condition amongst the studied neopterygians, the dermosphenotic is a relatively small, approximately rectangular bone (state 3).

120. Position of dermosphenotic relative to orbital margin
(0) dermosphenotic participates in orbital margin
(1) dermosphenotic is excluded from orbital margin

From Grande and Bemis ((9): ch. 38).

121. Dermosphenotic/sphenotic association
(0) closely associated with each other (i.e. contacting or fused to each other)
(1) not in contact with each other

From Grande ((5): ch. 22).

122. Dermosphenotic incorporated into skull roof in adult sized individuals
(0) absent, hinged to the side of skull roof
(1) present, sutured into skull roof, forming part of it

From Olsen and McCune ((15): ch. 23).

123. Shape of the postinfraorbital bones (jugal not included)
(0) deeper than long, sometimes almost tubular (e.g. †*Ticinolepis longaeva* (71): fig. 6)
(1) approximately quadrangular (e.g. †*Scheenstia zappi* López-Arbarello (53): fig. 5)
(2) longer than deep, expanded posteriorly, not longer than the orbital diameter (e.g. *Lepisosteus osseus* (5): figs. 27-28)
(3) very large, longer than the orbit (e.g. *Amia calva* (9): figs. 18-19)
(4) approximately triangular, tapering dorsally (e.g. †*Caturus furcatus* (9): fig. 401)
(5) subrectangular and elongated in posterodorsal to anteroventral direction (e.g. †*Macrepistius arenatus* (81): fig. 2)

Modified from López-Arbarello ((3): ch. 37).

124. Number of postorbital bones (infraorbital bones forming the posterior rim of the orbit (jugal not included)
(0) one
(1) two
(2) three
(3) none

We keep the absence state within this character (state 3 in this case), because it is not possible to establish one-to-one relationships of homology between individual infraorbital bones (see discussion in López-Arbarello (3)). Therefore, this is not a case of "pseudo-ordering" (2), but just different patterns of infraorbital bones.

125. The largest circumborbital bone is
(0) the jugal (infraorbital placed at the posteroventral corner of the orbit), and it is not much larger than other infraorbital bones (e.g. †*Luisiella feruglioi* (73): fig. 4)
(1) the jugal (infraorbital placed at the posteroventral corner of the orbit), and it is notably larger than other infraorbitals (e.g, †*Sangiorgioichthys sui* (77): fig. 4)
(2) a postorbital occupying the posteroventral and posterior rim of the orbit (in some cases this area is occupied by one or more bones of similar size) (e.g. *Amia calva* (9): fig. 18)
(3) the dermosphenotic or a supraorbital (all these bones being of similar size) (e.g. *Lepisosteus osseus* (5): figs. 27-28)
(4) one of the supraorbitals (the dermosphenotic is notably smaller than the supraorbitals) (e.g. †*Neosemionotus puntanus* (72): fig. 5)
(5) the infraorbital placed at the centre of the ventral margin of the orbit (e.g. †*Ticinolepis longaeva* (71): fig. 6)
(6) the lachrymal or infraorbital placed at the anteroventral corner of the orbit (e.g. †*Teoichthys brevipina* (82): fig. 2)
(7) one of the anterior infraorbitals (e.g. †*Macrosemimimus fegerti* (62): figs. 5-6)
(8) the jugal or the lachrymal, both with similar size (e.g. †*Archeosemionotus connectens* (14): fig. 6)

The high variation in the relative size of the infraorbital bones in neopterygians led us to coding eight different character states. These numerous character states cannot be separated as independent characters because it is not possible to establish one-to-one relationships of homology between most of the infraorbital bones (see discussion in López-Arbarello (3)).

126. Contact between preopercle and jugal or lower postinfraorbital
(0) absent
(1) present

Modified from López-Arbarello ((3): ch. 36).

127. General shape of the subinfraorbital bone(s) (Infraorbital bones forming the ventral border of the orbit between the lachrymal and the jugal)
(0) subrectangular to triangular, about 1,5 to 2,5 times deeper than long (e.g. †*Semionotus bergeri* (76): figs. 4-5)

(1) subtriangular, longer ventrally, more than 3 times deeper than long (e.g. †*Macrepistius arenatus* (81): fig. 2)
(2) quadrangular to longer than deep, notably smaller than the orbit (e.g. †*Ticinolepis longaeva* (71): fig. 6)
(3) subrectangular, a quarter to half the size of the orbit (e.g. †*Archeosemionotus connectens* (14): fig. 6)
(4) scroll-like (e.g. †*Propterus elongatus* Bartram (54): fig. 24)

Modified from Grande and Bemis ((9): ch. 43) code the shape of the anterior subinfraorbital bone, which corresponds to the most anterior bone forming the ventral rim of the orbit, when more than one infraorbital is present between the lachrymal and the jugal. In most halecomorphs there is usually only one infraorbital between these bones and, thus, theirs and ours character are equivalent, referring to the same bone. However, the character states in Grande and Bemis ((9): ch. 43) are not directly comparable to our character states. Grande and Bemis ((9): ch. 43) state 1 (subrectangular, deeper than long) is equivalent to our state (0) and we added a the new state (1) to represent the condition in †*Macrepistius*, which is unique of this taxon in our data matrix. On the other hand, our state (2) encompasses their states 0 (short, subrectangular longer than deep), 2 (long, very thin, tube like), and 3 (posteriorly expansive, tapering anteriorly) because the general shape in the three cases is approximately the same, with little variation (e.g. the tubular shape is usually present in very small specimens, and the bones are short when there are more than one infraorbital in this series, which is a different character).

128. Ventral surface of subinfraorbital bones intensely pitted
(0) absent
(1) present

Modified from Alvarado-Ortega and Espinosa-Arrubarrena ((78): ch. 18).

129. Lachrymal size relative to orbit
(0) significantly smaller than orbit (e.g. †*Ticinolepis longaeva* (71): fig. 6)
(1) about half to equal size of orbit (e.g. †*Archeosemionotus connectens* (14): fig. 6)
(2) about size of orbit or larger (e.g. †*Macrepistius arenatus* (81): fig. 2)

Modified from Grande and Bemis ((9): ch. 58) by adding new states to represent all the variation contained in our data set.

130. Anterior infraorbitals
(0) absent (e.g. †*Luisiella feruglioi* (73): fig. 4)
(1) present (e.g. †*Macrosemimimus fegerti* (62): figs. 4-6)

From Olsen and McCune ((15): ch. 1). The term ‘anterior infraorbitals’ (after Wenz (83)) refers to the infraorbital bones placed anterior to the anterior border of the orbit and posterior to the antorbital, which do not contribute to the orbital margin. Different names have been used for these bones in the literature: preorbitals (84), lachrymals (15, 46), or anterior infraorbitals (83, 85), among which the latter is preferred here because it highlights the homology of these bones with the other infraorbital bones (serial homology; (3): pp. 10–12).

131. Number of anterior infraorbitals
(0) one
(1) two or more
(2) three or more
(3) four or more
(4) five or more
(5) six or more
(6) seven or more

From López-Arbarello and Wencker ((40): ch. 31), modified from Bermúdez-Rochas and Poyato-Ariza ((86): ch. 92). The number of anterior infraorbital bones is intraspecifically and in some cases

even individually variable. Therefore, we score the minimal number of anterior infraorbitals. Character state 6 is autapomorphic for †*Tlayuamichin* in our data matrix.

132. Shape of most anterior anterior-infraorbital
(0) not distinct than adjacent infraorbital (e.g. †*Ticinolepis longaeva* (71): fig. 6)
(1) rectangular dorsal portion, ventrally expanded and higher than adjacent infraorbital (e.g. †*Lepidotes* (3): figs. 8, 20)
(2) narrowing dorsally, expanded ventrally, further ventral than the adjacent anterior infraorbitals (e.g. †*Macrosemimimus fegerti* (62): fig. 6)
(3) approximately rectangular, deeper than adjacent anterior infraorbital (e.g. †*Masillosteus janeae* (5): figs. 456-457)

Modified from López-Arbarello ((3): ch. 35).

133. Ventral border of infraorbital series
(0) follows a straight line or a gentle curve (e.g. †*Occitanichthys canjuerensis* (40): fig. 6)
(1) flexes abruptly dorsally at the anterior margin of the orbit (e.g. †*Lepidotes* (3): figs. 8, 20)

From López-Arbarello ((3): ch. 30). This character is only applicable for those taxa with anterior infraorbitals.

134. A series of toothed infraorbitals bordering the snout
(0) absent ((3): figs. 7A-C)
(1) present ((3): figs. D)

From Wiley ((46): ch. 3).

135. Independent antorbital bone
(0) present
(1) absent

From Cavin ((35): ch. 23).

136. Shape of antorbital bone
(0) approximately triangular or drop-like, narrowing posterodorsally
(1) rectangular
(2) clavate
(3) sickle- to L-shape
(4) approximately triradiated, Y-shape
(5) tubular
(6) splint-like

Modified from the multistate characters in Gardiner et al. ((38): ch. 18 App. 1), Hurley et al. ((43): ch. 32), and Sferco et al. ((10): ch. 38), including different character states, which have been merged and adapt to represent the morphotypes recognized in the taxa included in our analysis.

Arratia ((20): ch. 30 App. 3, (21): ch. 38, (23): ch. 34, (22): ch. 34, (27): ch. 33) uses a binary character coding the presence/absence of a comma-shaped antorbital bone only, thus producing an unspecified absence (1) including different morphologies, e.g. †*Anaethalion*, †*Dorsetichthys*, *Elops*, †*Thrissops*, and †*Varasichthys* (here state 0), †*Aspidorhynchus* and *Brycon* (here state 4), *Salmo* and †*Diplomystus* (here state 6), †*Pachycormus*, and *Amia* (here state 2).

Cavin et al. (35) questioned the presence of an antorbital bone in †*Thrissops*, but the bone is well preserved in specimens of †*Thrissops formosus* (e.g. JME-ETT 74, 887, 972, 1350, 1805).

137. Tapering canal bearing anterior arm of antorbital bone
(0) absent
(1) present

Modified from Hurley et al. (43) and Grande ((5): ch. 12).

138. Antorbital bone carrying a portion of the infraorbital sensory canal
(0) infraorbital canal extends into antorbital bone
(1) infraorbital canal ends blindly in the first infraorbital

From Arratia ((20): ch. 23 App. 3).

139. Antorbital bone participates in the anterior orbital margin
(0) absent
(1) present

From Xu et al. ((50): ch. 78).

140. Independent rostral bone
(0) absent, ethmoidal commissure in antorbitals (see †*Boreosomus piveteaui* (51): 69)
(1) present (e.g. *Amia calva* (9): fig. 10, *Atractosteus spatula* (5): figs. 216A, 217A)
(2) absent, ethmoidal commissure in a compound mesethmoid (Fig. 4D)
(3) absent, presence of a rostrodermethmoid (e.g. †*Notelops brama* (29): fig. 1)
(4) absent, no trace of ethmoidal commissure

Modified from Sferco et al. ((10): ch. 80).

141. Shape of rostral bone
(0) relatively small approximately rectangular to tube-like (e.g. †*Ticinolepis longaeva* (71): figs. 8-9)
(1) relatively large and shield-like without lateral horns (e.g. †*Dapedium pholidotum* (4): figs. 7-8)
(2) shield-like with lateral horns (e.g. †*Catervariolus hornemani* (87): figs. 9, 13, 17)
(3) relatively large and roughly rounded or rectangular-shaped without lateral horns (e.g. †*Siemensichthys macrocephalus* (18): fig. 145)
(4) roughly V-shaped, with lateral horns and caudal triangular process (e.g. *Amia calva* (9): figs. 10-15)

Modified from Gardiner et al. ((38): ch. 19 App. 1) and Arratia ((26): ch. 7). We separated their state 0 (plate-like or short tube-like, without lateral horns) in our states (0) and (1), and we added state 2 and 3 to represent the shape of the rostral in several Triassic and Jurassic teleosts (11).

142. Independent post-rostral bone
(0) present
(1) absent

From Xu et al. ((13): ch. 52).

143. Suborbital bones
(0) present
(1) absent

From Gardiner and Schaeffer ((12): ch. 9).

144. Number of suborbital bones
(0) one
(1) two
(2) three
(3) four or more, usually numerous suborbitals

Modified from López-Arbarello and Wencker ((40): ch. 39).

145. Distribution of suborbital bones
(0) suborbitals limited to the area between the infraorbitals and preopercle posterior to the orbit

(1) suborbitals occupy the area between the infraorbitals and preopercle posterior and below the orbit

Modified from López-Arbarello ((3): ch. 42), which is a compound character (2) and, thus, we now coded the different independent traits in the separate characters 145 and 146.

146. Arrangement of suborbital bones
(0) one row
(1) two rows
(2) mosaic of numerous suborbitals

Modified from López-Arbarello ((3): ch. 42).

147. Independent of the total number, there is a large suborbital covering almost the whole area between the infraorbital bones and the preopercle
(0) absent
(1) present

From López-Arbarello ((3): ch. 43).

148. Independent of the total number, there is a suborbital bone between the dermopterotic and preopercle
(0) absent
(1) present (see López-Arbarello and Alvarado-Ortega (88): fig. 7)

149. Relative size of the most anterior and most dorsal suborbitals
(0) of similar size than the other suborbitals
(1) notably larger than the other suborbitals (see (53): fig. 8)

From López-Arbarello ((3): ch. 44). The character is not applicable unless there is a series of at least three suborbital bones.

150. Suborbitals covering the quadrate laterally
(0) present, suborbitals within a series or mosaic, no distinct shape
(1) present, one or two suborbitals forming a triangular plate

From López-Arbarello ((3): ch. 46). The character is not applicable unless there are suborbitals lateral to the quadrate.

Jaws and dentition

151. Relative position of premaxillary bones
(0) anteriorly placed contacting at the midline
(1) anterolaterally to laterally placed separated by ethmoidal and/or rostral ossifications

Modified from Arratia ((11): ch. 9).

152. Premaxillary nasal processes (= lateral dermethmoids)
(0) small lining the nasal pits, without or only partially surrounding the olfactory foramen (Fig. 4A)
(1) large completely enclosing the olfactory fenestra (Fig. 4B)

Modified from Gardiner et al. ((38): ch. 20 App. 1). Since we are following the hypothesis of primary homology between the nasal process of the premaxilla of halecomorphs and ginglymodians and the lateral dermethmoids of teleosts (see discussion of character 48), this character is also scored for those teleosts in which the lateral dermethmoids are lining the nasal pits (†*Catervariolus*, †*Dorsetichthys*, †*Ichthyokentema*, †*Pachycormus*, †*Pholidorhynchodon*, and †*Siemensichthys*). In all these teleosts the lateral dermethmoids do not enclose the olfactory fenestra (state 0). The condition in other teleosts is

not comparable because the participation of the lateral dermethmoids in the nasal pit is very limited or null (18, 69, 80).

153. Premaxillary nasal process relationship to frontal
(0) does not suture to the frontal
(1) sutures to the ventral surface of the frontal
(2) sutures to the frontal and forms an external dermal component of the skull roof

Modified from Grande (5) characters 6 and 7, which are merged in this multistate character because they represent different conditions of the same trait.

154. Maxilla
(0) present and independent
(1) fused to toothed infraorbital bones

Modified from Xu and Ma ((89): ch. 32).

155. Maxilla relationship to preopercle
(0) attached to preopercle
(1) free from preopercle

From Gardiner and Schaeffer ((12): ch. 13).

156. Length of maxilla relative to coronoid process
(0) very long, extends beyond the coronoid process
(1) extends backwards partially covering the coronoid process laterally
(2) very short, does not reach the coronoid process

Modified from López-Arbarello ((3): ch. 50).

157. Posterior extent of maxilla relative to orbit
(0) beyond posterior orbital margin
(1) up to posterior orbital margin
(2) up to centre of the orbit
(3) up to anterior orbital margin
(4) in front of the orbit

Modified from Gardiner et al. ((38): ch. 26 App. 2).

158. Shape of maxilla
(0) elongate, broad posteriorly, stretches well behind the orbit
(1) elongate, shallow
(2) maxilla extremely slender
(3) deep, depth > 0,5 of its length

This character merges Grande and Bemis ((9): ch. 53), Hurley et al. ((43): ch. 39), and López-Arbarello ((3): ch. 51).

159. Ventral margin of maxilla
(0) straight or almost straight
(1) slightly convex
(2) slightly concave
(3) bends downwards posteriorly

Modified from Arratia and Tischlinger ((27): ch. 185).

160. Shape of posterodorsal corner of maxilla
(0) rounded to straight angle (e.g. †*Cyclurus* (9): figs. 243)
(1) acute angle (e.g. †*Vidalamia* (9): figs. 243)

Several authors code the shape of the posterior border of the maxilla (e.g. (9): ch. 62). However, the shape of the posterior border of the maxilla greatly depends on the shape of its posterodorsal corner (this character) and the presence of a postmaxillary process (next character). and we think that these two characters are more useful to represent the variation in the shape of the posterior end of the maxilla.

161. Postmaxillary process
(0) absent
(1) present and small
(2) present and notably large, posterior border of maxilla deeply excavated

Modified from Grande and Bemis ((9): ch. 30).

162. Shape of dorsal margin of maxilla
(0) straight or concave infraorbital and convex postorbital portions
(1) generally straight or convex
(2) gently concave allocating supramaxilla
(3) with a distinct supramaxillary notch

163. Supramaxilla
(0) absent
(1) present, single bone
(2) present, two bones

From Brito ((6): ch. 19).

164. Relative length of single supramaxilla
(0) long, about half the length of the maxillary blade (without articular process) or larger
(1) short, about a quarter of the length of the maxilla

165. Relative position of supramaxilla(ae)
(0) placed dorsal to the dorsal margin of maxilla
(1) placed posterodorsal to the maxilla

From Arratia ((22): ch. 42).

166. Posteroventral end of the dentary
(0) not particularly expanded
(1) forming a well-developed process extending beyond the coronoid process

From Cavin ((70): ch. 33).

167. Anterodorsal ascending margin of the dentary
(0) continuous
(1) interrupted by a characteristic notch (so-called leptolepid notch)

From Arratia ((20): ch. 45 App. 3).

168. Coronoid process
(0) absent
(1) present

From Gardiner and Schaeffer ((12): ch. 17).

169. Coronoid process
(0) made up by the dentary and surangular
(1) made up by the dentary only
(2) made up by the surangular only
(3) made up by the surangular and angular

(4) made up by the dentary and angular

Modified from Gardiner et al. ((90): ch. 28) and Arratia ((11): ch. 69).

170. Surangular
(0) absent
(1) present

From Patterson ((61): ch. 33). Nybelin ((90)) described a single angulo-supra-angular bone, but Arratia ((21): p. 292) indicated an independent surangular in †*Dorsetichthys bechei*.

171. Independent articular bone
(0) single meckelian bone
(1) a single element
(2) two separate elements not in contact with each other
(3) absent

Modified from Patterson ((61): ch. 48) and Gardiner et al. ((38): ch. 17 App. 2). Arratia (11) scored an articular fused with the angular and retroarticular in †*Dorsetichthys bechei*, but Nybelin (90) describes an independent articular bone.

172. Postarticular process of lower jaw
(0) poorly developed or absent
(1) well developed, extending posterior to the articular facet for quadrate

From Arratia ((20): ch. 44 App. 3).

173. Prearticular bone in lower jaw
(0) present
(1) absent

From Patterson ((61): ch. 26).

174. Retroarticular
(0) absent
(1) present and separate
(2) present and fused to angular only
(3) present and fused to articular only
(4) present and fused to angular and articular

Modified from Diogo et al. ((91): ch. 247) and Sferco et al. ((10): ch. 68).

175. Retroarticular and quadrato-mandibular joint
(0) retroarticular excluded from the joint facet for quadrate
(1) retroarticular included in the joint facet for quadrate

From Patterson ((61): ch. 50).

176. Coronoid bone(s) in lower jaw
(0) present
(1) absent

From Patterson ((61): ch. 13). Previous authors score the absence of coronoid bones in the lower jaw of †*Dorsetichthys bechei*, but this feature has not been described so far. Grande (5) refers to Patterson ((61): pp. 626 and 629) for this feature in the taxon . However, regarding this character, Patterson ((61): character 13 on page 629) indicates the specimens number NHMUK PV P 44973–5 and P 3592, which are identified as †*Ichthyokentema purbeckensis* and †*Pholidophorus higginsi*, respectively.

177. Plicidentine structure in teeth
(0) absent

(1) present

From Wiley ((46): ch. 27).

178. Organization of teeth on premaxilla
(0) single row
(1) more than one row
(2) single row plus anterior fang

179. Relative size of largest premaxillary teeth
(0) of similar size of dentary teeth
(1) larger than dentary teeth
(2) smaller than dentary teeth

180. Maxillary teeth
(0) present
(1) absent

From Arratia ((20): ch. 39 App. 3).

181. Dentary teeth
(0) present
(1) absent

From López-Arbarello and Wencker ((40): ch. 88).

182. Tooth organization on dentary
(0) teeth in a single row and of similar size
(1) in addition to a lateral single row of similar sized teeth, there is a medial row of much larger fangs
(2) teeth of similar size arranged in two or more rows

From Grande ((5): ch. 39).

183. Extent of teeth on dentary (excluding coronoid toothplates)
(0) tooth row extends over at least a third the length of dentary
(1) tooth row is present on only the anterior one third or less of dentary

From Grande ((5): ch. 56).

184. Morphology of dentary teeth
(0) conical
(1) pointed pencil like
(2) high, bluntly pencil like
(3) molariform, broader than high
(4) conical with labiolingually compressed, sharply carinate (keeled) caps

Modified from Bermúdez-Rochas and Poyato-Ariza ((86): ch. 55).

185. Morphology of teeth on anterior coronoids
(0) conical
(1) pointed pencil like
(2) high, blunt, flattened or broadly rounded
(3) molariform, broader than high

Modified from Grande and Bemis ((9): ch. 16), which is a compound character encompassing the morphology of the teeth on the anterior coronoids and vomer in a single character. We think there is not necessarily a biological dependence in the morphology of the teeth in these different bones, which are here considered in our independent characters 185 and 186.

186. Morphology of vomerine teeth
(0) conical
(1) pointed pencil like
(2) blunt, flattened or broadly rounded
(3) molariform
(4) heterogeneous sharply pointed fangs

Opercular bones, branchiostegals and gular

187. Shape of preopercle
(0) long, angularly bent, with almost horizontal anterodorsal and vertical posteroventral portions (e.g. †*Pteronisculus* Nielsen (51): figs. 27, 30)
(1) plate-like, broad dorsally, narrowing ventrally (e.g. †*Plesiofuro* Xu et al. ((13): fig. 5)
(2) comma-shape (e.g. †*Ticinolepis longaeva* (71): figs. 7, 9-10, 12)
(3) roughly L-shape (e.g. †*Luisiella feruglioi* (73): fig. 4)

Several authors have considered one or other of our states as independent characters (e.g. (6): ch. 21, (9): ch. 36, (43): ch. 41, (3): ch. 60, (75): ch. 20, (11): ch. 90).

188. Median anterior process of preopercle
(0) absent
(1) present

In †*Boreosomus* ((51): fig. 69) and †*Australosomus* ((92): fig. 25) and several non-neopterygian taxa, the preopercle has a distinct acute process protruding in anterior direction from the anterior border of the bone.

189. Anterior/anteroventral end of preopercle
(0) broadly tapered (e.g. †*Ticinolepis longaeva* (71): figs. 9-10, 12)
(1) finely tapered (e.g. †*Occitanichthys canjuerensis* (40): fig. 5-6)

190. Relative length of preopercular arms
(0) vertical arm is longer than horizontal arm (e.g. †*Luisiella feruglioi* (73): fig. 4)
(1) horizontal arm is longer than vertical arm (e.g. *Lepisosteus osseus* (5): figs. 60–61)

191. Posteroventral angle of an L-shape preopercle
(0) not distinctly expanded (e.g. †*Luisiella feruglioi* (73): fig. 4)
(1) fan shape expanded (e.g. †*Tharsis dubius* (93): table 12, fig. 3)

192. Anteromedial wing of preopercle
(0) absent
(1) present

193. Anterior notch of preopercle
(0) absent
(1) present

Modified from Arratia ((11): ch. 88). The preopercle of some Triassic teleosts presents a notch directly at the anterior exit of the preopercular canal, directly at the edge between the anteromedial and lateral wings of the bone. Therefore, the character is not applicable for those taxa with a preopercle without anteromedial wing.

194. Exposure of dorsal limb of preopercle
(0) infraorbital or suborbital bones do not overlap, but only the anterior rim of preopercle
(1) suborbital bones completely overlap the dorsal limb of preopercle

From Grande ((5): ch. 73).

195. Preopercle to dermopterotic relationship
(0) preopercle reaches at least close to dermopterotic
(1) preopercle and dermopterotic are well separated

196. Posterior border of the preopercle notched ventrally
(0) absent
(1) present

From López-Arbarello ((3): ch. 62).

197. Suprapreopercle
(0) absent
(1) present

From Sferco et al. ((10): ch. 53).

198. Shape of the opercle width/length
(0) deeper than long
(1) approximately as deep as long
(2) tapering anteroventrally
(3) longer than deep

This character merges characters from several authors. Grande and Bemis ((9): ch. 36) use a character equivalent to the presence/absence of our state (3). López-Arbarello ((3): ch. 63) state 1 is equivalent to our states 1 and 3 together. Our state (2) is equivalent to Sferco et al. ((10): ch. 57).

199. Opercle ornamentation in adult-sized individuals
(0) densely arranged low tubercles and/or ridges
(1) well-defined tubercles
(2) pattern of ridges radiating from the anterodorsal corner
(3) denticles
(4) ornamentation weak or absent

Modified from Grande and Bemis ((9): ch. 33), which includes the a/p of our state (2) only. These authors score the pattern of radiating ridges for Vidalamiini only, but we find this pattern in all amiids.

200. Ascending process of subopercle
(0) absent
(1) present

From López-Arbarello ((3): ch. 64).

201. Base of ascending process of the subopercle
(0) broad, more than 30% of the maximal length of the bone
(1) narrow, between 10 and 30% of the maximal length of the bone
(2) tiny, less than 10% of the maximal length of the bone

Modified from López-Arbarello ((3): ch. 65).

202. Height ascending process of the subopercle
(0) low, 20% of the maximal length of the bone
(1) medium, 20-40% of the maximal length of the bone
(2) high, approximately 50-60% of the maximal length of the bone
(3) very high, approximately 70% or more the maximal length of the bone

Modified from López-Arbarello ((3): ch. 66). It is only possible to score characters 201 and 202 if a subopercle is at least partially disarticulated and the whole ascending process is exposed. Otherwise a variably large portion of the process is overlapped and thus hidden by the opercle.

203. Subopercle maximal depth (excluding ascending process)

(0) more than half the depth of the opercle
(1) less than half the depth of the opercle
(2) deeper than the opercle

From López-Arbarello ((3): ch. 67).

204. Interopercle
(0) absent
(1) present

From Gardiner and Schaeffer ((12): ch. 18).

205. Relationship between interopercle and the lower jaw
(0) anterior end of interopercle close to mandible
(1) interopercle remote from mandible

Modified from Olsen and McCune ((15): ch. 11).

206. Branchiopercle
(0) absent
(1) present

The branchiopercle is generally distinguished because of its size, which is notably larger than the largest branchiostegal bone in most taxa. Additionally, the branchiopercle in *Amia calva* develops in a fold continuous with the lower edge of the lower jaw (94) and it is not only attached to the hyoid arch (anterior ceratohyal), but also to the lower jaw in adult specimens (9). Accordingly, the branchiopercle is aligned with the main axis of the lower jaw.

207. Number of branchiostegal rays
(0) 20 or more
(1) 15 to 20
(2) 10 to 15
(3) 6 to 9
(4) 3 or 4

The number of branchiostegals is used as a character in previous cladistic analyses (e.g. (9): ch. 54, (56): ch. 12, (5): ch. 74), but we here use different ranges for the character states.

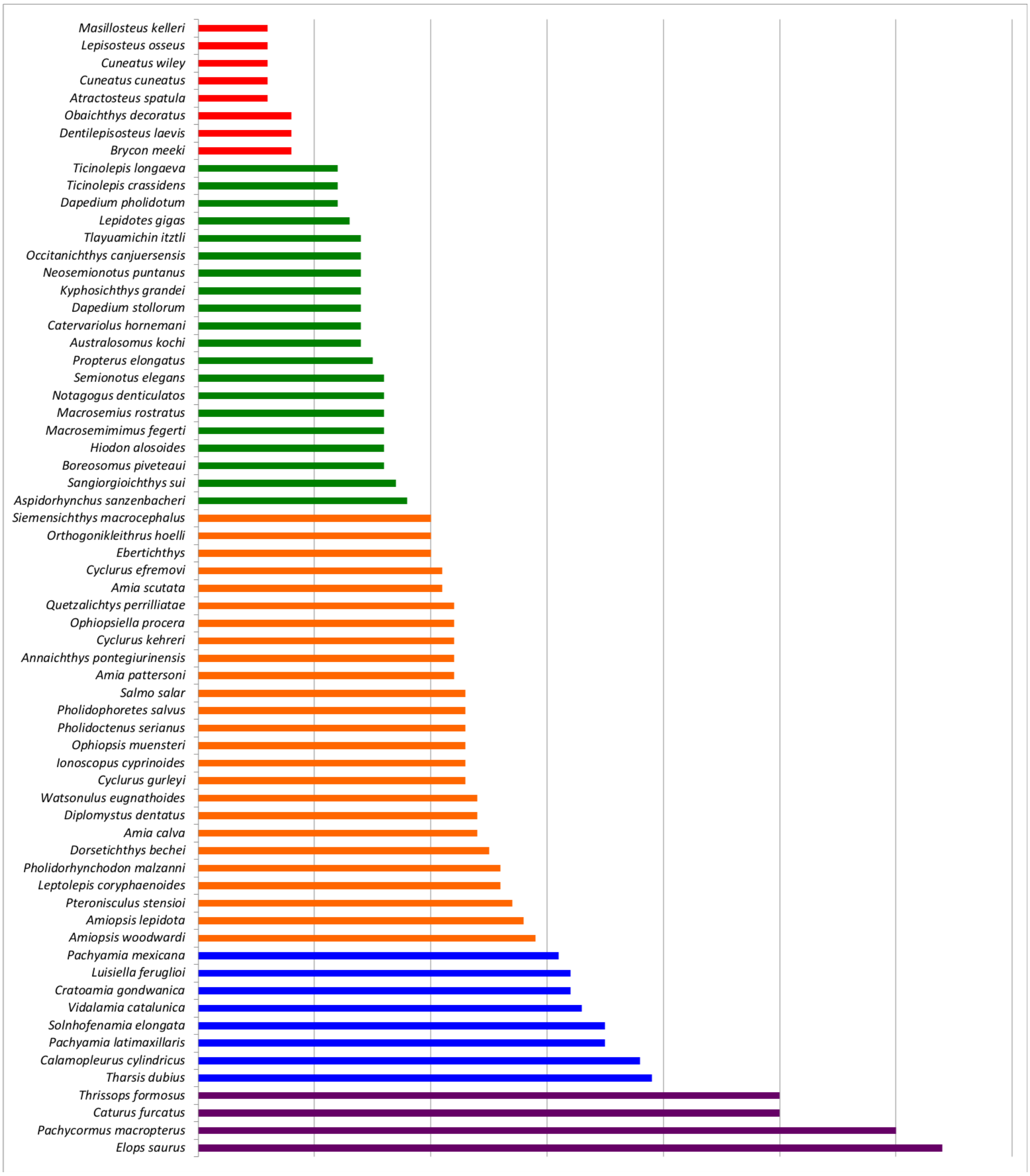

Figure S2-3: Average number of branchiostegal rays in 65 studied taxa. Different colors represent different character states: purple (0), blue (1), orange (2), green (3), and red (4).

208. Median gular
(0) absent
(1) present

From Olsen and McCune ((15): ch. 8).

Brito (6) scored absence of gulars for †*Ichthyokentema*, but †*I. purbeckensis* has a broad median gular according to Griffith and Patterson ((95): p. 19, text-fig. 7). Coates ((56): ch. 11 state 1) scores “lateral gulars equivalent to area of three branchiostegals or less, with posterior and lateral angles” for †*Watsonulus*, but there is single median gular in †*W. eugnathoides* (96).

209. Gular maximal with to maximal length ratio
(0) higher than 0.5

(1) between 0.3 and 0.5
(2) lower than 0.3

Modified from Grande and Bemis ((9): ch. 32).

Branchial arches

210. Basibranchials tooth plates
(0) absent
(1) present and paired
(2) present and median

From Sferco et al. ((10): ch. 86).

211. Number of hypobranchials
(0) three
(1) four

From Grande ((5): ch. 99).

212. Infrapharyngobranchials tooth plates
(0) absent
(1) present on infrapharyngobranchials 2 and 3 only
(2) present on infrapharyngobranchials 1 to 3
(3) present on infrapharyngobranchial 3 only

This character is the result of merging Sferco et al. ((10): chs. 87–89).

213. Epibranchials
(0) slender
(1) with uncinate processes

From Gardiner and Schaeffer ((12): ch. 15).

Sensory canals

214. Junction of supraorbital and temporal canals
(0) no direct connection between the temporal (otic) and supraorbital sensory canals
(1) direct connection between the temporal (otic) and supraorbital sensory canals

From Cavin ((35): ch. 7).

215. Position of junction of supraorbital and temporal canals
(0) exclusively within frontal bone
(1) exclusively within dermosphenotic bone
(2) exclusively within dermopterotic bone

Modified from Cavin et al. ((49): ch. 24).

216. Supraorbital sensory canal in parietal
(0) present
(1) absent

From Grande and Poyato-Ariza ((37): ch. 11).

217. Middle pit line
(0) leaving a groove or pore-line on the parietal and dermopterotic
(1) leaving a groove or pore-line on the parietal only
(2) in the dermopterotic only
(3) leaving no trace on the bones

Modified from Sferco et al. ((10): ch. 79).

218. Position of the supratemporal commissure
(0) pierces extrascapulars
(1) pierces extrascapulars and parietals
(2) pierces parietals only, or parietals and supraoccipital
(3) pierces dermopterotics

Modified from Arratia ((20): ch. 26 App. 3). In our data matrix, states 2 and 3 are autapomorphic for *Brycon* and †*Pachycormus*, respectively.

219. Orbital canal
(0) absent
(1) present

From López-Arbarello ((3): ch. 89).

220. Sensory canal in maxilla
(0) absent
(1) present

From Gardiner et al. ((38): ch. 24 App. 2).

221. Composition of preopercular sensory canal
(0) four or less, short simple tubules
(1) seven to 10, short simple or branched tubules
(2) 12 or more long, simple or branched tubules

From Sferco et al. ((10): ch. 83).

222. Posttemporal penetration by lateral line canal
(0) present
(1) absent

From Grande ((5): ch. 91).

223. Lateral line ossicles between caudal fin rays
(0) absent
(1) present

From Alvarado-Ortega and Espinosa-Arrubarrena ((78): ch. 13).

Vertebral column

224. Shape of vertebral centrum
(0) amphicoelous
(1) opisthocoelous

From Wiley ((46): ch. 26). This character is only scored for those taxa with solidly ossified vertebra because it is not applicable otherwise.

225. Arcocentrum
(0) absent
(1) present and thin, ring-like centra
(2) present and forming solidly ossified centra

226. Chordacentrum
(0) absent
(1) present

227. Autocentrum
(0) absent
(1) present

228. Diplospondylous centra in caudal region
(0) absent
(1) present

From Gardiner et al. ((38): ch. 11 App. 2). Grande and Bemis ((9): ch. 1) is a compound character (2) combining the diplospondily (this character) with the mode of ossification of the centra.

229. Surface of arcocentra
(0) sculptured with two pits
(1) sculptured with three or more pits
(2) smooth

The morphology of the lateral walls of the vertebral centra has been used as a character by several authors (e.g. (9): ch. 4, (36): ch. 59, (11): ch. 98, (14): ch. 3, (10): ch. 92), but the surface of a centrum might be formed by different tissues. Our character 229 is thus limited to the morphology of the superficial arcocentra and the next character 230 refers to the superficial morphology of the autocentra.

230. Surface of autocentra
(0) smooth
(1) sculptured

231. Large parapophyses
(0) absent
(1) present, not fused to the centra
(2) present, fused to the centra

Modified from Grande and Bemis ((9): ch. 10) by adding our state (1). The taxa presenting state (1) in our data matrix are scored with state 0 in the data matrix of Grande and Bemis (9).

The parapophyses are large in *Hiodon alosoides*, but they represent a different condition, which is uniquely present in this taxon in our data matrix. The parapophyses in this species are spine like and the ribs do not articulate with their distal tips, but they articulate dorsally, at the base of the parapophyses. Therefore, the character is not applicable (-) for this taxon.

232. Morphology of pleural ribs
(0) distal ends pointed or with rounded points
(1) distal ends flatly truncated, even in large adults

From Grande and Bemis ((9): ch. 31).

233. Relative shape of abdominal neural arches (lateral view)
(0) tapering distally
(1) distally expanded, hourglass-shaped

From Sferco et al. ((10): ch. 99).

234. Relationship between the abdominal neural arches and vertebral centra
(0) neuual arches attached to the centra but not fused to them
(1) all or most of the neural arches fused to the centra

From Sferco et al. ((10): ch. 94). In the holospondylous opisthocoelous vertebrae of gars, the neural arches are primarily continuous with the vertebral centra because the basidorsal arcualia directly participates in the formation of the centrum.

235. Neural spines of abdominal vertebrae

(0) paired
(1) median

From Sferco et al. ((10): ch. 96).

236. Infrahaemals
(0) absent
(1) present

The infrahaemal bones are median spine-shaped bones articulating with the distal end of haemal arches, but not fused to them (Goodrich (97)). The possible homology between infrahaemals and neural spines is still unclear. The number of infrahaemals is intraspecifically variable.

237. Supraneural bones
(0) absent
(1) present

From Sferco et al. ((10): ch. 101).

238. Distribution of supraneural bones
(0) extend below dorsal fin
(1) the series of supraneurals ends anterior to dorsal fin

239. Shape of most anterior supraneurals
(0) simple rod-like bones
(1) with membranous expansions in the longitudinal plane

From Cavin et al. ((36): ch. 57). In the living gars, the first one or two supraneurals are reduced in size and are comparatively more robust than the following supraneurals, but they are still rod-like and do not form membranous expansions.

240. Epineural bones
(0) absent
(1) present

From Sferco et al. ((10): ch. 102).

241. Epipleural bones
(0) absent
(1) present

From Sferco et al. ((10): ch. 103).

242. Position of epipleural bones relative to the column
(0) located in anterior caudal region
(1) located in abdominal and anterior caudal region

From Sferco et al. ((10): ch. 104).

243. Relationship between caudal neural arches and vertebral centra (excluding the last few preural vertebrae)
(0) unfused
(1) fused

244. Shape of preural neural spines
(0) spine-like or rod-like
(1) broad with a median groove
(2) broad due to anterior and posterior membranous outgrowths

245. Shape of the preural haemal spines

(0) spine-like or rod-like
(1) broad with a median groove
(2) broad due to anterior and posterior membranous outgrowths

246. Haemal spines spatulate in the transverse plane
(0) absent
(1) present

From Grande and Bemis ((9): ch. 65).

247. Relative length of neural spine on second preural vertebra (Pu2)
(0) as long as neural spine on third preural vertebra
(1) shorter than neural spine on third preural vertebra

From Sferco et al. ((10): ch. 129).

248. Abrupt change in the orientation of preural caudal haemal spines
(0) absent
(1) present

Modified from Grande and Bemis ((9): ch. 67).

249. Abrupt change in the orientation of preural caudal neural spines
(0) absent
(1) present

Modified from Grande and Bemis ((9): ch. 67).

250. Anteriorly projecting spine-like processes on caudal neural arches
(0) absent
(1) present

Modified from Grande and Bemis ((9): ch. 3).

251. Anteriorly projecting spine-like processes on caudal haemal arches
(0) absent
(1) present

Modified from Grande and Bemis ((9): ch. 3).

252. Anterior zygapophyses in neural arches of caudal vertebrae
(0) absent
(1) present

253. Anterior zygapophyses in haemal arches of caudal vertebrae
(0) absent
(1) present

254. Posterior zygapophyses in haemal arches of caudal vertebrae
(0) absent
(1) present

Characters 252 to 254 are exemplified in (29): fig. 22C.

255. Neural arch on preural centrum 1 (Pu1)
(0) with neural spine
(1) without neural spine

Modified from Sferco et al. ((10): ch. 132).

256. Neural spine on preural centrum 1 (Pu1)
(0) as long as preceding neural spines
(1) shorter than preceding neural spines

Modified from Sferco et al. ((10): ch. 132).

257. Condition of preural haemal arches (excluding centrum Pu1) in relation to their respective centra
(0) unfused
(1) fused

Modified from Sferco et al. ((10): ch. 133).

258. Relationship between parhypural and centrum Pu1
(0) unfused
(1) fused

Modified from Sferco et al. ((10): ch. 134).

259. Number of ural centra
(0) 11 to 22
(1) 4 to 10
(2) three

Modified from Gardiner et al. ((38): ch. 32 App. 2).

260. Fusions between ural centra
(0) absent, all centra separated
(1) only first and second ural centra fused
(2) all fused forming an urostyle

In our data matrix, state 2 is autapomorphic for *Brycon meeki*.

261. Relationship between centra Pu1 and U1
(0) independent from each other
(1) fused, forming a compound centrum

From Sferco et al. ((10): ch. 155).

262. Compound neural arch over preural centrum 1 and first ural centrum
(0) absent
(1) present

From Sferco et al. ((10): ch. 140).

263. Number of ossified ural neural arches
(0) normally four or more
(1) three
(2) normally two
(3) one
(4) none

Modified from Gardiner et al. ((38): ch. 10 App. 2).

264. Number of ural neural spines
(0) four or more
(1) three
(2) two
(3) one
(4) none

265. Epural bones
(0) absent
(1) present

Modified from Sferco et al. ((10): ch. 142).

266. Number of epurals
(0) four or more
(1) three
(2) two
(3) one

Modified from Sferco et al. ((10): ch. 142).

267. Series of 'epurals' in caudal region
(0) absent
(1) present

268. Number of hypurals
(0) eight or more
(1) seven
(2) six or less

From Sferco et al. ((10): ch. 151).

269. Relationship between H1 and H2
(0) independent from each other
(1) fused at their bases only
(2) fused to each other along their length

From Sferco et al. ((10): ch. 154).

270. Hypural-ural centra fusion in adult-sized individuals
(0) all hypurals autogenous (separate) from the centra
(1) all but first hypural fused to the centra
(2) first and second hypurals separate, other hypurals fused
(3) first and second hypurals fused, other hypurals separate
(4) only second hypural fused, other hypurals separate
(5) only first hypural fused, other hypurals separate
(6) all hypurals fused to the ural centra

Modified from Grande and Bemis ((9): ch. 9).

271. A space or diastema between H2 and H3
(0) absent
(1) present

From Sferco et al. ((10): ch. 156).

272. Stegural
(0) absent
(1) present

From Sferco et al. ((10): ch. 149).

273. Uroneurals (Fig. 7)
(0) absent
(1) present

From Gardiner et al. ((38): ch. 35 App. 1).

274. Number of uroneurals
(0) six or more
(1) five or four
(2) three or less

From Sferco et al. ((10): ch. 143).

275. 'Posterior uroneurals' (Fig. 7)
(0) present
(1) absent

276. Anterior extent of first uroneural
(0) preural centra 4 or 3
(1) preural centrum 2
(2) preural centrum 1
(3) does not extend anteriorly beyond the corresponding centrum

From Sferco et al. ((10): ch. 145).

277. Number or uroneurals extending forwards beyond second ural centrum (U2)
(0) three or four
(1) two
(2) one
(3) none

From Sferco et al. ((10): ch. 146).

Median fins

278. Shape of posterior margin of caudal fin
(0) forked
(1) convexly rounded
(2) concave

Modified from Grande and Bemis ((9): ch. 23).

279. One-to-one arrangement of hypurals and caudal fin rays
(0) absent
(1) present

From Gardiner et al. ((38): ch. 9 App. 2).

280. Mode of articulation of the central and ventral principal caudal fin rays
(0) hemitrichia placed distally, only slightly or not overlapping endoskeletal supports
(1) hemitrichia placed laterally, overlapping the endoskeletal supports

281. Arrangement of dorsal principal rays of caudal fin and dorsal hypurals
(0) aligned with main axis of hypurals
(1) oriented oblique to main axis of hypurals

Modified from Gardiner et al. ((38): ch. 34 App. 1).

282. Total number of principal caudal fin rays
(0) variable, more than 50
(1) variable, between 19 and 50
(2) stable at 19
(3) variable, up to 19

Modified from Sferco et al. ((10): ch. 163).

In *Amia*, there is a rather clear distinction between procurrent and principal rays on the dorsal margin, but not in the ventral margin where the rays increase in length gradually. We take the first branched ray as the first ventral principal ray.

283. Number of rays forming the dorsal margin of the caudal fin
(0) more than two (branched or not)
(1) two, at least the first unbranched
(2) one branched
(3) one unbranched

284. Number of principal caudal fin rays below lateral line
(0) more than eight
(1) eight
(2) seven
(3) six

Modified from López-Arbarello and Wencker ((40): ch. 76). This character merges characters 80 and 81 of López-Arbarello (3) because they are not independent, but represent different states of the same feature.

285. Number of principal caudal fin rays associated to the preural skeleton
(0) more than nine
(1) seven or eight
(2) four to six
(3) one to three

Modified from Sferco et al. ((10): ch. 171).

286. Number of rays forming the ventral margin of the caudal fin
(0) two or more (branched or not)
(1) one branched
(2) one unbranched

287. Dorsal processes of the bases of innermost principal caudal fin rays
(0) absent
(1) present

From Sferco et al. ((10): ch. 168).

288. Shape of the bases of innermost principal caudal fin rays
(0) narrow, with pointed proximal end
(1) expanded, with crenulated anterior margin

From Sferco et al. ((10): ch. 169).

289. Type of segmentation of marginal principal caudal rays
(0) straight
(1) 'Z' or step-like

From Sferco et al. ((10): ch. 170).

290. Number of hypaxial procurrent (segmented) caudal fin rays
(0) none
(1) one
(2) two to five
(3) six to eleven

291. Epaxial basal fulcra (not segmented)
(0) present and scale-like

(1) present as procurrent rays

292. Hypaxial basal fulcra (not segmented)
(0) present
(1) absent

From Sferco et al. ((10): ch. 160).

293. Accessory row of scales adjacent to the ventral border of the body lobe
(0) absent
(1) present, one row
(2) present, two or more rows

Modified from López-Arbarello ((3): ch. 82).

294. Urodermals
(0) presence of a complete body lobe
(1) present, few, isolated
(2) absent

From López-Arbarello et al. ((14): ch. 12). Grande and Bemis ((9): ch. 13) used a similar character, but did not distinguish character state 2, which represents a condition that they include in their character state 0 (presence of urodermals).

295. Tendon-bones (i.e., 'urodermals'; Fig. 7) associated with dorsalmost principal caudal rays
(0) absent
(1) present

Modified from Sferco et al. ((10): ch. 174).

296. Dorsal scute(s) preceding caudal fin
(0) present, more than one
(1) present, single
(2) absent

Modified from Sferco et al. ((10): ch. 158). The homologies between these isolated dorsal and ventral scutes preceding the caudal fin of teleosts and many halecomorphs are still unknown. These scutes might be homologous with the dorsal caudal fulcra or the scutes of preceding the series of dorsal caudal fulcra in several actinopterygians. Therefore, this and the next character (297) are inapplicable for those taxa with a complete series of dorsal caudal fulcra.

297. Ventral scute(s) preceding caudal fin
(0) present, more than one
(1) present, single
(2) absent

298. Fringing fulcra on caudal fin
(0) present on both dorsal and ventral margins
(1) present on dorsal margin only
(2) absent

Grande and Bemis ((9): ch. 45) code the presence/absence of fringing fulcra in all median fins in a single character. We distinguish the presence of fringing fulcra in the caudal fin (this character) from their presence in the dorsal and anal fin in an independent character (315), which has a different distribution.

299. Number of fringing fulcra on the first principal caudal fin ray
(0) numerous
(1) one to five

Modified from Sferco et al. ((10): ch. 162).

300. Relationship between dorsal and anal fin rays and radials

(0) fin rays more numerous than radials

(1) one to one relationship except for the most anterior radial

From Gardiner and Schaeffer ((12): ch. 25).

301. Number of dorsal fin rays

(0) 20 or less

(1) 21 to 34

(2) 36 to 42

(3) 43 or more

Modified from Grande and Bemis ((9): ch. 15). The ranges proposed in the character states represent the variation observed in our data set (Fig. S2-3).

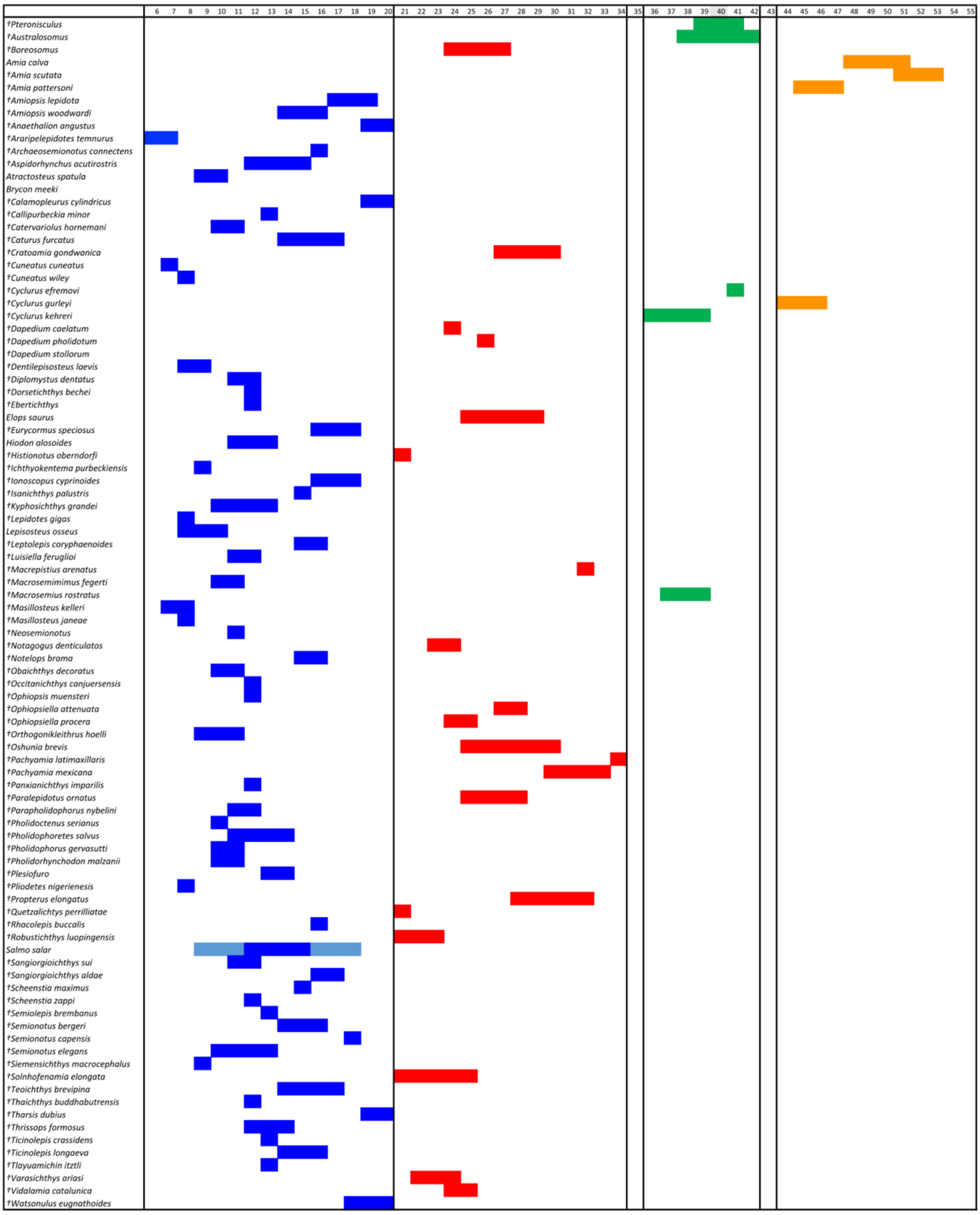

Figure S2-4: Number of dorsal fin rays in 94 studied taxa. The horizontal coloured bars indicate the range of variation within each taxon and the different colours (blue, red, green, orange) correspond to the four character states.

302. Shape of dorsal fin
(0) triangular
(1) anterior rays distinctly higher forming a sickle-shaped margin
(2) bow-shaped
(3) divided in two sections

Modified from Grande and Bemis ((9): ch. 15).

303. First dorsal proximal pterygiophore
(0) simple structure
(1) bipartite or tripartite

From Sferco et al. ((10): ch. 119).

304. Relationship between first dorsal proximal pterygiophore and fin rays
(0) articulates with procurrent rays or basal fulcra and first principal ray
(1) articulates with procurrent rays or basal fulcra only

From Sferco et al. ((10): ch. 120).

305. First principal dorsal ray
(0) branched
(1) unbranched

306. Number of anal fin rays
(0) less than 20
(1) 20 – 35
(2) more than 35

The ranges proposed in the character states represent the variation observed in our data set (Fig. S2-4).

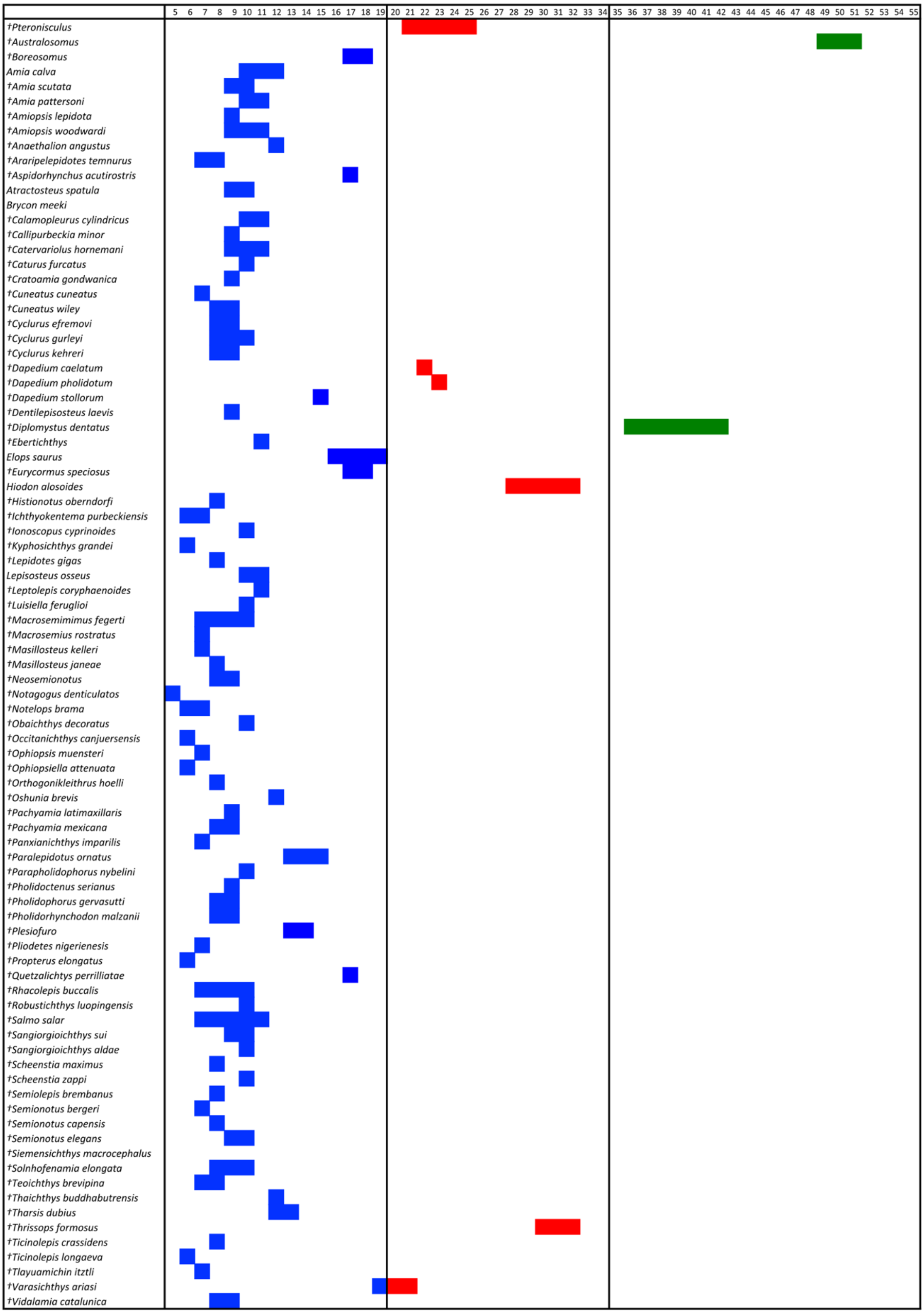

Figure S2-5: Number of anal fin rays in 87 studied taxa. The horizontal coloured bars indicate the range of variation within each taxon and the different colours (blue, red, green) correspond to the three character states.

307. Shape of anal fin

(0) triangular
(1) anterior rays distinctly deeper forming a sickle-shaped margin
(2) bow-shaped or straight

Modified from Sferco et al. ((10): ch. 122).

308. Relative position of first anal proximal pterygiophore
(0) posterior to first haemal spine or infrahaemal
(1) at first haemal spine or infrahaemal
(2) anterior to first haemal spine or infrahaemal

Modified from Sferco et al. ((10): ch. 123).

309. Relationship between first anal pterygiophore and fin rays
(0) articulates with procurrent rays or basal fulcra and first principal ray
(1) articulates with procurrent rays or basal fulcra only

310. First principal anal fin ray
(0) branched
(1) unbranched

311. Basal fulcra in the dorsal and anal fins
(0) absent
(1) present

312. Relative size of the basal fulcra in the dorsal fin
(0) slender, equalling or more slender than the basal segment of the first dorsal fin ray
(1) stout and short, shorter than the basal segment of the first dorsal fin ray
(2) stout and increasing up to relatively high, higher than the basal segment of the first dorsal fin ray
(3) ray-like

313. Relative size of the basal fulcra in the anal fin
(0) slender, equalling or more slender than the basal segment of the first anal fin ray
(1) stout and short, shorter than the basal segment of the first anal fin ray
(2) stout and increasing up to relatively high, higher than the basal segment of the first anal fin ray
(3) ray-like

Character 312 and 313 are modified from López-Arbarello ((3): ch. 78).
The basal fulcra in basal neopterygians are slender scale-like structures (state 0 of chs. 312 and 313) preceeding the bases of the fin rays. The more posterior basal fulcra are increasingly higher, but reaching as much as the height of the base of the first principal ray (e.g. (71): fig. 15). In most lepisosteiforms, the basal fulcra are comparatively shorter, their maximal height not reaching the height of the basal segment of the first principal ray (state 1 of chs. 312 and 313; e.g. (5): figs. 96, 100). However, the derived condition at the base of Neoginglymodi is the presence of notably large basal fulcra. In these fishes, the most anterior basal fulcrum is usually short, but the more posterior elements are rapidly increasing in height ant the most posterior basal fulcrum is higher than the base of the adjacent fin ray (state 2 of chs. 312 and 313; e.g. (62): fig. 9A; (40): fig. 9A). Independently in amiids and macrosemiids, the basal fulcra are extremely slender and ray-like (state 3 of chs. 312 and 313; e.g. (9): figs. 83, 86, indicated as rudimentary fin rays).

314. Preanal scute
(0) absent
(1) present, smooth surface and borders
(2) present, smooth surface, indented border

315. Fringing fulcra on dorsal and anal fins

(0) present
(1) absent

Modified from Grande and Bemis ((9): ch. 45).

316. Pattern of fringing fulcra on median fins
(0) formed by a combination of small, spiny bony fringing fulcra positioned between the terminal segments of the procurrent and/or marginal rays
(1) formed by a combination of small, spiny bony fringing fulcra positioned between the terminal segments of the procurrent and subsequently lying on the surface of the marginal ray(s)
(2) formed of small, paired, spine-like elements lying on the surface of the marginal ray(s) only

Modified from Coates ((56): ch. 61).

317. Relative height of the posttemporal bone
(0) high, reaching or almost reaching the dorsal midline
(1) low, approximately as high as the dermopterotic
(2) posttemporals very small

From López-Arbarello and Wencker ((40): ch. 91).

318. Anterior process of posttemporal bone
(0) absent
(1) present, blunt
(2) present, large and knob-like

Modified from López-Arbarello ((3): ch. 72).

319. Lateral edge of posttemporal in adult-sized individuals
(0) shorter than length of anterior edge
(1) elongate, about equal to or greater than width of anterior edge

From Grande and Bemis ((9): ch. 22).

320. Presupracleithrum
(0) absent
(1) present

From Gardiner et al. ((98): ch. 38).

321. Supracleithrum with a concave articular facet for articulation with the posttemporal
(0) absent
(1) present

From Grande ((5): ch. 93).

322. Relationship between lateral line system and supracleithrum
(0) emerging at the middle
(1) emerging at its upper half
(2) emerging at its lower half

Modified from Arratia ((20): ch. 70 App. 3).

323. General shape of cleithrum
(0) sickle to L-shape with approximately equally large vertical and horizontal portions
(1) sickle to L-shape with horizontal portion 1,5 to 2 times larger than vertical portion
(2) sickle without clearly distinct vertical and horizontal portions
(3) sickle with anterior portion bent downwards

324. Medial wing of cleithrum
(0) absent
(1) present

Modified from Grande ((5): ch. 94).

325. Ornamentation of the cleithrum
(0) absent
(1) one or more series of toothed ridges on the anterolateral surface

Modified from López-Arbarello ((3): ch. 74).

326. Number of postcleithra
(0) none
(1) one
(2) two
(3) three
(4) four
(5) five
(6) six or more

Modified from Sferco et al. ((10): ch. 108).

327. Clavicles
(0) present
(1) absent

From Olsen and McCune ((15): ch. 24).

328. Independent serrated appendages
(0) absent
(1) present

From Grande ((5): ch. 96). The serrated appendages have been considered homologous with the clavicle of other actinopterygians (19, 99-101). Accordingly, clavicles are present (327[0]) in those taxa with serrated appendages and character 328 is innaplicable for those taxa without clavicles.

329. Scapulocoracoid ossification in adult-sized individuals
(0) one or more elements present in the shoulder girdle
(1) absent

From Grande and Bemis ((9): ch. 11).

330. Coracoid bones
(0) small, separated from each other at the midline
(1) large, ventrally meeting its fellow in a midventral coracoid symphysis

From Sferco et al. ((10): ch. 109).

331. Relationship between pectoral propterygium and first pectoral ray
(0) unfused
(1) fused

From Sferco et al. ((10): ch. 110).

332. Number of proximal pectoral radials
(0) five or more
(1) four

From Sferco et al. ((10): ch. 111).

333. Bifurcation pattern of pectoral fin rays
(0) symmetric
(1) asymmetrtic, resulting in two unequal secondary rays

From Sferco et al. ((10): ch. 112).

334. Fringing fulcra on pectoral fin
(0) present
(1) absent

From López-Arbarello ((3): ch. 75).

335. Pectoral axillary process
(0) absent
(1) present

From Sferco et al. ((10): ch. 113).

336. Shape of basipterygium
(0) proximal end flat and widened anteriorly
(1) proximal end rodlike, without significant widening anteriorly
(2) approximately triangular, tapering anteriorly

Modified from Grande and Bemis ((9): ch. 29).

337. Fringing fulcra on pelvic fin
(0) present
(1) absent

From López-Arbarello ((3): ch. 76).

338. Spatulate fin rays
(0) absent
(1) in pectoral fins only
(2) in pelvic fins only
(3) in pectoral and pelvic fins

339. Pelvic axillary process
(0) absent
(1) present

From Sferco et al. ((10): ch. 114).

References

1. Jenner RA. Boolean logic and character state identity: pitfalls of character coding in metazoan cladistics. Contributions to Zoology. 2002;71(1-3):67-91.
2. Brazeau MD. Problematic character coding methods in morphology and their effects. Biological Journal of the Linnean Society. 2011;104(3):489-98.
3. López-Arbarello A. Phylogenetic Interrelationships of Ginglymodian Fishes (Actinopterygii: Neopterygii). Plos One. 2012;7(7).
4. Thies D, Waschkewitz J. Redescription of *Dapedium pholidotum* (Agassiz, 1832) (Actinopterygii, Neopterygii) from the Lower Jurassic Posidonia Shale, with comments on the phylogenetic position of *Dapedium* Leach, 1822. Journal of Systematic Palaeontology. 2016;14(4):339-64.
5. Grande L. An empirical synthetic pattern study of gars (Lepisosteiformes) and closely related species, based mostly on skeletal anatomy. The resurrection of Holostei. American Society of Ichthyologists and Herpetologists, Special Publication. 2010(6):1-863.

6. Brito PM. Révision des Aspidorhynchidae (Pisces, Actinopterygii) du Mésozoïque: ostéologie, relations phylogénétiques, données environnmentales et biogéographiques. Geodiversitas. 1997;19(4):681-772.
7. Lopez-Arbarello A, Schroeder KM. The species of *Aspidorhynchus* Agassiz, 1833 (Neopterygii, Aspidorhynchiformes) from the Jurassic plattenkalks of Southern Germany. Palaeontologische Zeitschrift. 2014;88(2):167-85.
8. Sire JY, Huysseune A. Formation of dermal skeletal and dental tissues in fish: a comparative and evolutionary approach. Biological Reviews. 2003;78(2):219-49.
9. Grande L, Bemis WE. A comprehensive phylogenetic study of amiid fishes (Amiidae) based on comparative skeletal anatomy: An empirical search for interconnected patterns of natural history. Memoirs of the Society of Vertebrate Paleontology. 1998;4:I-X, 1-690.
10. Sferco E, López-Arbarello A, Báez AM. Phylogenetic relationships of †*Luisiella feruglioi* (Bordas) and the recognition of a new clade of freshwater teleosts from the Jurassic of Gondwana. Bmc Evolutionary Biology. 2015;15:Article Number 268.
11. Arratia G. Morphology, taxonomy, and phylogeny of Triassic pholidophorid fishes (Actinopterygii, Teleostei). Journal of Vertebrate Paleontology. 2013;33:1-138.
12. Gardiner BG, Schaeffer B. Interrelationships of lower actinopterygian fishes. Zoological Journal of the Linnean Society. 1989;97:135-87.
13. Xu GH, Gao KQ, Coates MI. Taxonomic revision of *Plesiofuro mingshuica* from the Lower Triassic of Northern Gansu, China, and the relationships of early neopterygian clades. Journal of Vertebrate Paleontology. Journal of Vertebrate Paleontology. 2015;35(6):14.
14. López-Arbarello A, Stockar R, Bürgin T. Phylogenetic Relationships of the Triassic *Archaeosemionotus* Deecke (Halecomorphi, Ionoscopiformes) from the 'Perledo Fauna'. Plos One. 2014;9(10).
15. Olsen PE, McCune AR. Morphology of the *Semionotus elegans* species group from the Early Jurassic part of the Newark Supergroup of eastern North America with comments on the Family Semionotidae (Neopterygii). Journal of Vertebrate Paleontology. 1991;11(3):269-92.
16. McCune AR. Toward the phylogeny of a fossil species flock: semionotid fishes from a lake deposit in the Early Jurassic Towaco Formation, Newark Basin. Bulletin of the Peabody Museum of Natural History. 1987;43:1-108.
17. Bjerring HC. The term "Fossa Bridgei" and five endocranial fossae in teleostome fishes. Zoologica Scripta. 1984;13(3):231-138.
18. Patterson C. The braincase of pholidophorid and leptolepid fishes, with a review of the actinopterygian braincase. Philosophical Transactions of the Royal Society of London. 1975;269(899):275-579.
19. Sagemehl M. Beitrage zur vergleichenden Anatomie der Fische. 1 . Das cranium von *Amia calva* L. Morphologisches Jahrbuch. 1884;9:177-228.
20. Arratia G. Basal teleosts and teleostean phylogeny. Palaeo Ichthyologica. 1997;7:5-168.
21. Arratia G. The monophyly of Teleostei and stem-group teleosts. Consensus and disagreements. In: Arratia G, Schultze H-P, editors. Mesozoic Fishes 2 Systematics and Fossil Record. München: Verlag Dr. Friedrich Pfeil; 1999. p. 265-334.
22. Arratia G. The varasichthyid and other crossognathiform fishes, and the Break-up of Pangaea. In: Longbottom C, L., Richter M, editors. Fishes and the Break-up of Pangaea. 295: Geological Society Special Publications; 2008. p. 71-92.
23. Arratia G. New teleostean fishes from the Jurassic of southern Germany and the systematic problems concerning the 'pholidophoriforms'. Paläontologische Zeitschrift. 2000;74(1/2):113-43.
24. Arratia G. Remarkable teleostean fishes from the Late Jurassic of southern Germany and their phylogenetic relationships. Mitteilungen aus dem Museum für Naturkunde in Berlin, Geowissenschaftliche Reihe. 2000;3:137-79.
25. Arratia G. New remarkable Late Jurassic teleosts from southern Germany: Ascalaboidae n. fam., its content, morphology, and phylogenetic relationships. Fossil Record. 2016;19(1):31-59.

26. Arratia G. New Triassic teleosts (Actinopterygii, Teleosteomorpha) from Northern Italy and their phylogenetic relationships among the most basal teleosts. Journal of Vertebrate Paleontology. 2017;37(2):24.
27. Arratia G, Tischlinger H. The first record of Late Jurassic crossognathiform fishes from Europe and their phylogenetic importance for teleostean phylogeny. Fossil Record. 2010;13(2):317-41.
28. Weitzman SH. The osteology of *Brycon meeki*, a generalized characid fish, with an osteological definition of the family: Division of Systematic Biology, Stanford University; 1962.
29. Forey PL. The osteology of *Notelops* Woodward, *Rhacolepis* Agassiz and *Pachyrhizodus* Dixon (Pisces: Teleostei). Bulletin of the British Museum (Natural History) - Geology. 1977;28(2):123-204.
30. Maisey JG. Santana Fossils: An Illustrated Atlas. Maisey JG, editor. Neptune City: T.F.H. Publications, Inc.; 1991 //. 1-455 p.
31. Hilton EJ. Osteology of the Extant North American Fishes of the Genus *Hiodon* Lesueur, 1818 (Teleostei: Osteoglossomorpha: Hiodontiformes). Fieldiana: Zoology, New Series. 2002;100:1-142.
32. Patterson C, Rosen DE. Review of ichthyodectiform and other Mesozoic teleost fishes and the theory and practice of classifying fossils. Bulletin of the American Museum of Natural History. 1977;158(2):81-172.
33. Alvarado-Ortega J. Description and relationships of a new Ichthyodectiform fish from the Tlayúa Formation (Early Cretaceous: Albian), Puebla, Mexico. Journal of Vertebrate Paleontology. 2004;24(4):802-13.
34. Stewart JD. A new genus of Saurodontidae (Teleostei: Ichthyodectiformes) from the Upper Cretaceous rocks of the Western Interior of North America. 1999:335-60.
35. Cavin L. Osteology and phylogenetic relationships of the teleost *Goulmimichthys arambourgi* Cavin, 1995, from the Upper Cretaceous of Goulmima, Morocco. Eclogae geolHelv. 2001;94:509-35.
36. Cavin L, Forey PL, Giersch S. Osteology of *Eubiodectes libanicus* (Pictet & Humbert, 1866) and some other ichthyodectiformes (Teleostei): phylogenetic implications. Journal of Systematic Palaeontology. 2013;11(2):115-77.
37. Grande T, Poyato-Ariza FJ. Phylogenetic relationships of fossil and Recent gonorynchiform fishes (Teleostei: Ostariophysi). Zoological Journal of the Linnean Society. 1999;125:197-238.
38. Gardiner BG, Maisey JG, Littlewood DTJ. Interrelationships of Basal Neopterygians. 1996;Chapter 6:117-46.
39. Maisey JG. The supraotic bone in neopterygian fishes (Osteichthyes, Actinopterygii). American Museum Novitates. 1999;3267:1-52.
40. López-Arbarello A, Wencker LCM. New callipurbeckiid genus (Ginglymodi: Semionotiformes) from the Tithonian (Late Jurassic) of Canjuers, France. Palaeontologische Zeitschrift. 2016;90(3):543-60.
41. Thies D. *Lepidotes gloriae* sp. nov. (Actinopterygii: Semionotiformes) from the Late Jurassic of Cuba. Journal of Vertebrate Paleontology. 1989;9(1):18-40.
42. Wenz S, Brito PM. New data about the lepisosteids and semionotids from the Early Cretaceous of Chapada do Araripe (NE Brazil): Phylogenetic implications. In: Arratia G, Schultze H-P, editors. Mesozoic Fishes 2 - Systematics and Fossil Record. München: Verlag Dr. Friedrich Pfeil; 1996. p. 153-65.
43. Hurley IA, Lockridge Mueller R, Dunn KA, Schmidt EJ, Friedman M, Ho RK, et al. A new time-scale for ray-finned fish evolution. Proceedings of the Royal Society B. 2007;274:489-98.
44. Rayner DH. The structure of certain Jurassic holostean fishes with special reference to their neurocrania. *Philosophical Transactions of the Royal Society London B*. 1948;233:287-345.
45. Jollie M. Development of cranial and pectoral girdle bones of *Lepisosteus* with a note on scales. Copeia. 1984;1984(2):476-502.

46. Wiley EO. The Phylogeny and Biogeography of Fossil and Recent Gars (Actinopterygii: Lepisosteidae). University of Kansas, Miscellaneous Publications. 1976;64:1-111.
47. Bartram AWH. The holostean fish genus *Ophiopsis* Agassiz. Zoological Journal of the Linnean Society. 1975;56:183-205.
48. Xu G, Wu F. A deep-bodied ginglymodian fish from the Middle Triassic of eastern Yunnan Province, China, and the phylogeny of lower neopterygians. Chinese Science Bulletin. 2012;57(1):111-8.
49. Cavin L, Deesri U, Suteethorn V. Osteology and relationships of *Thaiichthys* nov gen.: a Ginglymodi from the Late Jurassic - Early Cretaceous of Thailand. Palaeontology. 2013;56(1):183-208.
50. Xu G-H, Zhao L-J, Coates MI. The oldest ionoscopiform from China sheds new light on the early evolution of halecomorph fishes. Biology Letters. 2014;10(5).
51. Nielsen E. Studies on Triassic Fishes I: *Glaucolepis* and *Boreosomus*. Meddelelser om Grønland. 1942;138:1-394.
52. Stensiö E. Triassic fishes from east Greenland collected by the Danish Expeditions in 1929-1931. Meddelelser om Grønland. 1932;83(3):1-305.
53. López-Arbarello A, Sferco E. New semionotiform (Actinopterygii: Neopterygii) from the Late Jurassic of southern Germany. Journal of Systematic Palaeontology. 2011;9(2):197-215.
54. Bartram AWH. The Macrosemiidae, a Mesozoic family of holostean fishes. Bulletin of the British Museum (Natural History), Geology. 1977;29(2):137-234.
55. Schaeffer B. The Braincase of the Holostean Fish *Macrepistius*, with Comments on Neurocranial Ossification in the Actinopterygii. American Museum Novitates. 1971;2459:1-34.
56. Coates MI. Endocranial preservation of a Carboniferous actinopterygian from Lancashire, UK, and the interrelationships of primitive actinopterygians. Philosophical Transactions of the Royal Society of London. 1999;B 345:435-62.
57. Patterson C. Morphology and interrelationships of primitive actinopterygian fishes. American Zoology. 1982;22:241-59.
58. Giles S, Coates MI, Garwood RJ, Brazeau MD, Atwood R, Johanson Z, et al. Endoskeletal structure in *Cheirolepis* (Osteichthyes, Actinopterygii), An early ray-finned fish. Palaeontology. 2015;58(5):849-70.
59. Giles S, Xu GH, Near TJ, Friedman M. Early members of 'living fossil' lineage imply later origin of modern ray-finned fishes. Nature. 2017;549(7671):265-+.
60. Hilton EJ, Forey PL. Redescription of *Chondrosteus acipenseroides* Egerton, 1858 (Acipenseriformes, Chondrosteidae) from the lower Lias of Lyme Regis (Dorset, England), with comments on the early evolution of sturgeons and paddlefishes. Journal of Systematic Palaeontology. 2009:27 p.
61. Patterson C. The contribution of paleontology to teleostean phylogeny. In: Hecht MK, Goody PC, Hecht BM, editors. Major Patterns in Vertebrate Evolution: Plenum Press; 1977. p. 579-643.
62. Schröder KM, Lopez-Arbarello A, Ebert M. *Macrosemimimus*, gen. nov. (Actinopterygii, Semionotiformes), from the Late Jurassic of Germany, England, and France. Journal of Vertebrate Paleontology. 2012;32(3):512-29.
63. Brito PM, Alvarado-Ortega J. *Cipactlichthys scutatus*, gen. nov., sp nov a New Halecomorph (Neopterygii, Holostei) from the Lower Cretaceous Tlayua Formation of Mexico. Plos One. 2013;8(9).
64. Allis EP. The cranial anatomy of the mail-cheeked fishes. Zoologica. 1909;57(22):96 p.
65. Holmgren S, Stensiö E. Kranium und Visceralskelett der Akranier, Cyclostomen und Fische. In: Bolk L, Göppert E, Kallius E, Lubosch W, editors. Handbuch der vergleichenden Anatomie der Wirbeltiere. 4. Berlin und Wien: Urban und Schwrzenberg; 1936. p. 233–500.
66. Patterson C. Interrelationships of holosteans. In: Greenwood PH, Miles RS, Patterson C, editors. Interrelationships of fishes. London: Academic Press; 1973. p. 233-305.

67. Jollie M. Development of the head skeleton and pectoral girdle in Esox. Journal of Morphology. 1975;147:61-88.
68. Jollie M. A primer of bone names for the understanding of the actinopterygian head and pectoral girdle skeletons. Canadian Journal of Zoology. 1986;64:365-79.
69. Sanford CPJ. Salmonoid fish osteology and phylogeny (Teleostei: Salmonoidei). Theses Zoologicae. 2000;33:264 p.
70. Cavin L. Diversity of Mesozoic semionotiform fishes and the origin of gars (Lepisosteidae). Naturwissenschaften. 2010;97(12):1035-40.
71. Lopez-Arbarello A, Burgin T, Furrer H, Stockar R. New holostean fishes (Actinopterygii: Neopterygii) from the Middle Triassic of the Monte San Giorgio (Canton Ticino, Switzerland). Peerj. 2016;4:61.
72. López-Arbarello A, Codorniú L. Semionotids (Neopterygii, Semionotiformes) from the Lower Cretaceous Lagarcito Formation, San Luis Province, Argentina. Journal of Vertebrate Paleontology. 2007;27(4):811-26.
73. Sferco E, Lopez-Arbarello A, Maria Baez A. Anatomical description and taxonomy of *Luisiella feruglioi* (Bordas), new combination, a freshwater teleost (Actinopterygii, Teleostei) from the Upper Jurassic of Patagonia. Journal of Vertebrate Paleontology. 2015;35(3).
74. GQ L, MVH W. Phylogeny of Osteoglossomorpha. In: MLJ S, LD P, GD J, editors. Interrelationships of fishes. San Diego, USA: Academic Press; 1996. p. 163-74.
75. Cavin L, Giner S. A large halecomorph fish (Actinopterygii: Holostei) from the Valanginian (Early Cretaceous) of southeast France. Cretaceous Research. 2012;37:201-8.
76. López-Arbarello A. Revision of *Semionotus bergeri* Agassiz, 1833 (Upper Triassic, Germany), with comments on the taxonomic status of *Semionotus* (Actinopterygii, Semionotiformes). Palaeontologische Zeitschrift. 2008;82(1):40-54.
77. López-Arbarello A, Sun Z-Y, Sferco E, Tintori A, Xu G-H, Sun Y-L, et al. New species of *Sangiorgioichthys* Tintori and Lombardo, 2007 (Neopterygii, Semionotiformes) from the Anisian of Luoping (Yunnan Province, South China). Zootaxa. 2011(2749):25-39.
78. Alvarado-Ortega J, Espinosa-Arrubarrena L. A new genus of ionoscopiform fish (Halecomorphi) from the Lower Cretaceous (Albian) lithographic limestones of the Tlayúa quarry, Puebla, Mexico. Journal of Paleontology. 2008;82(1):163-75.
79. LI G-Q, WILSON MV, editors. Early divergence of Hiodontiformes sensu stricto in East Asia and phylogeny of some Late Mesozoic teleosts from China. Mesozoic fishes 2: systematics and fossil record; 1999; Munich, Germany: Verlag Dr Friedrich Pfeil.
80. Hilton EJ. Comparative osteology and phylogenetic systematics of fossil and living bony-tongue fishes (Actinopterygii, Teleostei, Osteoglossomorpha). Zoological Journal of the Linnean Society. 2003;137:1-100.
81. Schaeffer B. The Cretaceous holostean fish *Macrepistius*. American Museum Novitates. 1960;2011:1-18.
82. Machado GP, Alvarado-Ortega J, Machado LP, Brito PM. *Teoichthys brevipina*, sp nov., a new ophiopsid fish
(Halecomorphi, Ionoscopiformes) from the Lower Cretaceous Tlayua Formation,
Central Mexico. Journal of Vertebrate Paleontology. 2013;33(2):482-7.
83. Wenz S. †*Pliodetes nigeriensis*, gen. nov. et. sp. nov., a new semionotid fish from the Lower Cretaceous of Gadoufaoua (Niger Republic): phylogenetic comments. In: Arratia G, Schultze H-P, editors. Mesozoic Fishes 2 – Systematics and Fossil Record. München, Germany: Verlag Dr. Friederich Pfeil; 1999. p. 107-20.
84. Woodward AS. The fossil fishes of the English Wealden and Purbeck formations. Palaeontographical Society. 1916-1919;Part II:49-104.
85. Wenz S. Les *Lepidotes* (Actinopterygii, Semionotiformes) du Crétacé inférieur (Barrémien) de Las Hoyas (Province de Cuenca, Espagne). Geodiversitas. 2003;25(3):481-99.
86. Bermudez-Rochas DD, Poyato-Ariza FJ. A new semionotiform actinopterygian fish from the Mesozoic of Spain and its phylogenetic implications. Journal of Systematic Palaeontology. 2015;13(4):265-85.
87. Taverne L. Ostéologie et relations de *Catervariolus* (Teleostei,"Pholidophoriformes") du Jurassique moyen de Kisangani (Formation de Stanleyville) en République Démocratique

du Congo. Bulletin de l'Institut Royal des Sciences Naturelles de Belgique Sciences de la Terre. 2011;81:175-212.
88. López-Arbarello A, Alvarado-Ortega J. New semionotiform (Neopterygii) from the Tlayua Quarry (Early Cretaceous, Albian), Mexico. Zootaxa. 2011(2749):1-24.
89. Xu GH, Ma XY. A Middle Triassic stem-neopterygian fish from China sheds new light on the peltopleuriform phylogeny and internal fertilization. Science Bulletin. 2016;61(22):1766-74.
90. Nybelin O. On certain Triassic and Liassic representatives of the family Pholidophoridae s. str. Bulletin of the British Museum (Natural History),Geology. 1966;11(8):351-432.
91. Diogo R, Doadrio I, Vandewalle P. Teleostean Phylogeny Based on Osteological and Myological Characters. International Journal of Morphology. 2008;26(3):463-522.
92. Nielsen E. Studies on Triassic Fishes II: *Australosomus* and *Birgeria*. Meddelelser om Grønland. 1949;146(1):1-309.
93. Nybelin O. *Leptolepis dubia* aus den Torleiten-Schichten des Oberen Jura von Eichstätt. Paläontologische Zeitschrift. 1961;35(3/4):118-22.
94. Hubbs CL. A comparative study of the bones forming the opercular series of fishes. Journal of Morphology. 1919;33(1):61-72.
95. Griffith J, Patterson C. The structure and relationships of the Jurassic fish Ichthyokentema purbeckensis. Bulletin of the British Museum (Natural History), Geology. 1963;8(1):3-43.
96. Olsen PE. The skull and pectoral girdle of the parasemionotid fish *Watsonulus eugnathoides* from the Early Triassic Sakamena Group of Madagascar, with comments on the relationships of the holostean fishes. Journal of Vertebrate Paleontology. 1984;4(3):481-99.
97. Goodrich ES. Studies on the Structure and Development of Vertebrates. Journal of the History of Biology. 1930;21(2):355-6.
98. Gardiner BG, Schaeffer B, Masserie JA. A review of the lower actinopterygian phylogeny. Zoological Journal of the Linnean Society. 2005;144:511-25.
99. Wright RR. On the function of the serrated appendages of the throat of *Amia*. Science. 1884;4(96):511.
100. Jarvik E. On the exoskeletal shoulder-girdle of teleostomian fishes, with special reference to *Eusthenopteron goordi* Whiteaves. K svenska VetenskAkad Handl. 1944;21(7):1-32.
101. Liem KF, Woods LP. A probable homologue of the clavicle in the holostean fish *Amia calva*. Journal of Zoology. 1973;170(AUG):521-31.
