# Supplementary material for: Neopterygian phylogeny: the merger assay
Source: R Soc Open Sci. 2018 Mar 21;5(3):172337. doi: 10.1098/rsos.172337 (PMC5882744; doi:10.1098/rsos.172337)

Supplementary File S3. Branch support values obtained for the cladistic analyses.
to

Neopterygian Phylogeny: the merger assay

Adriana López-Arbarello[1]*, and Emilia Sferco[2]

[1]Department of Earth- and Environmental Sciences, Palaeontology and Geobiology and GeoBio-Center, Ludwig Maximilian University, Richard-Wagner-Strasse 10, D-80333 Munich, Germany (a.Lopez-Arbarello@lrz.uni-muenchen.de)

[2]CICTERRA-CONICET-UNC. Av. Velez Sarsfield 1611, X0516GCA, Córdoba, Argentina.

The cladistic analyses were performed with TNT under equal and implied weighting. The weighting strength was determined by using K-values of 3 and 8. Branch support was evaluated also with TNT applying bootstrap expressed as values of GC (groups present/contradicted) through 10000 replicates and calculating Bremer decay indexes for each node. Support measurements were calculated for implied (K=8 and K=3) and equal weighting analyses.

Equal weighting analyses:

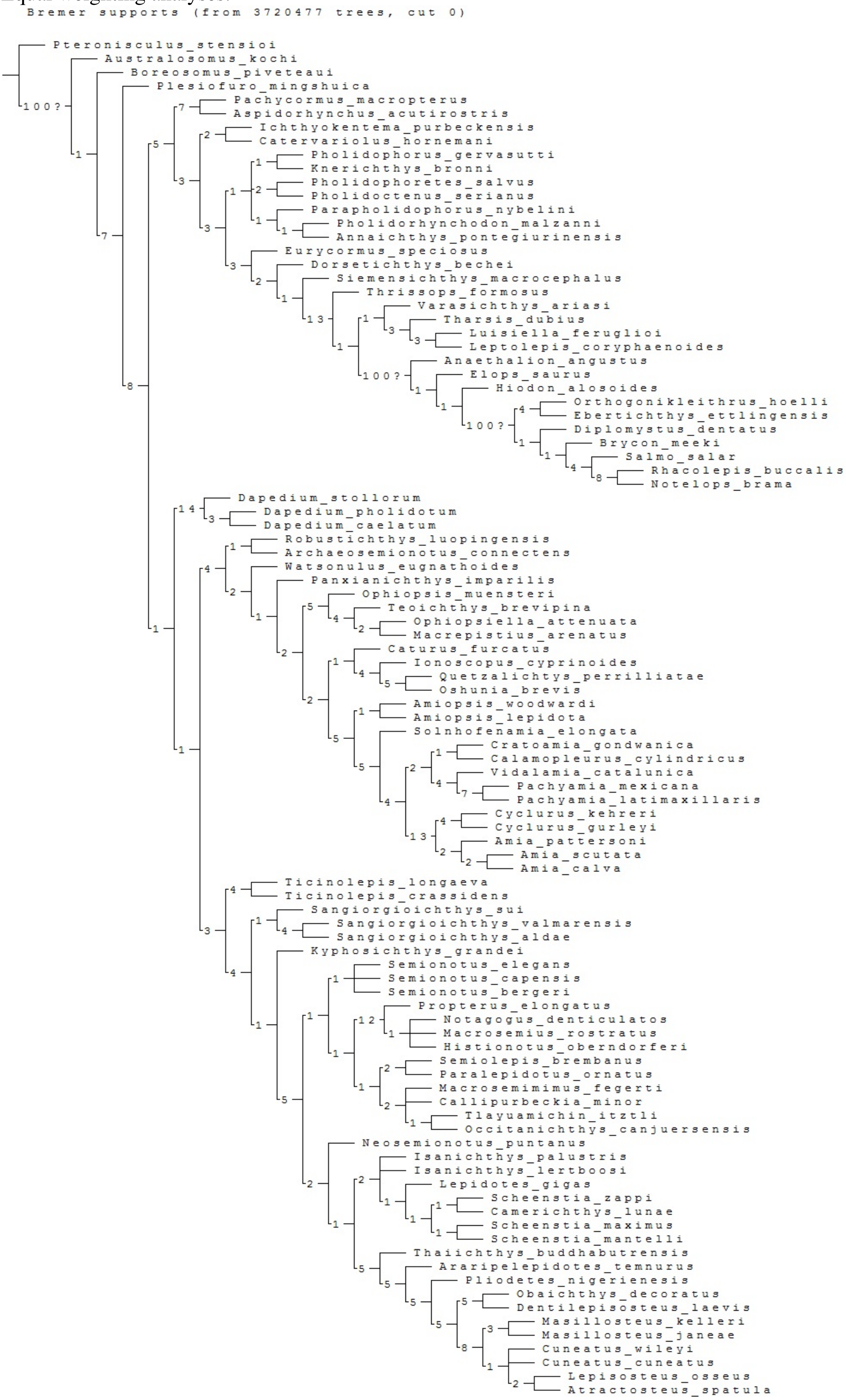

Equal weighting analyses:

GC values, 10000 replicates, cut=1 (tree 0) - Standard Bootstrap

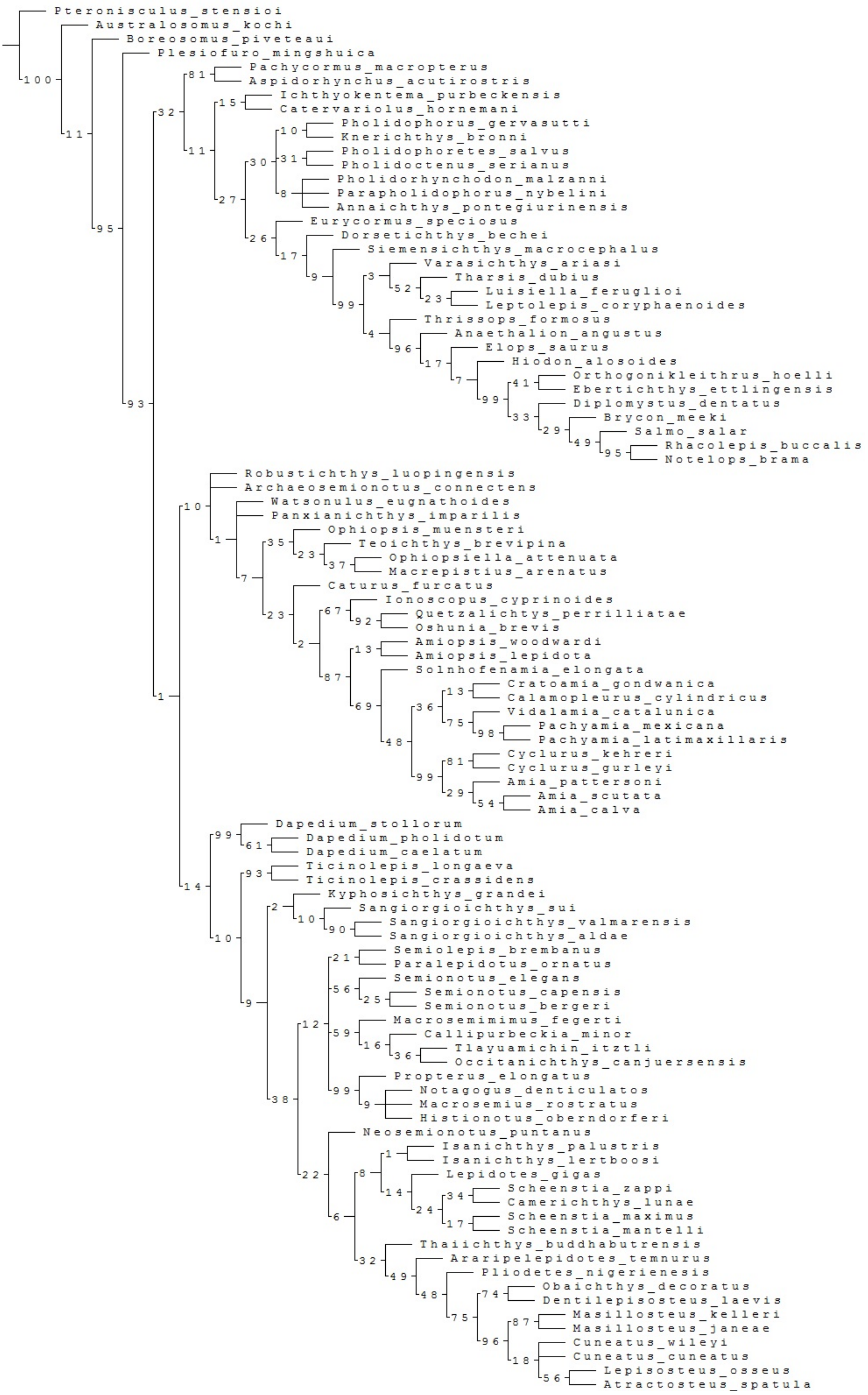

Implied weighting analyses (K = 3):

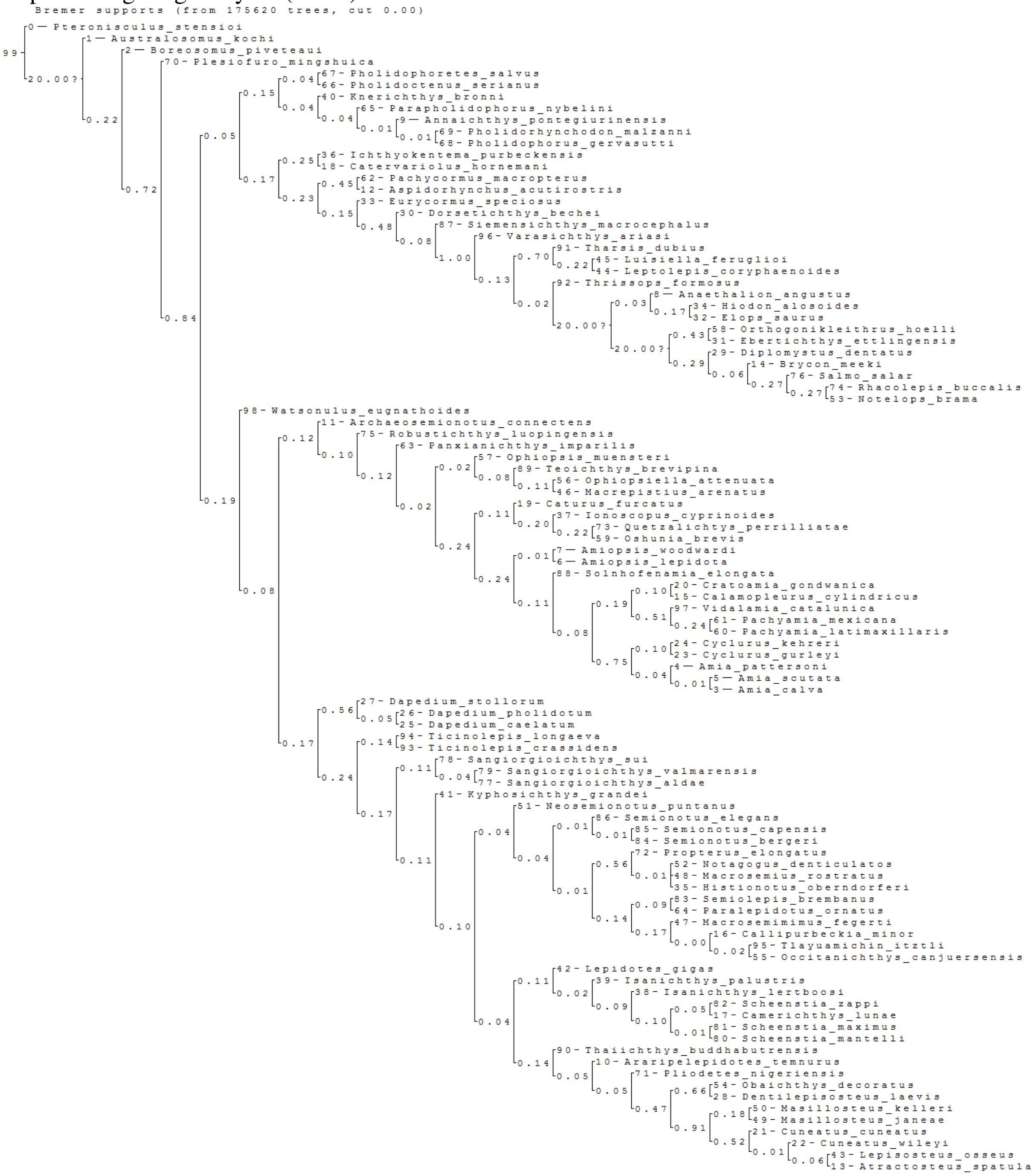

Implied weighting analyses (K = 3):

GC values, 1000 replicates, cut=1 (tree 0) - Standard Bootstrap

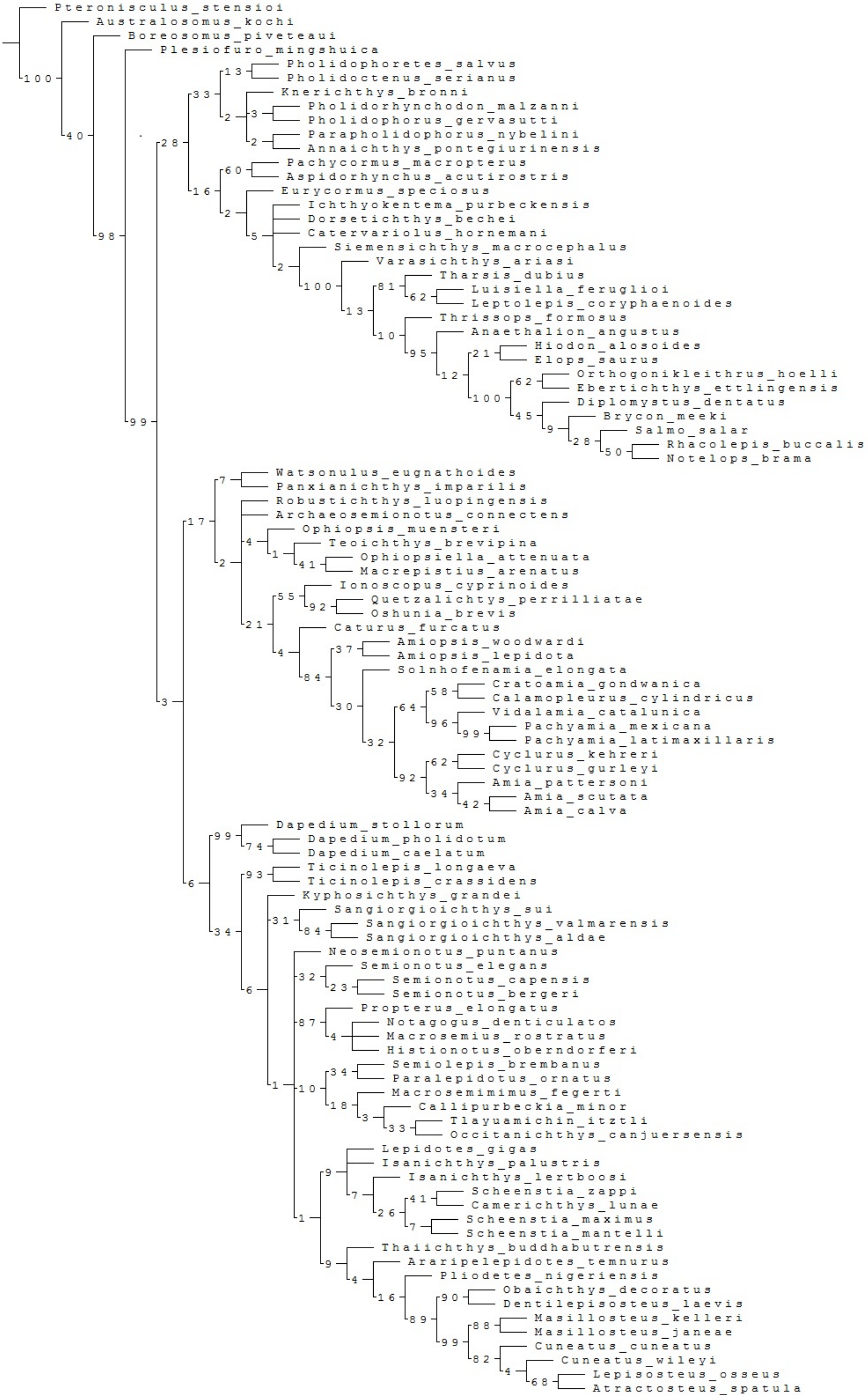

Implied weighting analyses (K = 8):

Bremer supports (from 176760 trees, cut 0.00)

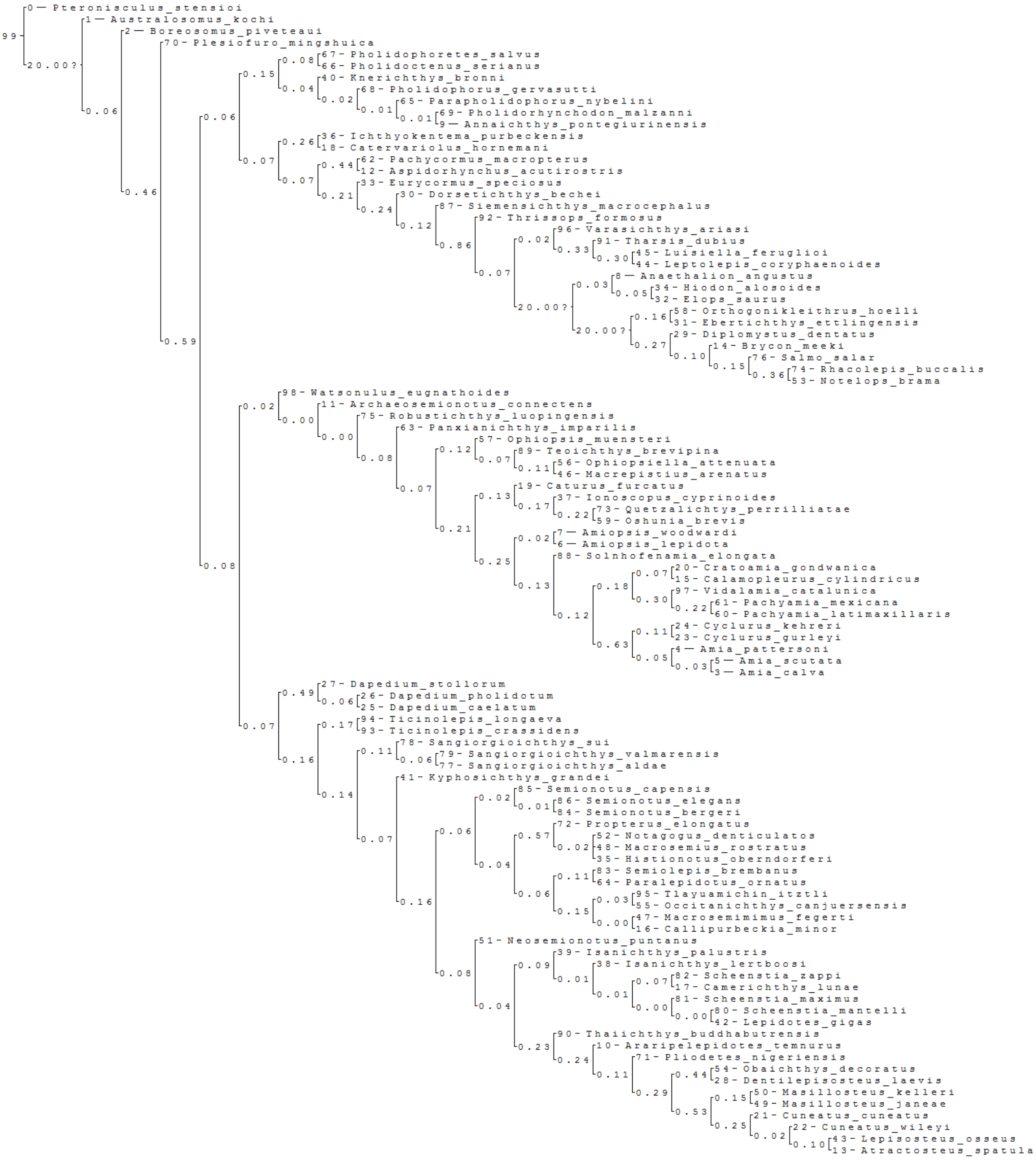

Implied weighting analyses (K = 8):

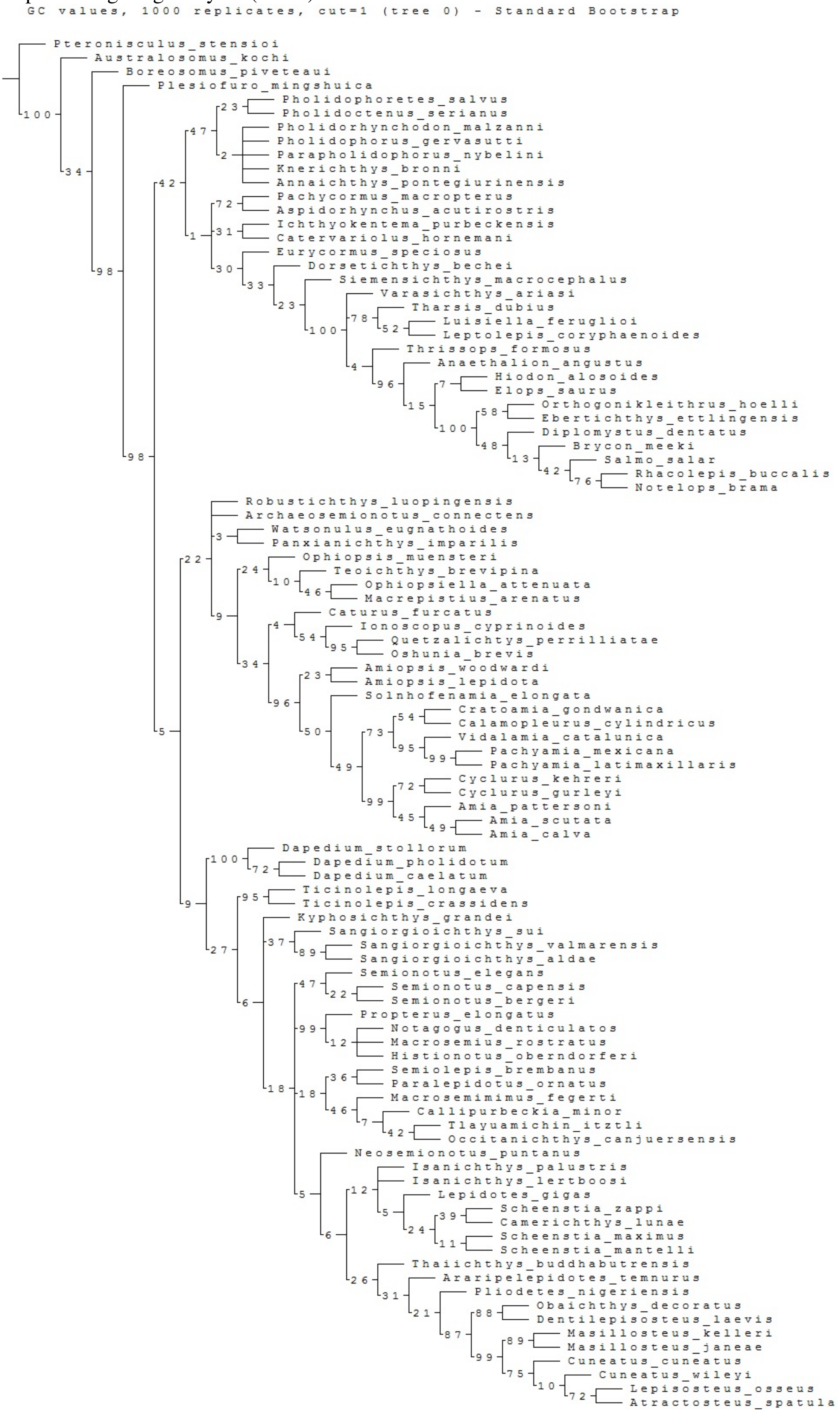

Supplement: Branch support [file rsos172337supp3.pdf]
