# Supplementary material for: Neopterygian phylogeny: the merger assay
Source: R Soc Open Sci. 2018 Mar 21;5(3):172337. doi: 10.1098/rsos.172337 (PMC5882744; doi:10.1098/rsos.172337)
Supplement: Optimization [file rsos172337supp4.pdf]

Crown-Neopterygii (Node 115):

Uniquely derived synapomorphies

1) Absence of postrostral bone (Char. 142: 0 --> 1)
2) Maxilla detached from preopercle (Char. 155: 0 --> 1)
3) Maxilla elongate and shallow (Char. 158: 0 --> 1)
4) Presence of supramaxilla (Char. 163: 0 --> 1)
5) Lower jaw with coronoid process (Char. 168: 0 --> 1)
6) Subopercle with ascending process (Char. 200: 0 --> 1)
7) Presence of interopercle (Char. 204: 0 --> 1)

Homoplastic synapomorphies

8) Absence of longitudinal articulation of scales (Char. 8: 1 --> 0)
9) Slightly concave ventral margin of maxilla (Char. 159: 3 --> 2)
10) Dorsal margin of maxilla generally straight or convex (Char. 162: 0 --> 1)
11) Dentary forming a well-developed process extending beyond the coronoid process (Char. 166: 0 --> 1)

Holostei (Node 114):

Homoplastic synapomorphies

1) Presence of four or more suborbital bones (Char. 144: 01 --> 3)
2) Presence of a presupracleithrum (Char. 319: 0 --> 1)

Potential synapomorphies

1) Four hypobranchials (211[1]; only in †*Pachycormus macrocephalus* outside Holostei)
2) Lateral dermethmoids forming the nasal processes of the premaxilla (48[0]; unique of Holostei)

†*Dapedium* (Node 164):

Homoplastic synapomorphies

1) Dorsal fin originating posterior to insertion of pelvic and extending backwards up to end of anal fin (Char. 1: 0 --> 5)
2) More than two pairs of extrascapulars (Char. 90: 0 --> 2)
3) Large supraorbital bones (Char. 117: 0 --> 1)
4) Large, keystone shaped dermosphenotic (Char. 119: 3 --> 4)
5) Edentulous maxilla (Char. 180: 0 --> 1)
6) Dentary tooth row present only on the anterior one third or less of dentary (Char. 183: 0 --> 1)
7) High, bluntly pencil like dentary teeth (Char. 184: 0 --> 2)
8) L-shape preopercle (Char. 187: 2 --> 3)
9) Suborbital bones completely overlap the dorsal limb of preopercle (Char. 194: 0 --> 1)
10) Convexly rounded caudal fin web (Char. 277: 0 --> 1)
11) One unbranched ray forming the ventral margin of the caudal fin (Char. 285: 0 --> 2)
12) 21 to 34 dorsal fin rays (Char. 300: 0 --> 1)
13) Bow-shaped or straight anal fin (Char. 306: 0 --> 2)
14) Low posttemporal, approximately as high as the dermopterotic (Char. 316: 0 --> 1)
15) Blunt anterior process of posttemporal (Char. 317: 0 --> 1)

Node 163:

Homoplastic synapomorphies

1) Ganoid scales with smooth posterior margin (Char. 6: 1 --> 0)
2) One hypaxial procurrent caudal fin ray (Char. 289: 2 --> 1)
3) 20 to 35 anal fin rays (Char. 305: 0 --> 1)

Ginglymodi + Halecomorphi (Node 113):

Uniquely derived synapomorphies

1) Antorbital bone with tapering canal bearing anterior arm (Char. 137: 0 --> 1)

Homoplastic synapomorphies (*=derived only within this clade)

2) *Supramaxillary notch (Char. 162: 1 --> 3)
3) *Branchiopercle (Char. 206: 0 --> 1)

4) Exposed surface of dermopterotic extending anterior to parietals by more than 40% but less than 50% of its total length (Char. 93: 3 --> 1)
5) Nasal bones not excavated by the posterior nostril (Char. 109: 0 --> 3)
6) Two supraorbital bones (Char. 115: 3 --> 2)
7) Maxilla with straight ventral margin (Char. 159: 2 --> 0)

Potential synapomorphies

1) Relatively small rostral, approximately rectangular to tube-like

Ginglymodi *sensu* López-Arbarello (2012) (Node 137):

Uniquely derived synapomorphies

1) Splint-like quadratojugal (Char. 72: 0 --> 1)
2) Anterior infraorbital bones (Char. 130: 0 --> 1)

Homoplastic synapomorphies

3) Ectopterygoid approximately crescent shape, convex dorsally, concave ventrally (Char. 62: 0 --> 1)
4) Antorbital bone excluded from the margin of the orbit (Char. 139: 1 --> 0)
5) Subopercle less than half the depth of the opercule (Char. 203: 0 --> 1)
6) Absence of gulars (Char. 208: 1 --> 0)

†*Ticinolepis* (Node 190):

Uniquely derived synapomorphies

1) The largest circumborbital bone is the infraorbital placed at the centre of the ventral margin of the orbit (Char. 125: 1 --> 5)

Homoplastic synapomorphies

2) Squamation of the ventrum made of numerous horizontal rows of distinctly shallow scales (Char. 10: 0 --> 1)
3) A mosaic of numerous suborbital bones (Char. 146: 0 --> 2)
4) Largest premaxillary teeth are smaller than dentary teeth (Char. 179: 0 --> 2)
5) Dorsal processes on the bases of innermost principal caudal fin rays (Char. 286: 0 --> 1)

(†*Sangiorgioichthys,* more derived ginglymodians) (Node 136):

Uniquely derived synapomorphies

1) Dorsal ridge of scales between the skull and the dorsal fin (Char. 11: 0 --> 1)
2) Anterior ceratohyal, deeply hourglass shape, without Beryciform foramen (Char. 81: 0 --> 3)

Homoplastic synapomorphies

3) Exposed area of dermopterotic not extending anterior to parietal (Char. 93: 1 --> 4)
4) Long and narrow nasal bones with anterior end incurved laterally (Char. 106: 0 --> 5)
5) Large premaxillary nasal processes, completely enclosing the olfactory fenestra (Char. 152: 0 --> 1)
6) Presupracleithrum absent (Char. 319: 1 --> 0)

†*Sangiorgioichthys* (Node 187):

Uniquely derived synapomorphies

1) Quadrate laterally covered by one or two suborbitals forming a triangular plate (Char. 150: 0 --> 1)

Homoplastic synapomorphies

2) Only one anterior infraorbital bone (Char. 131: 1 --> 0)
3) Dorsal margin of the maxilla gently concave allocating supramaxilla (Char. 162: 3 --> 2)
4) Coronoid process of the lower jaw is completely formed by the surangular (Char. 169: 0 --> 2)

(†*Sangiorgioichthys aldae,* †*Sangiorgioichthys valmarensis*) (Node 186):

Homoplastic synapomorphies

1) Ratio of frontals in adult sized individuals equal or larger than three (Char. 101: 0 --> 1)
2) Frontals tapering gradually (Char. 102: 0 --> 1)
3) Subinfraorbitals subrectangular, a quarter to half the size of the orbit (Char. 127: 2 --> 3)
4) Tooth row is present on only the anterior one third or less of dentary (Char. 183: 0 --> 1)

(†*Kyphosichthys grandei*, more derived ginglymodians) (Node 135):
Homoplastic synapomorphies
1) Longitudinal articulation of the scales of the body (Char. 8: 0 --> 1)
2) Subinfraorbitals subrectangular to triangular, about 1,5 to 2,5 times deeper than long (Char. 127: 2 --> 0)
3) Loss of branchiopercle (Char. 206: 1 --> 0)
4) Low posttemporal bone, approximately as high as the dermopterotic (Char. 316: 0 --> 1)

Neoginglymodi (Node 134):
Homoplastic synapomorphies
1) Dermal bones of the skull smooth or very slightly ornamented (Char. 3: 0 --> 1)
2) Absence of an ossified sclerotic ring (Char. 111: 0 --> 1)
3) Dorsal margin of maxilla generally straight or convex (Char. 162: 3 --> 1)
4) Separate retroarticular bone (Char. 174: 0 --> 1)
5) Absence of hypaxial procurrent caudal fin rays (Char. 289: 2 --> 0)
6) First principal anal fin ray branched (Char. 309: 1 --> 0)
7) Stout and increasing up to relatively high basal fulcra in the dorsal fin, higher than the basal segment of the first dorsal fin ray (Char. 311: 0 --> 2)

†Semionotiformes *sensu* López-Arbarello (2012) (Node 155):
Homoplastic synapomorphies
1) Absence of intercalar (Char. 33: 0 --> 1)
2) Ratio of frontals in adult sized individuals equal or larger than three (Char. 101: 0 --> 1)
3) Frontals tapering gradually (Char. 102: 0 --> 1)
4) The largest circumborbital bone is one of the anterior infraorbitals (Char. 125: 1 --> 7)
5) Minimum of three anterior infraorbitals (Char. 131: 1 --> 2)
6) One suborbital bone (Char. 144: 23 --> 0)
7) Absence of supraneural bones (Char. 237: 1 --> 0)
8) Maximum of 19 principal caudal fin rays (Char. 281: 1 --> 3)
9) Eight principal caudal fin rays below the lateral line (Char. 283: 0 --> 1)
10) Presence of serrated appendages (Char. 327: 0 --> 1)

†*Semionotus* (Node 189):
Homoplastic synapomorphies
1) Exposed surface of dermopterotic extending anterior to parietal by less than 30% of its total length (Char. 93: 4 --> 3)
2) Only one postorbital bone (Char. 124: 1 --> 0)
3) Tooth row is present on only the anterior one third or less of dentary (Char. 183: 0 --> 1)

(†Macrosemiidae, †Callipurbeckiidae) (Node 154):
Homoplastic synapomorphies
1) Ascending processes of the parasphenoid oriented perpendicularly (Char. 53: 2 --> 0)
2) Retroarticular bone included in the joint facet for quadrate (Char. 175: 0 --> 1)

†Macrosemiidae *sensu* López-Arbarello (2012) (Node 168):
Uniquely derived synapomorphies
1) Scroll-like subinfraorbital bones (Char. 127: 0 --> 4)
2) Dorsal fin divided in two sections (Char. 301: 0 --> 3)

Homoplastic synapomorphies
3) Dorsal fin originating anterior to insertion of pelvic and extending opposite to anal fins (Char. 1: 0 --> 3)
4) Well-developed peg-and-socket articulation (Char. 8: 1 --> 0)
5) Absence of Dorsal ridge of scales between the skull and the dorsal fin (Char. 11: 1 --> 0)
6) Absence of lateral ethmoids (Char. 46: 0 --> 1)
7) Tubular extrascapular bones (Char. 91: 0 --> 1)

8) Posterior extension of parietals median to the single pair of laterally placed extrascapular bones (Char. 92: 0 --> 1)
9) Frontal bones with tubular antorbital portion (Char. 102: 1 --> 3)
10) Approximately tubular nasal bones (Char. 106: 5 --> 2)
11) Widely separated nasal bones (Char. 107: 1 --> 2)
12) Minimum of four anterior infraorbital bones (Char. 131: 2 --> 3)
13) Tubular antorbital bone (Char. 136: 3 --> 5)
14) Absence of suborbital bones (Char. 143: 0 --> 1)
15) Slightly concave ventral margin of maxilla (Char. 159: 0 --> 2)
16) Absence of supramaxilla (Char. 163: 1 --> 0)
17) Posteroventral end of dentary not particularly expanded (Char. 166: 1 --> 0)
18) Posterior border of the preopercle notched ventrally (Char. 196: 0 --> 1)
19) Interopercle remote from mandible (Char. 205: 0 --> 1)
20) Supraorbital sensory canal does not enter in parietal (Char. 216: 0 --> 1)
21) No accessory row of scales adjacent to the ventral border of the body lobe (Char. 292: 1 --> 0)
22) 21 to 34 dorsal fin rays (Char. 300: 0 --> 1)
23) Anal fin bow-shaped or straight (Char. 306: 0 --> 2)
24) Ray-like basal fulcra in dorsal fin (Char. 311: 2 --> 3)
25) Absence of fringing fulcra in pectoral fin (Char. 334: 0 --> 1)

(†*Notagogous*, †*Macrosemius*, †*Histionotus*) (Node 167):
Homoplastic synapomorphies
1) Preopercle and dermopterotic are well separated by a space without bone (Char. 195: 0 --> 1)
2) Supratemporal commissure pierces extrascapulars and parietals (Char. 218: 0 --> 1)

†Callipurbeckiidae *sensu* López-Arbarello (2012) (Node 153):
Homoplastic synapomorphies
1) Dermal bones of the skull ornamented with tubercles or ridges (Char. 3: 1 --> 0)
2) Absence of marginal row of teeth on ectopterygoid (Char. 63: 1 --> 0)
3) Anterior/anteroventral end of preopercle finely tapered (Char. 189: 0 --> 1)
4) One unbranched ray rays forming the dorsal margin of the caudal fin (Char. 282: 2 --> 3)
5) One unbranched ray rays forming the ventral margin of the caudal fin (Char. 285: 1 --> 2)

(†*Semiolepis*, †*Paralepidotus*) (Node 184):
Homoplastic synapomorphies
1) More than four supraorbital bone (Char. 115: 1 --> 0)
2) Between 19 and 50 principal caudal fin rays (Char. 281: 3 --> 1)
3) More than eight principal caudal fin rays below the lateral line (Char. 283: 1 --> 0)
4) Two to five hypaxial procurrent caudal fin rays (Char. 289: 0 --> 2)
5) Slender basal fulcra in the dorsal fin, equalling or more slender than the basal segment of the first dorsal fin ray (Char. 311: 2 --> 0)

(†*Macrosemimimus*, †*Callipurbeckia* (†*Occitanichthys*, †*Tlayuamichin*)) (Node 152):
Homoplastic synapomorphies
1) Spiny, with a few small spines, posterior margin of ganoid scales (Char. 6: 1 --> 2)
2) Double longitudinal articulation of ganoid scales, anterior dorsal and ventral processes (Char. 9: 1 --> 2)
3) Most anterior anterior infraorbital narrowing dorsally, expanded ventrally, further ventral than the adjacent anterior infraorbitals (Char. 132: 0 --> 2)
4) Two suborbital bones (Char. 144: 0 --> 1)
5) Posterior border of maxilla at the anterior orbital margin (Char. 158: 1 --> 3)

(†*Occitanichthys*, †*Tlayuamichin*) (Node 180):
Homoplastic synapomorphies
1) Dorsal ridge scales with a low spine (Char. 12: 1 --> 0)
2) Three suborbital bones (Char. 144: 1 --> 2)

3) Large suborbital covering almost the whole area between the infraorbital bones and the preopercle (Char. 147: 1 --> 0)

(†*Neosemionotus*, Lepisosteiformes) (Node 133):
Homoplastic synapomorphies
1) Ganoid scales with smooth posterior margin (Char. 6: 1 --> 0)
2) Supraorbital bones large relative to orbit (Char. 117: 0 --> 1)
3) Large, keystone shaped dermosphenotic (Char. 119: 3 --> 4)
4) Concave posterior margin of caudal fin (Char. 277: 0 --> 2)
5) Lateral line emergin at the upper half of supracleithrum (Char. 321: 0 --> 1)

Lepisosteiformes *sensu* López-Arbarello (2012) (Node 132):
Uniquely derived synapomorphies
1) Broad kidney shaped nasal bones (Char. 106: 5 --> 1)
2) Most anterior supraorbital bone trapezoidal, longest ventrally, contacting more than one infraorbital bone (Char. 118: 0 --> 1)

Homoplastic synapomorphies
3) Two or more accessory row of scales adjacent to the ventral border of the body lobe (Char. 292: 1 --> 2)

(†*Isanichthys*, Lepidotidae) (Node 159):
Homoplastic synapomorphies
1) Dermal bones of the skull ornamented with tubercles or ridges (Char. 3: 1 --> 0)
2) The largest circumborbital bone is the dermosphenotic or a supraorbital (all these bones being of similar size) (Char. 125: 1 --> 3)
3) Suborbital bones occupy the area between the infraorbitals and preopercle posterior and below the orbit (Char. 145: 0 --> 1)
4) Pointed pencil like teeth on anterior coronoids (Char. 185: 0 --> 1)

Lepidotidae (Node 158):
Uniquely derived synapomorphies
1) Large and knob-like anteriro process of posttemporal bone (Char. 317: 1 --> 2)

Homoplastic synapomorphies
2) Ganoid scales with serrate or dentate posterior margin (Char. 6: 0 --> 1)
3) Frontals tapering gradually (Char. 102: 0 --> 1)
4) Ventral border of infraorbital series flexes abruptly dorsally at the anterior margin of the orbit (Char. 133: 0 --> 1)

Clade including †*Camerichthys* and †*Scheenstia* (Node 157):
Homoplastic synapomorphies
1) The most anterior and most dorsal suborbitals are notably larger than the other suborbitals (Char. 149: 0 --> 1)
2) Posterior border of maxilla at the anterior orbital margin (Char. 158: 1 --> 3)
3) Opercular ornamentation made of well-defined tubercles (Char. 199: 4 --> 1)

(†*Camerichthys lunae*, †*Scheenstia zappi*) (Node 156):
Homoplastic synapomorphies
1) Suprapreopercle present (Char. 197: 0 --> 1)
2) Subopercle is more than half de depth of the opercle (Char. 203: 1 --> 0)

†*Scheenstia maximus* + †*Scheenstia mantelli* (Node 188):
Homoplastic synapomorphies
1) Tooth row is present on only the anterior one third or less of dentary (Char. 183: 0 --> 1)

Lepisosteoidei *sensu* López-Arbarello (2012) (Node 131):
Uniquely derived synapomorphies

1) Parietals about half the length of frontals (Char. 100: 0 --> 1)

Homoplastic synapomorphies

2) Quadrate laterally covered by infraorbital bones (Char. 112: 0 --> 1)
3) Contact between preopercle and jugal or lower postinfraorbital (Char. 126: 0 --> 1)
4) Very short maxilla, does not reach the coronoid process (Char. 156: 1 --> 2)
5) Supramaxillae absent (Char. 163: 1 --> 0)
6) Dentary tooth row present only on the anterior one third or less of dentary (Char. 183: 0 --> 1)
7) L-shape preopercle (Char. 187: 2 --> 3)
8) Junction of supraorbital and temporal canals exclusively within dermopterotic bone (Char. 215: 1 --> 2)

Node 130:

Uniquely derived synapomorphies

1) Exoccipital placed anterior to basioccipital (Char. 28: 0 --> 2)

Homoplastic synapomorphies

2) Dorsal fin extending anterior to opposite of insertion of pelvic fins (Char. 1: 0 --> 2)
3) Ganoid scales with serrate or dentate posterior margin (Char. 6: 0 --> 1)
4) Absence of Dorsal ridge of scales between the skull and the dorsal fin (Char. 11: 1 --> 0)
5) Posteroventral end of dentary not particularly expanded (Char. 166: 1 --> 0)
6) Supraorbital sensory canal does not enter in parietal (Char. 216: 0 --> 1)
7) Maximum of 19 principal caudal fin rays (Char. 281: 1 --> 3)

Node 144:

Uniquely derived synapomorphies

1) Dermal bones of the skull ornamented with sharp tubercles (odontodes) resembling conical teeth (Char. 3: 1 --> 2)
2) Opercle ornamented with denticles (Char. 199: 4 --> 3)

Homoplastic synapomorphies

3) Double longitudinal articulation of ganoid scales, anterior dorsal and ventral processes (Char. 9: 2 --> 1)
4) The largest circumborbital bone is the dermosphenotic or a supraorbital (all these bones being of similar size) (Char. 125: 1 --> 3)
5) Ventral border of infraorbital series flexes abruptly dorsally at the anterior margin of the orbit (Char. 133: 0 --> 1)
6) Opercle approximately as deep as long (Char. 198: 0 --> 1)
7) Subopercle is more than half de depth of the opercle (Char. 203: 1 --> 0)

Lepisosteoidea *sensu* López-Arbarello (2012) (Node 143):

Uniquely derived synapomorphies

1) Ectopterygoid forms most of the palatal region (Char. 64: 0 --> 1)

Homoplastic synapomorphies

2) Dorsal fin originating approximately at the level of the origin of the anal fin and extending opposite to it (Char. 1: 2 --> 1)
3) Anterior ceratohyal elongate hourglass shape (Char. 81: 3 --> 1)
4) Frontals tapering gradually (Char. 102: 0 --> 1)
5) Large parapophyses fused to the centra (Char. 230: 0 --> 2)
6) All or most neural arches fused to the centra (Char. 233: 0 --> 1)
7) Very small posttemporal bones (Char. 316: 1 --> 2)

†Obaichthyidae (Node 165):

Uniquely derived synapomorphies

1) Sphenotic fused to dermosphenotic (Char. 42: 0 --> 1)

Homoplastic synapomorphies

2) Rostral region extends well anterior to the dentary symphysis by more than 50% of mandibular length (Char. 2: 0 --> 1)
3) More than two pairs of extrascapulars (Char. 90: 1 --> 2)

4) Parietals more than half the length of frontals (Char. 100: 1 --> 0)
5) Ratio of frontals in adult sized individuals equal or larger than three (Char. 101: 0 --> 1)
6) Quadrate is not laterally covered by infraorbital bones (Char. 112: 0 --> 1)
7) Small supraorbital bones (Char. 117: 1 --> 0)
8) Small dermosphenotic, not distinctly extended rostrally or caudally (Char. 119: 4 --> 3)
9) Dermosphenotic is excluded from orbital margin (Char. 120: 0 --> 1)
10) Anterior/anteroventral end of preopercle finely tapered (Char. 189: 0 --> 1)

Lepisosteidae *sensu* López-Arbarello (2012) (Node 142):

Uniquely derived synapomorphies

1) There is no dermosphenotic/sphenotic association (Char. 121: 0 --> 1)
2) Maxilla fused to toothed infraorbital bones (Char. 154: 0 --> 1)
3) Plicidentine structure in teeth (Char. 177: 0 --> 1)
4) Broad ascending process of the subopercle, more than 30% of the maximal length of the bone (Char. 201: 1 --> 0)

Homoplastic synapomorphies

5) Dermal bones of the skull ornamented with tubercles or ridges (Char. 3: 2 --> 0)
6) Ganoid scales with smooth posterior margin (Char. 6: 1 --> 0)
7) Suborbital bones completely overlap the dorsal limb of preopercle (Char. 194: 0 --> 1)
8) Opercle ornamentede with densely arranged low tubercles and/or ridges (Char. 199: 3 --> 0)
9) Interopercle absent (Char. 204: 1 --> 0)

†*Masillosteus* (Node 177):

Uniquely derived synapomorphies

1) Most anterior anterior infraorbital approximately rectangular, deeper than adjacent anterior infraorbital (Char. 132: 0 --> 3)

Homoplastic synapomorphies

2) Anterior coronoid teeth high, blunt, flattened or broadly rounded (Char. 185: 0 --> 2)
3) Vomerine teeth blunt, flattened or broadly rounded (Char. 186: 0 --> 2)

Lepisosteinae *sensu* Grande (2010) (Node 141):

Uniquely derived synapomorphies

1) Elongate ectopterygoid with bar-like anterior portion and deeply expanded posterior portion (Char. 62: 3 --> 4)
2) Part of dorsal surface of ectopterygoid ornamented and forming part of skull roof (Char. 65: 0 --> 1)
3) A series of toothed infraobordering the snout (Char. 134: 0 --> 1)

Homoplastic synapomorphies

4) Originating posterior to insertion of pelvic and extending backwards up to end of anal fin (Char. 1: 1 --> 4)
5) Length of parietals more than half the length of frontals (Char. 100: 1 --> 3)
6) Tooth row is present on only the anterior one third or less of dentary (Char. 183: 1 --> 0)

Lepisosteini *sensu* Grande (2010) (Node 140):

Uniquely derived synapomorphies

1) In addition to a lateral single row of similar sized dentary teeth, there is a medial row of much larger fangs (Char. 182: 0 --> 1)

Homoplastic synapomorphies

2) Lower jaw articulation placed in front of the orbit (Char. 69: 3 --> 4)
3) Length of parietals less than one half but more than one third the length of frontals (Char. 100: 3 --> 0)
4) Ratio of frontals in adult sized individuals equal or larger than three (Char. 101: 0 --> 1)
5) Subinfraorbitals subrectangular to triangular, about 1,5 to 2,5 times deeper than long (Char. 127: 2 --> 0)
6) Presence of serrated appendages (Char. 327: 0 --> 1)

Halecomorphi (Node 112):

Uniquely derived synapomorphies

1) Sensory canal in maxilla (Char. 220: 0 --> 1)

Homoplastic synapomorphies

2) Longitudinal articulation of the scales of the body (Char. 8: 0 --> 1)
3) Symplectic participates directly in the jaw joint (Char. 83: 0 --> 1)
4) The largest circumborbital bone is the jugal or the lacrimal, both with similar size (Char. 125: 1 --> 8)
5) Opercle ornamented with well-defined tubercles (Char. 199: 0 --> 1)

Node 138:

Homoplastic synapomorphies

1) Absence of quadratojugal (Char. 70: 0 --> 1)

Node 111:

Uniquely derived synapomorphies

1) Presence of a small postmaxillary process (Char. 161: 0 --> 1)

Homoplastic synapomorphies

2) Anterior ceratohyal narrow anteriorly, more expansive and laterally compressed posteriorly (Char. 81: 0 --> 2)
3) Frontals tapering gradually (Char. 102: 0 --> 1)
4) Posterodorsal corner of maxilla forming an acute angle (Char. 160: 0 --> 1)
5) Three postcleithra (Char. 325: 4 --> 3)

Node 110:

Uniquely derived synapomorphies

1) Rostral roughly V-shaped, with lateral horns and caudal triangular process (Char. 141: 0 --> 4)

Homoplastic synapomorphies

2) Ratio of frontals in adult sized individuals equal or larger than three (Char. 101: 0 --> 1)
3) Antorbital approximately tear-shape, broadest anteriorly, narrowing posteriorly (Char. 106: 0 --> 3)
4) Lateral line emerging at its upper half of supracleithrum Char. 322: 2 --> 1

Halecomorphi Section B of Grande & Bemis (1998) (Node 109):

Homoplastic synapomorphies

1) Absence of quadratojugal (Char. 70: 0 --> 1)
7) Subopercle less than half the depth of the opercle (Char. 203: 0 --> 1)
7) Presupracleithrum absent (Char. 319: 1 --> 0)

Node 176:

Homoplastic synapomorphies

1) Exposed area of dermopterotic not extending anterior to parietal (Char. 93: 1 --> 4)
2) Large preorbital portion of frontal, more than 1/3 of total length of frontal (Char. 104: 0 --> 1)
3) More than four supraorbital bones (Char. 115: 2 --> 0)
4) All supraorbitals small except the first, most anterior supraorbital, which is large, expanded anteriorly (Char. 117: 0 --> 2)
5) Presence of orbital canal (Char. 219: 0 --> 1)
6) Eight principal caudal fin rays below the lateral line (Char. 283: 0 --> 1)

†Ophiopsidae (Node 175):

Uniquely derived synapomorphies

1) Postinfraorbital bones subrectangular and elongated in posterodorsal to anteroventral direction (Char. 123: 0 --> 5)

Homoplastic synapomorphies

2) Dorsal fin originating anterior to insertion of pelvic and extending opposite to anal fins (Char. 1: 0 --> 3)
3) Frontals subrectangular not significantly narrower anteriorly than posteriorly (Char. 102: 1 --> 0)
4) Sickle- to L-shape antorbital bone (Char. 136: 2 --> 3)
5) Four or more, usually numerous suborbitals (Char. 144: 1 --> 3)
6) Absence of postmaxillary process (Char. 161: 1 --> 0)
7) Presence of lateral line ossicles between caudal fin rays (Char. 223: 0 --> 1)

Node 174:
Homoplastic synapomorphies
1) Dermosphenotic is excluded from orbital margin (Char. 120: 0 --> 1)
2) Smooth surface of arcocentra (Char. 229: 0 --> 2)
3) 21 to 34 dorsal fin rays (Char. 300: 0 --> 1)

Node 108:
Homoplastic synapomorphies
1) Dermal bones of the skull smooth or very slightly ornamented (Char. 3: 0 --> 1)
2) Elasmoid scales of amioid type (Char. 4: 0 --> 1)
3) Posterior extent of exoccipitals do not reach posterior margin of occiput (Char. 25: 0 --> 1)
4) Absence of pterotic bone (Char. 37: 0 --> 1)
5) Presence of few isolated urodermals (Char. 293: 0 --> 1)

(†*Caturus*, †Ionoscopidae) (Node 161):
Homoplastic synapomorphies
1) One or more, and notably large dermopterotic posterior projections (Char. 97: 0 --> 2)
2) Absence of supraneural bones (Char. 237: 1 --> 0)
3) Dorsal principal caudal fin rays oriented oblique to main axis of hypurals (Char. 280: 0 --> 1)
4) A single ventral scute at the base of the caudal fin (Char. 296: 2 --> 1)
5) First principal dorsal ray branched (Char. 304: 1 --> 0)
6) First principal anal fin ray branched (Char. 309: 1 --> 0)

†Ionoscopidae (Node 169):
Uniquely derived synapomorphies
1) Ventral surface of subinfraorbital bones intensely pitted (Char. 128: 0 --> 1)
Homoplastic synapomorphies
2) Absence of an ossified sclerotic ring (Char. 111: 0 --> 1)
3) Sickle- to L-shape antorbital bone (Char. 136: 2 --> 3)
4) Three suborbital bones (Char. 144: 1 --> 2)
5) Absence of diplospondylous centra in caudal region (Char. 228: 1 --> 0)
6) Large parapophyses separated from the centra (Char. 230: 0 --> 1)
7) Four or more ural neural spines (Char. 263: 4 --> 0)
8) Absence of hypaxial basal fulcra (Char. 291: 0 --> 1)

Node 181:
Uniquely derived synapomorphies
1) Inverted Y-shaped antorbital bone (Char. 106: 3 --> 6)
Homoplastic synapomorphies
2) Widely separated nasal bones (Char. 107: 1 --> 2)
3) Nasals placed lateral to frontals (Char. 108: 0 --> 1)
4) Numerous, 11 to 22 ural centra (Char. 258: 1 --> 0)
5) 21 to 34 dorsal fin rays (Char. 300: 0 --> 1)
6) Blunt anterior process of posttemporal (Char. 317: 0 --> 1)

Amiidae *sensu* Grande & Bemis (1998) (Node 107):
Uniquely derived synapomorphies

1) Gular maximal with to maximal length ratio between 0.3 and 0.5 (Char. 209: 0 --> 1)

Homoplastic synapomorphies

2) One suborbital bone (Char. 144: 1 --> 0)
3) Anteriorly projecting spine-like processes on caudal haemal arches (Char. 250: 0 --> 1)
4) Convexly rounded caudal fin web (Char. 277: 0 --> 1)
5) Ray-like basal fulcra in the dorsal fin (Char. 311: 0 --> 3)
6) Ray-like basal fulcra in the anal fin (Char. 312: 0 --> 3)
7) Absence of fringing fulcra on dorsal and anal fins (Char. 314: 0 --> 1)
8) Absence of fringing fulcra on pelvic fins (Char. 337: 0 --> 1)

†*Amiopsis* (Node 117):

Homoplastic synapomorphies

1) Absence of dorsal scute(s) preceding caudal fin (Char. 295: 2 --> 0)
2) More than one ventral scute(s) preceding caudal fin (Char. 296: 2 --> 0)

Amiida *sensu* Grande & Bemis (1998) (Node 106):

Homoplastic synapomorphies

1) Dorsal fin originating anterior to insertion of pelvic and extending opposite to anal fins (Char. 1: 0 --> 3)
2) Parietals less than one half but more than one third the length of frontals (Char. 100: 2 --> 0)
3) Absence of fringing fulcra on caudal fins (Char. 297: 0 --> 2)
4) 21 to 34 dorsal fin rays (Char. 300: 0 --> 1)
5) Bow-shaped dorsal fin (Char. 301: 0 --> 2)

Amiista *sensu* Grande & Bemis (1998) (Node 105):

Uniquely derived synapomorphies

1) Ascending processes of parasphenoid oriented anteriorly (Char. 53: 2 --> 1)
2) Two separate articular bones, not in contact with each other (Char. 171: 1 --> 2)

Homoplastic synapomorphies

3) Dermal bones of the skull ornamented with tubercles or ridges (Char. 3: 1 --> 0)
4) Large postorbital portion of frontal, more than 1/3 of total length of frontal (Char. 104: 0 --> 1)
5) Broad nasal bones, approximately rectangular to oval or trapezoidal (Char. 106: 3 --> 0)
6) The largest circumborbital bone is a postorbital occupying the posteroventral and posterior rim of the orbit (in some cases this area is occupied by one or more bones of similar size) (Char. 125: 1 --> 2)
7) Absence of suborbital bones (Char. 143: 0 --> 1)
8) Junction of supraorbital and temporal canals exclusively within frontal bone (Char. 215: 1 --> 0)

Amiinae *sensu* Grande & Bemis (1998) (Node 104):

Uniquely derived synapomorphies

1) Paired orbitosphenoid ossifications (Char. 44: 0 --> 1)
2) All but first hypural fused to the centra (Char. 269: 0 --> 1)
3) 43 or more dorsal fin rays (Char. 300: 1 --> 3)

Homoplastic synapomorphies

4) Length to width ratio of frontals is lower than three (Char. 101: 1 --> 0)
5) Nasal bones sutured to each other (Char. 107: 1 --> 0)
6) Absence of an ossified sclerotic ring (Char. 111: 0 --> 1)
7) Absence of supraorbital bones (Char. 113: 0 --> 1)
8) Dermosphenotic extended and tapering caudally, ending at the level of the anterior margin of the dermopterotic (or pterotic) (Char. 119: 3 --> 1)
9) Posterodorsal corner of maxilla rounded to straight angle (Char. 160: 1 --> 0)
10) Supramaxillary notch (Char. 162: 1 --> 3)
11) Middle pit-line leaving a groove or pore-line on the parietal and dermopterotic (Char. 217: 3 --> 0)

12) Large parapophyses fused to the centra (Char. 230: 0 --> 2)
13) Absence of urodermals (Char. 293: 1 --> 2)
14) Absence of scapulocoracoid ossification (Char. 328: 0 --> 1)

***Amia* (Node 103):**
Uniquely derived synapomorphies
Homoplastic synapomorphies
1) Absence of ossified ural neural arches (Char. 262: 3 --> 4)
2) Absence of dorsal scute(s) preceding caudal fin (Char. 295: 2 --> 0)
3) Lateral line emerging at the middle and supracleithrum (Char. 321: 1 --> 0)

Node 102:
Homoplastic synapomorphies
1) Dermopterotic (or pterotic) exposed surface extending anterior to parietal by more than 30% but less than 40% of its total length (Char. 93: 1 --> 2)
2) Opercle approximately as deep as long (Char. 198: 0 --> 1)

†*Cyclurus* (Node 162):
Homoplastic synapomorphies
1) Ascending processes of parasphenoid oriented perpendicular (Char. 53: 1 --> 0)
2) Dermopterotic (or pterotic) exposed surface extending posterior to parietal (Char. 94: 0 --> 1)
3) Parietal width to length ratio well exceeding 0.90 (Char. 99: 0 --> 1)
4) Posterior extent of maxilla up to posterior orbital margin (Char. 157: 0 --> 1)
5) Anterior coronoid teeth high, blunt, flattened or broadly rounded (Char. 185: 0 --> 2)
6) Vomerine teeth blunt, flattened or broadly rounded (Char. 186: 0 --> 2)

†Vidalaminae *sensu* Grande & Bemis (1998) (Node 151):
Homoplastic synapomorphies
1) Dentary teeth conical with labiolingually compressed, sharply carinate (keeled) caps (Char. 184: 0 --> 4)
2) Gular maximal with to maximal length ratio higher than 0.5 (Char. 209: 1 --> 0)
3) Numerous, 11 to 22 ural centra (Char. 258: 1 --> 0)
4) One ural neural spine (Char. 263: 4 --> 3)
5) Lateral edge of posttemporal elongate, about equal to or greater than width of anterior edge (Char. 318: 0 --> 1)
6) Absence of clavicles (Char. 326: 0 --> 1)

†Calamopleurini *sensu* Grande & Bemis (1998) (Node 150):
Homoplastic synapomorphies
1) Pu1 and U1 fused, forming a compound centrum (Char. 260: 0 --> 1)

†Vidalamiini *sensu* Grande & Bemis (1998) (Node 183):
Uniquely derived synapomorphies
1) Postmaxillary process notably large, posterior border of maxilla deeply excavated (Char. 161: 1 --> 2)
2) Distal ends of pleural ribs flatly truncated (Char. 231: 0 --> 1)
3) Basypterigium proximal end rodlike, without significant widening anteriorly (Char. 336: 0 --> 1)

Homoplastic synapomorphies
4) Supramaxilla short, about a quarter of the length of the maxilla (Char. 164: 0 --> 1)
5) Opercle longer than deep (Char. 198: 0 --> 3)

†*Pachyamia sensu* Grande & Bemis (1998) (Node 182):
Homoplastic synapomorphies
1) Dermopterotic (or pterotic) exposed surface extending anterior to parietal by more than 50% of its total length (Char. 93: 1 --> 0)

2) Parietals less than one third the length of frontals (Char. 100: 0 --> 2)
3) Presence of an interfrontal fontanelle (Char. 105: 0 --> 1)
4) Nasal bones sutured to each other (Char. 107: 1 --> 0)
5) Dermosphenotic extended and tapering caudally, ending at the level of the anterior margin of the dermopterotic (or pterotic) (Char. 119: 3 --> 1)
6) Dermosphenotic is excluded from orbital margin (Char. 120: 0 --> 1)
7) Absence of ossified ural neural arches (Char. 262: 3 --> 4)

Teleostei (Node 126):

Uniquely derived synapomorphies

1) Orbitosphenoid confined to the dorsal portion of the orbit (Char. 45: 0 --> 1)
2) Quadratojugal fused to quadrate (Char. 71: 0 --> 1)
3) Presence of median basibranchial tooth plates (Char. 210: 0 --> 2)

Homoplastic synapomorphies

4) Dermal bones of the skull smooth or very slightly ornamented (Char. 3: 0 --> 1)
5) Ganoid scales with smooth posterior margin (Char. 6: 1 --> 0)
6) Exit of the vagus nerve (X) placed between intercalar and exoccipital (Char. 24: 0 --> 1)
7) Dermal component of intercalar (Char. 34: 0 --> 1)
8) Parietals less than one third the length of frontals (Char. 100: 0 --> 2)
9) Approximately quadrangular postinfraorbital bones (Char. 123: 0 --> 1)
10) Opercle ornamentation weak or absent (Char. 199: 0 --> 4)
11) Presence of a chordacentrum (Char. 226: 0 --> 1)
12) Dorsal principal caudal fin rays oriented oblique to main axis of hypurals (Char. 280: 0 --> 1)

(†*Aspidorhynchus*, †*Pachycormus*) (Node 139):

Uniquely derived synapomorphies

1) Supramaxilla placed posterodorsal to the maxilla (Char. 165: 0 --> 1)
2) Absence of epural bones (Char. 264: 1 --> 0)

Homoplastic synapomorphies

3) Opercular process of hyomandibula notably elongated (Char. 87: 0 --> 1)
4) The largest infraorbital is the lacrimal or infraorbital placed at the anteroventral corner of the orbit (Char. 125: 1 --> 6)
5) Antorbital bone excluded from the margin of the orbit (Char. 139: 1 --> 0)
6) Independent of the total number, there is a large suborbital covering almost the whole area between the infraorbital bones and the preopercle (Char. 147: 0 --> 1)
7) Maxilla extremely slender (Char. 158: 1 --> 2)
8) Supramaxilla short, about a quarter of the length of the maxilla (Char. 164: 0 --> 1)
9) Retroarticular present and fused to articular only (Char. 174: 0 --> 3)
10) Arcocentrum present and thin, ring-like centra (Char. 225: 0 --> 1)
11) 'Z' or step-like segmentation of marginal principal caudal fin rays (Char. 288: 0 --> 1)
12) Epaxial basal fulcra present as procurrent rays (Char. 290: 0 --> 1)
13) Lateral line emergin at the upper half of supracleithrum (Char. 321: 0 --> 1)

Node 125:

Uniquely derived synapomorphies

1) Symplectic shape slightly curved tube or splint (Char. 85: 1 --> 0)
2) Antorbital bone approximately triangular or drop-like, narrowing posterodorsally (Char. 136: 1 --> 0)

Homoplastic synapomorphies

3) Posterior end of the fossa parampullaris of Bjerring (1984) in autopterotic (Char. 14: 0 --> 2)
4) Well-developed basipterygoid processes of parasphenoid (Char. 51: 0 --> 1)
5) Lower jaw articulation placed around the level of the posterior border of the orbit (Char. 69: 0 --> 1)
6) Anterodorsal ascending margin of the dentary interrupted by a characteristic notch (so-called leptolepid notch) (Char. 167: 0 --> 1)

Node 160 :
Homoplastic synapomorphies
1) Presence of supraoccipital bone (Char. 17: 0 --> 1)
2) Ascending processes of the parasphenoid oriented perpendicularly (Char. 53: 2 --> 0)
3) Absence of marginal row of teeth on ectopterygoid (Char. 63: 1 --> 0)
4) Nasal bones sutured to each other (Char. 107: 2 --> 0)
5) Three or four supraorbital bones (Char. 115: 3 --> 1)
6) Junction of supraorbital and temporal canals no direct connection between the temporal (otic) and supraorbital sensory canals (Char. 214: 1 --> 0)
7) Median neural spines on abdominal vertebrae (Char. 234: 0 --> 1)

Node 124:
Uniquely derived synapomorphies
1) Two supramaxillae (Char. 163: 1 --> 2)
2) Gular maximal with to maximal length ratio lower than 0.3 (Char. 209: 0 --> 2)

Homoplastic synapomorphies
3) Presence of a parasphenoid tooth patch (Char. 56: 1 --> 0)
4) Posterior nostril completely included in the posterior portion of the nasals (Char. 109: 0 --> 1)
5) Maxilla very long, extends beyond the coronoid process (Char. 156: 1 --> 0)
6) Preopercle and dermopterotic are well separated by a space without bone (Char. 195: 0 --> 1)
7) 10 to 15 branchiostegal rays (Char. 207: 3 --> 2)

Pholidophoriformes (Node 129):
Homoplastic synapomorphies
1) Absence of pterotic bone (Char. 37: 0 --> 1)
2) Absence of lateral dermethmoids (Char. 47: 1 --> 0)
3) Symplectic participates directly in the jaw joint (Char. 83: 0 --> 1)
4) Two supraorbital bones (Char. 115: 3 --> 2)
5) Supraorbital sensory canal does not enter in parietal (Char. 216: 0 --> 1)
6) Presence of a pectoral axillary process (Char. 335: 0 --> 1)
7) Spatulate fin rays only in pectoral fins (Char. 339: 0 --> 1)

Node 128:
Uniquely derived synapomorphies
1) Anterior notch of preopercle (Char. 193: 0 --> 1)

Homoplastic synapomorphies
2) Lower jaw articulation at the level of the centre of the orbit (Char. 69: 1 --> 2)
3) Largest premaxillary teeth larger than dentary teeth (Char. 179: 0 --> 1)

Node 127:
Homoplastic synapomorphies
1) Opercular ornamentation made of well-defined tubercles (Char. 199: 4 --> 1)
2) One accessory row of scales adjacent to the ventral border of the body lobe (Char. 292: 0 --> 1)

Node 170:
Homoplastic synapomorphies
1) Independent of the total number, there is a large suborbital covering almost the whole area between the infraorbital bones and the preopercle (Char. 147: 0 --> 1)
2) Opercular ornamentation made of densely arranged low tubercles and/or ridges (Char. 199: 4 --> 0)
3) Tiny ascending process of subopercle, its base being less than 10% of the maximal length of the bone (Char. 201: 1 --> 2)

Node 185:

Homoplastic synapomorphies

1) Extrascapular bones semicircular, expanded caudolaterally (Char. 91: 0 --> 2)
2) Antorbital approximately tear-shape, broadest anteriorly, narrowing posteriorly (Char. 106: 0 --> 3)
3) Nasal bones broad kidney shaped (Char. 107: 2 --> 1)
4) Posterodorsal corner of maxilla forming an acute angle (Char. 160: 0 --> 1)

Node 123:

Uniquely derived synapomorphies

1) Posteroventral angle of an L-shape preopercle expanded fan shape (Char. 191: 0 --> 1)

Homoplastic synapomorphies

2) Independent of the total number, there is a large suborbital covering almost the whole area between the infraorbital bones and the preopercle (Char. 147: 0 --> 1)
3) L-shape preopercle (Char. 187: 2 --> 3)
4) Low ascending process of preopercle, its height being 20% of the maximal length of the bone (Char. 202: 1 --> 0)
5) Preopercular sensory canal with 12 or more long, simple or branched tubules (Char. 221: 1 --> 2)
6) Epineural bones present (Char. 239: 0 --> 1)
7) Neural spine on second preural vertebra (Pu2) shorter than neural spine on third preural vertebra (Char. 246: 0 --> 1)
8) Two fin rays, at least the first unbranched, forming the dorsal margin of the caudal fin (Char. 282: 3 --> 1)
9) Dorsal processes of the bases of innermost principal caudal fin rays (Char. 286: 0 --> 1)
10) 'Z' or step-like segmentation of marginal principal caudal fin rays (Char. 288: 0 --> 1)
11) Lateral line emerging at the upper half of supracleithrum (Char. 321: 0 --> 1)

Node 122:

Uniquely derived synapomorphies

1) Presence of urohyal (Char. 75: 0 --> 1)
2) Exposed surface of dermopterotic resembling an inverted L (Char. 95: 0 --> 1)
3) Rostral bone relatively large and roughly rounded or rectangular-shaped without lateral horns (Char. 141: 2 --> 3)

Homoplastic synapomorphies

4) Presence of supraoccipital bone (Char. 17: 0 --> 1)
5) Absence of a notch in the anterodorsal ascending margin of the dentary (Char. 167: 1 --> 0)

Node 121:

Homoplastic synapomorphies

1) Absence of suborbital bones (Char. 143: 0 --> 1)
2) Arcocentrum present and thin, ring-like centra (Char. 225: 0 --> 1)
3) Absence of diplospondylous centra in caudal region (Char. 228: 1 --> 0)
4) Epaxial basal fulcra present as procurrent rays (Char. 290: 0 --> 1)

Node 120:

Uniquely derived synapomorphies

1) Elasmoid scales of cycloid type (Char. 4: 0 --> 2)
2) Lateral dermethmoids forming part of a compound mesethmoid with chondral and dermal components (Char. 48: 1 --> 3)
3) Well-developed postarticular process of lower jaw, extending posterior to the articular facet for quadrate (Char. 172: 0 --> 1)
4) Presence of autocentrum (Char. 227: 0 --> 1)

Homoplastic synapomorphies

5) Absence of canal or groove for the dorsal aorta in the surface of the basioccipital (Char. 32: 1 --> 2)
6) Absence of opisthotic (Char. 36: 0 --> 1)

7) Dermopterotic (or pterotic) exposed surface extending posterior to parietal (Char. 94: 0 --> 1)
8) Absence of surangular (Char. 170: 1 --> 0)
9) Anteromedial wing of preopercle absent (Char. 192: 1 --> 0)
10) Absence of fringing fulcra on dorsal and anal fins (Char. 314: 0 --> 1)
11) Cleithrum sickle with anterior portion bent downwards (Char. 322: 2 --> 3)
12) Cleithrum not ornamented (Char. 324: 1 --> 0)
13) Absence of fringing fulcra in pectoral fin (Char. 334: 0 --> 1)
14) Absence of fringing fulcra on pelvic fins (Char. 337: 0 --> 1)

Node 119:
Uniquely derived synapomorphies
1) Epipleural bones (Char. 240: 0 --> 1)
Homoplastic synapomorphies
2) Absence of posterior projection(s) of dermopterotic (Char. 97: 1 --> 0)
3) Absence of 'posterior uroneurals' (Char. 274: 1 --> 0)
4) One unbranched ray rays forming the dorsal margin of the caudal fin (Char. 282: 1 --> 3)
5) One unbranched ray forming the ventral margin of the caudal fin (Char. 285: 0 --> 2)
6) Bases of innermost principal caudal fin rays narrow, with pointed proximal end (Char. 287: 0 --> 1)

Node 173:
Homoplastic synapomorphies
1) Anterodorsal ascending margin of the dentary interrupted by a characteristic notch (so-called leptolepid notch) (Char. 167: 0 --> 1)
2) Epipleural bones in abdominal and anterior caudal region (Char. 242: 0 --> 1)
3) Absence of hypaxial basal fulcra (Char. 291: 0 --> 1)
4) Fist anal pterygiophore posterior to first haemal spine or infrahaemal (Char. 307: 1 --> 0)

Leptolepid-clade (Node 172):
Uniquely derived synapomorphies
1) Preopercular process of hyomandibula (Char. 86: 0 --> 1)
2) Preural neural spines broad with a median groove (Char. 243: 0 --> 1)
3) Preural haemal spines broad with a median groove (Char. 244: 0 --> 1)
Homoplastic synapomorphies
4) Retroarticular bone included in the joint facet for quadrate (Char. 175: 0 --> 1)
5) Anteriorly projecting spine-like processes on caudal neural arches (Char. 249: 0 --> 1)
6) First and second hypurals fused at their bases only (Char. 268: 0 --> 1)
7) Absence of space or diastema between second and third hypurals (Char. 270: 1 --> 0)

Node 171:
Homoplastic synapomorphies
1) Dermopterotic (or pterotic) exposed surface not extending posterior to parietal (Char. 94: 1 --> 0)
2) Posteroventral angle of an L-shape preopercle not distinctly expanded (Char. 191: 1 --> 0)
3) Tiny ascending process of subopercle, its base being less than 10% of the maximal length of the bone (Char. 201: 1 --> 2)
4) Subopercle is more than half de depth of the opercle (Char. 203: 1 --> 0)
5) Smooth autocentra (Char. 330: 1 --> 0)
6) Anterior supraneurals not expanded, simple rod-like (Char. 238: 1 --> 0)
7) Epipleural bones absent (Char. 240: 1 --> 0)

Teleocephala (Node 118):
Homoplastic synapomorphies
1) Basipterygoid processes of parasphenoid almost or totally absent (Char. 51: 1 --> 0)
2) Absence of parasphenoid tooth patch (Char. 56: 0 --> 1)
3) Dentary teeth of similar size arranged in two or more rows and (Char. 182: 0 --> 2)

4) Seven hypurals (Char. 267: 0 --> 1)
5) Fist uroneural extent up to preural centrum 2(Char. 275: 0 --> 1)
6) Two uroneurals extending forwards beyond second ural centrum (Char. 276: 0 --> 1)
7) Absence of dorsal processes of the bases of innermost principal caudal fin rays (Char. 286: 1 --> 0)
8) Lateral line emerging at the middle (Char. 322: 3 --> 0)
9) Medial wing of cleithrum absent (Char. 323: 1 --> 0)

Node 149:

Homoplastic synapomorphies

1) Ascending process of subopercle of medium height, 20-40% of the maximal length of the bone (Char. 202: 0 --> 1)
2) Absence of ural neural spines (Char. 263: 3 --> 4)
3) Three or less uroneurals (Char. 273: 1 --> 2)
4) Simple first dorsal proximal pterygiophore (Char. 302: 1 --> 0)

Node 148:

Uniquely derived synapomorphies

1) Bar-like ectopterygoid (Char. 62: 2 --> 5)
2) Tooth plates present on infrapharyngobranchials 1 to 3 (Char. 212: 2 --> 3)

Homoplastic synapomorphies

3) Intercalar without extensive dermal outgrowths (Char. 35: 1 --> 0)
4) Rostral bone absent, no trace of ethmoidal commissure (Char. 140: 2 --> 4)
5) Absence of gulars (Char. 208: 1 --> 0)
6) Preural haemal arches (excluding the parhypural) fused to the centra (Char. 256: 0 --> 1)

Clupeocephala (Node 147):

Homoplastic synapomorphies

1) Maxilla extent up to centre of the orbit (Char. 157: 1 --> 2)
2) Posteroventral angle of an L-shape preopercle not distinctly expanded (Char. 191: 1 --> 0)
3) Preopercular senory canal with four or less, short simple tubules (Char. 221: 1 --> 0)
4) Epipleural bones in abdominal and anterior caudal region (Char. 242: 0 --> 1)
5) Six or less hypurals (Char. 267: 1 --> 2)
6) One to three principal caudal fin rays associated with the preural skeleton (Char. 284: 3 --> 4)

Node 166:

Uniquely derived synapomorphies

1) Preural neural spines broad due to anterior and posterior membranous outgrowths (Char. 243: 0 --> 2)
2) Preural haemal spines broad due to anterior and posterior membranous outgrowths (Char. 244: 0 --> 2)

Homoplastic synapomorphies

3) Anteriorly projecting spine-like processes on caudal neural arches (Char. 249: 0 --> 1)
4) First and second hypurals fused at their bases only (Char. 268: 0 --> 1)
5) Bipartite or tripartite first dorsal proximal pterygiophore (Char. 302: 1 --> 0)
6) Cleithrum sickle with anterior portion bent downwards (Char. 322: 0 --> 3)
7) Medial wing of cleithrum absent (Char. 323: 0 --> 1)

Node 146:

Uniquely derived synapomorphies

1) Splint-like antorbital bone (Char. 136: 0 --> 6)

Homoplastic synapomorphies

2) Tubular extrascapular bones (Char. 91: 0 --> 1)
3) First uroneural extend anteriorly up to preural centrum 1 (Char. 275: 1 --> 2)

Node 145:

Uniquely derived synapomorphies

1) Lateroparietal skull roof (Char. 98: 0 --> 1)

Homoplastic synapomorphies

2) Presence of a supraoccipital crest (Char. 18: 0 --> 1)
3) Presence of a parasphenoid tooth patch (Char. 56: 1 --> 0)
4) Straight or almost straight ventral margin of maxilla (Char. 159: 1 --> 0)
5) Presence of 'posterior uroneurals' (Char. 274: 0 --> 1)

Node 179:

Uniquely derived synapomorphies

1) High and triangular supraoccipital crest (Char. 19: 1 --> 0)

Homoplastic synapomorphies

2) Dorsal fin extending anterior to opposite of insertion of pelvic fins (Char. 1: 0 --> 2)
3) Parasphenoid long, reaching the occipital condyle or close to it (Char. 50: 1 --> 2)
4) Very large dermosphenotic, extended and tapering caudally, reaching close to the level of the preopercular canal (Char. 119: 0 --> 2)
5) Presence of a rostrodermethmoid (Char. 140: 4 --> 3)
6) Junction of supraorbital and temporal canals no direct connection between the temporal (otic) and supraorbital sensory canals (Char. 214: 1 --> 0)
7) Epipleural bones absent (Char. 240: 1 --> 0)

Node 178:

Uniquely derived synapomorphies

1) Premaxillary teeth organized in a single row plus anterior fang (Char. 178: 0 --> 2)

Homoplastic synapomorphies

2) Posterior process of epioccipital (Char. 21: 0 --> 1)
3) Ectopterygoid approximately boomerang shape (Char. 62: 5 --> 2)
4) Semicircular extrascapulars, expanded caudolaterally (Char. 91: 1 --> 2)
5) Absence of antorbital bone (Char. 135: 0 --> 1)
6) Maxilla extremely slender (Char. 158: 1 --> 2)
7) Posterodorsal corner of maxilla forming an acute angle (Char. 160: 0 --> 1)
4) Largest premaxillary teeth larger than dentary teeth Char. 179: 0 --> 1
5) Neural spine on second preural vertebra (Pu2) shorter than neural spine on third preural vertebra (Char. 246: 0 --> 1)
6) First and second hypurals fused to each other along their length (Char. 268: 0 --> 2)
